# Unsymmetrically-Substituted 5,12-dihydrodibenzo[*b*,*f*][1,4]diazocine-6,11-dione Scaffold—A Useful Tool for Bioactive Molecules Design

**DOI:** 10.3390/molecules25122855

**Published:** 2020-06-20

**Authors:** Bartosz Bieszczad, Damian Garbicz, Damian Trzybiński, Marta K. Dudek, Krzysztof Woźniak, Elżbieta Grzesiuk, Adam Mieczkowski

**Affiliations:** 1 Institute of Biochemistry and Biophysics, Polish Academy of Sciences, 02-106 Warszawa, Poland; b.bieszczad@ibb.waw.pl (B.B.); dgarbicz@ibb.waw.pl (D.G.); elag@ibb.waw.pl (E.G.); 2 Biological and Chemical Research Centre, University of Warsaw, 02-089 Warszawa, Poland; dtrzybinski@cnbc.uw.edu.pl (D.T.); kwozniak@chem.uw.edu.pl (K.W.); 3 Centre of Molecular and Macromolecular Studies, Polish Academy of Sciences, 90-363 Łódź, Poland

**Keywords:** unsymmetrical dibenzo[*b*,*f*][1,4]diazocines, X-ray diffraction analysis, cytotoxic effect, privilege structure, nitrogen heterocycles

## Abstract

Unsymmetrically *N*-substituted and *N,N’*-disubstituted 5,12-dihydrodibenzo [*b*,*f*][1,4]diazocine-6,11-diones were synthesized in the new protocol. The desired modifications of the dibenzodiazocine scaffold were introduced at the stages of proper selection of building blocks as well as post-cyclization modifications with alkylation or acylation agents, expanding the structural diversity and possible applications of synthesized molecules. The extension of developed method resulted in the synthesis of novel: tricyclic 5,10-dihydrobenzo[*b*]thieno[3,4-*f*][1,4]diazocine-4,11-dione scaffold and fused pentacyclic framework possessing two benzodiazocine rings within its structure. Additionally, the unprecedented rearrangement of 5,12-dihydrodibenzo[*b*,*f*][1,4]diazocine-6,11-diones to 2-(2-aminophenyl)isoindoline-1,3-diones was observed under the basic conditions in the presence of sodium hydride for secondary dilactams. The structures of nine synthesized products have been established by single-crystal X-ray diffraction analysis. Detailed crystallographic analysis of the investigated tri- and pentacyclic systems has shed more light on their structural features. One cell line derived from non-cancerous cells (EUFA30—human fibroblasts) and three tumor cells (U87—human primary glioblastoma, HeLa—cervix adenocarcinoma, BICR18—laryngeal squamous cell carcinoma) were used to determine the cytotoxic effect of the newly synthesized compounds. Although these compounds showed a relatively weak cytotoxic effect, the framework obtained for 5,12-dihydrodibenzo[*b*,*f*][1,4]diazocine-6,11-dione could serve as a convenient privilege structure for the design and development of novel bioactive molecules suitable for drug design, development and optimization programs.

## 1. Introduction

**Figure 1:**
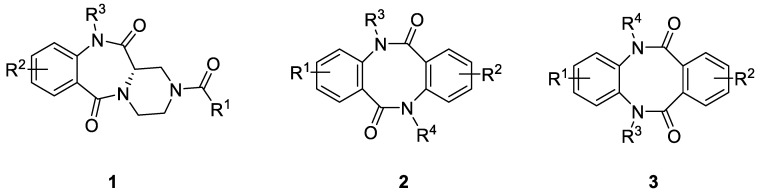
Selective anti-leukemic agents **1** [7] and their structural analogues **2** [9] and **3**—potential scaffolds for the design of bioactive molecules.

Modern medicinal chemistry is mainly based on the design of novel, biologically active compounds containing diverse heterocyclic scaffolds, including privileged structures. Its further development is largely dependent on the availability of new synthetic methods and approaches leading to heterocyclic derivatives [1]. Tricyclic heterocyclic scaffolds, possessing two non-polar phenyl rings separated by a seven-membered heterocycle, and their structural analogues, are challenging synthetic targets for organic chemists [2,3,4,5]. The development of a wide range of novel synthetic methods has led to the synthesis of a broad variety of derivatives with significant biological activity, where the tricyclic antipsychotic drugs focused the most attention [6]. Our own research on the development of novel therapeutic agents, based on tricyclic benzodiazepine derivatives, resulted in the development of unique antileucemic agents **1** (Figure 1), exhibiting a selective cytotoxic effect against MV4-11 and CCRF-7 cell lines [7,8].

Contrary to dibenzoazepines, azolobenzodiazepines and their structural analogues, tricyclic heterocyclic scaffolds, possessing two non-polar phenyl rings separated by an eight-membered heterocycle (Figure 1), such as dibenzodiazocines **2** [9] and **3**, are rarely recognized and considered to be potential privileged structures for design and development of new drugs, despite great structural similarity to their seven-membered heterocyclic analogues. The sparse reports in the literature of application of such molecules include compound **2** (R^1^ = R^2^ = 2,8-di-OMe, R^3^ = R^4^ = Bn) developed as a chemosensitizer [10,11] or compound **3** (R^1^ = R^2^ = R^3^ = R^4^ = H) developed as a ligand for GABA_A_R receptors exhibiting anticonvulsant activity [12].

While searching for novel tricyclic heterocyclic derivatives of medicinal importance [7,8,9], we turned our attention to 5,12-dihydrodibenzo[*b,f*][1,4]diazocine-6,11-dione **3** as a potential privileged scaffold for design and development of novel bioactive compounds. A brief literature survey revealed, that such compounds could be obtained from a condensation reaction of benzene-1,2-diamine and diethyl phthalate [13], benzene-1,2-diamine, and benzocyclobutene-l,2-dione [14,15] or Beckmann rearrangement of appropriate tricyclic dibenzoazepine oximes [16,17]. The parent compound **3** (R^1^ = R^2^ = R^3^ = R^4^ = H) could also be synthesized from benzene-1,2-diamine and 1,2-phenylenebis((1*H*-benzo[*d*][1,2,3]triazol-1-yl)methanone) obtained from phthaloyl dichloride and 1*H*-benzo[*d*][1,2,3]triazole [18], while condensation of 5,6-diaminouracil with phtalic anhydride resulted in the formation of fused pyrimido[4,5-*b*][1,4]diazocine ring [19].

Unfortunately, all the methods described in the literature led to the products **3** having no substituents introduced to the dilactam ring (only secondary lactams R^3^ = R^4^ = H). This limitation significantly decreased the possibilities of modification of this structure, introduction of side chains, and thus the modulation of biological properties of compounds. The main goals of our research were focused on the development of methods of synthesis of previously unknown, asymmetric, *N*-substituted (R^3^ = H, R^4^ ≠ H) and *N,N’*-disubstituted (R^3^ ≠ R^4^ ≠ H) 5,12-dihydrodibenzo [*b*,*f*][1,4]diazocine-6,11-diones **3** and the introduction of some other fused heterocyclic rings in the place of the benzene ring to the tricyclic scaffold. We also focused our efforts on the development of a synthetic method leading to the novel fused pentacyclic scaffold possessing two benzodiazocine rings within its structure, as well as post-cyclisation modifications of obtained products. Additionally, we confirmed the identity of the synthesized scaffolds by single-crystal X-ray diffraction analysis and performed the detailed crystallographic analysis to gain deeper insight in their structural features in the solid state. Finally, the cytotoxic effect of the synthesized compounds was determined using one cell line derived from non-cancerous cells (EUFA30—human fibroblasts) and three tumor cells (U87—human primary glioblastoma; HeLa—cervix adenocarcinoma, BICR18—laryngeal squamous cell carcinoma).

## 2. Results

### 2.1. Chemistry

**Scheme 1:**
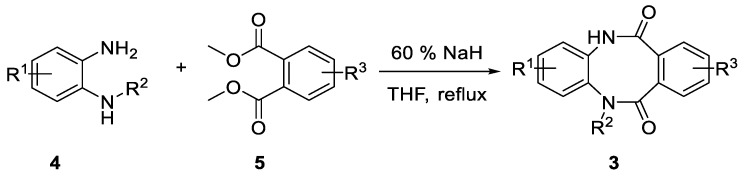
The synthesis of 5,12-dihydrodibenzo[b,f][1,4]diazocine-6,11-dione scaffold.

For the preparation of *N*-substituted (R^3^ = H, R^4^ ≠ H) and *N,N’*-disubstituted (R^3^ ≠ R^4^ ≠ H) 5,12-dihydrodibenzo[*b*,*f*][1,4]diazocine-6,11-diones **3**, we decided to use two types of building blocks: Appropriately modified benzene-1,2-diamines **4** and properly prepared phthalic esters **5** (Scheme 1). To our knowledge, such derivatives containing modified and substituted eight-membered dilactam rings have not yet been described in the literature so far. Our synthetic efforts leading to the fused diazocine-2,5-diones, were started from the synthesis of 1,3,4,6-tetrahydrobenzo[*b*][1,4]diazocine-2,5-dione (**6**), obtained from benzene-1,2-diamine (**4a**) and dimethyl succinate (**7**). In a similar way, 5,12-dihydrodibenzo[*b*,*f*][1,4]diazocine-6,11-dione **(3a)** was synthesized from benzene-1,2-diamine (**4a**) and dimethyl phthalate (**5a**). These compounds **6** [20] and **3a** [13,14,18] were synthesized previously, but for the first time the crystal structures of **6** and **3a** were reported by us. Surprisingly, we did not find any crystallographic structure of compounds possessing neither 1,3,4,6-tetrahydrobenzo[*b*][1,4]diazocine-2,5-dione (**6**) nor 5,12-dihydrodibenzo[*b*,*f*][1,4]diazocine-6,11-dione (**3**) cores in the Cambridge Structural Database [21], which made this group of chemical compounds an interesting and appropriate target for crystallographic studies. To get some more information about the structural features of compounds possessing the 5,12-dihydrodibenzo[*b*,*f*][1,4]diazocine-6,11-dione (**3a**) core, we synthesized tricyclic derivatives substituted with nitro (**3b**, **3d**) or benzoyl group (**3c**). Dimethyl phthalate (**5a**) smoothly reacted with *C*-substituted benzene-1,2-diamines: 4-nitrobenzene-1,2-diamine (**4b**) and 4-benzoyl-1,2-diamine (**4c**), while dimethyl 4-nitrophthalate (**5b**) reacted with benzene-1,2-diamine (**4a**) which resulted in the tricyclic 5,12-dihydrodibenzo[*b*,*f*][1,4]diazocine-6,11-diones (**3b**-**3d**). Despite of the presence of a nitro (**3b**, **5d**) or benzoyl group (**3c**), which, in our opinion should facilitate the crystallization process, we were unable to obtain crystals appropriate for crystallographic analysis and consequently, we focused our attention on the modification of the eight-membered diazocine-6,11-dione dilactam ring. Such a modification could be desirable in the design and synthesis of novel bioactive compounds and could be responsible for the modulation of biological effect and properties. This chosen direction of research was supported by our previous results [7], where the alkylation of a seven-membered dilactam ring in tricyclic heterocyclic derivative with benzyl bromide, potentiated the selectivity and cytotoxic effect of the investigated compounds toward leukemic and colon cancer cell lines.

For the preparation of previously unknown *N*-substituted (R^3^ = H, R^4^ ≠ H) 5,12-dihydrodibenzo[b,f][1,4]diazocine-6,11-diones **3**, we used commercially available *N*^1^-methylbenzene-1,2-diamine (**4d**) as well as 4,5-dichloro*-N*^1^-methylbenzene-1,2-diamine (**4e**) and *N*^1^-benzylbenzene-1,2-diamine (**4f**) obtained in facile two-step syntheses. *N*^1^-methylbenzene-1,2-diamine (**4d**) and 4,5-dichloro*-N*^1^-methylbenzene-1,2-diamine (**4e**) reacted smoothly with dimethyl phthalate (**5a**), dimethyl 4,5-dichlorophthalate (**5c**) or dimethyl 3,4,5,6-tetrachlorophthalate (**5d**), in the presence of sodium hydride, giving requested products **3e–h** with high yields (Table 1). We also succeeded in the condensation of *N*^1^-methylbenzene-1,2-diamine (**4d**) with heterocyclic analogue of phtalic ester, dimethyl thiophene-3,4-dicarboxylate (**5e**), which resulted in the formation of a novel heterocyclic scaffold: 5-methyl-5,10-dihydrobenzo[*b*]thieno[3,4-*f*][1,4]diazocine-4,11-dione (**3i**). In a similar manner, *N*^1^-benzylbenzene-1,2-diamine (**4f**) reacted with dimethyl phthalate (**5a**), giving 5-benzyl-5,12-dihydrodibenzo[*b*,*f*][1,4]diazocine-6,11-dione (**3j**) as a sole product.

**Scheme 2:**
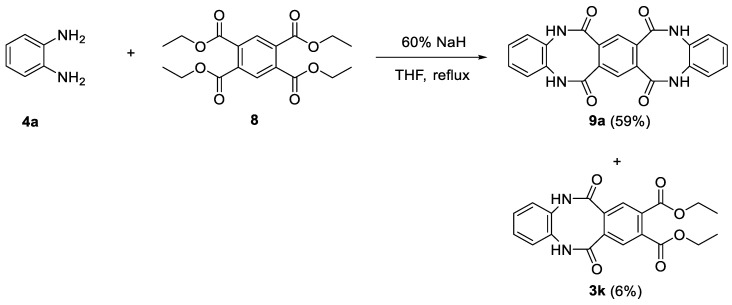
The synthesis of pentacyclic scaffold.

We also investigated the reaction between benzene-1,2-diamine (**4a**) and tetraethyl benzene-1,2,4,5-tetracarboxylate (**8**) in a 2:1 ratio and observed the formation of a novel pentacyclic product **9a**, possessing a unique scaffold consisting of two diazocine-6,11-dione rings separated by a benzene ring (Scheme 2). Product **9a** precipitated out of the reaction mixture after the reaction took place and was separated by filtration under reduced pressure. In the obtained filtrate, we also identified small amounts of the intermediate tricyclic diethyl 6,11-dioxo-5,6,11,12-tetrahydrodibenzo[*b*,*f*][1,4]diazocine-8,9-dicarboxylate (**3k**), which was purified and isolated by column chromatography.

**Scheme 3:**
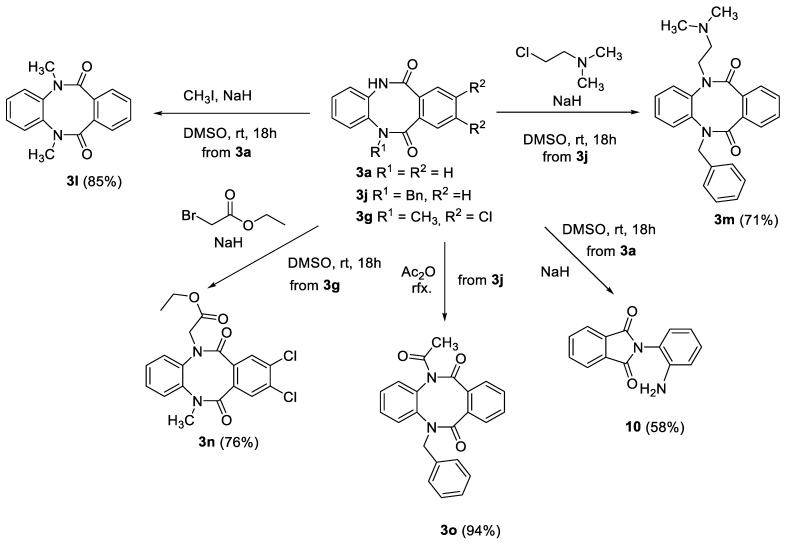
Chemical modifications of 5,12-dihydrodibenzo[*b*,*f*][1,4]diazocine-6,11-dione scaffold.

The treatment of 5,12-dihydrodibenzo[*b*,*f*][1,4]diazocine-6,11-dione (**3a**) with an excess of methyl iodide, in the presence of sodium hydride, resulted in the formation of 5,12-dimethyl-5,12-dihydrodibenzo[*b*,*f*][1,4]diazocine-6,11-dione (**3l**) (Scheme 3). Surprisingly, in the absence of an alkylating reagent, we observed a novel, unprecedented rearrangement of 5,12-dihydrodibenzo[*b*,*f*][1,4]diazocine-6,11-dione (**3a**) to 2-(2-aminophenyl)isoindoline-1,3-dione (**10**), which structure was confirmed by X-ray crystallography [22]. The structure of compound **10** resembles the structure of Thalidomide, a drug used in anticancer therapy, and the described method of synthesis can be used to obtain a variety of Thalidomide analogues [23,24]. Although, rearrangement of **3a** under acidic conditions, in the presence of phosphoryl chloride [25] or phosphorus pentachloride [13], leading to 2-phenyl-1*H*-benzo[*d*]imidazole derivatives, has already been described in the literature, the base-promoted contraction of eight-membered diazocine-6,11-dione ring in **3a** to the five-membered pyrrolidine-2,5-dione ring in **10** was observed and described by us for the first time.

For the synthesis of *N,N’*-disubstituted (R^3^ ≠ R^4^ ≠ H) 5,12-dihydrodibenzo[*b*,*f*][1,4]diazocine-6,11-diones **3**, we used 8,9-dichloro-5-methyl-5,12-dihydrodibenzo[*b*,*f*][1,4]diazocine-6,11-dione (**3g**) and 5-benzyl-5,12-dihydrodibenzo[*b*,*f*][1,4]diazocine-6,11-dione (**3j**) (Scheme 3).

**Scheme 4:**
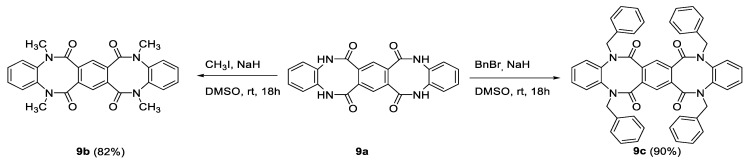
Alkylation of pentacyclic scaffold.

Compound **3j** was treated with 2-chloro-*N*,*N*-dimethylethan-1-amine, in the presence of sodium hydride, which resulted in the formation of 5-benzyl-12-(2-(dimethylamino)ethyl)-5,12-dihydrodibenzo[*b*,*f*][1,4]diazocine-6,11-dione (**3m**). In a similar way, the treatment of **3g** with ethyl bromoacetate led to the formation of ethyl 2-(8,9-dichloro-12-methyl-6,11-dioxo-11,12-dihydrodibenzo[*b*,*f*][1,4]diazocin-5(6*H*)-yl)acetate (**3n**) (Scheme 3). The heating of **3j** in acetic anhydride at reflux temperature gave the *N*-acyl derivative, 5-acetyl-12-benzyl-5,12-dihydrodibenzo[*b*,*f*][1,4]diazocine-6,11-dione (**3o**). We also attempted to alkylate the pentacyclic derivative **9a** by treating it with methyl iodide or benzyl bromide in the presence of sodium hydride (Scheme 4), which resulted in the tetramethyl derivative **9b** and tetrabenzyl derivative **9c**. The ^1^H NMR spectrum registered for **9a** indicates the presence of two conformational isomers in the ratio of 1:0.25, which can be deduced from two sets of signals originating from NH and Ar-H protons. These conformers are the result of dihydrodiazocine-6,11-dione rings being able to adopt two possible orientations, leading to a ‘U’-shaped or a ‘Z’-shaped molecule, as seen in the crystal structures of compounds **9c** and **9a**, respectively. Similarly, we noticed the presence of two sets of signals in the ^1^H NMR spectrum of **9b** (here in the ratio of 1:0.6), suggesting that in this case conformational equilibrium of two possible orientations of the mentioned ring is also observed. The ^1^H NMR spectra in the temperature range of 275–325 K (2–52 °C) registered for **9b** dissolved in trifluoroacetic acid-*d* shows that the temperature drift of ^1^H signals originating from both conformers is different, indicating somewhat different dynamics in both forms (for temperature ^1^H NMR spectra showing this temperature drift see Supporting Information, Figure S25b). Interestingly, in the ^1^H NMR spectrum of **9c** only one set of ^1^H signals is visible. This is probably the result of the presence of four bulky substituents at the nitrogen atoms, which effectively prevents **9c** from adopting a less energetically favorable conformation.

### 2.2. Crystallographic Analysis

Attempts to obtain single-crystals suitable for X-ray diffraction measurements were undertaken for all synthesized compounds. However, they were only successful in nine cases (**3a**, **3g–3j**, **6**, **9a**, **9c** and **10**). Single crystals appropriate for this purpose were grown by slow evaporation from: **3a**—DMSO:DMF (1:1), **3g**—*n*-butyl acetate, **3h**—ethanol, **3i**—THF:water (9:1), **3j**—ethanol, **6**—DMF, **9a**—DMSO:NMP (1:1), **9c**—dimethylacetamide (DMA), **10**—ethanol.

**Figure 2:**
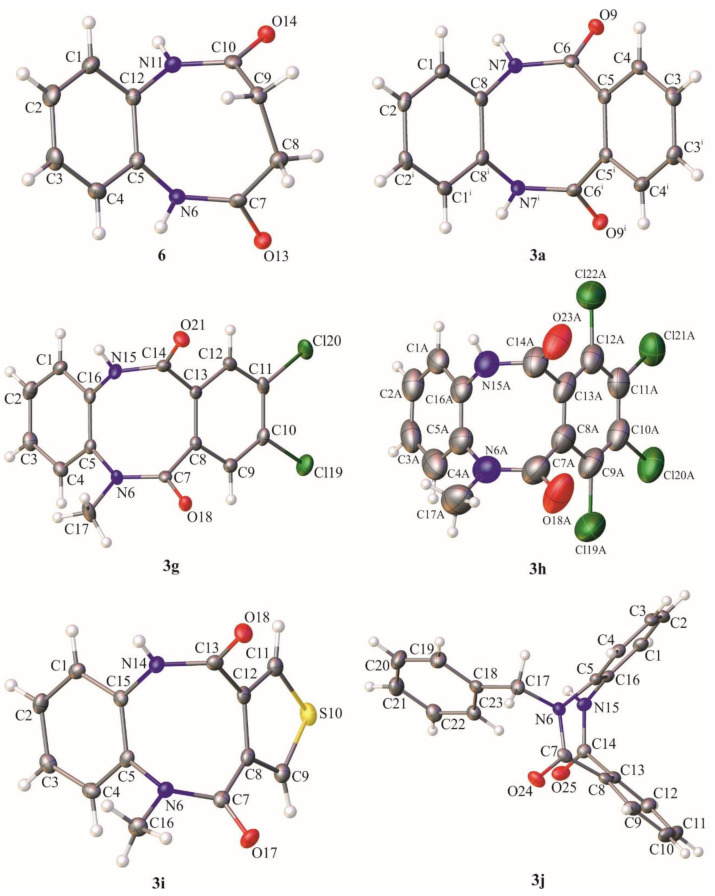
Molecular structure of **3a**, **3g**-**j** and **6** with crystallographic atom numbering. Displacement ellipsoids are drawn at the 50% probability level. The H-atoms are shown as small spheres of arbitrary radius. Solvent molecule in case of **3i** and the second crystallographically independent molecule of **3h** have been omitted for clarity. Symmetry code: (i) *x*, −*y* + 1, *z*.

The investigated compounds crystallized in the monoclinic *P*2_1_/*n* (**3h**, **3i**, **9a** and **10**), *P*2_1_/*c* (**3g**, **9c**), *C*2/*c* (**3j**) and *C*2/*m* (**3a**) or triclinic *P*-1 (**6**) space groups. The asymmetric unit of the crystal lattice contain half (**3a** and **9a**), one (**3g**, **3i**, **3j**, **6**, **9c** and **10**) or two molecules of given compound (**3h**) (Figure 2 and Figures S1−S5 (ESI)). Three products crystallized in the form of solvates (**3i**, **9a** and **9c**) (Figures S2−S4 (ESI)). The refinement parameters and details of the crystallographic data are presented in Table S1 (ESI). The values of bond lengths, valence and torsion angles are presented in Tables S2−S28 (ESI).

**Figure 3:**
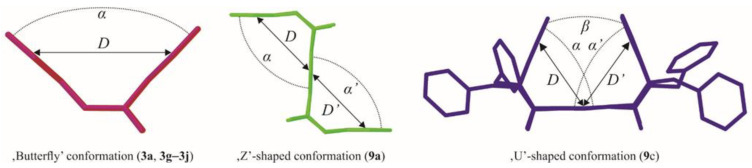
The graphical representation of the conformation types of molecules of investigated tri- and pentacyclic compounds with geometrical parameters used for their description.

The conformation of the dibenzodiazocine skeleton in the case of all investigated tricyclic derivatives (**3a**, **3g−3j**) resembles a butterfly (Figure 3). Differences in the molecular geometry of the above moiety can be emphasized using two parameters: *α*, which is the angle between mean-planes defined by the non-hydrogen atoms of the outer aromatic rings and *D*—a distance between geometrical centers of gravity of these rings (Figure 3). The value of *α* for **3a**, **3g−j** ranges from 72.3(3) to 87.74(4)° (Table 2); however, in most cases (**3a**, **3g**, **3i** and **3j**) the values are very close to each other. Detailed analysis indicates that the angle is less obtuse in cases, where outer aromatic rings within the molecule are differently substituted (**3g** and **3h**) or composed (**3j**). This is particularly evident in **3h** where one of the phenyl moieties is bearing four chlorine atoms. In this case the mean-planes of the phenyl rings are inclined to each other just by 72.3(3)/79.9(3)° (molecule A/molecule B). This can probably be explained by the specific mutual orientation of the adjacent molecules of the compound in the crystal network. The molecules of **3h** are held together by the N−H···O hydrogen bond (Figure S1 (ESI)) in a way that their neighboring phenyl rings are close enough to force compression of the dibenzodiazocine moieties. The respective orientation of the above aromatic rings manifests also in the presence of the π–π contact between them (d(Cg*I*···*Cg*J) = 3.644(5) Å, Figure S1 (ESI)). Interestingly this type of intermolecular interaction was not identified in the crystals of the remaining tricyclic systems. As for the distance between the geometric centers of the outer aromatic rings within the molecules of investigated tricyclic compounds (*D*)—here the values are more scattered (4.231(5)−4.695(2) Å, Table 2). The highest value was observed in case of the 5,12-dihydrodibenzo[*b*,*f*][1,4]diazocine-6,11-dione (**3a**), while the lowest was for the **3h**. Like in the case of *α,* also here the smaller parameter values accompany compounds bearing differently substituted or composed outer phenyl rings within the molecule (**3g**, **3h** and **3j**).

**Figure 4:**
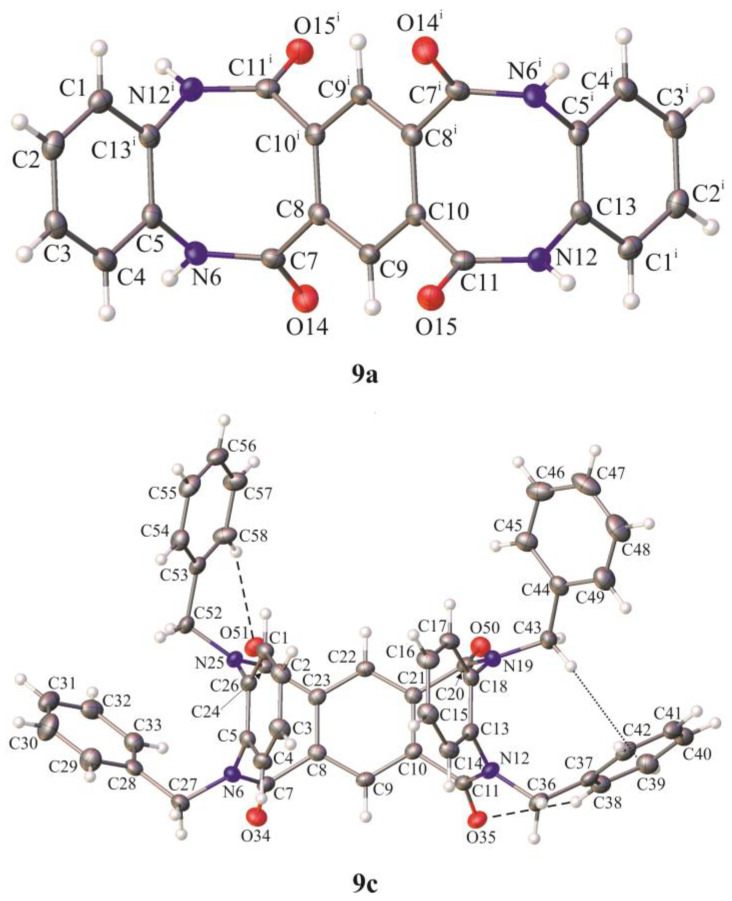
Molecular structure of **9a** and **9c** with crystallographic atom numbering. Displacement ellipsoids are drawn at the 50% probability level. The H-atoms are shown as small spheres of arbitrary radius. The solvent molecules have been omitted for clarity. The intramolecular C−H···O hydrogen bonds are represented by dashed lines, while the C−H···π contact by dotted lines. Symmetry code: (i) −*x* + 2, −*y* + 1, −*z* + 1.

The structure of both pentacyclic products composed of two fused dibenzodiazocine units (**9a** and **9c**) is more complex. The molecular skeleton of **9a** adopts the ‘Z’-shaped conformation (Figure 3). The middle phenyl ring is situated directly at the inversion center, what makes both sides of the molecule describable by the same values of geometric parameters. The values of *α* in **9a** are slightly larger comparing to the previously analyzed tricyclic systems, while the value of *D* is comparable to **3j** (Table 2). In turn, the conformation of **9c**, which is *N*-substituted by four bulky benzyl groups can be described as the “U”-shaped (Figure 3). In the case of this derivative, the aromatic rings within the respective dibenzodiazocine moieties are inclined to each other at more acute angles (64.78(6)° and 69.52(6)°), and the distance between their geometric centers of gravity is the shortest among the all investigated compounds (4.057(2) and 4.170(2) Å, Table 2). The outer phenyl rings of the pentacyclic moiety are inclined to themselves at an angle of 45.74(7)°. The mutual orientation of the benzyl groups attached to the N12 and N25 nitrogen atoms relative to the main molecular unit results in the appearance of weak intramolecular C−H···O hydrogen bonds involving the nearest carbonyl O-atoms (Figure 4 and Figure S4, Table S30 (ESI)). In turn the substituent attached to the N19 atoms participating in the formation of weak intramolecular C−H···π contact (Figure 4 and Figure S4, Table S31 (ESI)). Besides the aforementioned differences in structural features, **9a** and **9c** differ significantly in a way in which their molecules are organized in the crystals. Both systems crystallized with additional solvent molecules incorporated in their crystal networks. What is interesting—in the crystal lattice of **9a** two different solvents (dimethyl sulfoxide and 1-methyl-pyrrolidin-2-one) are present. In the case of **9c,** the *N*,*N*-dimethylacetamide was found. In both of the investigated crystals, molecules are involved in a network of intermolecular hydrogen bonds (Tables S29 and S30). In the crystal of **9c** also the intermolecular C−H···π contacts were identified (Table S31 (ESI)). A close look at figures presenting the supramolecular architecture of above solvates reveals that in the case of **9a,** separate layers formed by molecules of the compound and solvents can be distinguished (Figure S6 (ESI)), whereas in **9c,** the molecules of the solvent are “interlocked” in pockets of the framework formed by the molecules of the compound (Figure S7 (ESI)).

### 2.3. Cytotoxic Effect of 5,12-dihydrodibenzo[b,f][1,4]diazocine-6,11-diones **3a-o**

**Figure 5:**
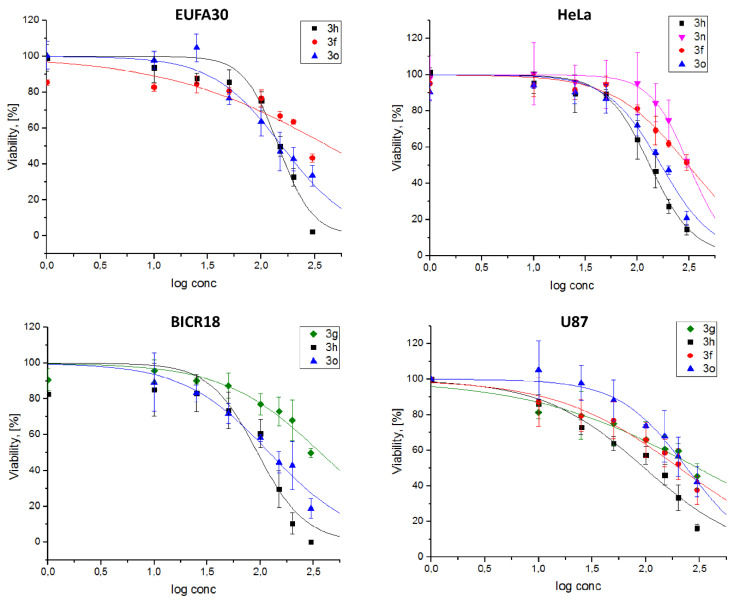
The cytotoxic effect of indicated 5,12-dihydrodibenzo[*b*,*f*][1,4]diazocine-6,11-diones on normal EUFA30 and cancerous HeLa, BICR18, and U87 cells after 24 h based on AlamarBlue assay. Non-linear fitting with logistic dose response model was employed.

We evaluated the cytotoxic efficacy of 17 synthesized 5,12-dihydrodibenzo[*b*,*f*][1,4]diazocine-6,11-diones **3a-o** on one normal (EUFA30) and three cancerous (HeLa, BICR18, U87) cell lines at concentrations between 1 and 300 µM (Figure 5). Among the tested compounds, five: **3f**, **3g**, **3h**, **3n**, and **3o,** showed cytotoxic effect at the same time maintaining the selectivity of action –– their IC_50_ ranged from several dozen (lowest: 93.7 µM) to several hundred (highest: 322.8 µM). Nevertheless, the IC_50_ for the normal cell line was higher than for the cancerous one. For the majority of the remaining compounds, IC_50_ values were above the highest concentration used (Table 3).

**Figure 6:**
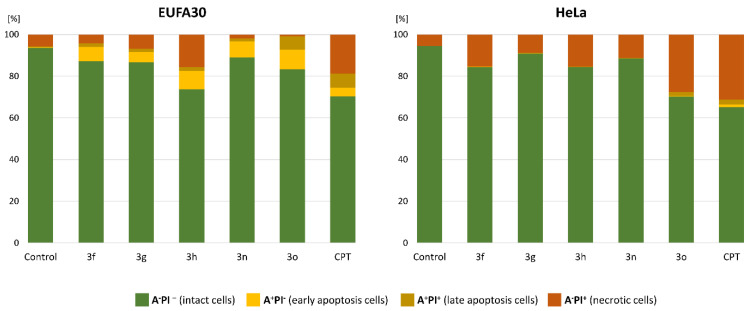
Flow cytometry analysis of EUFA30 and HeLa cells stained with Annexin-FITC (A), and propidium iodide (PI). Cells were treated with 200 µM of indicated compounds for 24 h. Camptothecin (10 μM) was used as experimental control.

To measure the induction of apoptosis, cells were treated with 200 µM of **3f**, **3g**, **3h**, **3n**, and **3o** compound, for 24 h (Figure 6). The experiment was performed on one normal (EUFA30) and one cancer (HeLa) cell line. After 24 h of EUFA30 treatment we observed significant increase in dead cell fractions: Early apoptosis, late apoptosis, and necrosis for almost all tested compounds: 6.9%, 1.6% and 4.3% for **3f**, 5.0%, 1.5% and 6.8% for **3g**, 9.0%, 1.8% and 15.6% for **3h**, 7.8%, 1.4% and 1.9% for **3n**, 9.5%, 6.4% and 0.8% for **3o,** respectively, comparing to 0.3%, 0.4% and 5.7% in the non-treated control. In the positive control (camptothecin—CPT) the proportion of dead cell fractions was 4.1%, 6.8% and 18.7%, respectively. The best active compound was **3h** and the least **3n**.

The HeLa cells were sensitive to all tested compounds with a significant increase in necrotic phase only. After 24 h, the number of necrotic cells for **3f**, **3g**, **3h**, **3n**, and **3o** were 15.3, 8.8, 15.3, 11.3, and 27.7%, respectively, in comparison to 5.4% in the non-treated control. The best cytotoxic agent appears to be **3o**, the compound that killed cells similarly to CPT, a positive control with 31.2% of necrotic cells.

In HeLa cells these compounds cause mainly necrosis in contrast to EUFA30 cells, where in the most cases apoptosis, early and late, appeared in the dead cell fractions.

Although most compounds showed a weak cytotoxic effect, some relationships between structure and activity could be observed. Compounds substituted with chlorine atoms in the benzene ring **3f, 3g, 3h,** and **3n** proved to be the most cytotoxic, and this effect was the strongest for compound **3h**, substituted with four chlorine atoms (IC_50_ = 93.7 ± 5.2 μM for BIRC18, IC_50_ = 97.1 ± 13.2 μM for U87). In the group of compounds substituted with chlorine atoms, the alkylation of **3g** with ethyl bromoacetate resulted in derivative **3n**, did not significantly change the cytotoxicity for the cell lines tested. The acylation of **3j** resulting in the **3o** derivative significantly enhanced the cytotoxic effect. For compound **3j**, the IC_50_ values was above 300 µM for all tested cell lines, while for **3o** these values ranged from 121.0 ± 11.4 µM (for BIRC18) to 240.2 ± 6.1 µM (for U87).

## 3. Materials and Methods

### 3.1. Chemistry

#### 3.1.1. General Information

Commercially available chemicals were of reagent grade and used as received. The reactions were monitored by thin layer chromatography (TLC), using silica gel plates (Kieselgel 60F_254_, E. Merck, Darmstadt, Germany). Column chromatography was performed on silica gel 60 M (0.040–0.063 mm, E. Merck, Darmstadt, Germany). Melting points are uncorrected and were measured on a Büchi (New Castle, DE, USA) Melting Point B-540 apparatus. All ^1^H and ^13^C NMR spectra were registered with a Bruker Avance III (Billerica, MA, USA) spectrometer operating at 500.13 and 125.77 MHz for ^1^H and ^13^C, respectively, and equipped with a 5 mm probe head with Z-gradient coils. The experiments were performed using pulse programs from the standard Bruker library for samples dissolved in DMSO-*d_6_*, methanol-*d_4_* or trifluoroacetic acid-*d*, depending on the sample. In each case spectra were calibrated at residual solvent resonances. For the temperature ^1^H NMR spectra a BCU unit was used for temperature control and stabilization. After reaching the required temperature measured samples were equilibrated at this temperature for at least 10 min prior to measurement. Solvent signals in NMR spectra were determined on the basis of literature data [26]. High resolution mass spectra were performed by the Laboratory of Mass Spectrometry, Institute of Biochemistry and Biophysics PAS, on a LTQ Orbitrap Velos instrument, Thermo Scientific (Waltham, MA, USA). IR spectra were recorded with the Jasco 6200 (Easton, MD, USA) FT/IR spectrometer in the Laboratory of Optical Spectroscopy, Institute of Organic Chemistry PAS (Warsaw, Poland).

#### 3.1.2. General Procedure for Synthesis 5,12-dihydrodibenzo[*b*,*f*][1,4]diazocine-6,11-diones (**3a**-**j**)

To a solution of diamine **4a–f** (1 equiv.) and diester **5a–e** (1 equiv.) in anhydrous THF (5 mL/mmol), 60% sodium hydride in mineral oil (2 equiv.) was added and the resulting mixture was refluxed for 18 h. The reaction mixture was poured into water, acidified with concentrated HCl to pH 2 and extracted with ethyl acetate. The organic phase was washed with brine and dried over anhydrous magnesium sulfate. The drying agent was filtered off and the resulting filtrate was evaporated under reduced pressure. The crude product was purified either by crystallization or by column chromatography.

*5,12-dihydrodibenzo[b,f][1,4]diazocine-6,11-dione* (**3a**), crystallized from DMF, yield 78%, white crystals, m.p. 286.0–287.0 °C. ^1^H NMR (500 MHz, DMSO-*d*_6_) δ 10.18 (s, 2H, 2 × NH), 7.65–6.85 (m, 8H, H_Ar_); ^13^C NMR (125 MHz, DMSO-*d*_6_) δ 170.4, 134.8, 132.3, 130.2, 127.5, 127.0, 126.6; IR (KBr): cm^−1^ 3178, 3049, 2938, 2901, 2874, 1936, 1825, 1651, 1593, 1573, 1502, 1461, 1403, 1304, 1265, 1239, 1140, 1115, 1043; HRMS (ESI): *m/z* [M + H]^+^ calcd for C_14_H_11_N_2_O_2_: 239.08150, found: 239.08174.

*2-nitro-5,12-dihydrodibenzo[b,f][1,4]diazocine-6,11-dione* (**3b**), crystallized from ethyl acetate, yield 53%, yellow crystals, m.p. 230.0 °C (decomposition), R.f. = 0.38 (hexane:ethyl acetate 2:8 *v*/*v*). ^1^H NMR (500 MHz, DMSO-*d*_6_) δ 10.66 (s, 1H, NH), 10.44 (s, 1H, NH), 8.06 (dd, 1H, J = 2.0 Hz, J = 8.0 Hz, H_Ar_), 7.99 (d, 1H, J = 2.0 Hz, H_Ar_), 7.51–7.30 (m, 5H, H_Ar_); ^13^C NMR (125 MHz, DMSO-*d*_6_) δ 170.2, 170.0, 145.5, 140.9, 135.2, 131.6, 131.4, 130.72, 130.66, 127.6, 126.9, 126.8, 122.6, 122.1; IR (KBr): cm^−1^ 3169, 3077, 3035, 2924, 2862, 1678, 1654, 1593, 1573, 1533, 1501, 1393, 1349, 1238, 1141, 1064; HRMS (ESI): *m/z* [M + H]^+^ calcd for C_14_H_10_N_3_O_4_: 284.06658, found: 284.06699.

*2-benzoyl-5,12-dihydrodibenzo[b,f]*[1,4]*diazocine-6,11-dione* (**3c**), crystallized from ethyl acetate, yield 55%, beige crystals, m.p. 156.0–157.0 °C, R.f. = 0.24 (hexane:ethyl acetate 2:8 *v*/*v*). ^1^H NMR (500 MHz, DMSO-*d*_6_) δ 10.52 (s, 1H, NH), 10.32 (s, 1H, NH), 7.71–7.63 (m, 3H, H_Ar_), 7.59–7.50 (m, 4H, H_Ar_), 7.49–7.43 (m, 2H, H_Ar_), 7.38–7.30 (m, 3H, H_Ar_); ^13^C NMR (125 MHz, DMSO-*d*_6_) δ 194.0, 170.3, 170.2, 138.6, 136.5, 135.5, 134.7, 132.9, 131.9, 131.8, 130.51,130.49, 129.5 (2 × C), 128.9, 128.6 (2 × C), 128.2, 126.9, 126.80, 120.77; IR (KBr): cm^−1^ 3171, 3060, 2931, 2873, 1734, 1685, 1659, 1601, 1574, 1492, 1446, 1392, 1314, 1278, 1263, 1239, 1179, 1141, 1125, 1078, 1043; HRMS (ESI): *m/z* [M + H]^+^ calcd for C_21_H_15_N_2_O_3_: 343.10772, found: 343.10731.

*8-nitro-5,12-dihydrodibenzo[b,f][1,4]diazocine-6,11-dione* (**3d**), crystallized from ethyl acetate, yield 50%, yellow crystals, m.p. 243.0–244.0 °C, R.f. = 0.40 (hexane:ethyl acetate 2:8 *v*/*v*). ^1^H NMR (500 MHz, DMSO-*d*_6_) δ 10.521 (s, 1H, NH), 10.492 (s, 1H, NH), 8.22 (dd, 1H, J = 2.5 Hz, J = 8.5 Hz, H_Ar_), 8.11 (d, 1H, J = 2.5 Hz, H_Ar_), 7.63 (d, 1H, J = 8.5 Hz, H_Ar_), 7.26–7.15 (m, 4H, H_Ar_); ^13^C NMR (125 MHz, DMSO-*d*_6_) δ 168.5, 168.2, 148.3, 137.7, 134.4, 134.3, 133.4, 128.7, 128.01, 127.98, 127.4, 127.2, 125.2, 121.9; IR (KBr): cm^−1^ 3245, 3078, 1674, 1645, 1612, 1582, 1527, 1503, 1487, 1446, 1396, 1346, 1301, 1236, 1149, 1065; HRMS (ESI): *m/z* [M + H]^+^ calcd for C_14_H_10_N_3_O_4_: 284.06658, found: 286.06693.

*5-methyl-5,12-dihydrodibenzo[b,f][1,4]diazocine-6,11-dione* (**3e**) crystallized from ethyl acetate, yield 61%, white crystals, m.p. 206.0–207.0 °C, R.f. = 0.36 (hexane:ethyl acetate 2:8 *v*/*v*). ^1^H NMR (500 MHz, DMSO-*d*_6_) δ 10.21 (s, 1H, NH), 7.47–7.41 (m, 1H, H_Ar_), 7.40–7.34 (m, 2H, H_Ar_), 7.31–7.19 (m, 4H, H_Ar_), 7.19–7.12 (m, 1H, H_Ar_), 3.30 (s, 3H, Me); ^13^C NMR (125 MHz, DMSO-*d*_6_) δ 170.3, 168.6, 140.4, 135.1, 133.3, 131.7, 130.2, 130.1, 128.3, 128.2, 127.2, 127.1, 126.4, 126.2, 36.6; IR (KBr): cm^−1^ 3213, 3063, 2927, 2883, 1965, 1935, 1828, 1675, 1626, 1594, 1571, 1502, 1488, 1420, 1392, 1346, 1309, 1256, 1236, 1191, 1162, 1140, 1080, 1036, 1007; HRMS (ESI): *m/z* [M + H]^+^ calcd for C_15_H_13_N_2_O_2_: 253.09715, found: 253.09709.

*2,3-dichloro-5-methyl-5,12-dihydrodibenzo[b,f][1,4]diazocine-6,11-dione* (**3f**) crystallized from THF, yield 55%, white crystals, m.p. 198.0–199.0 °C, R.f. = 0.57 (hexane:ethyl acetate 2:8 *v*/*v*). ^1^H NMR (500 MHz, DMSO-*d*_6_) δ 10.35 (s, 1H, NH), 7.91 (s, 1H, H_Ar_); 7.49 (s, 1H, H_Ar_); 7.47–7.40 (m, 2H, H_Ar_), 7.32–7.25 (m, 2H, H_Ar_), 3.30 (s, 3H, Me); ^13^C NMR (125 MHz, DMSO-*d*_6_) δ 170.0, 168.3, 140.3, 135.4, 132.7, 131.1, 130.6, 130.4, 130.3, 130.0, 129.1, 128.3, 126.6, 126.4, 36.6; IR (KBr): cm^−1^ 3177, 3095, 3039, 2894, 1680, 1633, 1592, 1567, 1485, 1427, 1398, 1357, 1305, 1262, 1226, 1138, 1110, 1063, 1019; HRMS (ESI): *m/z* [M + H]^+^ calcd for C_15_H_11_N_2_O_2_Cl_2_: 321.01921, 323.01626, found: 321.01957, 323.01658.

*8,9-dichloro-5-methyl-5,12-dihydrodibenzo[b,f][1,4]diazocine-6,11-dione* (**3g**) crystallized from ethyl acetate, yield 67%, white crystals, m.p. 207.0–208.0 °C, R.f. = 0.53 (hexane:ethyl acetate 2:8 *v*/*v*). ^1^H NMR (500 MHz, DMSO-*d*_6_) δ 10.43 (s, 1H, NH), 7.59 (s, 2H, H_Ar_), 7.46 (d, 1H, J = 7.0 Hz, H_Ar_), 7.35–7.24 (m, 2H, H_Ar_), 7.19 (d, 1H, J = 7.0 Hz, H_Ar_), 3.30 (s, 3H, Me); ^13^C NMR (125 MHz, DMSO-*d*_6_) δ 167.8, 166.2, 139.9, 134.6, 133.5, 133.13, 133.05, 132.1, 128.8, 128.63, 128.59, 128.4, 127.43, 127.36, 36.7; IR (KBr): cm^−1^ 3213, 3087, 2931, 1671, 1640, 1593, 1546, 1501, 1424, 1396, 1375, 1316, 1256, 1234, 1130, 1092, 1020; HRMS (ESI): *m/z* [M + H]^+^ calcd for C_15_H_11_N_2_O_2_Cl_2_: 321.01921, 323.01626, found: 321.01957, 323.01664.

*7,8,9,10-tetrachloro-5-methyl-5,12-dihydrodibenzo[b,f][1,4]diazocine-6,11-dione* (**3h**) column chromatography methanol:chloroform 1:9 *v*/*v*, yield 15%, white crystals, m.p. 233.0 °C (decomposition), R.f. = 0.67 (hexane:ethyl acetate 2:8 *v*/*v*). ^1^H NMR (500 MHz, DMSO-*d*_6_) δ 10.78 (s, 1H, NH), 7.50 (d, 1H, J = 7.0 Hz, H_Ar_), 7.37–7.26 (m, 2H, H_Ar_), 7.23 (d, 1H, J = 7.0 Hz, H_Ar_), 3.31 (s, 3H, Me); ^13^C NMR (125 MHz, DMSO-*d*_6_) δ 164.5, 163.3, 140.1, 134.7, 134.2, 134.1, 133.1, 131.7, 130.0, 129.9, 128.4, 128.2, 127.9, 127.8, 36.7; IR (KBr): cm^−1^ 3347, 3209, 3107, 2936, 2889, 2857, 1678, 1652, 1596, 1537, 1497, 1433, 1401, 1353, 1299, 1264, 1232, 1149, 1098, 1038; HRMS (ESI): *m/z* [M + H]^+^ calcd for C_15_H_9_N_2_O_2_Cl_4_: 388.94126, 390.93831, 392.93536, found: 388.94181, 390.93890, 392.93563.

*5-methyl-5,10-dihydrobenzo[b]thieno[3,4-f][1,4]diazocine-4,11-dione* (**3i**) colum chromatography hexane:ethyl acetate 2:8 *v*/*v*, yield 47%, white crystals, m.p. 241.0–242.0 °C, R.f. = 0.32 (hexane:ethyl acetate 2:8 *v*/*v*). ^1^H NMR (500 MHz, DMSO-*d*_6_) δ 10.78 (s, 1H, NH), 7.76–7.40 (m, 2H, H_Ar_), 7.47–7.40 (m, 1H, H_Ar_), 7.32–7.23 (m, 2H, H_Ar_), 7.20–7.14 (m, 1H, H_Ar_), 3.27 (s, 3H, Me); ^13^C NMR (125 MHz, DMSO-*d*_6_) δ 166.9, 165.2, 140.0, 135.1, 134.7, 134.2, 128.4, 128.3, 128.2, 127.8, 127.52, 127.48; IR (KBr): cm^−1^ 3176, 3108, 3055, 2904, 1735, 1645, 1587, 1503, 1469, 1366, 1301, 1283, 1243, 1160, 1105, 1044; HRMS (ESI): *m/z* [M + H]^+^ calcd for C_13_H_11_N_2_O_2_S: 259.05357, found: 259.05396.

*5-benzyl-5,12-dihydrodibenzo[b,f][1,4]diazocine-6,11-dione* (**3j**) crystallized from ethyl acetate, yield 74%, white crystals, m.p. 205.0–206.0 °C, R.f. = 0.62 (hexane:ethyl acetate 2:8 *v*/*v*). ^1^H NMR (500 MHz, DMSO-*d*_6_) δ 10.09 (s, 1H, NH), 7.44–7.37 (m, 2H, H_Ar_), 7.36–7.21 (m, 8H, H_Ar_), 7.19–7.7.11 (m, 2H, H_Ar_), 7.09–7.02 (m, 1H, H_Ar_), 5.30 (d, 1H, J = 15.0 Hz, CH_2_), 4.81 (d, 1H, J = 15.0 Hz, CH_2_); ^13^C NMR (125 MHz, DMSO-*d*_6_) δ 170.2, 168.9, 138.9, 136.7, 135.7, 133.2, 131.6, 130.3, 130.1, 128.4 (2 × C), 128.3, 127.8 (3 × C), 127.3, 127.2, 127.0, 126.5, 126.2, 52.4; IR (KBr): cm^−1^ 3160, 3038, 2900, 2869, 1655, 1596, 1572, 1501, 1487, 1430, 1401, 1384, 1360, 1321, 1270, 1224, 1164, 1141, 1121, 1089, 1046, 1025; HRMS (ESI): *m/z* [M + H]^+^ calcd for C_21_H_17_N_2_O_2_: 329.12845, found: 329.12877.

#### 3.1.3. 5,12-dimethyl-5,12-dihydrodibenzo[*b*,*f*][1,4]diazocine-6,11-dione (**3l**)

To a solution of 5,12-dihydrodibenzo[*b*,*f*][1,4]diazocine-6,11-dione (**3a**) (2.0 g, 8.4 mmol, 1.0 equiv.) in anhydrous DMSO (20 mL), 60% sodium hydride in mineral oil (0.74 g, 18.4 mmol, 2.2 equiv.) was added and resulting mixture was stirred for 30 min. After the addition of methyl iodide (1.2 mL, 18.4 mmol 2.2 equiv.), the reaction mixture was stirred for 18 h at room temperature, then was poured into water and the crude product was extracted with ethyl acetate (3 × 50 mL). The organic phase was washed with brine (1 × 50 mL) and dried over anhydrous magnesium sulfate. The crude product was crystallized from ethyl acetate giving **3l** as colorless crystals (1.9 g, yield 85%). M.p. 265.0–266.0 °C, R.f. = 0.42 (hexane:ethyl acetate 2:8 *v*/*v*). ^1^H NMR (500 MHz, DMSO-*d*_6_) δ 7.48–7.41 (m, 1H, H_Ar_), 7.38–7.32 (m, 1H, H_Ar_), 7.31–7.25 (m, 1H, H_Ar_), 7.24–7.18 (m, 1H, H_Ar_), 3.35 (s, 6H, 2 × Me, overlapped with H_2_O); ^13^C NMR (125 MHz, DMSO-*d*_6_) δ 168.4, 140.4, 132.8, 130.0, 128.9, 127.2, 126.1, 36.2; IR (KBr): cm^−1^ 3278, 3065, 3045, 2937, 1651, 1593, 1571, 1502, 1486, 1454, 1420, 1388, 1310, 1277, 1162, 1119, 1034, 1003; HRMS (ESI): *m/z* [M + H]^+^ calcd for C_16_H_15_N_2_O_2_: 267.11280, found: 267.11304.

#### 3.1.4. 5-benzyl-12-(2-(dimethylamino)ethyl)-5,12-dihydrodibenzo[*b*,*f*][1,4]diazocine-6,11-dione (**3m**)

To a solution of 5-benzyl-5,12-dihydrodibenzo[*b*,*f*][1,4]diazocine-6,11-dione (**3j**) (1.5 g, 4.6 mmol, 1.0 equiv.) in anhydrous DMSO (30 mL), 60% sodium hydride in mineral oil (0.45 g, 11.4 mmol, 2.5 equiv.) was added, and the resulting mixture was stirred for 30 min. After the addition of 2-chloro-*N*,*N*-dimethylethan-1-amine hydrochloride (0.82 g, 5.7 mmol, 1.2 equiv.) the reaction mixture was stirred for 18 h at room temperature, then was poured into water and the crude product was extracted with ethyl acetate (3 × 50 mL). The organic phase was washed with brine (1 × 50 mL) and dried over anhydrous magnesium sulfate. The crude product was purified by column chromatography (triethylamine:ethyl acetate 5:95 *v*/*v*) giving **3m** as white crystals (1.29 g, yield 71%). M.p. 154.0–155.0 °C, R.f. = 0.38 (triethylamine:ethyl acetate 5:95 *v*/*v*). ^1^H NMR (500 MHz, DMSO-*d*_6_) δ 7.40–7.14 (m, 13H, H_Ar_), 5.30 (d, 1H, J = 15.0 Hz, CH_2_),4.80 (d, 1H, J = 15.0 Hz, CH_2_), 3.47 (dt, 1H, J = 7.0 Hz, J = 14.0 Hz, CH_2_), 2.71 (dt, 1H, J = 7.0 Hz, J = 14.0 Hz, CH_2_), 2.34 (t, 2H, J = 7.0 Hz, CH_2_), 2.01 (s, 6H, 2 × Me); ^13^C NMR (125 MHz, DMSO-*d*_6_) δ 168.5, 168.3, 140.2, 139.3, 137.2, 133.0, 132.4, 130.1, 130.0, 129.0, 128.8, 128.7 (2 × C), 128.6 (2 × C), 127.7, 127.5 (2 × C), 126.0, 125.9, 56.5, 51.7, 46.4, 45.1; IR (KBr): cm^−1^ 3276, 3061, 2982, 2952, 2858, 2823, 2798, 2775, 1981, 1653, 1583, 1572, 1498, 1486, 1458, 1440, 1401, 1380, 1364, 1330, 1319, 1239, 1218, 1195, 1154, 1120, 1080, 1060, 1042, 1025; HRMS (ESI): *m/z* [M + H]^+^ calcd for C_25_H_26_N_3_O_2_: 400.20195, found: 400.20245.

#### 3.1.5. Ethyl 2-(8,9-dichloro-12-methyl-6,11-dioxo-11,12-dihydrodibenzo[*b*,*f*] [1,4]diazocin-5(6*H*)-yl)acetate (**3n**)

To a solution of 8,9-dichloro-5-methyl-5,12-dihydrodibenzo[*b*,*f*][1,4]diazocine-6,11-dione (**3g**) (2.0 g, 6.2 mmol, 1.0 equiv.) in anhydrous DMSO (20 mL), 60% sodium hydride in mineral oil (0.37 g, 9.4 mmol, 1.5 equiv.) was added and the resulting mixture was stirred for 30 min. After the addition of ethyl bromoacetate (1.0 mL, 9.4 mmol, 1.5 equiv.) the reaction mixture was stirred for 18 h at room temperature, then was poured into water and the crude product was extracted with ethyl acetate (3 × 50 mL). The organic phase was washed with brine (1 × 50 mL) and dried over anhydrous magnesium sulfate. The crude product was purified by column chromatography (hexane:ethyl acetate 7:3 *v*/*v*) giving **3n** as white crystals (1.93 g, yield 76%). M.p. 114.0–115.0 °C, R.f. = 0.32 (hexane:ethyl acetate 6:4 *v*/*v*). ^1^H NMR (500 MHz, DMSO-*d*_6_) δ 7.58 (s, 1H, H_Ar_), 7.52–7.42 (m, 3H, H_Ar_), 7.37–7.29 (m, 2H, H_Ar_), 4.78 (d, 1H, J = 17.0 Hz, CH_2_), 4.49 (d, 1H, J = 17.0 Hz, CH_2_), 4.15 (q, 2H, J = 7.0 Hz, CH_2_), 3.36 (s, 3H, Me, partially overlapped with H_2_O signal), 1.20 (t, 3H, J = 7.0 Hz, Me); ^13^C NMR (125 MHz, DMSO-*d*_6_) δ 168.1, 166.5, 165.7, 140.2, 138.9, 133.3, 133.0, 132.1, 129.4, 129.3, 128.5, 128.3, 127.6, 127.1, 61.0, 51.0, 36.6, 14.0; IR (KBr): cm^−1^ 3291, 3087, 2986, 2927, 2849, 1757, 1660, 1587, 1548, 1501, 1475, 1449, 1416, 1390, 1353, 1319, 1254, 1204, 1156, 1135, 1096, 1061, 1025, 1011; HRMS (ESI): *m/z* [M + H]^+^ calcd for C_19_H_17_N_2_O_4_Cl_2_: 407.05599, 409.05304, found: 407.05674, 409.05381.

#### 3.1.6. Acylation of 5-benzyl-5,12-dihydrodibenzo[*b,f*][1,4]diazocine-6,11-dione (**3j**)

A suspension of 5-benzyl-5,12-dihydrodibenzo[*b*,*f*][1,4]diazocine-6,11-dione (**3j**) (1.0 g, 3.0 mmol) in acetic anhydride (15 mL) was refluxed for 1 h, then the excess of anhydride was evaporated and crude 5-acetyl-12-benzyl-5,12-dihydrodibenzo[*b*,*f*][1,4]diazocine-6,11-dione (**3o**) was crystallized from mixture of cyclohexane:ethyl acetate 9:1 giving **3o** as colorless crystals (1.06 g, 94% yield). M.p. 174.0–175.0 °C, R.f. = 0.54 (hexane:ethyl acetate 6:4 *v*/*v*). ^1^H NMR (500 MHz, DMSO-*d*_6_) δ 7.49–7.17 (m, 13H, H_Ar_), 5.21 (d, 1H, J = 14.5 Hz, CH_2_), 4.78 (d, 1H, J = 14.5 Hz, CH_2_), 2.31 (s, 3H, Me); ^13^C NMR (125 MHz, DMSO-*d*_6_) δ 171.0, 169.5, 168.3, 139.7, 136.8, 136.3, 132.7, 132.0, 131.7, 130.6, 130.2, 130.1, 128.7 (2 × C), 128.4, 128.3 (2 × C), 127.6, 127.0, 126.52, 126.47, 51.7, 26.4; IR (KBr): cm^−1^ 3064, 3004, 2934, 1949, 1831, 1709, 1649, 1595, 1572, 1496, 1455, 1433, 1393, 1368, 1303, 1278, 1253, 1203, 1156, 1139, 1078, 1045, 1019; HRMS (ESI): *m/z* [M + H]^+^ calcd for C_23_H_19_N_2_O_3_: 371.13902, found: 371.13952.

#### 3.1.7. 1,3,4,6-tetrahydrobenzo[*b*][1,4]diazocine-2,5-dione (**6**)

Following the procedure of Elgureo et al. [20] purified by column chromatography methanol:chloroform 1:9 *v*/*v*, yield 27%, white crystals, m.p. 244.0–245.0 °C, R.f. = 0.22 (methanol:chloroform 1:9 *v*/*v*).^1^H NMR (500 MHz, DMSO-*d*_6_) δ 9.55 (2, 2H, 2 × NH); 7.34–7.27 (m, 2H, H_Ar_), 7.22–7.15 (m, 2H, H_Ar_), 2.33 (bs, 4H, 2 × CH_2_); ^13^C NMR (125 MHz, DMSO-*d*_6_) δ 172.2, 134.8, 127.4, 126.9, 30.5; IR (KBr): cm^−1^ 3179, 3051, 2920, 1992, 1947, 1681, 1644, 1587, 1504, 1455, 1409, 1332, 1308, 1232, 1176, 1124, 1050, 1010; HRMS (ESI): *m/z* [M + H]^+^ calcd for C_23_H_19_N_2_O_3_: 191.08150, found: 191.08141.

#### 3.1.8. Synthesis of **9a**

To a solution of tetraethyl benzene-1,2,4,5-tetracarboxylate (8.0 g, 21.9 mmol, 1.0 equiv.) and phenylenediamine (4.7 g, 43.7 mmol, 2.0 equiv.) in anhydrous THF (100 mL), 60% sodium hydride (3.7 g, 91.8 mmol, 4.2 equiv.) was added and the resulting mixture was refluxed overnight. Then the mixture was poured into water (500 mL), acidified with concentrated HCl to pH 2. The obtained precipitate, containing crude **9a**, was filtered through a Schott funnel, washed several times with ethyl acetate and crystallized from the mixture of DMSO:NMP 1:1. The filtrate was extracted with ethyl acetate (3 × 100 mL), the combined organic phases containing tricyclic product **3k** were washed with brine (1 × 50 mL) and dried over magnesium sulfate. Evaporation of the solvent gave the crude product which was purified by column chromatography (100% ethyl acetate), giving the final product **3k** as a white crystalline solid.

**9a**: yield 59% (5.13 g), white crystals, m.p. 375.0–376.0 °C. ^1^H NMR (500 MHz, DMSO-*d*_6_) δ 10.16. 10.10 (2 × s, 4H, 4 × NH), 7.30–7.16 (m, 8H, H_Ar_), 7.12–7.00 (m, 2H, H_Ar_); ^13^C NMR (125 MHz, DMSO-*d*_6_) δ, main conformer only: 168.5, 134.3, 133.7, 127.5, 126.9, 124.6; IR (KBr): cm^−1^ 3465, 3189, 3059, 2886, 1669, 1592, 1552, 1503, 1415, 1371, 1302, 1229, 1154, 1113, 1038; HRMS (ESI): *m/z* [M + H]^+^ calcd for C_22_H_15_N_4_O_4_: 399.10878, found: 399.10842.

*diethyl 6,11-dioxo-5,6,11,12-tetrahydrodibenzo[b,f][1,4]diazocine-8,9-dicarboxylate* (**3k**)**:** yield 6% (0.5 g), white crystals, m.p. 242.0–243.0 °C, R.f. = 0.28 (hexane:ethyl acetate 2:8 *v*/*v*). ^1^H NMR (500 MHz, DMSO-*d*_6_) δ 10.48 (s, 2H, 2 × NH), 7.67 (s, 2H, H_Ar_), 7.26–7.18 (m, 4H, H_Ar_), 4.25 (q, 4H, J = 7.0 Hz, 2 × CH_2_), 1.24 (t, 6H, J = 7.0 Hz, 2 × Me); ^13^C NMR (125 MHz, DMSO-*d*_6_) δ 168.5, 165.5, 134.6, 134.4, 133.1, 128.0, 127.31, 127.25, 61.9, 13.8; IR (KBr): cm^−1^ 3195, 3062, 2984, 2937, 2901, 1736, 1718, 1665, 1592, 1561, 1501, 1444, 1391, 1363, 1291, 1168, 1144, 1110, 1015; HRMS (ESI): *m/z* [M + H]^+^ calcd for C_20_H_19_N_2_O_6_: 383.12376, found: 383.12371.

#### 3.1.9. General Procedure for Alkylation of **9a**

To a solution of **9a** (1 equiv.) in anhydrous DMSO (2 mL/mmol), 60% sodium hydride in mineral oil (5 equiv.) was added. The resulting mixture was stirred for 30 min, then appropriate halide (6 eq.), methyl iodide or benzyl bromide, was added. The reaction mixture was stirred for 18 h at room temperature, then was poured into water and the precipitated product was filtered through a Schott funnel. The obtained solids were washed with ethyl acetate, then with acetone and dried in air to give pure products.

(**9b**) yield 82%, white crystals, m.p. 400.0 °C, R.f. = 0.56 (methanol:chloroform 1:9 *v*/*v*). ^1^H NMR (500 MHz, CF_3_COOD) δ 7.42 (s, 2H, H_Ar_), 7.37–7.05 (m, 8H, H_Ar_), 3.46, 3.41 (2 × s, 12H, 4 × Me); ^13^C NMR (125 MHz, CF_3_COOD) δ, both conformers: 172.2, 141.0, 140.4, 136.3, 136.2, 133.1, 132.4, 128.8, 128.7, 127.8, 127.2; IR (KBr): cm^−1^ 3464, 3040, 2936, 1649, 1594, 1553, 1503, 1448, 1418, 1383, 1310, 1272, 1177, 1122, 1045; HRMS (ESI): *m/z* [M + H]^+^ calcd for C_26_H_23_N_4_O_4_: 455.17138, found: 455.17135;

(**9c**) yield 90%, white crystals, m.p. 385.0–386.0 °C, R.f. = 0.67 (hexane:ethyl acetate 2:8 *v*/*v*). ^1^H NMR (500 MHz, DMSO-*d*_6_) δ 7.45–7.30 (m, 12H, H_Ar_), 7.28 (s, 2H, H_Ar_), 7.27–7.20 (m, 4H, H_Ar_), 7.17–7.01 (m, 12H, H_Ar_), 4.62 (d, 4H, J = 15.0 Hz, 2 × CH_2_), 3.96 (d, 4H, J = 15.0 Hz, 2 × CH_2_); ^13^C NMR (125 MHz, DMSO-*d*_6_) δ 166.7, 138.9, 136.8, 134.0, 129.5, 128.9, 128.4, 127.9, 127.7, 123.9, 51.2; IR (KBr): cm^−1^ 3290, 3084, 3062, 3029, 2935, 1955, 1665, 1651, 1598, 1584, 1495, 1455, 1431, 1399, 1362, 1324, 1298, 1261, 1224, 1201, 1157, 1148, 1119, 1079, 1053, 1030; HRMS (ESI): *m/z* [M + H]^+^ calcd for C_50_H_38_N_4_O_4_: 759.29658, found: 759.29776;

#### 3.1.10. Rearrangement of 5,12-dihydrodibenzo[*b*,*f*][1,4]diazocine-6,11-dione (**3a**) to 2-(2-aminophenyl)isoindoline-1,3-dione (**10**)

To a suspension of 5,12-dihydrodibenzo[*b*,*f*][1,4]diazocine-6,11-dione (**3a**) (1.0 g, 4.2 mmol, 1 equiv.) in DMSO, 60% (0.18 g, 4.6 mmol, 1.1 equiv.) sodium hydride in mineral oil was added and the resulting mixture was stirred overnight at room temperature. The red clear solution which was formed was poured into water and extracted with ethyl acetate (3 × 50 mL), the organic phase was washed with brine (1 × 50 mL) and dried over anhydrous magnesium sulfate. The crude product was purified by column chromatography (hexane:ethyl acetate 7:3 *v*/*v*), giving **10** as a yellow solid (0.58 g, 58% yield). m.p. 195.0–196.0 °C, R.f. = 0.35 (hexane:ethyl acetate 6:4 *v*/*v*). ^1^H NMR (500 MHz, DMSO-*d*_6_) δ 7.95–7.83 (m, 4H, H_Ar_), 7.12 (t, 1H, J = 7.5 Hz, H_Ar_), 7.01 (d, 1H, J = 7.5 Hz, H_Ar_), 6.75 (d, 1H, J = 7.5 Hz, H_Ar_), 6.56 (t, 1H, J = 7.5 Hz, H_Ar_), 6.36 (s, 2H, NH_2_); ^13^C NMR (125 MHz, DMSO-*d*_6_) δ 167.6, 146.5, 134.1, 132.5, 130.0, 129.7, 123.1, 115.9, 115.32, 115.28; IR (KBr): cm^−1^ 3458, 3380, 3030, 1783, 1708, 1623, 1578, 1502, 1461, 1377, 1314, 1271, 1216, 1159, 1143, 1100, 1083, 1049, 1026; HRMS (ESI): *m/z* [M + H]^+^ calcd for C_14_H_11_N_2_O_2_: 239.08150, found: 239.08171;

#### 3.1.11. Synthesis of 4,5-dichloro-*N*^1^-methylbenzene-1,2-diamine (**4e**)

To a solution of 4,5-dichloro-2-nitroaniline (30.0 g, 0.14 mol, 1.0 equiv.) in DMF (150 mL), 60% sodium hydride in mineral oil (6.4 g, 0.16 mol, 1.1 equiv.) was added. After 30 min of stirring in room temperature, methyl iodide (13.5 mL, 0.22 mol, 1.5 equiv.) was added to the reaction mixture and the solution warmed up spontaneously. After 1h, the reaction mixture was poured into water (500 mL) and the product was extracted with ethyl acetate (3 × 100 mL). The organic phase was washed with brine (1 × 50 mL) and dried over anhydrous magnesium sulfate. The drying agent was filtered off, the resulting filtrate was concentrated under reduced pressure and left in the fridge for crystallization. The orange crystals of 4,5-dichloro-*N*-methyl-2-nitroaniline were formed, which were washed with a small amount of ethyl acetate and dried. Yield: 25.0 g (78%). A slurry of 4,5-dichloro-*N*-methyl-2-nitroaniline (15.0 g, 0.07 mol, 1.0 equiv.) and iron powder (19.0 g, 0.34 mol, 5.0 equiv.) in a mixture of 95% ethanol (200 mL) and glacial acetic acid (100 mL) was stirred at 60 °C for 2h. The progress of reaction was monitored by TLC, when the reaction was completed; the excess of volatiles was evaporated under reduced pressure. The obtained residue was treated with water and the pH was adjusted to ca. 8 using sodium hydroxide. The crude product was extracted with ethyl acetate (3 × 150 mL), organic phase was washed with brine (1 × 50 mL) and dried over anhydrous magnesium sulfate. Evaporation of the solvent resulted in the 4,5-dichloro-*N*^1^-methylbenzene-1,2-diamine (**4e**), obtained as a brown solid (12.6 g, 97% yield) which was used in the next step without further purification. M.p. 100.0–101.0 °C, R.f. = 0.35 (hexane:ethyl acetate 9:1 *v*/*v*). ^1^H NMR (500 MHz, CDCl_3_) δ 6.72 (s, 1H, H_Ar_), 6.62 (s, 1H, H_Ar_), 3.30 (bs, 3H, NH_2_+NH), 2.81 (s, 3H, Me); ^13^C NMR (125 MHz, CDCl_3_) δ 138.8, 132.8, 123.2, 120.3, 117.1, 112.0, 31.0; HRMS (ESI): *m/z* [M + H]^+^ calcd for C_7_H_9_Cl_2_N_2_: 191.01373, 193.01078, 175.00785, found: 191.01403, 193.01110, 175.00788;

#### 3.1.12. Synthesis of *N*^1^-benzylbenzene-1,2-diamine (**4f**)

A solution of 1-fluoro-2-nitrobenzene (15.0 mL, 0.14 mol, 1.0 equiv.), benzylamine (17.0 mL, 0.15 mol, 1.1 equiv.) and TEA (25.0 mL, 0.18 mol, 1.3 equiv.) in DMSO (10 mL) was stirred at 100 °C for 18 h. After cooling down, the obtained orange solution was poured into water and the reaction product was extracted with ethyl acetate (3 × 100 mL). The organic phase was washed with brine (1 × 50 mL) and dried over anhydrous magnesium sulfate. Evaporation of the solvent gave *N*-benzyl-2-nitroaniline as a red solid (29.0 g, 89.5% yield), which was used in the next step without further purification. A slurry of *N*-benzyl-2-nitroaniline (10.0 g, 44.0 mmol, 1.0 equiv.) and iron powder (12.3 g, 220.0 mmol, 5.0 equiv.) in a mixture of 95% ethanol (100 mL) and glacial acetic acid (50 mL) was stirred at 60 °C for 2 h. The progress of reaction was monitored by TLC, when the reaction was completed, the excess of volatiles was evaporated under reduced pressure. The obtained residue was treated with water, the pH was adjusted to ca. 8 using sodium hydroxide and the reaction product was extracted with ethyl acetate (3 × 150 mL). The organic phase was washed with brine (1 × 50 mL) and dried over anhydrous magnesium sulfate. The drying agent was filtered off and the resulting filtrate was evaporated under reduced pressure, giving *N*^1^*-*benzylbenzene-1,2-diamine (**4f**) as a brown solid (8.3 g, 96% yield) used in the next step without further purification. M.p. 49.0–50.0 °C, R.f. = 0.33 (hexane:ethyl acetate 9:1 *v*/*v*). ^1^H NMR (500 MHz, CDCl_3_) δ 7.48–7.43 (m, 2H, H_Ar_), 7.43–7.37 (m, 2H, H_Ar_), 7.36–7.31 (m, 1H, H_Ar_), 6.89-6.83 (m, 1H, H_Ar_), 6.80-6.69 (m, 3H, H_Ar_), 4.35 (s, 2H, CH_2_), 3.46 (bs, 3H, NH_2_ + NH); ^13^C NMR (125 MHz, CDCl_3_) δ 139.4, 137.7, 134.2, 128.7, 127.9, 127.3, 120.8, 118.9, 116.6, 112.1, 48.7; HRMS (ESI): *m/z* [M + H]^+^ calcd for C_13_H_15_N_2_: 199.12298, found: 199.12313;

#### 3.1.13. General Procedure for Synthesis of Phthalic Acids Methyl Esters **5b–d**

To a stirred slurry of appropriate phthalic acid (0.1 mol) in methanol (200 mL), concentrated sulphuric acid (20 mL) was added dropwise (caution: exothermic!). The reaction mixture was stirred for ten minutes in room temperature which led to the dissolution of phthalic acid. The obtained solution was refluxed for 18 h, then the excess of methanol was evaporated under reduced pressure and the remaining residue was poured into water. The pH was adjusted to ca. 8 by the addition of solid sodium hydroxide and the reaction product was extracted with ethyl acetate (3 × 100 mL) The organic phase was washed with brine (1 × 50 mL) and dried over anhydrous magnesium sulfate. The drying agent was filtered off and the resulting filtrate was evaporated under reduced pressure. Evaporation of the solvent gave the crude product which was used in next step without further purification.

*dimethyl 4-nitrophthalate* (**5b**): yield 76%, beige solid, m.p. 66.0–67.0 °C, R.f. = 0.20 (hexane:ethyl acetate 9:1 *v*/*v*). ^1^H NMR (500 MHz, CDCl_3_) δ 8.58 (d, 1H, J = 2.5 Hz, H_Ar_), 8.36 (dd, 1H, J = 2.5 Hz, J = 8.5 Hz, H_Ar_), 7.81 (d, 1H, J = 8.5 Hz, H_Ar_), 3.94 (s, 3H, Me); 3.93 (s, 3H, Me); ^13^C NMR (125 MHz, CDCl_3_) δ 166.8, 165.5, 148.9, 138.2, 132.7, 130.1, 126.2, 124.5, 53.4; HRMS (ESI): *m/z* [M + H]^+^ calcd for C_10_H_10_NO_6_: 240.05026, found: 240.05031;

*dimethyl 4,5-dichlorophthalate* (**5c**): yield 91%, pale oil, R.f. = 0.49 (hexane:ethyl acetate 9:1 *v*/*v*). ^1^H NMR (500 MHz, CDCl_3_) δ 7.77 (s, 2H, H_Ar_), 3.88 (s, 6H, 2 × Me); ^13^C NMR (125 MHz, CDCl_3_) δ 166.0, 135.8, 131.4, 131.0, 53.1; HRMS (ESI): *m/z* [M + H]^+^ calcd for C_10_H_9_O_4_Cl_2_: 262.98724, 264.98429, found: 262.98744, 264.98445;

*dimethyl 3,4,5,6-tetrachlorophthalate* (**5d**): yield 35%, white solid, m.p. 92.0–93.0 °C, R.f. = 0.49 (hexane:ethyl acetate 9:1 *v*/*v*). ^1^H NMR (500 MHz, CDCl_3_) δ 3.92 (s, 6H, 2 × Me); ^13^C NMR (125 MHz, CDCl_3_) δ 164.3, 136.2, 132.1, 130.7, 53.6; HRMS (ESI): *m/z* [M + H]^+^ calcd for C_10_H_7_O_4_Cl_4_: 330.90930, 332.90635, 334.90340, found: 330.90909, 332.90613, 334.90316.

#### 3.1.14. Synthesis of dimethyl thiophene-3,4-dicarboxylate (**5e**)

To a slurry of tiophene-3,4-dicarboxylic acid (4.0 g, 23.3 mmol, 1.0 equiv.) and potassium carbonate (8.1 g, 58.3 mmol, 2.5 equiv.) in DMF (20 mL), methyl iodide (3.2 mL, 51.1 mmol, 2.2 equiv.) was added dropwise. The resulting mixture was stirred for 18 h at 40 °C, then poured into water (100 mL) and the pH was adjusted to ca. 8 using sodium hydroxide. The reaction product was extracted with ethyl acetate (3 × 50 mL), the organic phase was washed with brine (1 × 50 mL) and dried over anhydrous magnesium sulfate. The drying agent was filtered off, the resulting filtrate was concentrated under reduced pressure and left in the fridge for crystallization. Big, colorless crystals of **5e** were formed (1.83 g, 40% yield). M.p. 59.0–60.0 °C, R.f. = 0.25 (hexane:ethyl acetate 9:1 *v*/*v*). ^1^H NMR (500 MHz, CDCl_3_) δ 7.84 (s, 2H, H_Ar_), 3.85 (s, 6H, 2 × Me); ^13^C NMR (125 MHz, CDCl_3_) δ 163.5, 133.2, 131.9, 52.42; HRMS (ESI): *m/z* [M + H]^+^ calcd for C_8_H_9_O_4_S: 201.02161, found: 201.02177;

#### 3.1.15. Synthesis of tetraethyl benzene-1,2,4,5-tetracarboxylate (**8**)

Following the procedure of Berthold et.al. [27] the slurry of pyromellitic dianhydride (25.0 g, 0.11 mol) in absolute ethanol (250 mL) was refluxed for 2 h, then sulfuric acid (15 mL) was added dropwise. The reaction mixture was refluxed for an additional 18 h, then the excess ethanol was evaporated. The residue was poured into water and the pH was adjusted to ca. 8 using sodium hydroxide. The reaction product was extracted with ethyl acetate (3 × 100 mL), the organic phase was washed with brine (1 × 50 mL) and dried over anhydrous magnesium sulfate. Evaporation of the solvent gave **8** as an oily residue, which solidified upon storage (27.16 g, 64% yield). M.p. 54.0–55.0 °C, R.f. = 0.28 (hexane:ethyl acetate 9:1 *v*/*v*). ^1^H NMR (500 MHz, CDCl_3_) δ 8.02 (s, 2H, H_Ar_), 4.36 (q, 8H, J = 7.0 Hz, 4 × CH_2_), 1.35 (t, 12H, J = 7.0 Hz, 4 × Me); ^13^C NMR (125 MHz, CDCl_3_) δ 166.1, 134.4, 129.6, 62.3, 14.1; HRMS (ESI): *m/z* [M + H]^+^ calcd for C_18_H_23_O_8_: 367.13674, found: 367.13834;

### 3.2. X-ray Data Collection and Data Refinement

Good quality single-crystals of **3a**, **3g–j**, **9a**, **9c**, and **10**, were selected for the X-ray diffraction experiments at *T* = 100(2) K. Diffraction data were collected on the Agilent Technologies (Santa Clara, CA, USA) SuperNova Dual Source diffractometer with Cu*K*α radiation (*λ* = 1.54184 Å) (**3a**, **3g–j**, **9a**, **9c**, and **10**) and the Agilent Technologies SuperNova Single Source diffractometer with Mo*K*α radiation (*λ* = 0.71073 Å) (**6**), using CrysAlis RED software [28]. The multi-scan empirical absorption correction using spherical harmonics (**3a**, **3i**, **3j**, **6**, **9c**, and **10**), the analytical numeric absorption correction using a multifaceted crystal model based on expressions derived by Clark and Reid [29] (**3g** and **9a**) and numerical absorption correction based on gaussian integration over a multifaceted crystal model (**3h**), implemented in SCALE3 ABSPACK scaling algorithm were applied [28]. The structural determination procedure was carried out using the SHELX package [30]. The structures were solved with direct methods and then successive least-square refinement was carried out based on the full-matrix least-squares method on *F*^2^ using the SHELXL program [30]. All H-atoms linked to the N and O-atoms were located on Fourier differential maps and refined with *U*_iso_(H) = x*U*_eq_(N,O), where x = 1.2 for the amine and 1.5 for the hydroxyl H-atoms, respectively. In all the cases, the N−H bonds were subject to the DFIX 0.87 restraint. In the case of **3i,** the length of the O−H bond was restrained to 0.85 Å, and the distance between the H-atoms of water was restrained to 1.39 Å. Other H-atoms were positioned geometrically, with C–H equal to 0.93, 0.96 and 0.97 Å for the aromatic, methyl and methylene H-atoms, respectively, and constrained to ride on their parent atoms with *U*_iso_(H) = x*U*_eq_(C), where x = 1.2 for the aromatic and methylene H-atoms, and 1.5 for the methyl H-atoms, respectively. The **10** was refined as two-component twin with a 0.502(2):0.498(2) domain ratio (second component was rotated by the –179.98° around [100] (direct)). The molecular interactions in crystals have been identified using the PLATON program [31]. The figures for this publication were prepared using Olex2 and Mercury programs [32,33].

### 3.3. Cell Culturing

HeLa, U87, BICR18, EUFA30 cell lines were cultured in DMEM medium (Life Technology) supplemented with 10% fetal bovine serum (Life Technology) and 0.1% antibiotics, penicillin and streptomycin (Life Technology). Cells were grown in a humidified atmosphere of CO_2_/air (5/95%) at 37 °C.

### 3.4. Cytotoxicity Assay

Exponentially growing cells were seeded onto a 96-well plate at the density of 2 × 10^3^ cells/well, cultured for 18 h, and treated with newly synthesized derivatives at concentrations 1–300 µM, or with DMSO as a control, for 24 or 48 h. Alamar Blue (Invitrogen) was added accordingly to manufacturer protocol. After 4 h, light emission at 590 nm was measured with excitation at 560 nm using a scanning multiwell spectrophotometer (DTX 880, Beckman Coulter). The experiments were carried out at least three times with three replicates for each inhibitor concentration. After background subtraction, inhibition rate, IC_50_ was calculated as the concentration of the component inhibiting cell growth by 50%. All the calculations were performed using Origin 9.0 software.

### 3.5. Flow Cytometry

The Annexin V-FITC apoptosis detection kit (BD Biosciences) was used to detect apoptosis by flow cytometry. Cells were seeded at Petri dishes, cultured for 18 h with tested agents applied for 24 h. DMSO was added as the non-treated control and camptothecin (CPT) as the positive control. Subsequently, cells were washed with PBS, resuspended in binding buffer at a concentration of 2 × 10^6^ cells/mL, and anti-Annexin V FITC-conjugated antibody and propidium iodide were added to 100 µL aliquots. The mixtures were incubated for 15 min at room temperature, supplemented with binding buffer to 500 µL and processed by BD FACSCalibur (BD Biosciences). Data were analyzed in Flowing Software version 2.5.1 (Flowing Software, http://www.uskonaskel.fi/flowingsoftware).

## 4. Conclusions

We have shown, that previously unknown, unsymmetrically *N*-substituted and *N*,*N*-disubstituted 5,12-dihydrodibenzo[*b*,*f*][1,4]diazocine-6,11-diones, bearing various substituents on the dilactam ring, could be obtained in our novel, convenient protocol for the synthesis. The developed procedure allows for the introduction of diverse modifications and side chains into the dilactam ring as well as various functional groups attached to the benzene rings. The investigated synthesis was proved useful by obtaining five dilactam-unsubstituted (**3a–d**, **3k**) and twelve dilactam-substituted (**3e–j**, **3l–o**) tricyclic dibenzodiazocine derivatives and one benzothienodiazocine (**3i**). We have shown that the desired modifications of the dibenzodiazocine scaffold can be introduced both at the stage of appropriate selection of substrates for synthesis and subsequent post-cyclisation modifications, using the alkylation or acylation reactions of the resulting tricyclic system. Such compounds, due to their structural similarities to tricyclic dibenzodiazepine drugs, could be treated as a privileged structures, useful scaffolds for design and discovery of novel bioactive substances. The performed single-crystal X-ray structural analysis revealed the structural features of newly synthesized compounds, which supports design and development of new chemical materials or therapeutic agents with given or assumed chemical or biological properties. The investigated method, also allowing the synthesis of novel pentacyclic scaffold, consists of three benzene and two diazocine rings (**9a–c**), and led to the observation of a new type of rearrangement, contraction of the diazocine ring to the isoindoline ring (**10**). Although the synthesized compounds, tested on selected cancerous and non-cancerous cell lines, exhibited a rather weak cytotoxic effect, our synthetic methods are useful to design compounds with improved biological activity. Such studies on the biological applications of dibenzodiazocine derivatives are ongoing in our laboratory and will be published in due course.

## Figures and Tables

**Table 1:** Compounds synthesized in the reaction shown in Scheme 1.

**Amine**	**Ester**	**Product**
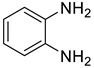	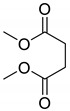	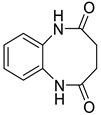
**4a**	**7**	**6** (27%)
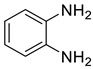	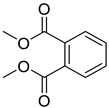	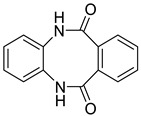
**4a**	**5a**	**3a** (78%)
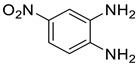	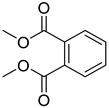	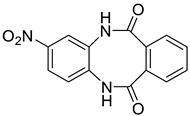
**4b**	**5a**	**3b** (53%)
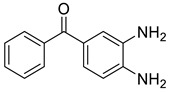	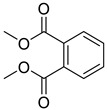	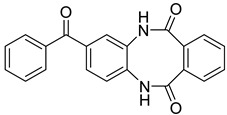
4**c**	**5a**	**3c** (55%)
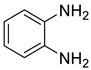	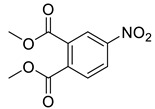	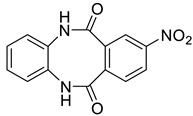
**4a**	**5b**	**3d** (50%)
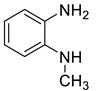	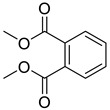	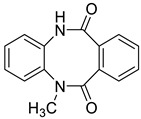
**4d**	**5a**	**3e** (61%)
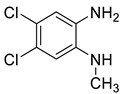	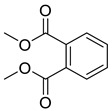	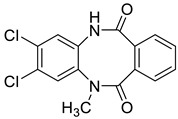
**4e**	**5a**	**3f** (55%)
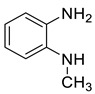	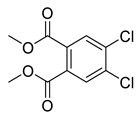	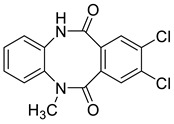
**4d**	**5c**	**3g** (67%)
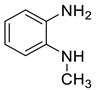	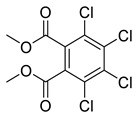	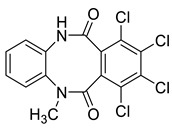
**4d**	**5d**	**3h** (15%)
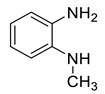	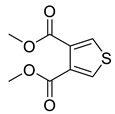	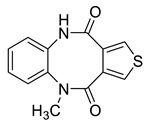
**4d**	**5e**	**3i** (47%)
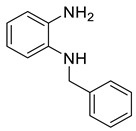	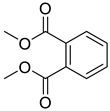	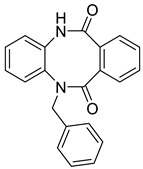
**4f**	**5a**	**3j** (74%)

**Table 2:** The values of geometric parameters describing the conformation of molecules of investigated tri- and pentacyclic benzodiazocine derivatives.

Compound	Geometric Parameter				
	*α* [°]	*α’* [°]	*D* [Å]	*D’* [Å]	*β*
**3a**	87.68(2)		4.695(2)		
**3g**	86.68(6)		4.680(2)		
**3h** (molecule A/molecule B)	72.3(3)/79.9(3)		4.231(5)/4.349(6)		
**3i**	87.05(6)		4.372(2)		
**3j**	87.74(4)		4.567(7)		
**9a**	88.82(7)	88.82(7)	4.589(2)	4.589(2)	
**9c**	64.78(6)	69.52(6)	4.057(2)	4.170(2)	45.74(7)

Where: *α* and *α’*: angle between mean-planes defined by the non-hydrogen atoms of the outer aromatic rings of the benzodiazocine moiety; *D* and *D’*: distance between geometrical centers of gravity of outer aromatic rings of the dibenzodiazocine moiety; *β*: angle between mean-planes defined by the non-hydrogen atoms of the outer aromatic rings of fused pentacyclic benzodiazocine moiety (see Figure 3).

**Table 3:** IC_50_ (μM) of 5,12-dihydrodibenzo[*b*,*f*][1,4]diazocine-6,11-diones based on the survival of non-cancerous (EUFA30) and cancerous (HeLa, BICR18, and U87) cells after 24 and 48 h of treatment.

Compound	Cell Types							
	EUFA30		HeLa		BICR18		U87	
	24 h	48 h	24 h	48 h	24 h	48 h	24 h	48 h
**3a**	[21.2] * ± 4.8	[9.0] * ± 5.2	[20.7] * ± 2.4	[1.5] * ± 1.2	[7.1] * ± 2.7	N.A.	[17.6] * ± 4.5	N.A.
**3b**	[31.0] * ± 3.0	[18.6] * ± 1.8	[33.2] * ± 6.2	[4.7] * ± 1.5	[2.2] * ± 1.4	N.A.	[10.4] * ± 2.4	N.A.
**3c**	[28.8] * ± 5.1	N.A.	[14.5] * ± 4.4	N.A.	[10.0] * ± 3.3	N.A.	[15.0] * ± 9.2	N.A.
**3d**	[9.2] * ± 2.7	[4.2] * ± 2.9	[23.4] * ± 4.6	[3.9] * ± 2.9	[25.1] * ± 7.4	[2.5] * ± 5.3	[27.5] * ± 5.2	N.A.
**3e**	[31.6] * ± 8.9	[17.5] * ± 3.1	[19.8] * ± 3.3	[5.7] * ± 1.0	[22.7] * ± 8.6	N.A.	[34.1] * ± 3.2	N.A.
**3f**	**320.0** ± 36	[19.1] * ± 8.5	**298.4** ± 11.0	[7.8] * ± 2.1	[47.5] * ± 7.6	[7.6] * ± 4.2	**207.0** ± 23.6	[29.9] * ± 18.3
**3g**	[41.6] * ± 4.5	[29.5] * ± 9.9	[43.1] * ± 4.7	[5.3] * ± 2.3	**266.4** ± 27.6	[12.3] ± 1.8	**322.8** ± 23.9	[2.4] * ± 2.4
**3h**	**144.5** ± 10.6	**153.2** ± 4.1	**132.1** ± 6.0	**250.7** ± 5.1	**93.7** ± 5.2	**177.7** ± 3.0	**97.1** ± 13.2	**293.6** ± 22.4
**3i**	[29.4] * ± 6.2	[1.5] * ± 1.4	[14.8] * ± 6.8	[1.6] * ± 2.9	[24.0] * ± 5.7	N.A.	[36.6] * ± 4.8	[2.8] * ± 1.9
**3j**	[33.2] * ± 8.0	[32.6] * ± 2.5	[34.3] * ± 7.2	[3.3] * ± 2.3	[42.2] * ± 12.2	[6.1] * ± 3.0	[33.0] * ± 2.8	N.A.
**3k**	[36.7] * ± 5.0	N.A.	[28.2] * ± 1.5	N.A.	[16.8] * ± 5.9	[5.6] * ± 3.2	[37.2] * ± 5.5	[3.1] * ± 2.6
**3l**	[38.1] * ± 2.1	[19.5] * ± 5.8	[29.5] * ± 9.3	[2.8] * ± 1.6	[22.1] * ± 3.5	N.A.	[39.0] * ± 4.7	N.A.
**3m**	[42.2] * ± 8.0	[9.9] * ± 5.7	[23.4] * ± 9.3	N.A.	[30.1] * ± 9.7	[4.7] * ± 2.8	[36.8] * ± 2.8	[4.1] * ± 3.6
**3n**	[41.5] * ± 6.8	N.A.	**311.4** ± 14.1	N.A.	[20.9] * ± 10.2	N.A.	[30.7] * ± 8.7	N.A.
**3o**	**155.4** ± 14.6	[41.8] * ± 7.9	**169.1** ± 13.4	[33.4] * ± 8.9	**121.0** ± 11.4	**294.3** ± 3.2	**240.2** ± 6.1	[33.8] * ± 4.6
**6**	[29.4] * ± 5.9	[14.0] ± 6.1	[21.5] * ± 4.3	[4.3] * ± 1.4	[11.3] * ± 4.8	N.A.	[19.6] * ± 5.8	N.A.
**10**	[25.6] * ± 6.6	N.A.	[4.9] * ± 3.1	N.A.	[11.4] * ± 3.7	N.A.	[22.9] * ± 4.3	N.A.

* proliferation inhibition expressed as a percentage at highest concentration used: 300 µM. N.A.: not active.

## Supplementary Materials

^1^H-NMR (**3a–o**, **4e**, **4f**, **5b–e**, **6**, **8**, **9a–c**, **10**), ^13^C-NMR (**3a–o**, **4e**, **4f**, **5b–e**, **6**, **8**, **9a–c**, **10**), dept135 (**3a–o**, **4e**, **4f**, **5b–e**, **6**, **8**, **9a–c**, **10**) IR **(3a–o**, **6**, **9a–c**, **10**), HRMS **(3a–o**, **4e**, **4f**, **5b–e**, **6**, **8**, **9a–c**, **10**) and X-ray analysis data (**3a**, **3g**–**3j**, **6**, **9a**, **9c**, **10**) are available online. CCDC 1998020–1998028 contain the supplementary crystallographic data for this paper. These data can be obtained freely via http://www.ccdc.cam.ac.uk/data_request/cif, by e-mailing data_request@ccdc.cam.ac.uk or by contacting directly the Cambridge Crystallographic Data Centre (12 Union Road, Cambridge CB2 1EZ, UK. Fax: +44 1223 336033).
