# Supplementary material for: Unsymmetrically-Substituted 5,12-dihydrodibenzo[b,f][1,4]diazocine-6,11-dione Scaffold—A Useful Tool for Bioactive Molecules Design
Source: Molecules. 2020 Jun 20;25(12):2855. doi: 10.3390/molecules25122855 (PMC7356613; doi:10.3390/molecules25122855)
Supplement: Supplementary file 1 [file molecules-25-02855-s001.pdf]

molecules

Supplementary materials

Unsymmetrically-Substituted 5,12-dihydrodibenzo[*b*,*f*][1,4]diazocine-6,11-dione Scaffold—A Useful Tool for Bioactive Molecules Design — Crystallographic Data, 1H-NMR, 13C-NMR, dept135, ESI HRMS, IR spectra

Bartosz Bieszczad [1], Damian Garbicz [1], Damian Trzybiński [2], Marta K. Dudek [3], Krzysztof Woźniak [2], Elżbieta Grzesiuk [1] and Adam Mieczkowski [1,*]

[1] Institute of Biochemistry and Biophysics, Polish Academy of Sciences, 02-106 Warszawa, Poland; b.bieszczad@ibb.waw.pl (B.B.); dgarbicz@ibb.waw.pl (D.G.); elag@ibb.waw.pl (E.G.)

[2] Biological and Chemical Research Centre, University of Warsaw, 02-089 Warszawa, Poland; dtrzybinski@cnbc.uw.edu.pl (D.T.); kwozniak@chem.uw.edu.pl (K.W.)

[3] Centre of Molecular and Macromolecular Studies, Polish Academy of Sciences, 90-363 Łódź, Poland; mdudek@cbmm.lodz.pl

* Correspondence: amiecz@ibb.waw.pl

TABLE OF CONTENTS

1. **crystallographic data** (Figures S1–S7, Tables S1–S31)	**2–21**
2. **1H-NMR spectra** (Figures S8–S34)	**22–49**
3. **13C-NMR spectra** (Figures S35–S61)	**50–77**
4. **dept135 spectra** (Figures S62–S88)	**78–91**
5. **ESI HMRS spectra** (Figures S89–S115)	**92–118**
6. **IR spectra** (Figures S116–S135)	**119–138**

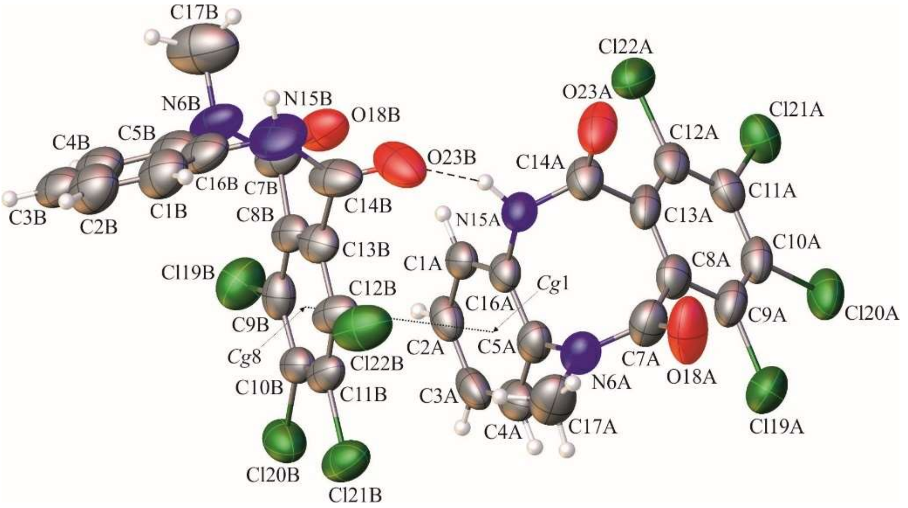

Figure S1. Asymmetric unit of the crystal lattice of **3h** with crystallographic atom numbering. Displacement ellipsoids are drawn at the 50% probability level. The H-atoms are shown as small spheres of arbitrary radius. The N−H···O hydrogen bond is represented by a dashed line, while the π-π contact by a dotted line. The *Cg*1 and *Cg*8 denote geometric centers of gravity of the aromatic rings defined by the C1A−C5A/C16A atoms.

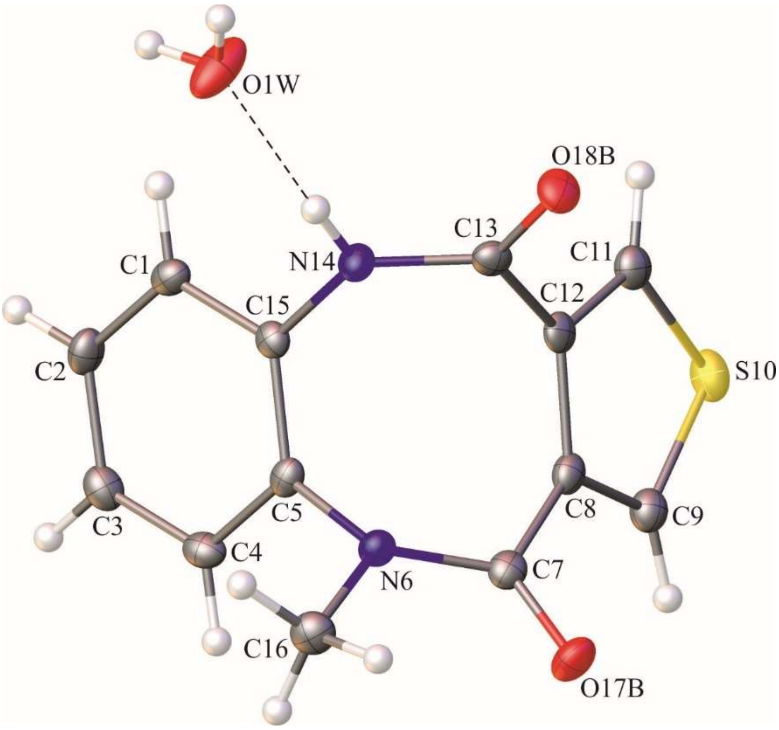

Figure S2. Asymmetric unit of the crystal lattice of **3i** with crystallographic atom numbering. Displacement ellipsoids are drawn at the 50% probability level. The H-atoms are shown as small spheres of arbitrary radius. The N−H···O hydrogen bond is represented by a dashed line.

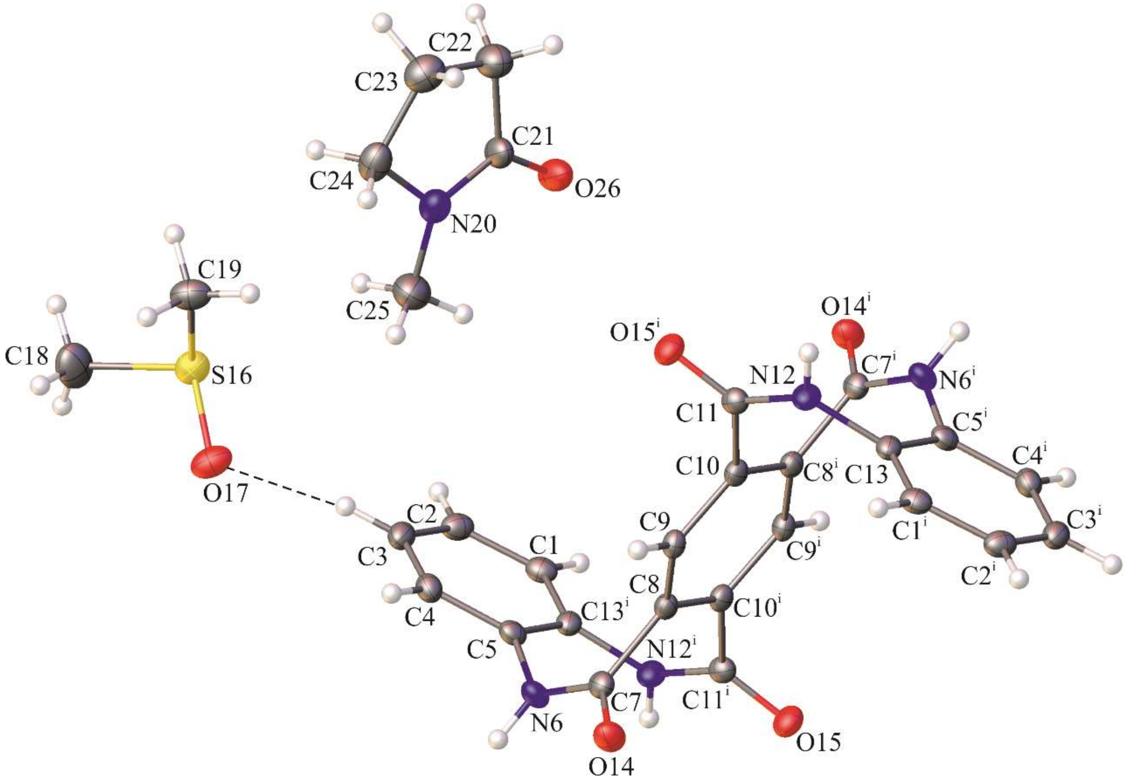

Figure S3. Asymmetric unit of the crystal lattice of **9a** with crystallographic atom numbering. Displacement ellipsoids are drawn at the 50% probability level. The H-atoms are shown as small spheres of arbitrary radius. The intermolecular C−H···O hydrogen bond is represented by a dashed line. Symmetry code: (i) $-x + 2$, $-y + 1$, $-z + 1$.

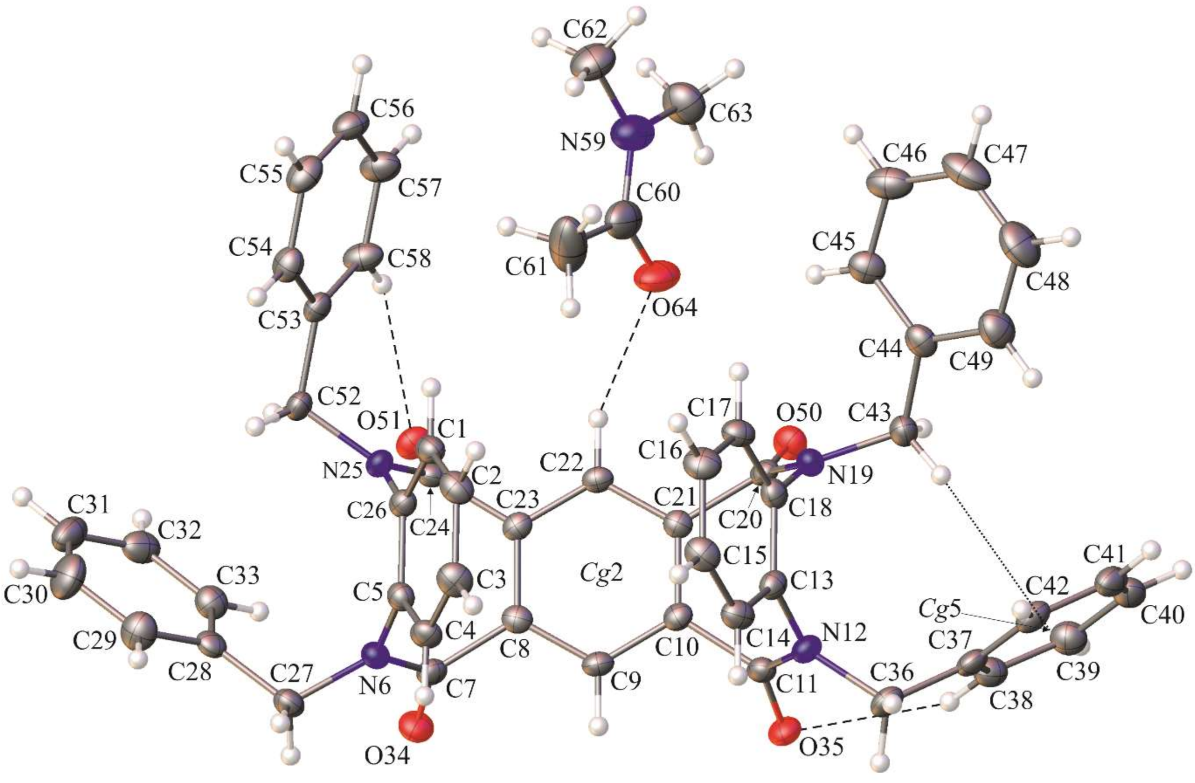

Figure S4. Asymmetric unit of the crystal lattice of **9c** with crystallographic atom numbering. Displacement ellipsoids are drawn at the 50% probability level. The H-atoms are shown as small

spheres of arbitrary radius. The intra- and intermolecular C−H···O hydrogen bonds are represented by a dashed lines, while the intramolecular C−H···π contact by a dotted line. The *Cg*2 and *Cg*5 denote geometric centers of gravity of the aromatic rings defined by the C8−C10/C21−C23 and C37−C42, respectively.

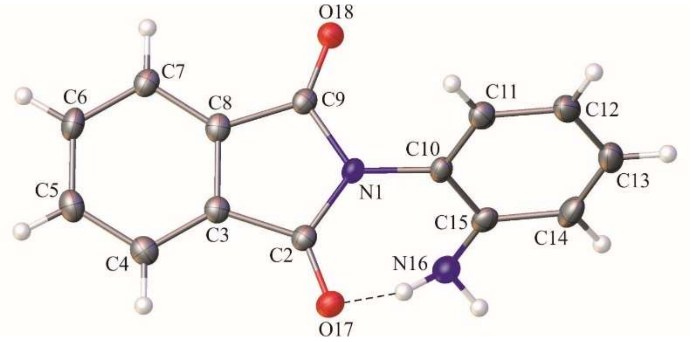

Figure S5. Asymmetric unit of the crystal lattice of **10** with crystallographic atom numbering. Displacement ellipsoids are drawn at the 50% probability level. The H-atoms are shown as small spheres of arbitrary radius. The intramolecular N−H···O hydrogen bond is represented by a dashed line.

Table S1. Crystal data and structure refinement details for investigated compounds.

**Identification code**	**3a**	**3g**	**3h**	**3i**	**3j**	**6**	**9a**	**9c**	**10**
Empirical formula	$C_{14}H_{10}N_2O_2$	$C_{15}H_{10}Cl_2N_2O_2$	$C_{15}H_8Cl_4N_2O_2$	$C_{13}H_{12}N_2O_3S$	$C_{21}H_{16}N_2O_2$	$C_{10}H_{10}N_2O_2$	$C_{36}H_{44}N_6O_8S_2$	$C_{54}H_{47}N_5O_5$	$C_{14}H_{10}N_2O_2$
Formula weight	238.24	321.15	390.03	276.31	328.36	190.20	752.89	845.96	238.24
Temperature /K	100(2)	100(2)	100(2)	100(2)	100(2)	100(2)	100(2)	100(2)	100(2)
Crystal system	monoclinic	monoclinic	monoclinic	monoclinic	monoclinic	triclinic	monoclinic	monoclinic	monoclinic
Space group	$C2/m$	$P2_1/c$	$P2_1/n$	$P2_1$/n	$C2/c$	P–1	$P2_1/n$	$P2_1/c$	$P2_1/n$
a/Å	12.7246(4)	14.2183(6)	8.6515(6)	8.7588(4)	22.8190(11)	4.7890(4)	12.0649(4)	9.6689(3)	5.9096(3)
b/Å	11.8130(3)	8.6661(3)	15.1757(9)	10.0474(5)	9.4527(4)	8.5254(7)	9.4270(3)	26.7240(7)	7.8513(3)
c/Å	7.11026(18)	12.4144(6)	24.6970(12)	13.9960(7)	17.0398(8)	11.4586(10)	16.1785(5)	17.1875(4)	23.4492(11)
α/°	90	90	90	90	90	88.417(7)	90	90	90
β/°	90.822(2)	113.854(5)	99.157(6)	89.820(4)	111.238(6)	87.337(7)	99.636(3)	102.085(3)	93.121(4)
γ/°	90	90	90	90	90	75.762(7)	90	90	90
Volume/Å^3	1068.67(5)	1399.01(12)	3201.2(3)	1231.68(10)	3425.9(3)	452.91(7)	1814.13(10)	4342.7(2)	1086.38(9)
Z	4	4	8	4	8	2	2	4	4
ρ_{calc}g/cm^3	1.481	1.525	1.619	1.490	1.273	1.395	1.378	1.294	1.457
μ/mm^{-1}	0.831	4.228	6.814	2.405	0.666	0.100	1.838	0.670	0.817
F(000)	496.0	656.0	1568.0	576.0	1376.0	200.0	796.0	1784.0	496.0
Crystal size/mm^3	0.16 × 0.07 × 0.07	0.20 × 0.11 × 0.10	0.28 × 0.20 × 0.10	0.15 × 0.10 × 0.05	0.27 × 0.16 × 0.14	0.32 × 0.23 × 0.09	0.45 × 0.10 × 0.08	0.29 × 0.23 × 0.12	0.29 × 0.13 × 0.09
Radiation	Cu$K\alpha$ (λ = 1.54184)	Cu$K\alpha$ (λ = 1.54184)	Cu$K\alpha$ (λ = 1.54184)	Cu$K\alpha$ (λ = 1.54184)	Cu$K\alpha$ (λ = 1.54184)	Mo$K\alpha$ (λ = 0.71073)	Cu$K\alpha$ (λ = 1.54184)	Cu$K\alpha$ (λ = 1.54184)	Cu$K\alpha$ (λ = 1.54184)
2Θ range for data collection/°	10.218 to 134.146	6.798 to 134.068	6.86 to 134.154	10.84 to 134.138	10.24 to 134.152	3.558 to 52.704	8.496 to 134.146	6.212 to 134.152	7.552 to 134.758
Index ranges	$-15 \le h \le 15$, $-14 \le k \le 14$, $-8 \le l \le 8$	$-16 \le h \le 12$, $-9 \le k \le 10$, $-14 \le l \le 14$	$-10 \le h \le 10$, $-18 \le k \le 18$, $-29 \le l \le 29$	$-10 \le h \le 7$, $-12 \le k \le 11$, $-15 \le l \le 16$	$-27 \le h \le 26$, $-10 \le k \le 11$, $-13 \le l \le 20$	$-5 \le h \le 5$, $-10 \le k \le 10$, $-14 \le l \le 14$	$-14 \le h \le 14$, $-11 \le k \le 10$, $-19 \le l \le 13$	$-9 \le h \le 11$, $-31 \le k \le 18$, $-20 \le l \le 20$	$-6 \le h \le 7$, $-9 \le k \le 9$, $-28 \le l \le 28$
Reflections collected	8061	5025	49256	5415	6506	5542	6149	16851	3020
Independent reflections	1014 [R_{int} = 0.0283, R_{sigma} = 0.0126]	2495 [R_{int} = 0.0220, R_{sigma} = 0.0276]	5722 [R_{int} = 0.0622, R_{sigma} = 0.0258]	2191 [R_{int} = 0.0319, R_{sigma} = 0.0344]	3052 [R_{int} = 0.0201, R_{sigma} = 0.0238]	1837 [R_{int} = 0.0365, R_{sigma} = 0.0464]	3239 [R_{int} = 0.0225, R_{sigma} = 0.0288]	7761 [R_{int} = 0.0307, R_{sigma} = 0.0411]	3020 [R_{sigma} = 0.0121]
Data/restraints/parameters	1014/2/85	2495/1/194	5722/62/423	2191/4/182	3052/1/229	1837/1/134	3239/5/244	7761/0/580	3020/2/165
Goodness-of-fit on F^2	1.079	1.066	1.079	1.036	1.060	1.051	1.054	1.048	1.185
Final R indexes [I>=2σ (I)]	R_1 = 0.0323, wR_2 = 0.0794	R_1 = 0.0315, wR_2 = 0.0812	R_1 = 0.1384, wR_2 = 0.3022	R_1 = 0.0353, wR_2 = 0.0864	R_1 = 0.0345, wR_2 = 0.0852	R_1 = 0.0443, wR_2 = 0.0959	R_1 = 0.0387, wR_2 = 0.1025	R_1 = 0.0469, wR_2 = 0.1087	R_1 = 0.0393, wR_2 = 0.1192
Final R indexes [all data]	R_1 = 0.0345, wR_2 = 0.0814	R_1 = 0.0347, wR_2 = 0.0840	R_1 = 0.1489, wR_2 = 0.3092	R_1 = 0.0440, wR_2 = 0.0940	R_1 = 0.0382, wR_2 = 0.0886	R_1 = 0.0557, wR_2 = 0.1047	R_1 = 0.0447, wR_2 = 0.1088	R_1 = 0.0617, wR_2 = 0.1209	R_1 = 0.0424, wR_2 = 0.1207

Largest diff. peak/hole/e $Å^{-3}$	0.20/–0.22	0.29/–0.27	1.30/–0.87	0.25/–0.33	0.18/–0.20	0.24/–0.24	0.36/–0.38	1.17/–0.36	0.39/–0.30

Table S1. S2. Bond lengths for **3a**.

Atom	Atom	Length/Å	Atom	Atom	Length/Å
C(1)	C(2)	1.3861(17)	C(5)	C(5)[1]	1.397(2)
C(1)	C(8)	1.3893(17)	C(5)	C(6)	1.5008(17)
C(2)	C(2)[1]	1.385(3)	C(6)	N(7)	1.3440(16)
C(3)	C(3)[1]	1.392(3)	C(6)	O(9)	1.2366(15)
C(3)	C(4)	1.3829(18)	C(8)	C(8)[1]	1.394(2)
C(4)	C(5)	1.3960(17)	C(8)	N(7)	1.4244(15)

[1]+X,1-Y,+Z.

Table S3. Values of valence angles for **3a**.

Atom	Atom	Atom	Angle/°	Atom	Atom	Atom	Angle/°
C(2)	C(1)	C(8)	120.52(12)	N(7)	C(6)	C(5)	119.23(10)
C(2)[1]	C(2)	C(1)	119.88(7)	O(9)	C(6)	C(5)	119.37(10)
C(4)	C(3)	C(3)[1]	119.76(8)	O(9)	C(6)	N(7)	121.34(11)
C(3)	C(4)	C(5)	120.87(12)	C(1)	C(8)	C(8)[1]	119.59(7)
C(4)	C(5)	C(5)[1]	119.32(7)	C(1)	C(8)	N(7)	118.52(11)
C(4)	C(5)	C(6)	117.00(11)	C(8)[1]	C(8)	N(7)	121.61(6)
C(5)[1]	C(5)	C(6)	123.43(6)	C(6)	N(7)	C(8)	127.28(10)

[1]+X,1-Y,+Z.

Table S4. Values of torsion angles for **3a**.

A	B	C	D	Angle/°	A	B	C	D	Angle/°
C(1)	C(8)	N(7)	C(6)	–118.36(13)	C(4)	C(5)	C(6)	O(9)	–54.44(15)
C(2)	C(1)	C(8)	C(8)[1]	0.26(13)	C(5)[1]	C(5)	C(6)	N(7)	–63.09(12)
C(2)	C(1)	C(8)	N(7)	–173.77(10)	C(5)[1]	C(5)	C(6)	O(9)	119.70(9)
C(3)[1]	C(3)	C(4)	C(5)	–2.44(14)	C(5)	C(6)	N(7)	C(8)	–3.93(18)
C(3)	C(4)	C(5)	C(5)[1]	2.43(14)	C(8)	C(1)	C(2)	C(2)[1]	–0.27(13)
C(3)	C(4)	C(5)	C(6)	176.82(11)	C(8)[1]	C(8)	N(7)	C(6)	67.73(13)
C(4)	C(5)	C(6)	N(7)	122.77(12)	O(9)	C(6)	N(7)	C(8)	173.22(11)

[1]+X,1-Y,+Z.

Table S5. Bond lengths for **3g**.

Atom	Atom	Length/Å	Atom	Atom	Length/Å
C(1)	C(2)	1.385(3)	C(9)	C(10)	1.384(2)
C(1)	C(16)	1.397(2)	C(10)	C(11)	1.396(3)
C(2)	C(3)	1.389(3)	C(10)	Cl(19)	1.7290(17)
C(3)	C(4)	1.381(3)	C(11)	C(12)	1.383(3)
C(4)	C(5)	1.389(2)	C(11)	Cl(20)	1.7271(17)
C(5)	C(16)	1.393(2)	C(12)	C(13)	1.392(2)
C(5)	N(6)	1.433(2)	C(13)	C(14)	1.497(2)
C(7)	C(8)	1.507(2)	C(14)	N(15)	1.352(2)
C(7)	N(6)	1.341(2)	C(14)	O(21)	1.225(2)

C(7)	O(18)	1.236(2)	C(16)	N(15)	1.423(2)
C(8)	C(9)	1.398(2)	C(17)	N(6)	1.474(2)
C(8)	C(13)	1.396(2)			

Table S6. Values of valence angles for **3g**.

Atom	Atom	Atom	Angle/°	Atom	Atom	Atom	Angle/°
C(2)	C(1)	C(16)	120.53(16)	C(10)	C(11)	Cl(20)	121.25(13)
C(1)	C(2)	C(3)	119.95(16)	C(12)	C(11)	C(10)	119.51(16)
C(4)	C(3)	C(2)	119.81(17)	C(12)	C(11)	Cl(20)	119.21(14)
C(3)	C(4)	C(5)	120.58(17)	C(11)	C(12)	C(13)	120.64(16)
C(4)	C(5)	C(16)	119.98(15)	C(8)	C(13)	C(14)	123.26(15)
C(4)	C(5)	N(6)	118.49(15)	C(12)	C(13)	C(8)	119.91(15)
C(16)	C(5)	N(6)	121.19(15)	C(12)	C(13)	C(14)	116.55(15)
N(6)	C(7)	C(8)	119.80(14)	N(15)	C(14)	C(13)	117.99(15)
O(18)	C(7)	C(8)	119.36(14)	O(21)	C(14)	C(13)	120.17(15)
O(18)	C(7)	N(6)	120.81(15)	O(21)	C(14)	N(15)	121.82(16)
C(9)	C(8)	C(7)	117.79(15)	C(1)	C(16)	N(15)	118.79(16)
C(13)	C(8)	C(7)	122.14(15)	C(5)	C(16)	C(1)	119.07(16)
C(13)	C(8)	C(9)	119.36(15)	C(5)	C(16)	N(15)	121.92(15)
C(10)	C(9)	C(8)	120.25(16)	C(5)	N(6)	C(17)	117.49(13)
C(9)	C(10)	C(11)	120.28(16)	C(7)	N(6)	C(5)	124.98(14)
C(9)	C(10)	Cl(19)	119.23(14)	C(7)	N(6)	C(17)	117.53(14)
C(11)	C(10)	Cl(19)	120.48(13)	C(14)	N(15)	C(16)	127.36(14)

Table S7. Values of torsion angles for **3g**.

A	B	C	D	Angle/°	A	B	C	D	Angle/°
C(1)	C(2)	C(3)	C(4)	−1.2(3)	C(9)	C(10)	C(11)	Cl(20)	177.50(14)
C(1)	C(16)	N(15)	C(14)	−113.36(19)	C(10)	C(11)	C(12)	C(13)	−1.4(3)
C(2)	C(1)	C(16)	C(5)	2.6(2)	C(11)	C(12)	C(13)	C(8)	1.6(3)
C(2)	C(1)	C(16)	N(15)	−172.11(16)	C(11)	C(12)	C(13)	C(14)	175.73(15)
C(2)	C(3)	C(4)	C(5)	2.2(3)	C(12)	C(13)	C(14)	N(15)	127.23(17)
C(3)	C(4)	C(5)	C(16)	−0.7(3)	C(12)	C(13)	C(14)	O(21)	−51.4(2)
C(3)	C(4)	C(5)	N(6)	172.76(15)	C(13)	C(8)	C(9)	C(10)	−1.9(2)
C(4)	C(5)	C(16)	C(1)	−1.7(2)	C(13)	C(14)	N(15)	C(16)	−8.0(3)
C(4)	C(5)	C(16)	N(15)	172.85(15)	C(16)	C(1)	C(2)	C(3)	−1.1(3)
C(4)	C(5)	N(6)	C(7)	123.20(18)	C(16)	C(5)	N(6)	C(7)	−63.4(2)
C(4)	C(5)	N(6)	C(17)	−57.4(2)	C(16)	C(5)	N(6)	C(17)	116.00(18)
C(5)	C(16)	N(15)	C(14)	72.1(2)	Cl(19)	C(10)	C(11)	C(12)	−179.31(13)
C(7)	C(8)	C(9)	C(10)	−172.37(15)	Cl(19)	C(10)	C(11)	Cl(20)	−1.4(2)
C(7)	C(8)	C(13)	C(12)	170.10(15)	Cl(20)	C(11)	C(12)	C(13)	−179.40(13)
C(7)	C(8)	C(13)	C(14)	−3.6(2)	N(6)	C(5)	C(16)	C(1)	−174.94(15)
C(8)	C(7)	N(6)	C(5)	−3.2(2)	N(6)	C(5)	C(16)	N(15)	−0.4(2)
C(8)	C(7)	N(6)	C(17)	177.40(15)	N(6)	C(7)	C(8)	C(9)	−118.57(18)
C(8)	C(9)	C(10)	C(11)	2.1(3)	N(6)	C(7)	C(8)	C(13)	71.2(2)
C(8)	C(9)	C(10)	Cl(19)	−179.03(13)	O(18)	C(7)	C(8)	C(9)	59.7(2)
C(8)	C(13)	C(14)	N(15)	−58.9(2)	O(18)	C(7)	C(8)	C(13)	−110.51(18)
C(8)	C(13)	C(14)	O(21)	122.51(18)	O(18)	C(7)	N(6)	C(5)	178.58(15)
C(9)	C(8)	C(13)	C(12)	0.0(2)	O(18)	C(7)	N(6)	C(17)	−0.8(2)

C(9)	C(8)	C(13)	C(14)	−173.67(15)	O(21)	C(14)	N(15)	C(16)	170.64(17)
C(9)	C(10)	C(11)	C(12)	−0.4(3)					

Table S8. Bond lengths for **3h**.

Atom	Atom	Length/Å	Atom	Atom	Length/Å
C(1A)	C(2A)	1.380(13)	C(1B)	C(2B)	1.303(19)
C(1A)	C(16A)	1.392(13)	C(1B)	C(16B)	1.397(16)
C(2A)	C(3A)	1.384(15)	C(2B)	C(3B)	1.344(19)
C(3A)	C(4A)	1.384(16)	C(3B)	C(4B)	1.435(16)
C(4A)	C(5A)	1.394(15)	C(4B)	C(5B)	1.394(17)
C(5A)	C(16A)	1.392(13)	C(5B)	C(16B)	1.386(18)
C(5A)	N(6A)	1.423(13)	C(5B)	N(6B)	1.440(13)
C(7A)	C(8A)	1.508(15)	C(7B)	C(8B)	1.508(14)
C(7A)	N(6A)	1.349(14)	C(7B)	N(6B)	1.338(13)
C(7A)	O(18A)	1.232(13)	C(7B)	O(18B)	1.217(12)
C(8A)	C(9A)	1.386(13)	C(8B)	C(9B)	1.406(14)
C(8A)	C(13A)	1.393(14)	C(8B)	C(13B)	1.380(15)
C(9A)	C(10A)	1.396(15)	C(9B)	C(10B)	1.383(14)
C(9A)	Cl(3)	1.724(11)	C(9B)	Cl(19)	1.718(10)
C(10A)	C(11A)	1.395(15)	C(10B)	C(11B)	1.385(14)
C(10A)	Cl(4)	1.711(9)	C(10B)	Cl(20)	1.716(10)
C(11A)	C(12A)	1.369(13)	C(11B)	C(12B)	1.366(15)
C(11A)	Cl(2)	1.727(10)	C(11B)	Cl(21)	1.730(10)
C(12A)	C(13A)	1.415(15)	C(12B)	C(13B)	1.401(14)
C(12A)	Cl(1)	1.722(11)	C(12B)	Cl(22)	1.735(12)
C(13A)	C(14A)	1.503(12)	C(13B)	C(14B)	1.511(16)
C(14A)	N(15A)	1.339(12)	C(14B)	N(15B)	1.336(15)
C(14A)	O(23A)	1.235(12)	C(14B)	O(23B)	1.236(16)
C(16A)	N(15A)	1.441(11)	C(16B)	N(15B)	1.400(18)
C(17A)	N(6A)	1.480(13)	C(17B)	N(6B)	1.444(17)

Table S9. Values of valence angles for **3h**.

Atom	Atom	Atom	Angle/°	Atom	Atom	Atom	Angle/°
C(2A)	C(1A)	C(16A)	118.5(9)	C(2B)	C(1B)	C(16B)	119.1(15)
C(1A)	C(2A)	C(3A)	121.0(10)	C(1B)	C(2B)	C(3B)	122.9(14)
C(4A)	C(3A)	C(2A)	120.1(10)	C(2B)	C(3B)	C(4B)	120.3(13)
C(3A)	C(4A)	C(5A)	120.3(10)	C(5B)	C(4B)	C(3B)	117.6(13)
C(4A)	C(5A)	N(6A)	120.6(10)	C(4B)	C(5B)	N(6B)	121.7(13)
C(16A)	C(5A)	C(4A)	118.6(10)	C(16B)	C(5B)	C(4B)	118.2(11)
C(16A)	C(5A)	N(6A)	120.8(9)	C(16B)	C(5B)	N(6B)	120.0(12)
N(6A)	C(7A)	C(8A)	116.4(9)	N(6B)	C(7B)	C(8B)	115.1(8)
O(18A)	C(7A)	C(8A)	120.1(10)	O(18B)	C(7B)	C(8B)	119.4(9)
O(18A)	C(7A)	N(6A)	123.5(11)	O(18B)	C(7B)	N(6B)	125.5(10)
C(9A)	C(8A)	C(7A)	118.3(10)	C(9B)	C(8B)	C(7B)	121.4(10)
C(9A)	C(8A)	C(13A)	119.9(10)	C(13B)	C(8B)	C(7B)	120.1(10)
C(13A)	C(8A)	C(7A)	121.7(9)	C(13B)	C(8B)	C(9B)	118.4(9)
C(8A)	C(9A)	C(10A)	118.9(10)	C(8B)	C(9B)	Cl(19)	118.4(8)
C(8A)	C(9A)	Cl(3)	121.0(9)	C(10B)	C(9B)	C(8B)	120.7(9)

C(10A)	C(9A)	Cl(3)	120.1(7)	C(10B)	C(9B)	Cl(19)	120.9(8)
C(9A)	C(10A)	Cl(4)	118.5(8)	C(9B)	C(10B)	C(11B)	119.9(9)
C(11A)	C(10A)	C(9A)	121.1(8)	C(9B)	C(10B)	Cl(20)	120.3(8)
C(11A)	C(10A)	Cl(4)	120.4(8)	C(11B)	C(10B)	Cl(20)	119.8(7)
C(10A)	C(11A)	Cl(2)	119.8(7)	C(10B)	C(11B)	Cl(21)	119.9(8)
C(12A)	C(11A)	C(10A)	120.7(10)	C(12B)	C(11B)	C(10B)	120.2(9)
C(12A)	C(11A)	Cl(2)	119.6(9)	C(12B)	C(11B)	Cl(21)	119.9(9)
C(11A)	C(12A)	C(13A)	118.4(9)	C(11B)	C(12B)	C(13B)	120.2(11)
C(11A)	C(12A)	Cl(1)	121.3(9)	C(11B)	C(12B)	Cl(22)	118.8(8)
C(13A)	C(12A)	Cl(1)	120.3(7)	C(13B)	C(12B)	Cl(22)	120.9(9)
C(8A)	C(13A)	C(12A)	121.1(8)	C(8B)	C(13B)	C(12B)	120.6(10)
C(8A)	C(13A)	C(14A)	118.9(10)	C(8B)	C(13B)	C(14B)	120.4(10)
C(12A)	C(13A)	C(14A)	120.1(9)	C(12B)	C(13B)	C(14B)	118.9(11)
N(15A)	C(14A)	C(13A)	116.6(8)	N(15B)	C(14B)	C(13B)	115.8(13)
O(23A)	C(14A)	C(13A)	119.6(9)	O(23B)	C(14B)	C(13B)	119.9(10)
O(23A)	C(14A)	N(15A)	123.8(9)	O(23B)	C(14B)	N(15B)	124.3(12)
C(1A)	C(16A)	C(5A)	121.6(9)	C(1B)	C(16B)	N(15B)	115.3(13)
C(1A)	C(16A)	N(15A)	118.4(8)	C(5B)	C(16B)	C(1B)	121.8(14)
C(5A)	C(16A)	N(15A)	120.1(9)	C(5B)	C(16B)	N(15B)	122.9(11)
C(5A)	N(6A)	C(17A)	117.8(9)	C(5B)	N(6B)	C(17B)	115.3(9)
C(7A)	N(6A)	C(5A)	123.1(9)	C(7B)	N(6B)	C(5B)	123.5(9)
C(7A)	N(6A)	C(17A)	119.2(10)	C(7B)	N(6B)	C(17B)	118.7(10)
C(14A)	N(15A)	C(16A)	124.1(7)	C(14B)	N(15B)	C(16B)	126.0(12)

Table S10. Values of torsion angles for **3h**.

A	B	C	D	Angle/°	A	B	C	D	Angle/°
C(1A).	C(2A)	C(3A)	C(4A)	–1.4(14)	C(1B)	C(2B)	C(3B)	C(4B)	0(2)
C(1A)	C(16A)	N(15A)	C(14A)	–108.4(11)	C(1B)	C(16B)	N(15B)	C(14B)	109.0(15)
C(2A)	C(1A)	C(16A)	C(5A)	1.2(12)	C(2B)	C(1B)	C(16B)	C(5B)	–1.3(19)
C(2A)	C(1A)	C(16A)	N(15A)	–179.3(7)	C(2B)	C(1B)	C(16B)	N(15B)	179.0(12)
C(2A)	C(3A)	C(4A)	C(5A)	–0.1(14)	C(2B)	C(3B)	C(4B)	C(5B)	–1.3(17)
C(3A)	C(4A)	C(5A)	C(16A)	2.1(14)	C(3B)	C(4B)	C(5B)	C(16B)	1.6(16)
C(3A)	C(4A)	C(5A)	N(6A)	–178.3(8)	C(3B)	C(4B)	C(5B)	N(6B)	–174.9(9)
C(4A)	C(5A)	C(16A)	C(1A)	–2.6(13)	C(4B)	C(5B)	C(16B)	C(1B)	–0.3(17)
C(4A)	C(5A)	C(16A)	N(15A)	177.8(8)	C(4B)	C(5B)	C(16B)	N(15B)	179.3(10)
C(4A)	C(5A)	N(6A)	C(7A)	113.2(12)	C(4B)	C(5B)	N(6B)	C(7B)	–123.1(13)
C(4A)	C(5A)	N(6A)	C(17A)	–67.0(13)	C(4B)	C(5B)	N(6B)	C(17B)	75.5(17)
C(5A)	C(16A)	N(15A)	C(14A)	71.2(12)	C(5B)	C(16B)	N(15B)	C(14B)	–70.6(19)
C(7A)	C(8A)	C(9A)	C(10A)	–177.8(9)	C(7B)	C(8B)	C(9B)	C(10B)	173.2(8)
C(7A)	C(8A)	C(9A)	Cl(3)	5.1(13)	C(7B)	C(8B)	C(9B)	Cl(19)	–7.2(12)
C(7A)	C(8A)	C(13A)	C(12A)	177.7(9)	C(7B)	C(8B)	C(13B)	C(12B)	–175.4(9)
C(7A)	C(8A)	C(13A)	C(14A)	–3.2(14)	C(7B)	C(8B)	C(13B)	C(14B)	1.6(13)
C(8A)	C(7A)	N(6A)	C(5A)	–0.9(15)	C(8B)	C(7B)	N(6B)	C(5B)	15.2(18)
C(8A)	C(7A)	N(6A)	C(17A)	179.3(10)	C(8B)	C(7B)	N(6B)	C(17B)	176.1(13)
C(8A)	C(9A)	C(10A)	C(11A)	1.4(15)	C(8B)	C(9B)	C(10B)	C(11B)	2.0(13)
C(8A)	C(9A)	C(10A)	Cl(4)	–178.4(8)	C(8B)	C(9B)	C(10B)	Cl(20)	–179.2(7)
C(8A)	C(13A)	C(14A)	N(15A)	–68.7(12)	C(8B)	C(13B)	C(14B)	N(15B)	64.5(14)
C(8A)	C(13A)	C(14A)	O(23A)	110.2(12)	C(8B)	C(13B)	C(14B)	O(23B)	–114.7(14)

C(9A)	C(8A)	C(13A)	C(12A)	2.5(14)	C(9B)	C(8B)	C(13B)	C(12B)	−0.2(13)
C(9A)	C(8A)	C(13A)	C(14A)	−178.4(9)	C(9B)	C(8B)	C(13B)	C(14B)	176.8(9)
C(9A)	C(10A)	C(11A)	C(12A)	−0.4(15)	C(9B)	C(10B)	C(11B)	C(12B)	0.2(13)
C(9A)	C(10A)	C(11A)	Cl(2)	−179.5(8)	C(9B)	C(10B)	C(11B)	Cl(21)	177.5(7)
C(10A)	C(11A)	C(12A)	C(13A)	0.4(14)	C(10B)	C(11B)	C(12B)	C(13B)	−2.4(14)
C(10A)	C(11A)	C(12A)	Cl(1)	−178.3(7)	C(10B)	C(11B)	C(12B)	Cl(22)	173.9(7)
C(11A)	C(12A)	C(13A)	C(8A)	−1.4(13)	C(11B)	C(12B)	C(13B)	C(8B)	2.4(14)
C(11A)	C(12A)	C(13A)	C(14A)	179.5(8)	C(11B)	C(12B)	C(13B)	C(14B)	−174.7(9)
C(12A)	C(13A)	C(14A)	N(15A)	110.4(11)	C(12B)	C(13B)	C(14B)	N(15B)	−118.5(13)
C(12A)	C(13A)	C(14A)	O(23A)	−70.7(14)	C(12B)	C(13B)	C(14B)	O(23B)	62.3(14)
C(13A)	C(8A)	C(9A)	C(10A)	−2.4(14)	C(13B)	C(8B)	C(9B)	C(10B)	−1.9(13)
C(13A)	C(8A)	C(9A)	Cl(3)	−179.5(7)	C(13B)	C(8B)	C(9B)	Cl(19)	177.7(7)
C(13A)	C(14A)	N(15A)	C(16A)	1.2(15)	C(13B)	C(14B)	N(15B)	C(16B)	6(2)
C(16A)	C(1A)	C(2A)	C(3A)	0.9(12)	C(16B)	C(1B)	C(2B)	C(3B)	2(2)
C(16A)	C(5A)	N(6A)	C(7A)	−67.2(13)	C(16B)	C(5B)	N(6B)	C(7B)	60.5(17)
C(16A)	C(5A)	N(6A)	C(17A)	112.6(11)	C(16B)	C(5B)	N(6B)	C(17B)	−100.9(15)
Cl(1)	C(12A)	C(13A)	C(8A)	177.3(7)	Cl(19)	C(9B)	C(10B)	C(11B)	−177.7(7)
Cl(1)	C(12A)	C(13A)	C(14A)	−1.8(12)	Cl(19)	C(9B)	C(10B)	Cl(20)	1.1(11)
Cl(2)	C(11A)	C(12A)	C(13A)	179.5(7)	Cl(20)	C(10B)	C(11B)	C(12B)	−178.6(7)
Cl(2)	C(11A)	C(12A)	Cl(1)	0.8(11)	Cl(20)	C(10B)	C(11B)	Cl(21)	−1.3(10)
Cl(3)	C(9A)	C(10A)	C(11A)	178.6(8)	Cl(21)	C(11B)	C(12B)	C(13B)	−179.7(7)
Cl(3)	C(9A)	C(10A)	Cl(4)	−1.2(12)	Cl(21)	C(11B)	C(12B)	Cl(22)	−3.4(11)
Cl(4)	C(10A)	C(11A)	C(12A)	179.4(7)	Cl(22)	C(12B)	C(13B)	C(8B)	−173.8(7)
Cl(4)	C(10A)	C(11A)	Cl(2)	0.2(12)	Cl(22)	C(12B)	C(13B)	C(14B)	9.1(13)
N(6A)	C(5A)	C(16A)	C(1A)	177.7(8)	N(6B)	C(5B)	C(16B)	C(1B)	176.2(10)
N(6A)	C(5A)	C(16A)	N(15A)	−1.8(12)	N(6B)	C(5B)	C(16B)	N(15B)	−4.2(16)
N(6A)	C(7A)	C(8A)	C(9A)	−111.2(11)	N(6B)	C(7B)	C(8B)	C(9B)	104.1(12)
N(6A)	C(7A)	C(8A)	C(13A)	73.5(13)	N(6B)	C(7B)	C(8B)	C(13B)	−80.9(13)
O(18A)	C(7A)	C(8A)	C(9A)	70.3(15)	O(18B)	C(7B)	C(8B)	C(9B)	−75.0(15)
O(18A)	C(7A)	C(8A)	C(13A)	−105.0(13)	O(18B)	C(7B)	C(8B)	C(13B)	100.0(12)
O(18A)	C(7A)	N(6A)	C(5A)	177.5(11)	O(18B)	C(7B)	N(6B)	C(5B)	−165.7(13)
O(18A)	C(7A)	N(6A)	C(17A)	−2.3(18)	O(18B)	C(7B)	N(6B)	C(17B)	−5(2)
O(23A)	C(14A)	N(15A)	C(16A)	−177.7(10)	O(23B)	C(14B)	N(15B)	C(16B)	−174.5(13)

Table S11. Bond lengths for **3i**.

Atom	Atom	Length/Å	Atom	Atom	Length/Å
C(1)	C(2)	1.389(3)	C(8)	C(9)	1.364(3)
C(1)	C(15)	1.395(3)	C(8)	C(12)	1.432(3)
C(2)	C(3)	1.390(3)	C(9)	S(10)	1.711(2)
C(3)	C(4)	1.385(3)	C(11)	C(12)	1.367(3)
C(4)	C(5)	1.388(3)	C(11)	S(10)	1.711(2)
C(5)	C(15)	1.396(3)	C(12)	C(13)	1.500(3)
C(5)	N(6)	1.438(2)	C(13)	N(14)	1.350(2)
C(7)	C(8)	1.490(3)	C(13)	O(18)	1.228(2)
C(7)	N(6)	1.354(2)	C(15)	N(14)	1.421(2)
C(7)	O(17)	1.228(2)	C(16)	N(6)	1.464(2)

Table S12. Values of valence angles for **3i**.

Atom	Atom	Atom	Angle/˚	Atom	Atom	Atom	Angle/˚
C(2)	C(1)	C(15)	119.96(18)	C(8)	C(12)	C(13)	126.51(17)
C(1)	C(2)	C(3)	120.46(18)	C(11)	C(12)	C(8)	111.81(17)
C(4)	C(3)	C(2)	119.45(18)	C(11)	C(12)	C(13)	121.42(17)
C(3)	C(4)	C(5)	120.66(18)	N(14)	C(13)	C(12)	118.11(16)
C(4)	C(5)	C(15)	119.86(17)	O(18)	C(13)	C(12)	119.87(17)
C(4)	C(5)	N(6)	118.53(17)	O(18)	C(13)	N(14)	121.91(18)
C(15)	C(5)	N(6)	121.30(16)	C(1)	C(15)	C(5)	119.53(17)
N(6)	C(7)	C(8)	115.64(16)	C(1)	C(15)	N(14)	119.28(17)
O(17)	C(7)	C(8)	121.82(17)	C(5)	C(15)	N(14)	121.14(17)
O(17)	C(7)	N(6)	122.47(18)	C(5)	N(6)	C(16)	115.53(15)
C(9)	C(8)	C(7)	123.54(17)	C(7)	N(6)	C(5)	122.86(16)
C(9)	C(8)	C(12)	112.48(17)	C(7)	N(6)	C(16)	118.69(16)
C(12)	C(8)	C(7)	123.77(17)	C(13)	N(14)	C(15)	125.11(16)
C(8)	C(9)	S(10)	111.71(15)	C(9)	S(10)	C(11)	92.01(10)
C(12)	C(11)	S(10)	111.95(15)				

Table S13. Values of torsion angles for **3i**.

A	B	C	D	Angle/˚	A	B	C	D	Angle/˚
C(1)	C(2)	C(3)	C(4)	1.8(3)	C(9)	C(8)	C(12)	C(13)	−173.69(17)
C(1)	C(15)	N(14)	C(13)	−124.8(2)	C(11)	C(12)	C(13)	N(14)	116.5(2)
C(2)	C(1)	C(15)	C(5)	−1.2(3)	C(11)	C(12)	C(13)	O(18)	−59.9(3)
C(2)	C(1)	C(15)	N(14)	−178.64(17)	C(12)	C(8)	C(9)	S(10)	−1.6(2)
C(2)	C(3)	C(4)	C(5)	0.1(3)	C(12)	C(11)	S(10)	C(9)	−1.52(15)
C(3)	C(4)	C(5)	C(15)	−2.5(3)	C(12)	C(13)	N(14)	C(15)	4.8(3)
C(3)	C(4)	C(5)	N(6)	171.29(17)	C(15)	C(1)	C(2)	C(3)	−1.2(3)
C(4)	C(5)	C(15)	C(1)	3.0(3)	C(15)	C(5)	N(6)	C(7)	−89.2(2)
C(4)	C(5)	C(15)	N(14)	−179.60(17)	C(15)	C(5)	N(6)	C(16)	110.39(19)
C(4)	C(5)	N(6)	C(7)	97.1(2)	N(6)	C(5)	C(15)	C(1)	−170.55(17)
C(4)	C(5)	N(6)	C(16)	−63.3(2)	N(6)	C(5)	C(15)	N(14)	6.8(3)
C(5)	C(15)	N(14)	C(13)	57.8(3)	N(6)	C(7)	C(8)	C(9)	−135.68(19)
C(7)	C(8)	C(9)	S(10)	−176.62(14)	N(6)	C(7)	C(8)	C(12)	49.9(2)
C(7)	C(8)	C(12)	C(11)	175.47(17)	O(17)	C(7)	C(8)	C(9)	47.2(3)
C(7)	C(8)	C(12)	C(13)	1.3(3)	O(17)	C(7)	C(8)	C(12)	−127.2(2)
C(8)	C(7)	N(6)	C(5)	24.5(2)	O(17)	C(7)	N(6)	C(5)	−158.49(18)
C(8)	C(7)	N(6)	C(16)	−175.74(16)	O(17)	C(7)	N(6)	C(16)	1.3(3)
C(8)	C(9)	S(10)	C(11)	1.79(15)	O(18)	C(13)	N(14)	C(15)	−178.96(17)
C(8)	C(12)	C(13)	N(14)	−69.9(2)	S(10)	C(11)	C(12)	C(8)	0.9(2)
C(8)	C(12)	C(13)	O(18)	113.8(2)	S(10)	C(11)	C(12)	C(13)	175.38(14)
C(9)	C(8)	C(12)	C(11)	0.5(2)					

Table S14. Bond lengths for **3j**.

Atom	Atom	Length/Å	Atom	Atom	Length/Å
C(1)	C(2)	1.3857(18)	C(10)	C(11)	1.387(2)
C(1)	C(16)	1.3909(17)	C(11)	C(12)	1.3849(19)
C(2)	C(3)	1.3902(18)	C(12)	C(13)	1.3950(18)
C(3)	C(4)	1.3841(17)	C(13)	C(14)	1.5035(16)
C(4)	C(5)	1.3929(17)	C(14)	N(15)	1.3396(16)

C(16)	C(5)	1.3947(16)	C(14)	O(25)	1.2394(15)
C(16)	N(15)	1.4238(15)	C(17)	C(18)	1.5149(17)
C(5)	N(6)	1.4374(14)	C(17)	N(6)	1.4707(15)
C(7)	C(8)	1.4980(17)	C(18)	C(19)	1.3961(18)
C(7)	N(6)	1.3572(15)	C(18)	C(23)	1.3892(17)
C(7)	O(24)	1.2306(14)	C(19)	C(20)	1.384(2)
C(8)	C(9)	1.3972(17)	C(20)	C(21)	1.385(2)
C(8)	C(13)	1.3967(17)	C(21)	C(22)	1.3833(19)
C(9)	C(10)	1.380(2)	C(22)	C(23)	1.3894(18)

Table S15. Values of valence angles for **3j**.

Atom	Atom	Atom	Angle/°	Atom	Atom	Atom	Angle/°
C(2)	C(1)	C(16)	120.10(11)	C(8)	C(13)	C(14)	123.15(11)
C(1)	C(2)	C(3)	120.27(11)	C(12)	C(13)	C(8)	119.67(11)
C(4)	C(3)	C(2)	119.69(11)	C(12)	C(13)	C(14)	116.80(11)
C(3)	C(4)	C(5)	120.50(11)	N(15)	C(14)	C(13)	119.21(10)
C(1)	C(16)	C(5)	119.83(11)	O(25)	C(14)	C(13)	118.79(11)
C(1)	C(16)	N(15)	119.65(10)	O(25)	C(14)	N(15)	121.95(11)
C(5)	C(16)	N(15)	120.47(10)	N(6)	C(17)	C(18)	114.64(10)
C(4)	C(5)	C(16)	119.58(11)	C(19)	C(18)	C(17)	117.91(11)
C(4)	C(5)	N(6)	119.23(10)	C(23)	C(18)	C(17)	123.63(11)
C(16)	C(5)	N(6)	121.10(10)	C(23)	C(18)	C(19)	118.42(11)
N(6)	C(7)	C(8)	118.61(10)	C(20)	C(19)	C(18)	120.66(13)
O(24)	C(7)	C(8)	119.47(11)	C(19)	C(20)	C(21)	120.34(13)
O(24)	C(7)	N(6)	121.89(11)	C(22)	C(21)	C(20)	119.65(12)
C(9)	C(8)	C(7)	118.11(11)	C(21)	C(22)	C(23)	120.01(12)
C(13)	C(8)	C(7)	122.01(10)	C(18)	C(23)	C(22)	120.91(12)
C(13)	C(8)	C(9)	119.13(11)	C(5)	N(6)	C(17)	118.11(9)
C(10)	C(9)	C(8)	120.74(12)	C(7)	N(6)	C(5)	122.43(10)
C(9)	C(10)	C(11)	120.10(12)	C(7)	N(6)	C(17)	118.12(10)
C(12)	C(11)	C(10)	119.80(12)	C(14)	N(15)	C(16)	125.33(10)
C(11)	C(12)	C(13)	120.51(12)				

Table S16. Values of torsion angles for **3j**.

A	B	C	D	Angle/°	A	B	C	D	Angle/°
C(1)	C(2)	C(3)	C(4)	−0.71(18)	C(11)	C(12)	C(13)	C(8)	−1.79(19)
C(1)	C(16)	C(5)	C(4)	−1.60(17)	C(11)	C(12)	C(13)	C(14)	−174.92(12)
C(1)	C(16)	C(5)	N(6)	174.97(10)	C(12)	C(13)	C(14)	N(15)	−117.66(13)
C(1)	C(16)	N(15)	C(14)	121.27(13)	C(12)	C(13)	C(14)	O(25)	60.06(15)
C(2)	C(1)	C(16)	C(5)	0.98(17)	C(13)	C(8)	C(9)	C(10)	1.75(18)
C(2)	C(1)	C(16)	N(15)	178.25(10)	C(13)	C(14)	N(15)	C(16)	−6.60(17)
C(2)	C(3)	C(4)	C(5)	0.07(17)	C(17)	C(18)	C(19)	C(20)	−177.13(12)
C(3)	C(4)	C(5)	C(16)	1.09(17)	C(17)	C(18)	C(23)	C(22)	176.71(11)
C(3)	C(4)	C(5)	N(6)	−175.55(10)	C(18)	C(17)	N(6)	C(5)	104.13(12)
C(4)	C(5)	N(6)	C(7)	−103.40(13)	C(18)	C(17)	N(6)	C(7)	−88.74(13)
C(4)	C(5)	N(6)	C(17)	63.15(14)	C(18)	C(19)	C(20)	C(21)	0.3(2)
C(16)	C(1)	C(2)	C(3)	0.18(18)	C(19)	C(18)	C(23)	C(22)	−0.88(18)
C(16)	C(5)	N(6)	C(7)	80.02(14)	C(19)	C(20)	C(21)	C(22)	−0.9(2)

C(16)	C(5)	N(6)	C(17)	−113.44(12)	C(20)	C(21)	C(22)	C(23)	0.6(2)
C(5)	C(16)	N(15)	C(14)	−61.47(16)	C(21)	C(22)	C(23)	C(18)	0.26(19)
C(7)	C(8)	C(9)	C(10)	172.03(12)	C(23)	C(18)	C(19)	C(20)	0.60(19)
C(7)	C(8)	C(13)	C(12)	−169.67(11)	N(6)	C(7)	C(8)	C(9)	131.22(12)
C(7)	C(8)	C(13)	C(14)	3.01(17)	N(6)	C(7)	C(8)	C(13)	−58.80(15)
C(8)	C(7)	N(6)	C(5)	−16.05(16)	N(6)	C(17)	C(18)	C(19)	−169.64(11)
C(8)	C(7)	N(6)	C(17)	177.41(10)	N(6)	C(17)	C(18)	C(23)	12.76(16)
C(8)	C(9)	C(10)	C(11)	−2.2(2)	N(15)	C(16)	C(5)	C(4)	−178.86(10)
C(8)	C(13)	C(14)	N(15)	69.47(15)	N(15)	C(16)	C(5)	N(6)	−2.28(16)
C(8)	C(13)	C(14)	O(25)	−112.81(13)	O(24)	C(7)	C(8)	C(9)	−50.62(16)
C(9)	C(8)	C(13)	C(12)	0.22(17)	O(24)	C(7)	C(8)	C(13)	119.36(13)
C(9)	C(8)	C(13)	C(14)	172.89(11)	O(24)	C(7)	N(6)	C(5)	165.84(11)
C(9)	C(10)	C(11)	C(12)	0.6(2)	O(24)	C(7)	N(6)	C(17)	−0.70(16)
C(10)	C(11)	C(12)	C(13)	1.4(2)	O(25)	C(14)	N(15)	C(16)	175.75(11)

Table S17. Bond lengths for **6**.

Atom	Atom	Length/Å	Atom	Atom	Length/Å
C(1)	C(2)	1.381(2)	C(7)	N(6)	1.349(2)
C(1)	C(12)	1.391(2)	C(7)	O(13)	1.2389(19)
C(2)	C(3)	1.391(3)	C(8)	C(9)	1.531(2)
C(3)	C(4)	1.381(2)	C(9)	C(10)	1.508(2)
C(4)	C(5)	1.395(2)	C(10)	N(11)	1.343(2)
C(5)	C(12)	1.402(2)	C(10)	O(14)	1.2370(19)
C(5)	N(6)	1.432(2)	C(12)	N(11)	1.424(2)
C(7)	C(8)	1.517(2)			

Table S18. Values of valence angles for **6**.

Atom	Atom	Atom	Angle/°	Atom	Atom	Atom	Angle/°
C(2)	C(1)	C(12)	120.60(17)	C(7)	C(8)	C(9)	120.12(14)
C(1)	C(2)	C(3)	119.71(17)	C(10)	C(9)	C(8)	111.20(13)
C(4)	C(3)	C(2)	120.21(17)	N(11)	C(10)	C(9)	116.60(15)
C(3)	C(4)	C(5)	120.60(16)	O(14)	C(10)	C(9)	121.33(15)
C(4)	C(5)	C(12)	119.07(15)	O(14)	C(10)	N(11)	122.07(15)
C(4)	C(5)	N(6)	118.27(15)	C(1)	C(12)	C(5)	119.78(15)
C(12)	C(5)	N(6)	122.47(15)	C(1)	C(12)	N(11)	119.63(15)
N(6)	C(7)	C(8)	123.36(14)	C(5)	C(12)	N(11)	120.58(15)
O(13)	C(7)	C(8)	117.01(14)	C(7)	N(6)	C(5)	131.48(14)
O(13)	C(7)	N(6)	119.62(15)	C(10)	N(11)	C(12)	123.78(14)

Table S19. Values of torsion angles for **6**.

A	B	C	D	Angle/°	A	B	C	D	Angle/°
C(1)	C(2)	C(3)	C(4)	0.1(3)	C(8)	C(7)	N(6)	C(5)	−6.4(3)
C(1)	C(12)	N(11)	C(10)	114.74(18)	C(8)	C(9)	C(10)	N(11)	94.12(17)
C(2)	C(1)	C(12)	C(5)	−1.3(2)	C(8)	C(9)	C(10)	O(14)	−84.92(19)
C(2)	C(1)	C(12)	N(11)	179.52(15)	C(9)	C(10)	N(11)	C(12)	−0.4(2)
C(2)	C(3)	C(4)	C(5)	0.0(3)	C(12)	C(1)	C(2)	C(3)	0.6(3)
C(3)	C(4)	C(5)	C(12)	−0.7(2)	C(12)	C(5)	N(6)	C(7)	68.6(2)
C(3)	C(4)	C(5)	N(6)	−175.77(15)	N(6)	C(5)	C(12)	C(1)	176.20(15)

C(4)	C(5)	C(12)	C(1)	1.3(2)	N(6)	C(5)	C(12)	N(11)	−4.6(2)
C(4)	C(5)	C(12)	N(11)	−179.49(14)	N(6)	C(7)	C(8)	C(9)	−28.2(2)
C(4)	C(5)	N(6)	C(7)	−116.46(19)	O(13)	C(7)	C(8)	C(9)	153.35(15)
C(5)	C(12)	N(11)	C(10)	−64.4(2)	O(13)	C(7)	N(6)	C(5)	171.99(16)
C(7)	C(8)	C(9)	C(10)	−47.3(2)	O(14)	C(10)	N(11)	C(12)	178.65(14)

Table S20. Bond lengths for **9a**.

Atom	Atom	Length/Å	Atom	Atom	Length/Å
C(1)	C(2)	1.387(3)	C(11)	C(10)[1]	1.504(2)
C(1)	C(13)	1.391(3)	C(11)	N(12)	1.338(2)
C(2)	C(3)	1.387(3)	C(11)	O(15)	1.234(2)
C(3)	C(4)	1.383(3)	C(13)	N(12)	1.426(2)
C(4)	C(5)	1.395(2)	C(21)	C(22)	1.522(3)
C(5)	C(13)	1.395(2)	C(21)	N(20)	1.330(2)
C(5)	N(6)	1.428(2)	C(21)	O(26)	1.233(2)
C(7)	C(8)	1.504(2)	C(22)	C(23)	1.510(3)
C(7)	N(6)	1.347(2)	C(23)	C(24)	1.529(3)
C(7)	O(14)	1.227(2)	C(24)	N(20)	1.452(3)
C(8)	C(9)	1.391(2)	C(25)	N(20)	1.440(3)
C(8)	C(10)[1]	1.398(2)	C(18)	S(16)	1.779(2)
C(9)	C(10)	1.395(2)	C(19)	S(16)	1.784(2)
C(10)	C(8)[1]	1.398(2)	O(17)	S(16)	1.5095(14)
C(10)	C(11)[1]	1.504(2)			

[1]2-X,1-Y,1-Z.

Table S21. Values of valence angles for **9a**.

Atom	Atom	Atom	Angle/°	Atom	Atom	Atom	Angle/°
C(2)	C(1)	C(13)	119.96(17)	O(15)	C(11)	N(12)	122.63(16)
C(3)	C(2)	C(1)	120.03(17)	C(1)	C(13)	C(5)	120.27(16)
C(4)	C(3)	C(2)	120.11(17)	C(1)	C(13)	N(12)	118.66(15)
C(3)	C(4)	C(5)	120.49(16)	C(5)	C(13)	N(12)	120.84(16)
C(4)	C(5)	N(6)	119.25(15)	C(7)	N(6)	C(5)	127.23(14)
C(13)	C(5)	C(4)	119.13(16)	C(11)	N(12)	C(13)	126.15(14)
C(13)	C(5)	N(6)	121.35(15)	N(20)	C(21)	C(22)	108.19(17)
N(6)	C(7)	C(8)	117.45(15)	O(26)	C(21)	C(22)	126.07(17)
O(14)	C(7)	C(8)	120.25(15)	O(26)	C(21)	N(20)	125.65(19)
O(14)	C(7)	N(6)	122.29(16)	C(23)	C(22)	C(21)	105.09(17)
C(9)	C(8)	C(7)	118.29(15)	C(22)	C(23)	C(24)	105.54(17)
C(9)	C(8)	C(10)[1]	119.92(16)	N(20)	C(24)	C(23)	103.62(16)
C(10)[1]	C(8)	C(7)	121.68(15)	C(21)	N(20)	C(24)	114.75(17)
C(8)	C(9)	C(10)	120.08(16)	C(21)	N(20)	C(25)	121.48(18)
C(8)[1]	C(10)	C(11)[1]	121.41(15)	C(25)	N(20)	C(24)	121.75(17)
C(9)	C(10)	C(8)[1]	120.00(16)	C(18)	S(16)	C(19)	98.47(11)
C(9)	C(10)	C(11)[1]	118.30(15)	O(17)	S(16)	C(18)	105.69(10)
N(12)	C(11)	C(10)[1]	117.84(14)	O(17)	S(16)	C(19)	106.30(8)
O(15)	C(11)	C(10)[1]	119.52(15)				

[1]2-X,1-Y,1-Z.

Table S22. Values of torsion angles for **9a**.

A	B	C	D	Angle/°	A	B	C	D	Angle/°
C(1)	C(2)	C(3)	C(4)	−0.6(3)	N(6)	C(5)	C(13)	C(1)	173.80(15)
C(1)	C(13)	N(12)	C(11)	124.12(19)	N(6)	C(5)	C(13)	N(12)	−0.6(2)
C(2)	C(1)	C(13)	C(5)	−0.8(3)	N(6)	C(7)	C(8)	C(9)	122.49(17)
C(2)	C(1)	C(13)	N(12)	173.68(15)	N(6)	C(7)	C(8)	C(10)[1]	−61.3(2)
C(2)	C(3)	C(4)	C(5)	−0.5(3)	O(14)	C(7)	C(8)	C(9)	−56.6(2)
C(3)	C(4)	C(5)	C(13)	0.9(2)	O(14)	C(7)	C(8)	C(10)[1]	119.65(19)
C(3)	C(4)	C(5)	N(6)	−173.25(16)	O(14)	C(7)	N(6)	C(5)	172.28(16)
C(4)	C(5)	C(13)	C(1)	−0.2(2)	O(15)	C(11)	N(12)	C(13)	173.54(16)
C(4)	C(5)	C(13)	N(12)	−174.61(15)	C(21)	C(22)	C(23)	C(24)	−14.0(2)
C(4)	C(5)	N(6)	C(7)	−113.96(19)	C(22)	C(21)	N(20)	C(24)	5.2(2)
C(5)	C(13)	N(12)	C(11)	−61.4(2)	C(22)	C(21)	N(20)	C(25)	169.27(19)
C(7)	C(8)	C(9)	C(10)	177.09(15)	C(22)	C(23)	C(24)	N(20)	16.6(2)
C(8)	C(7)	N(6)	C(5)	−6.8(3)	C(23)	C(24)	N(20)	C(21)	−14.0(2)
C(8)	C(9)	C(10)	C(8)[1]	−0.8(3)	C(23)	C(24)	N(20)	C(25)	−178.03(18)
C(8)	C(9)	C(10)	C(11)[1]	173.15(15)	N(20)	C(21)	C(22)	C(23)	6.1(2)
C(10)[1]	C(8)	C(9)	C(10)	0.8(3)	O(26)	C(21)	C(22)	C(23)	−170.7(2)
C(10)[1]	C(11)	N(12)	C(13)	−7.3(3)	O(26)	C(21)	N(20)	C(24)	−178.1(2)
C(13)	C(1)	C(2)	C(3)	1.2(3)	O(26)	C(21)	N(20)	C(25)	−14.0(3)
C(13)	C(5)	N(6)	C(7)	72.0(2)					

[1]2-X,1-Y,1-Z.

Table S23. Bond lengths for **9c**.

Atom	Atom	Length/Å	Atom	Atom	Length/Å
C(1)	C(2)	1.385(3)	C(28)	C(29)	1.384(3)
C(1)	C(26)	1.389(3)	C(28)	C(33)	1.388(3)
C(2)	C(3)	1.386(3)	C(29)	C(30)	1.393(3)
C(3)	C(4)	1.387(3)	C(30)	C(31)	1.370(3)
C(4)	C(5)	1.388(3)	C(31)	C(32)	1.381(3)
C(5)	C(26)	1.401(3)	C(32)	C(33)	1.385(3)
C(5)	N(6)	1.440(2)	C(36)	C(37)	1.509(3)
C(7)	C(8)	1.498(3)	C(36)	N(12)	1.479(2)
C(7)	N(6)	1.357(2)	C(37)	C(38)	1.388(3)
C(7)	O(34)	1.227(2)	C(37)	C(42)	1.389(3)
C(8)	C(9)	1.390(3)	C(38)	C(39)	1.385(3)
C(8)	C(23)	1.399(2)	C(39)	C(40)	1.386(3)
C(9)	C(10)	1.389(3)	C(40)	C(41)	1.382(3)
C(10)	C(11)	1.506(3)	C(41)	C(42)	1.392(3)
C(10)	C(21)	1.402(3)	C(43)	C(44)	1.508(3)
C(11)	N(12)	1.357(2)	C(43)	N(19)	1.468(2)
C(11)	O(35)	1.228(2)	C(44)	C(45)	1.389(3)
C(13)	C(14)	1.391(3)	C(44)	C(49)	1.388(3)
C(13)	C(18)	1.394(3)	C(45)	C(46)	1.388(3)
C(13)	N(12)	1.437(2)	C(46)	C(47)	1.382(4)
C(14)	C(15)	1.381(3)	C(47)	C(48)	1.380(4)
C(15)	C(16)	1.386(3)	C(48)	C(49)	1.388(3)
C(16)	C(17)	1.388(3)	C(52)	C(53)	1.515(3)
C(17)	C(18)	1.389(3)	C(52)	N(25)	1.480(2)

C(18)	N(19)	1.433(2)	C(53)	C(54)	1.389(3)
C(20)	C(21)	1.502(3)	C(53)	C(58)	1.396(3)
C(20)	N(19)	1.356(2)	C(54)	C(55)	1.384(3)
C(20)	O(50)	1.226(2)	C(55)	C(56)	1.382(3)
C(21)	C(22)	1.391(3)	C(56)	C(57)	1.385(3)
C(22)	C(23)	1.387(3)	C(57)	C(58)	1.385(3)
C(23)	C(24)	1.508(2)	C(60)	C(61)	1.528(4)
C(24)	N(25)	1.355(2)	C(60)	N(59)	1.322(3)
C(24)	O(51)	1.228(2)	C(60)	O(64)	1.214(3)
C(26)	N(25)	1.433(2)	C(62)	N(59)	1.469(3)
C(27)	C(28)	1.507(3)	C(63)	N(59)	1.475(3)
C(27)	N(6)	1.475(2)			

Table S24. Values of valence angles for **9c**.

Atom	Atom	Atom	Angle/°	Atom	Atom	Atom	Angle/°
C(2)	C(1)	C(26)	120.50(17)	C(28)	C(29)	C(30)	120.2(2)
C(1)	C(2)	C(3)	120.02(18)	C(31)	C(30)	C(29)	120.4(2)
C(2)	C(3)	C(4)	119.94(18)	C(30)	C(31)	C(32)	119.7(2)
C(3)	C(4)	C(5)	120.40(18)	C(31)	C(32)	C(33)	120.2(2)
C(4)	C(5)	C(26)	119.66(17)	C(32)	C(33)	C(28)	120.4(2)
C(4)	C(5)	N(6)	118.87(16)	N(12)	C(36)	C(37)	112.46(15)
C(26)	C(5)	N(6)	121.45(16)	C(38)	C(37)	C(36)	119.89(18)
N(6)	C(7)	C(8)	115.01(16)	C(38)	C(37)	C(42)	118.80(19)
O(34)	C(7)	C(8)	121.10(17)	C(42)	C(37)	C(36)	121.31(18)
O(34)	C(7)	N(6)	123.88(18)	C(39)	C(38)	C(37)	120.93(19)
C(9)	C(8)	C(7)	119.04(16)	C(38)	C(39)	C(40)	120.0(2)
C(9)	C(8)	C(23)	119.59(17)	C(41)	C(40)	C(39)	119.6(2)
C(23)	C(8)	C(7)	121.35(17)	C(40)	C(41)	C(42)	120.37(19)
C(10)	C(9)	C(8)	120.42(17)	C(37)	C(42)	C(41)	120.31(19)
C(9)	C(10)	C(11)	117.07(16)	N(19)	C(43)	C(44)	116.01(16)
C(9)	C(10)	C(21)	119.56(17)	C(45)	C(44)	C(43)	121.24(18)
C(21)	C(10)	C(11)	123.35(17)	C(49)	C(44)	C(43)	119.60(19)
N(12)	C(11)	C(10)	116.77(15)	C(49)	C(44)	C(45)	119.0(2)
O(35)	C(11)	C(10)	120.50(17)	C(46)	C(45)	C(44)	120.0(2)
O(35)	C(11)	N(12)	122.65(17)	C(47)	C(46)	C(45)	120.6(2)
C(14)	C(13)	C(18)	119.90(17)	C(48)	C(47)	C(46)	119.7(2)
C(14)	C(13)	N(12)	119.55(16)	C(47)	C(48)	C(49)	119.9(2)
C(18)	C(13)	N(12)	120.54(16)	C(44)	C(49)	C(48)	120.8(2)
C(15)	C(14)	C(13)	120.27(18)	N(25)	C(52)	C(53)	113.87(15)
C(14)	C(15)	C(16)	119.83(18)	C(54)	C(53)	C(52)	121.42(17)
C(15)	C(16)	C(17)	120.39(18)	C(54)	C(53)	C(58)	118.39(18)
C(16)	C(17)	C(18)	119.93(18)	C(58)	C(53)	C(52)	120.17(17)
C(13)	C(18)	N(19)	120.26(16)	C(55)	C(54)	C(53)	120.89(19)
C(17)	C(18)	C(13)	119.67(17)	C(56)	C(55)	C(54)	120.34(19)
C(17)	C(18)	N(19)	120.07(16)	C(55)	C(56)	C(57)	119.46(19)
N(19)	C(20)	C(21)	116.06(16)	C(58)	C(57)	C(56)	120.3(2)
O(50)	C(20)	C(21)	120.52(17)	C(57)	C(58)	C(53)	120.65(18)
O(50)	C(20)	N(19)	123.42(18)	C(5)	N(6)	C(27)	118.34(15)

C(10)	C(21)	C(20)	121.03(16)	C(7)	N(6)	C(5)	121.40(16)
C(22)	C(21)	C(10)	119.94(17)	C(7)	N(6)	C(27)	120.09(16)
C(22)	C(21)	C(20)	118.81(16)	C(11)	N(12)	C(13)	122.37(15)
C(23)	C(22)	C(21)	120.00(16)	C(11)	N(12)	C(36)	119.03(15)
C(8)	C(23)	C(24)	121.53(17)	C(13)	N(12)	C(36)	118.18(15)
C(22)	C(23)	C(8)	120.15(17)	C(18)	N(19)	C(43)	118.85(15)
C(22)	C(23)	C(24)	118.29(16)	C(20)	N(19)	C(18)	121.01(16)
N(25)	C(24)	C(23)	115.42(15)	C(20)	N(19)	C(43)	119.57(16)
O(51)	C(24)	C(23)	120.82(16)	C(24)	N(25)	C(26)	121.41(15)
O(51)	C(24)	N(25)	123.68(17)	C(24)	N(25)	C(52)	119.06(15)
C(1)	C(26)	C(5)	119.47(17)	C(26)	N(25)	C(52)	119.50(15)
C(1)	C(26)	N(25)	119.85(16)	N(59)	C(60)	C(61)	114.8(2)
C(5)	C(26)	N(25)	120.65(16)	O(64)	C(60)	C(61)	122.3(2)
N(6)	C(27)	C(28)	111.92(15)	O(64)	C(60)	N(59)	122.9(2)
C(29)	C(28)	C(27)	120.88(18)	C(60)	N(59)	C(62)	125.1(2)
C(29)	C(28)	C(33)	118.97(19)	C(60)	N(59)	C(63)	118.2(2)
C(33)	C(28)	C(27)	120.14(17)	C(62)	N(59)	C(63)	116.65(19)

Table S25. Values of torsion angles for **9c**.

A	B	C	D	Angle/°	A	B	C	D	Angle/°
C(1)	C(2)	C(3)	C(4)	−0.4(3)	C(28)	C(29)	C(30)	C(31)	−0.7(4)
C(1)	C(26)	N(25)	C(24)	110.8(2)	C(29)	C(28)	C(33)	C(32)	1.4(3)
C(1)	C(26)	N(25)	C(52)	−67.1(2)	C(29)	C(30)	C(31)	C(32)	1.2(4)
C(2)	C(1)	C(26)	C(5)	0.8(3)	C(30)	C(31)	C(32)	C(33)	−0.4(3)
C(2)	C(1)	C(26)	N(25)	−177.31(17)	C(31)	C(32)	C(33)	C(28)	−0.9(3)
C(2)	C(3)	C(4)	C(5)	1.2(3)	C(33)	C(28)	C(29)	C(30)	−0.5(3)
C(3)	C(4)	C(5)	C(26)	−1.0(3)	C(36)	C(37)	C(38)	C(39)	−178.84(18)
C(3)	C(4)	C(5)	N(6)	177.25(17)	C(36)	C(37)	C(42)	C(41)	179.86(17)
C(4)	C(5)	C(26)	C(1)	0.0(3)	C(37)	C(36)	N(12)	C(11)	89.8(2)
C(4)	C(5)	C(26)	N(25)	178.07(17)	C(37)	C(36)	N(12)	C(13)	−97.5(2)
C(4)	C(5)	N(6)	C(7)	−103.5(2)	C(37)	C(38)	C(39)	C(40)	−1.0(3)
C(4)	C(5)	N(6)	C(27)	71.8(2)	C(38)	C(37)	C(42)	C(41)	−0.1(3)
C(5)	C(26)	N(25)	C(24)	−67.3(2)	C(38)	C(39)	C(40)	C(41)	−0.1(3)
C(5)	C(26)	N(25)	C(52)	114.79(19)	C(39)	C(40)	C(41)	C(42)	1.1(3)
C(7)	C(8)	C(9)	C(10)	−177.29(16)	C(40)	C(41)	C(42)	C(37)	−1.0(3)
C(7)	C(8)	C(23)	C(22)	−177.45(16)	C(42)	C(37)	C(38)	C(39)	1.1(3)
C(7)	C(8)	C(23)	C(24)	0.7(3)	C(43)	C(44)	C(45)	C(46)	−173.56(19)
C(8)	C(7)	N(6)	C(5)	−4.9(2)	C(43)	C(44)	C(49)	C(48)	172.9(2)
C(8)	C(7)	N(6)	C(27)	179.79(15)	C(44)	C(43)	N(19)	C(18)	−73.7(2)
C(8)	C(9)	C(10)	C(11)	175.86(16)	C(44)	C(43)	N(19)	C(20)	114.83(19)
C(8)	C(9)	C(10)	C(21)	−5.7(3)	C(44)	C(45)	C(46)	C(47)	0.3(3)
C(8)	C(23)	C(24)	N(25)	73.8(2)	C(45)	C(44)	C(49)	C(48)	−1.9(3)
C(8)	C(23)	C(24)	O(51)	−109.2(2)	C(45)	C(46)	C(47)	C(48)	−1.2(3)
C(9)	C(8)	C(23)	C(22)	0.9(3)	C(46)	C(47)	C(48)	C(49)	0.5(4)
C(9)	C(8)	C(23)	C(24)	178.99(16)	C(47)	C(48)	C(49)	C(44)	1.1(4)
C(9)	C(10)	C(11)	N(12)	−118.55(19)	C(49)	C(44)	C(45)	C(46)	1.2(3)
C(9)	C(10)	C(11)	O(35)	58.4(2)	C(52)	C(53)	C(54)	C(55)	−179.32(18)
C(9)	C(10)	C(21)	C(20)	−172.64(16)	C(52)	C(53)	C(58)	C(57)	179.94(19)

C(9)	C(10)	C(21)	C(22)	1.9(3)	C(53)	C(52)	N(25)	C(24)	−86.4(2)
C(10)	C(11)	N(12)	C(13)	4.9(2)	C(53)	C(52)	N(25)	C(26)	91.5(2)
C(10)	C(11)	N(12)	C(36)	177.41(16)	C(53)	C(54)	C(55)	C(56)	−0.1(3)
C(10)	C(21)	C(22)	C(23)	3.3(3)	C(54)	C(53)	C(58)	C(57)	1.3(3)
C(11)	C(10)	C(21)	C(20)	5.7(3)	C(54)	C(55)	C(56)	C(57)	0.3(3)
C(11)	C(10)	C(21)	C(22)	−179.80(16)	C(55)	C(56)	C(57)	C(58)	0.3(3)
C(13)	C(14)	C(15)	C(16)	0.0(3)	C(56)	C(57)	C(58)	C(53)	−1.1(3)
C(13)	C(18)	N(19)	C(20)	74.8(2)	C(58)	C(53)	C(54)	C(55)	−0.7(3)
C(13)	C(18)	N(19)	C(43)	−96.5(2)	N(6)	C(5)	C(26)	C(1)	−178.19(17)
C(14)	C(13)	C(18)	C(17)	−1.0(3)	N(6)	C(5)	C(26)	N(25)	−0.1(3)
C(14)	C(13)	C(18)	N(19)	−179.85(17)	N(6)	C(7)	C(8)	C(9)	113.83(19)
C(14)	C(13)	N(12)	C(11)	106.2(2)	N(6)	C(7)	C(8)	C(23)	−67.8(2)
C(14)	C(13)	N(12)	C(36)	−66.4(2)	N(6)	C(27)	C(28)	C(29)	−125.5(2)
C(14)	C(15)	C(16)	C(17)	−0.7(3)	N(6)	C(27)	C(28)	C(33)	54.9(2)
C(15)	C(16)	C(17)	C(18)	0.6(3)	N(12)	C(13)	C(14)	C(15)	−177.68(18)
C(16)	C(17)	C(18)	C(13)	0.3(3)	N(12)	C(13)	C(18)	C(17)	177.53(17)
C(16)	C(17)	C(18)	N(19)	179.10(18)	N(12)	C(13)	C(18)	N(19)	−1.3(3)
C(17)	C(18)	N(19)	C(20)	−104.0(2)	N(12)	C(36)	C(37)	C(38)	−74.4(2)
C(17)	C(18)	N(19)	C(43)	84.7(2)	N(12)	C(36)	C(37)	C(42)	105.6(2)
C(18)	C(13)	C(14)	C(15)	0.9(3)	N(19)	C(20)	C(21)	C(10)	−70.6(2)
C(18)	C(13)	N(12)	C(11)	−72.4(2)	N(19)	C(20)	C(21)	C(22)	114.77(19)
C(18)	C(13)	N(12)	C(36)	115.1(2)	N(19)	C(43)	C(44)	C(45)	−47.4(3)
C(20)	C(21)	C(22)	C(23)	177.94(16)	N(19)	C(43)	C(44)	C(49)	137.83(19)
C(21)	C(10)	C(11)	N(12)	63.1(2)	N(25)	C(52)	C(53)	C(54)	−109.5(2)
C(21)	C(10)	C(11)	O(35)	−120.0(2)	N(25)	C(52)	C(53)	C(58)	72.0(2)
C(21)	C(20)	N(19)	C(18)	−4.7(2)	O(34)	C(7)	C(8)	C(9)	−65.1(2)
C(21)	C(20)	N(19)	C(43)	166.57(16)	O(34)	C(7)	C(8)	C(23)	113.2(2)
C(21)	C(22)	C(23)	C(8)	−4.7(3)	O(34)	C(7)	N(6)	C(5)	174.00(17)
C(21)	C(22)	C(23)	C(24)	177.15(16)	O(34)	C(7)	N(6)	C(27)	−1.3(3)
C(22)	C(23)	C(24)	N(25)	−108.08(19)	O(35)	C(11)	N(12)	C(13)	−171.92(17)
C(22)	C(23)	C(24)	O(51)	68.9(2)	O(35)	C(11)	N(12)	C(36)	0.5(3)
C(23)	C(8)	C(9)	C(10)	4.3(3)	O(50)	C(20)	C(21)	C(10)	108.5(2)
C(23)	C(24)	N(25)	C(26)	−4.1(2)	O(50)	C(20)	C(21)	C(22)	−66.0(2)
C(23)	C(24)	N(25)	C(52)	173.81(15)	O(50)	C(20)	N(19)	C(18)	176.17(17)
C(26)	C(1)	C(2)	C(3)	−0.6(3)	O(50)	C(20)	N(19)	C(43)	−12.6(3)
C(26)	C(5)	N(6)	C(7)	74.6(2)	O(51)	C(24)	N(25)	C(26)	178.98(17)
C(26)	C(5)	N(6)	C(27)	−110.0(2)	O(51)	C(24)	N(25)	C(52)	−3.1(3)
C(27)	C(28)	C(29)	C(30)	179.9(2)	C(61)	C(60)	N(59)	C(62)	−1.4(3)
C(27)	C(28)	C(33)	C(32)	−179.04(18)	C(61)	C(60)	N(59)	C(63)	−179.1(2)
C(28)	C(27)	N(6)	C(5)	81.2(2)	O(64)	C(60)	N(59)	C(62)	179.6(2)
C(28)	C(27)	N(6)	C(7)	−103.39(19)	O(64)	C(60)	N(59)	C(63)	2.0(4)

Table S26. Bond lengths for **10**.

Atom	Atom	Length/Å	Atom	Atom	Length/Å
C(2)	C(3)	1.495(3)	C(9)	N(1)	1.415(3)
C(2)	N(1)	1.404(3)	C(9)	O(18)	1.208(3)
C(2)	O(17)	1.212(3)	C(10)	C(11)	1.399(3)
C(3)	C(4)	1.384(3)	C(10)	C(15)	1.403(3)

C(3)	C(8)	1.390(3)	C(10)	N(1)	1.438(3)
C(4)	C(5)	1.399(3)	C(11)	C(12)	1.387(3)
C(5)	C(6)	1.390(4)	C(12)	C(13)	1.391(4)
C(6)	C(7)	1.396(3)	C(13)	C(14)	1.384(4)
C(7)	C(8)	1.387(3)	C(14)	C(15)	1.412(3)
C(8)	C(9)	1.487(3)	C(15)	N(16)	1.365(3)

Table S27. Values of valence angles for **10**.

Atom	Atom	Atom	Angle/°	Atom	Atom	Atom	Angle/°
N(1)	C(2)	C(3)	106.19(18)	O(18)	C(9)	N(1)	125.54(19)
O(17)	C(2)	C(3)	129.0(2)	C(11)	C(10)	C(15)	121.2(2)
O(17)	C(2)	N(1)	124.83(19)	C(11)	C(10)	N(1)	118.5(2)
C(4)	C(3)	C(2)	130.6(2)	C(15)	C(10)	N(1)	120.2(2)
C(4)	C(3)	C(8)	121.5(2)	C(12)	C(11)	C(10)	120.8(2)
C(8)	C(3)	C(2)	107.84(18)	C(11)	C(12)	C(13)	118.6(2)
C(3)	C(4)	C(5)	116.9(2)	C(14)	C(13)	C(12)	121.0(2)
C(6)	C(5)	C(4)	121.4(2)	C(13)	C(14)	C(15)	121.4(2)
C(5)	C(6)	C(7)	121.6(2)	C(10)	C(15)	C(14)	116.9(2)
C(8)	C(7)	C(6)	116.5(2)	N(16)	C(15)	C(10)	122.3(2)
C(3)	C(8)	C(9)	108.84(18)	N(16)	C(15)	C(14)	120.5(2)
C(7)	C(8)	C(3)	122.1(2)	C(2)	N(1)	C(9)	111.42(17)
C(7)	C(8)	C(9)	129.1(2)	C(2)	N(1)	C(10)	125.43(18)
N(1)	C(9)	C(8)	105.66(18)	C(9)	N(1)	C(10)	123.09(18)
O(18)	C(9)	C(8)	128.8(2)				

Table S28. Values of torsion angles for **10**.

A	B	C	D	Angle/°	A	B	C	D	Angle/°
C(2)	C(3)	C(4)	C(5)	177.3(2)	C(11)	C(10)	C(15)	N(16)	−176.1(2)
C(2)	C(3)	C(8)	C(7)	−177.88(19)	C(11)	C(10)	N(1)	C(2)	−119.2(2)
C(2)	C(3)	C(8)	C(9)	1.9(2)	C(11)	C(10)	N(1)	C(9)	57.7(3)
C(3)	C(2)	N(1)	C(9)	1.5(2)	C(11)	C(12)	C(13)	C(14)	0.2(4)
C(3)	C(2)	N(1)	C(10)	178.72(19)	C(12)	C(13)	C(14)	C(15)	0.1(4)
C(3)	C(4)	C(5)	C(6)	0.1(3)	C(13)	C(14)	C(15)	C(10)	0.3(3)
C(3)	C(8)	C(9)	N(1)	−1.0(2)	C(13)	C(14)	C(15)	N(16)	175.5(2)
C(3)	C(8)	C(9)	O(18)	178.7(2)	C(15)	C(10)	C(11)	C(12)	1.2(3)
C(4)	C(3)	C(8)	C(7)	0.0(3)	C(15)	C(10)	N(1)	C(2)	63.4(3)
C(4)	C(3)	C(8)	C(9)	179.8(2)	C(15)	C(10)	N(1)	C(9)	−119.8(2)
C(4)	C(5)	C(6)	C(7)	−0.1(3)	N(1)	C(2)	C(3)	C(4)	−179.8(2)
C(5)	C(6)	C(7)	C(8)	0.1(3)	N(1)	C(2)	C(3)	C(8)	−2.2(2)
C(6)	C(7)	C(8)	C(3)	0.0(3)	N(1)	C(10)	C(11)	C(12)	−176.2(2)
C(6)	C(7)	C(8)	C(9)	−179.8(2)	N(1)	C(10)	C(15)	C(14)	176.5(2)
C(7)	C(8)	C(9)	N(1)	178.8(2)	N(1)	C(10)	C(15)	N(16)	1.3(3)
C(7)	C(8)	C(9)	O(18)	−1.5(4)	O(17)	C(2)	C(3)	C(4)	0.1(4)
C(8)	C(3)	C(4)	C(5)	−0.1(3)	O(17)	C(2)	C(3)	C(8)	177.8(2)
C(8)	C(9)	N(1)	C(2)	−0.4(2)	O(17)	C(2)	N(1)	C(9)	−178.4(2)
C(8)	C(9)	N(1)	C(10)	−177.64(18)	O(17)	C(2)	N(1)	C(10)	−1.2(3)
C(10)	C(11)	C(12)	C(13)	−0.8(3)	O(18)	C(9)	N(1)	C(2)	179.9(2)
C(11)	C(10)	C(15)	C(14)	−0.9(3)	O(18)	C(9)	N(1)	C(10)	2.6(3)

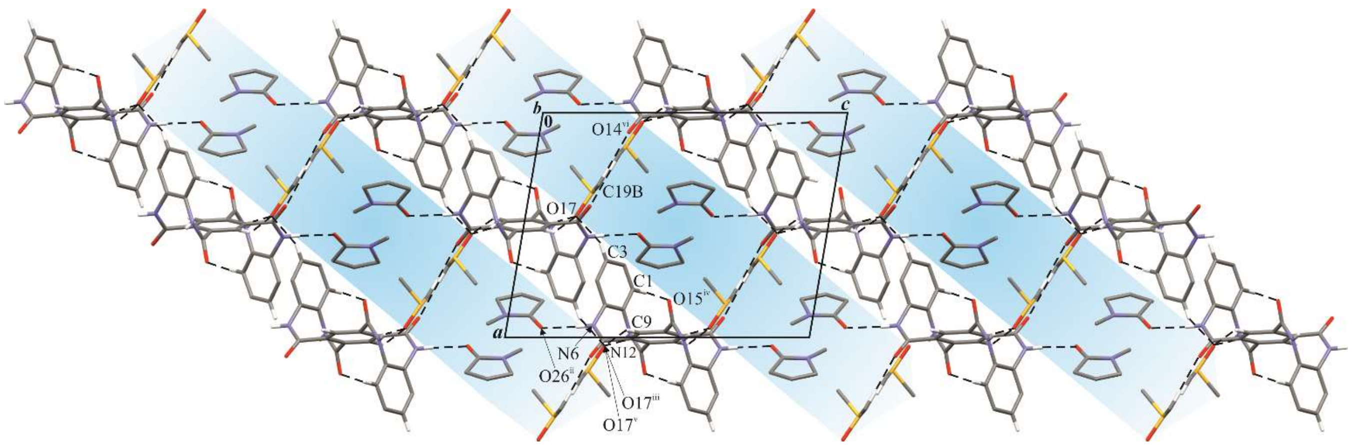

Figure S6. Supramolecular architecture of molecules in the crystal of **9a**, viewed along *b*-direction. The N−H···O and C−H···O hydrogen bonds are represented by a dashed lines. The H-atoms not involved in the intermolecular interactions have been omitted for clarity. The layers built from the solvent molecules are highlighted in blue. Symmetry codes: (ii) *x* + 1/2, −*y* + 3/2, *z* − 1/2; (iii) −*x* + 3/2, *y* + 1/2. −*z* + 1/2; (iv) −*x* + 2, −*y* + 2, −*z* + 1; (v) −*x* + 3/2, *y* − 1/2, −*z* + 1/2; (vi) *x* − 1, *y*, *z*; (vii) −*x* + 1, −*y* + 1, −*z* + 1.

Table S29. Hydrogen-bond geometry in the crystal of **9a**.

D−H···A	d(D−H) [Å]	d(H···A) [Å]	d(D···A) [Å]	<D−H···A [°]
N6−H6···O26^{ii}	0.863(14)	1.943(13)	2.8048(19)	176(2)
N12−H12···O17^{iii}	0.871(17)	1.916(10)	2.7660(19)	165(2)
C1−H1···O15^{iv}	0.93	2.34	3.253(2)	169
C3−H3···O17	0.93	2.46	3.287(2)	148
C9−H9···O17^{v}	0.93	2.48	3.313(2)	149
C19−H19B···O14^{vi}	0.96	2.34	3.268(2)	163

Symmetry codes: (ii) *x* + 1/2, −*y* + 3/2, *z* − 1/2; (iii) −*x* + 3/2, *y* + 1/2. −*z* + 1/2; (iv) −*x* + 2, −*y* + 2, −*z* + 1; (v) −*x* + 3/2, *y* − 1/2, −*z* + 1/2; (vi) *x* − 1, *y*, *z*; (vii) −*x* + 1, −*y* + 1, −*z* + 1. (*) intramolecular interaction.

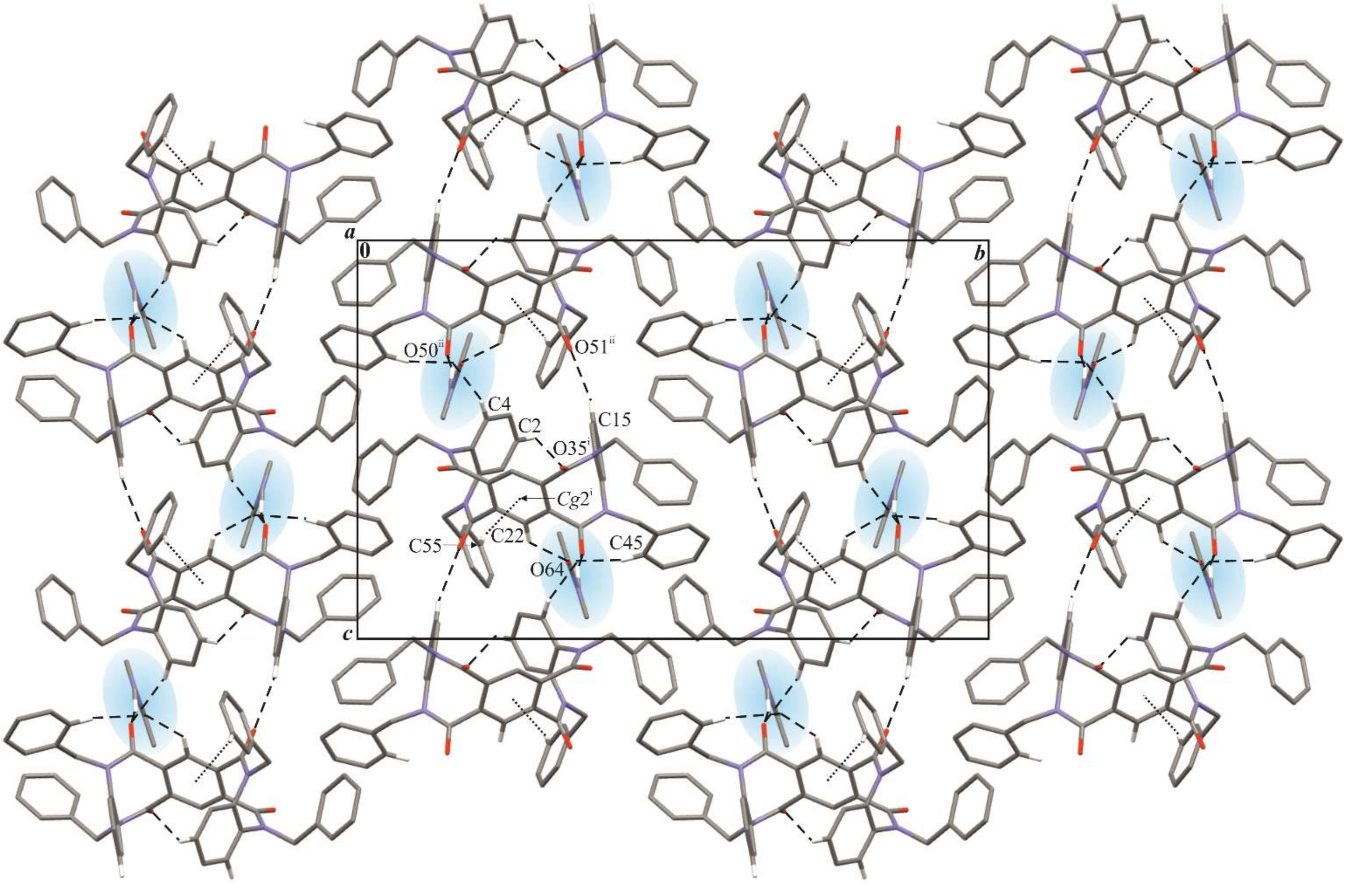

Figure S7. Supramolecular architecture of molecules in the crystal of **9c**, viewed along *a*-direction. The hydrogen bonds have are represented by a dashed lines, while the C−H···π contacts by a dotted lines. The H-atoms not involved in the intermolecular interactions have been omitted for clarity. The solvent molecules are highlighted in blue. Symmetry codes: (i) *x* + 1, *y*, *z*; (ii) *x*, −*y* + 1/2, *z* − 1/2.

Table S30. Hydrogen-bond geometry in the crystal of **9c**.

D−H···A	d(D−H) [Å]	d(H···A) [Å]	d(D···A) [Å]	<D−H···A [°]
C2−H2···O35^{i}	0.93	2.55	3.337(2)	142
C4−H4···O50^{ii}	0.93	2.56	3.414(2)	153
C15−H15···O51^{ii}	0.93	2.56	3.457(2)	163
C22−H22···O64	0.93	2.45	3.229(2)	141
C38−H38···O35*	0.93	2.51	3.206(3)	132
C45−H45···O64	0.93	2.41	3.284(3)	157
C58−H58···O51*	0.93	2.49	3.193(2)	133
C62−H62C···O50^{i}	0.96	2.53	3.185(3)	126

Symmetry codes: (i) *x* + 1, *y*, *z*; (ii) *x*, −*y* + 1/2, *z* − 1/2; (*) intramolecular interaction.

Table S31. The geometry of the C−H···π contacts in the crystal of **9c**.

D−H	*Cg*I	d(H··· *Cg*I) [Å]	d(D···*Cg*I) [Å]	<D−H···*Cg*I [°]
C43−H43B	*Cg*5*	2.86	3.663(2)	141
C55−H55	*Cg*2^{i}	2.81	3.598(2)	143

The *Cg*2 and *Cg*5 denote geometric centers of gravity of the aromatic rings defined by the C8−C10/C21−C23 and C37−C42, respectively (see Fig. S4). Symmetry codes: (i) *x* + 1, *y*, *z*; (*) intramolecular interaction.

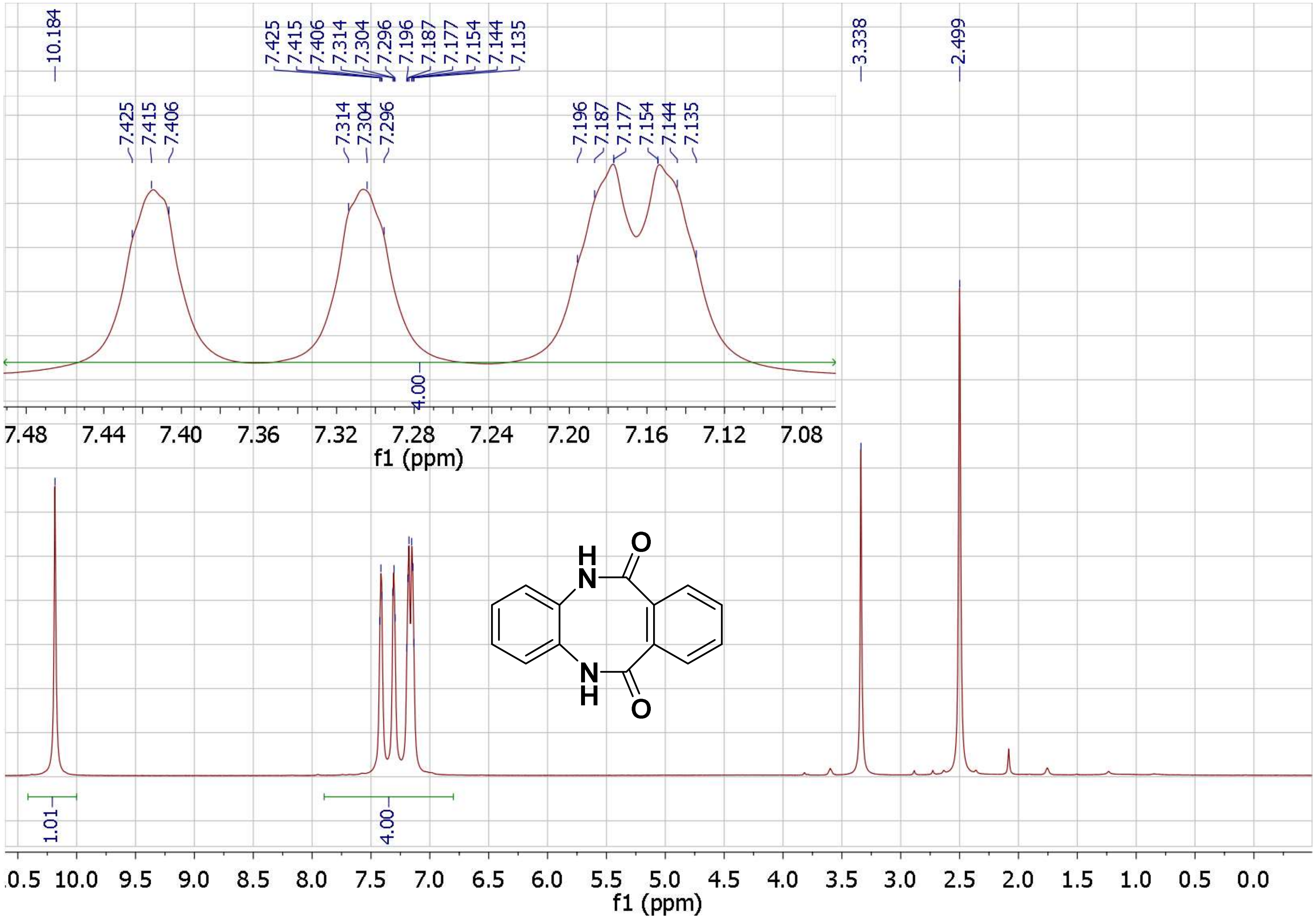

Figure S8. 1H-NMR spectrum for 5,12-dihydrodibenzo[*b*,*f*][1,4]diazocine–6,11-dione (**3a**).

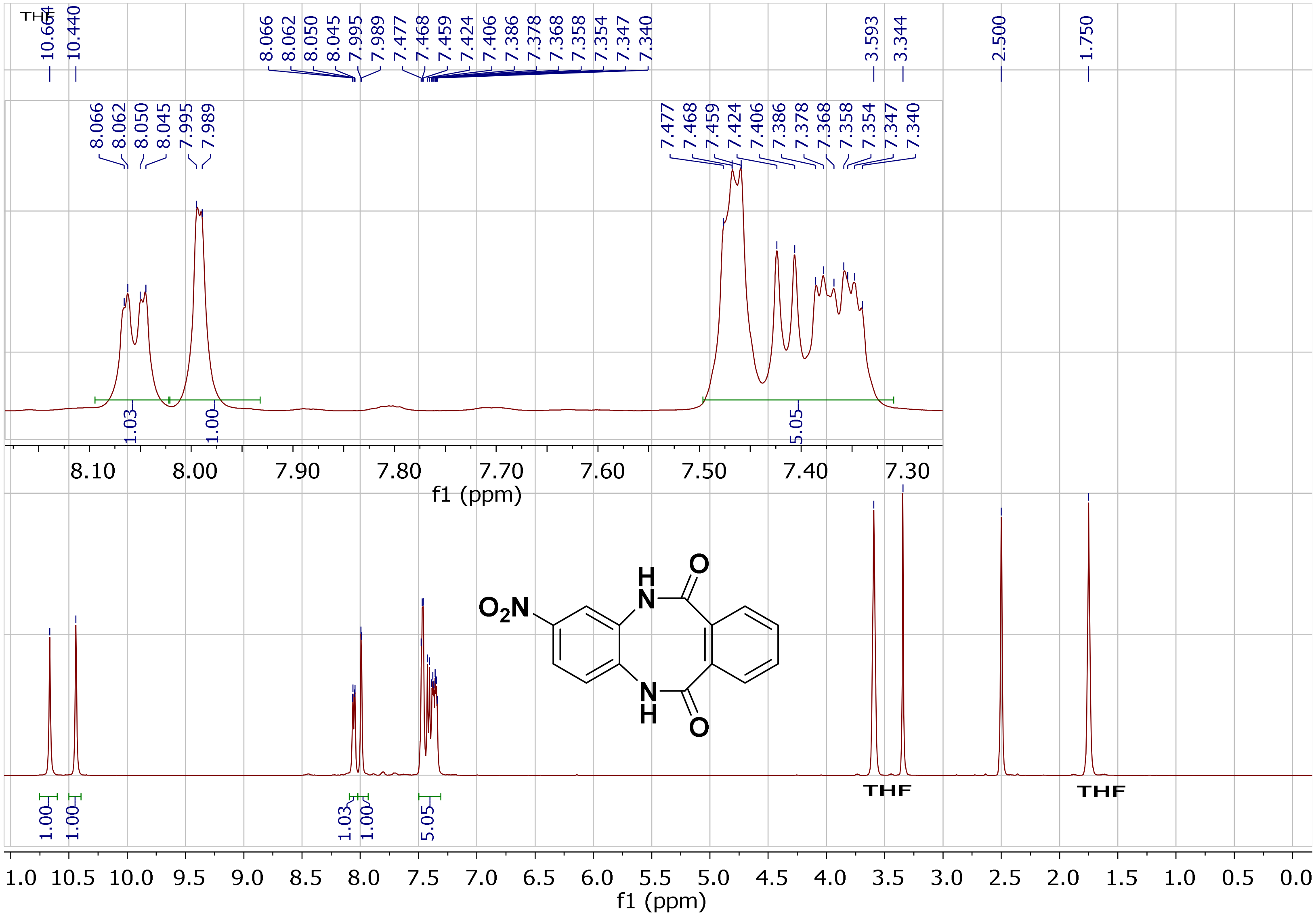

Figure S9. 1H-NMR spectrum for 2-nitro–5,12-dihydrodibenzo[*b*,*f*][1,4]diazocine–6,11-dione (**3b**).

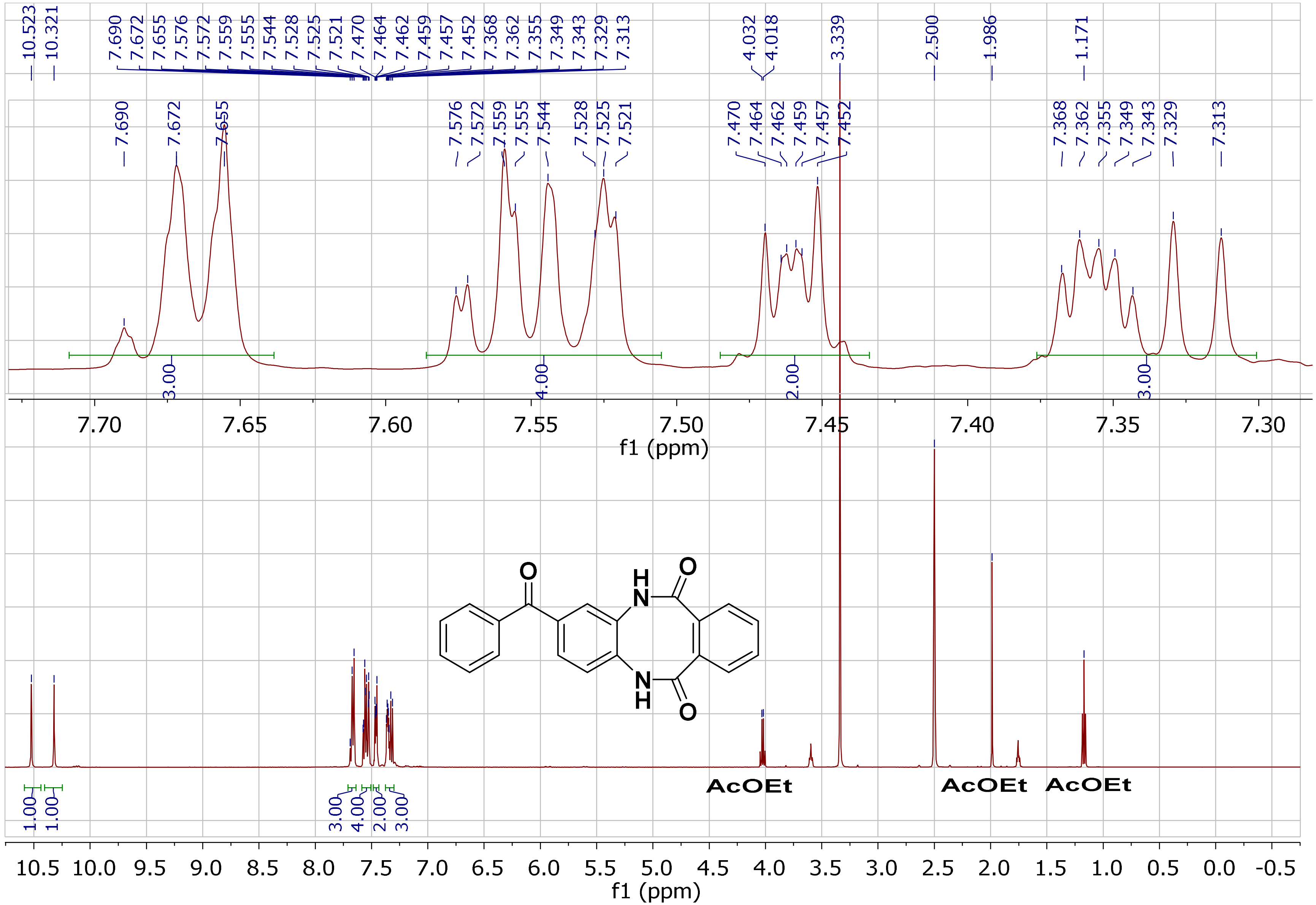

Figure S10. 1H-NMR spectrum for 2-benzoyl–5,12-dihydrodibenzo[*b*,*f*][1,4]diazocine–6,11-dione (**3c**).

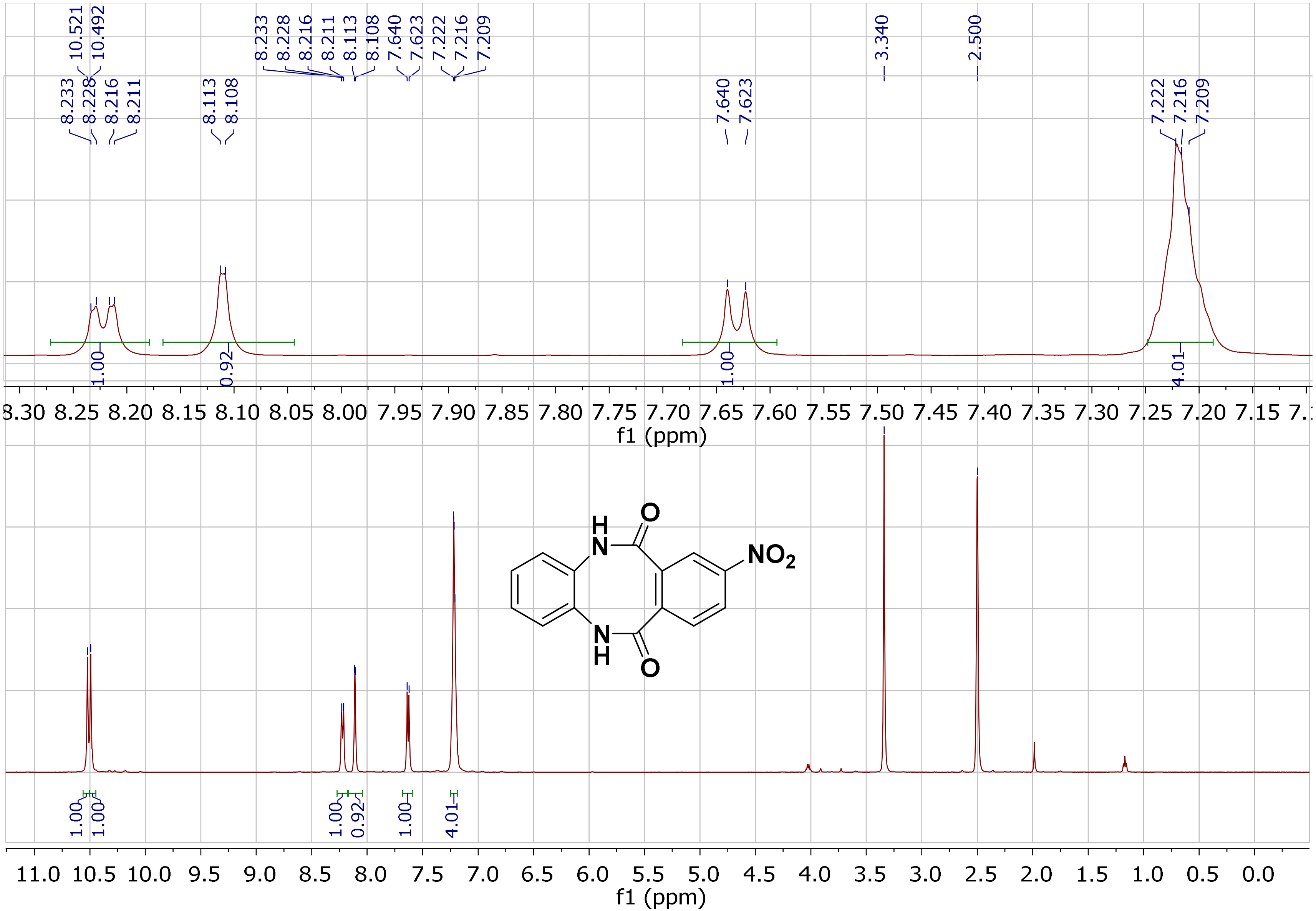

Figure S11. 1H-NMR spectrum for 8-nitro–5,12-dihydrodibenzo[*b*,*f*][1,4]diazocine–6,11-dione (**3d**).

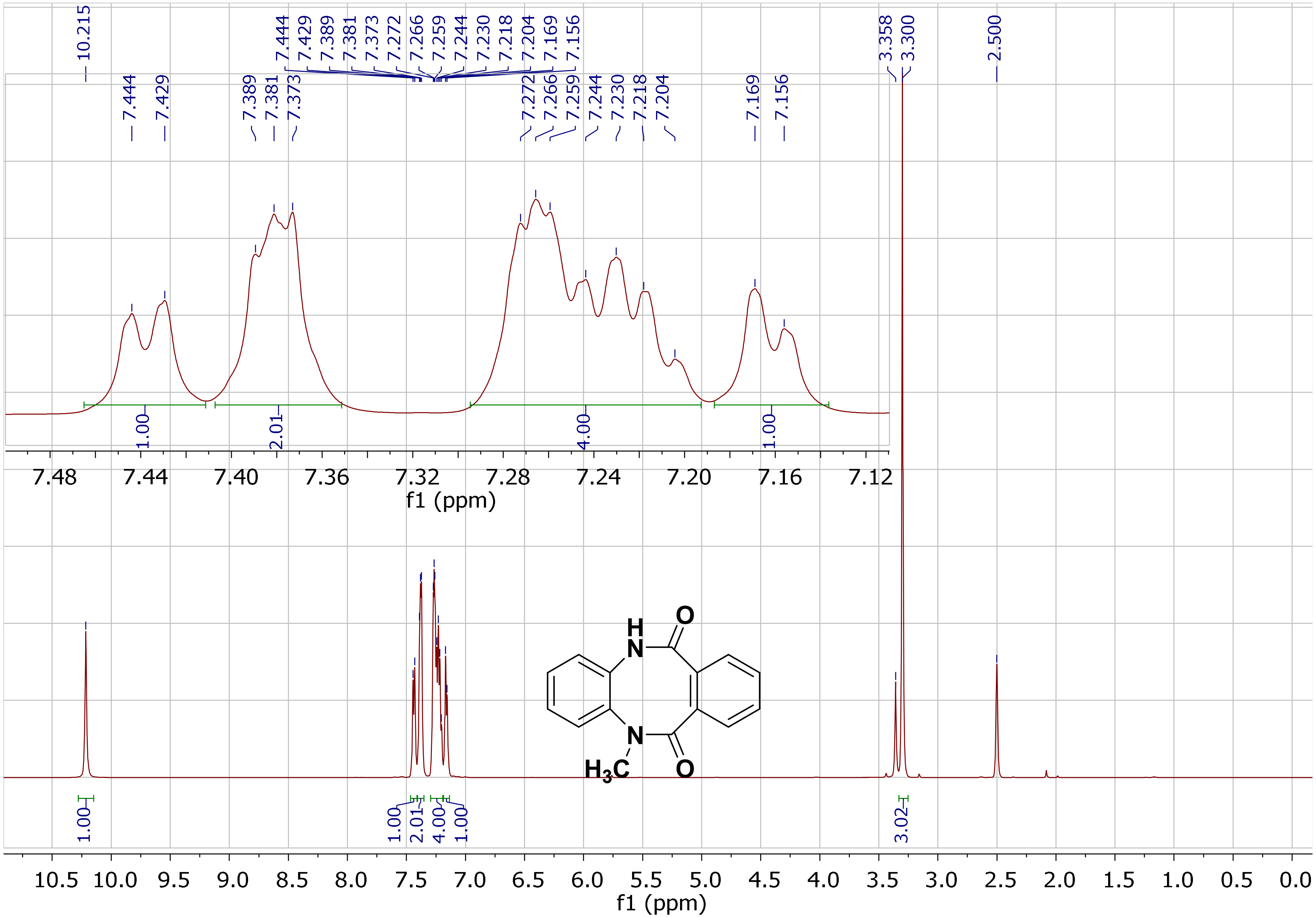

Figure S12. 1H-NMR spectrum for 5-methyl–5,12-dihydrodibenzo[*b*,*f*][1,4]diazocine–6,11-dione (**3e**).

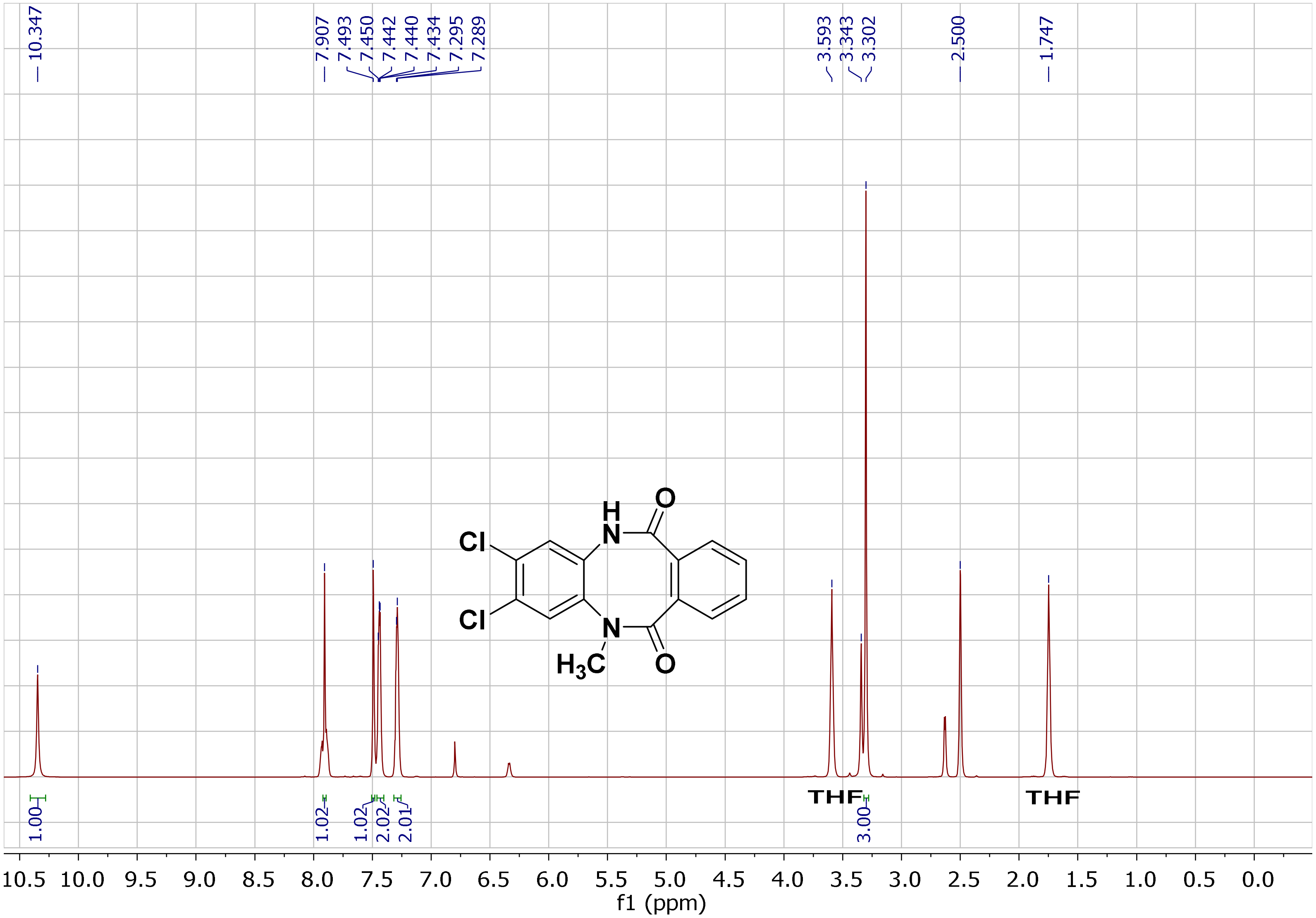

Figure S13. 1H-NMR spectrum for 2,3-dichloro–5-methyl–5,12-dihydrodibenzo[*b*,*f*][1,4]diazocine–6,11-dione (**3f**).

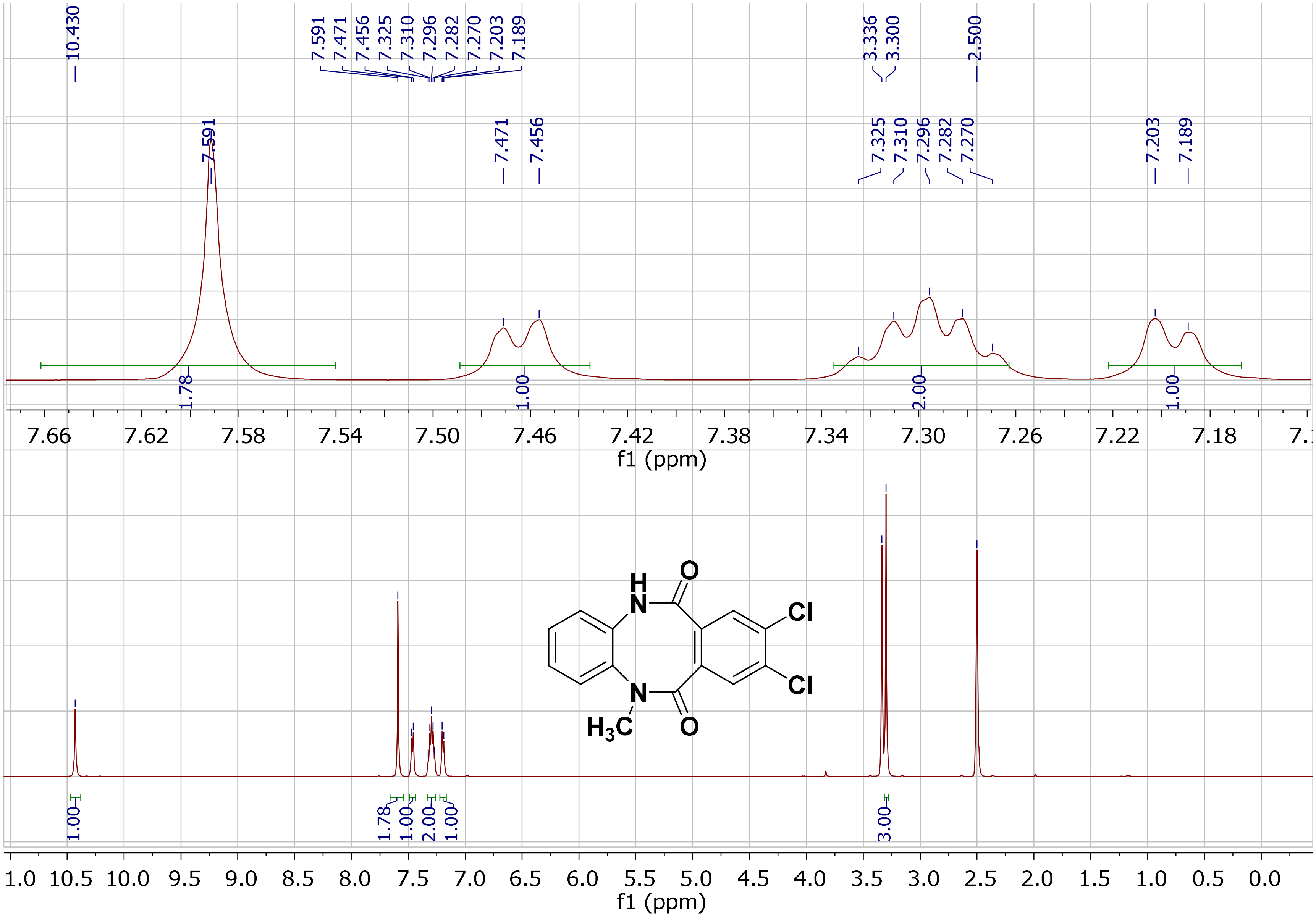

Figure S14. 1H-NMR spectrum for 8,9-dichloro–5-methyl–5,12-dihydrodibenzo[*b*,*f*][1,4]diazocine–6,11-dione (**3g**).

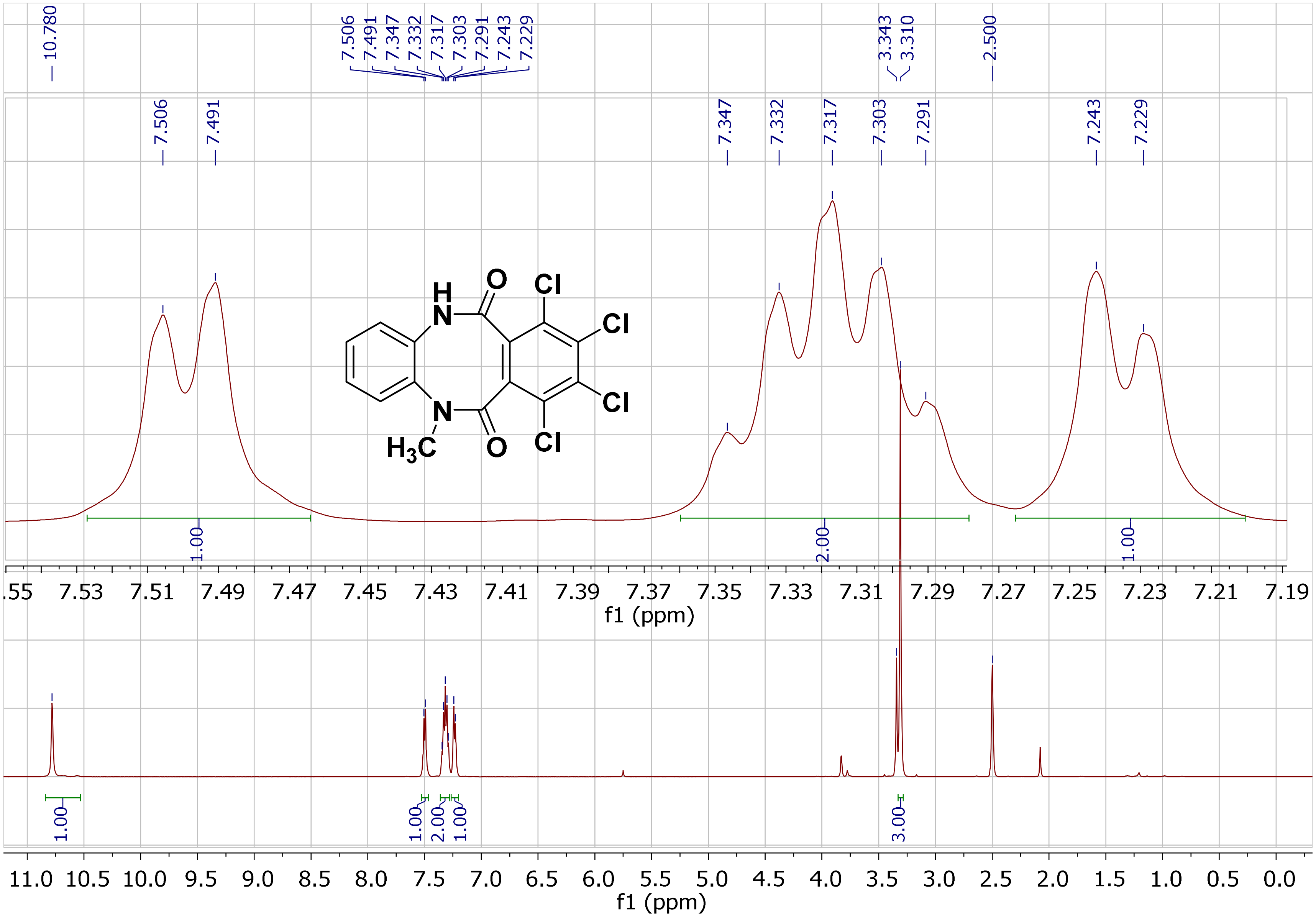

Figure S15. 1H-NMR spectrum for 7,8,9,10-tetrachloro–5-methyl–5,12-dihydrodibenzo[*b,f*][1,4]diazocine–6,11-dione (**3h**).

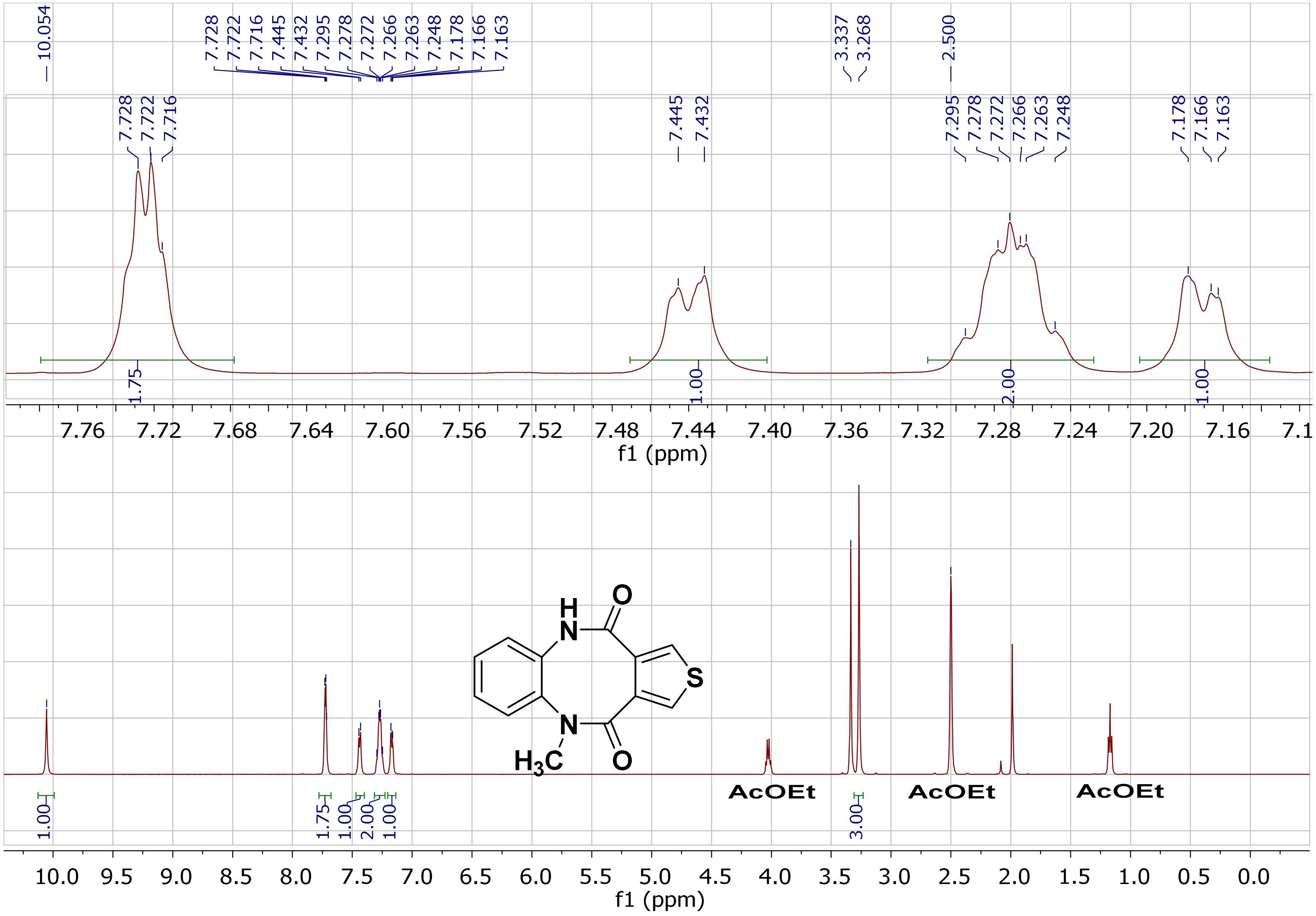

Figure S16. 1H-NMR spectrum for 5-methyl–5,10-dihydrobenzo[*b*]thieno[3,4-*f*][1,4]diazocine–4,11-dione (**3i**).

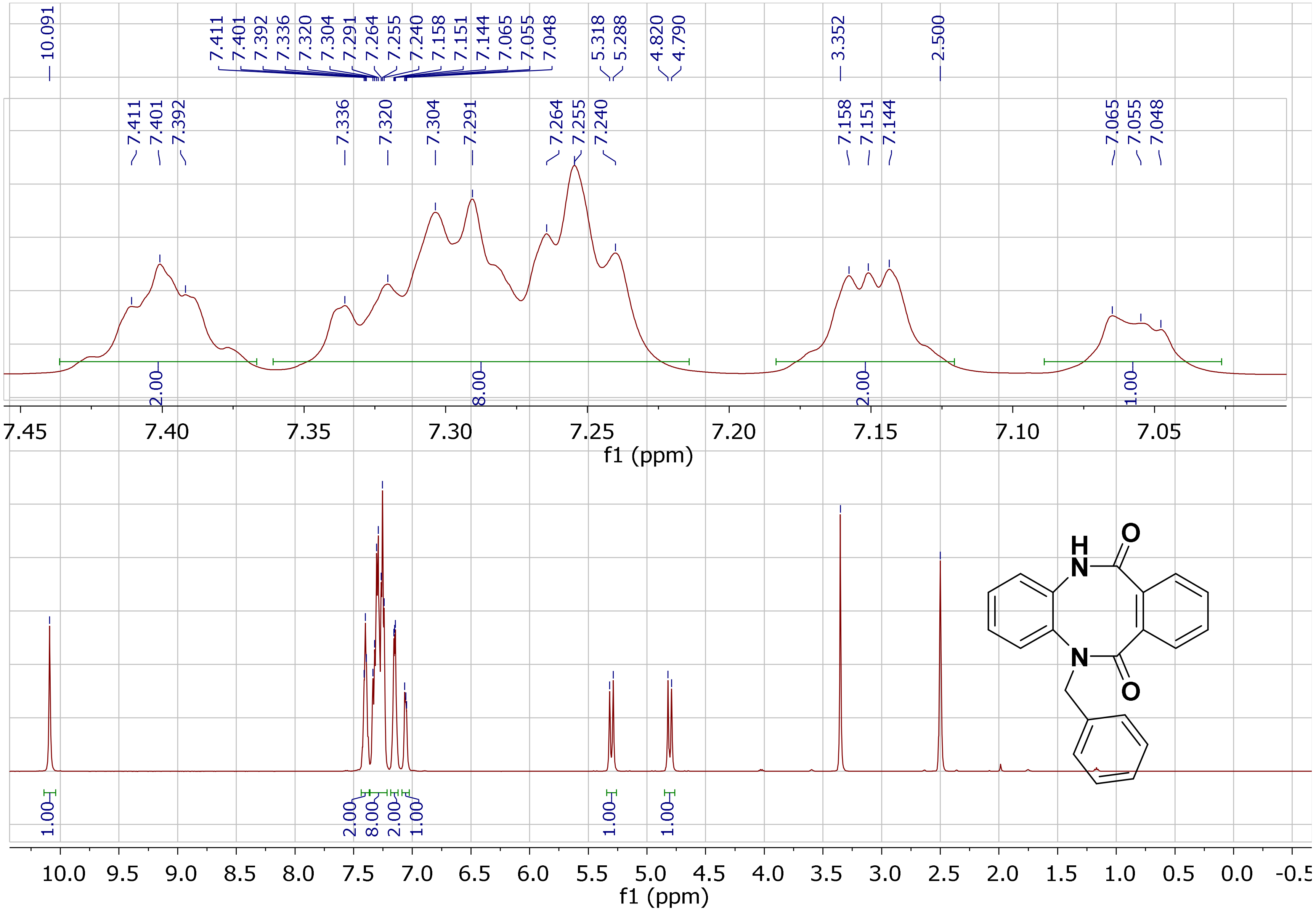

Figure S17. 1H-NMR spectrum for 5-benzyl–5,12-dihydrodibenzo[*b*,*f*][1,4]diazocine–6,11-dione (**3j**).

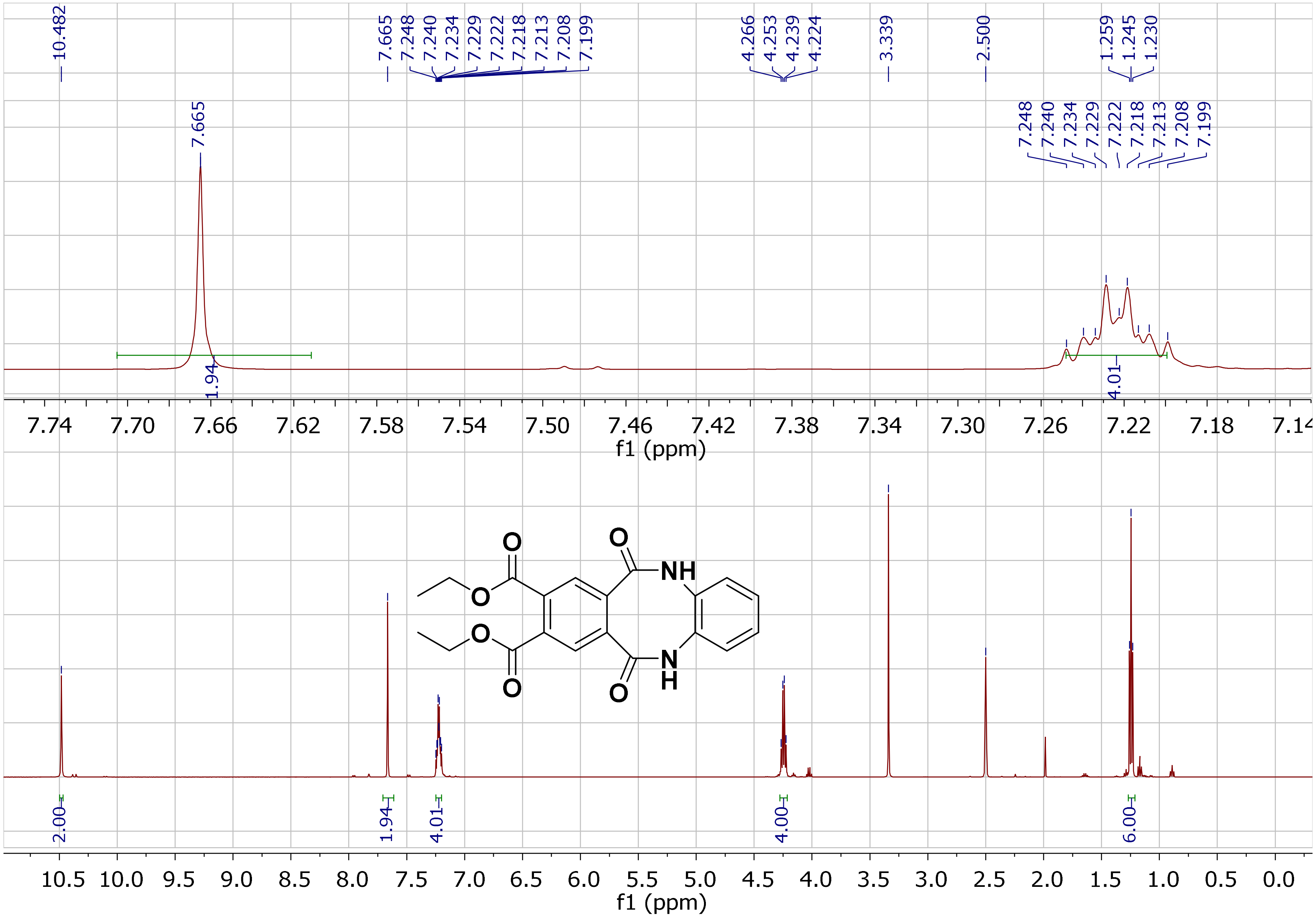

Figure S18. 1H-NMR spectrum for diethyl 6,11-dioxo–5,6,11,12-tetrahydrodibenzo[*b,f*][1,4]diazocine–8,9-dicarboxylate (**3k**).

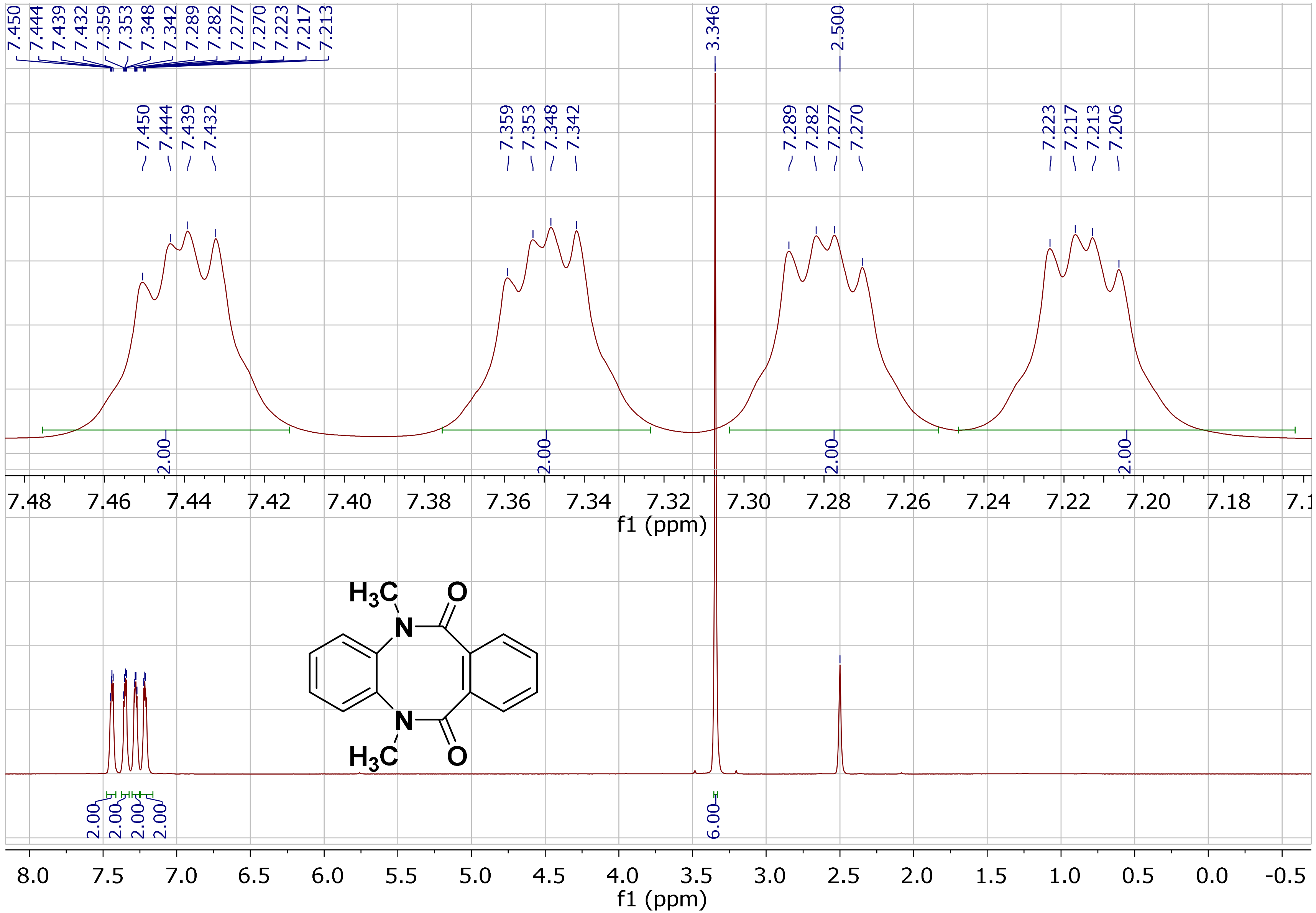

Figure S19. 1H-NMR spectrum for 5,12-dimethyl–5,12-dihydrodibenzo[*b*,*f*][1,4]diazocine–6,11-dione (**3l**).

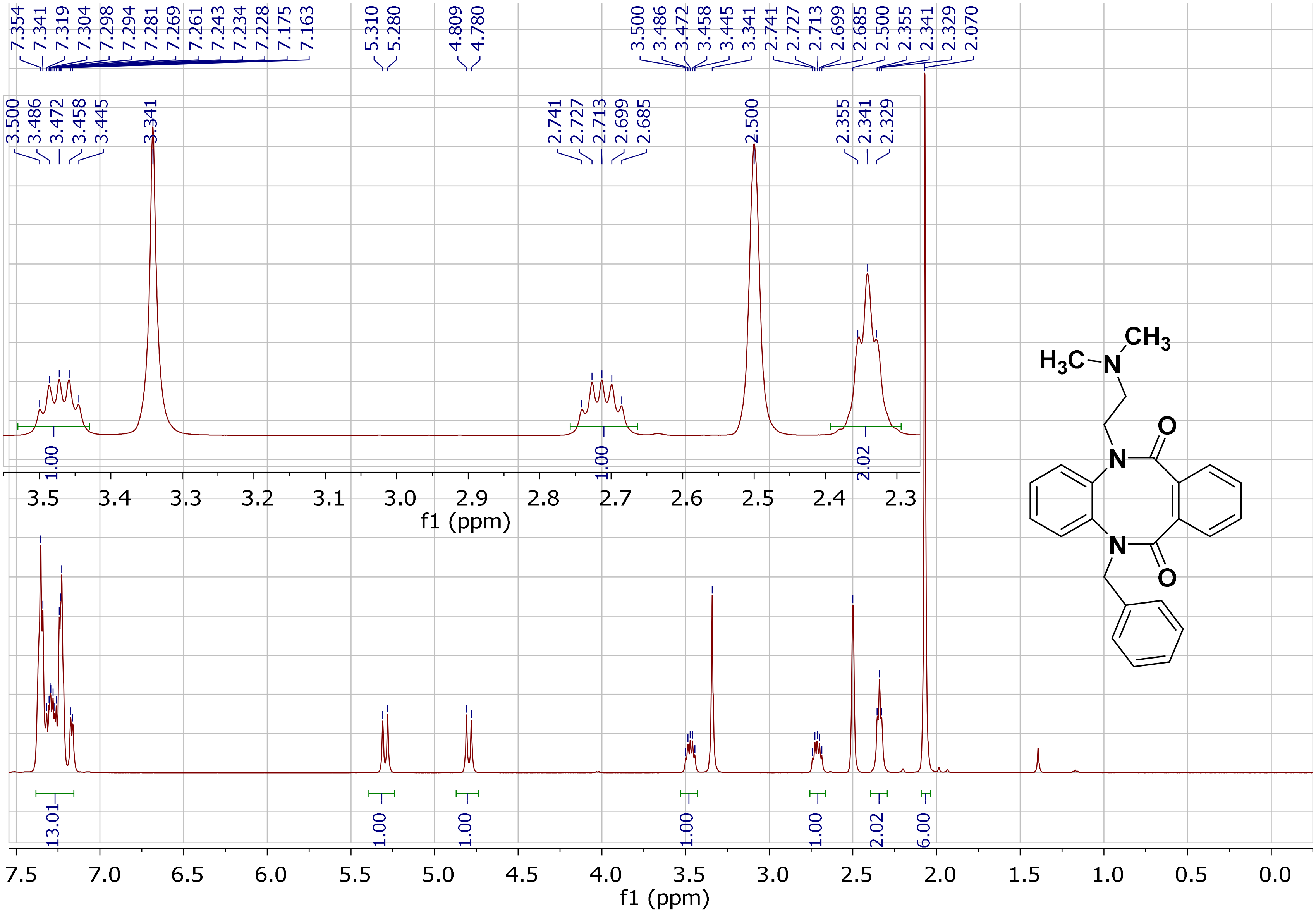

Figure S20. 1H-NMR spectrum for 5-benzyl–12-(2-(dimethylamino)ethyl)–5,12-dihydrodibenzo[*b*,*f*][1,4]diazocine–6,11-dione (**3m**).

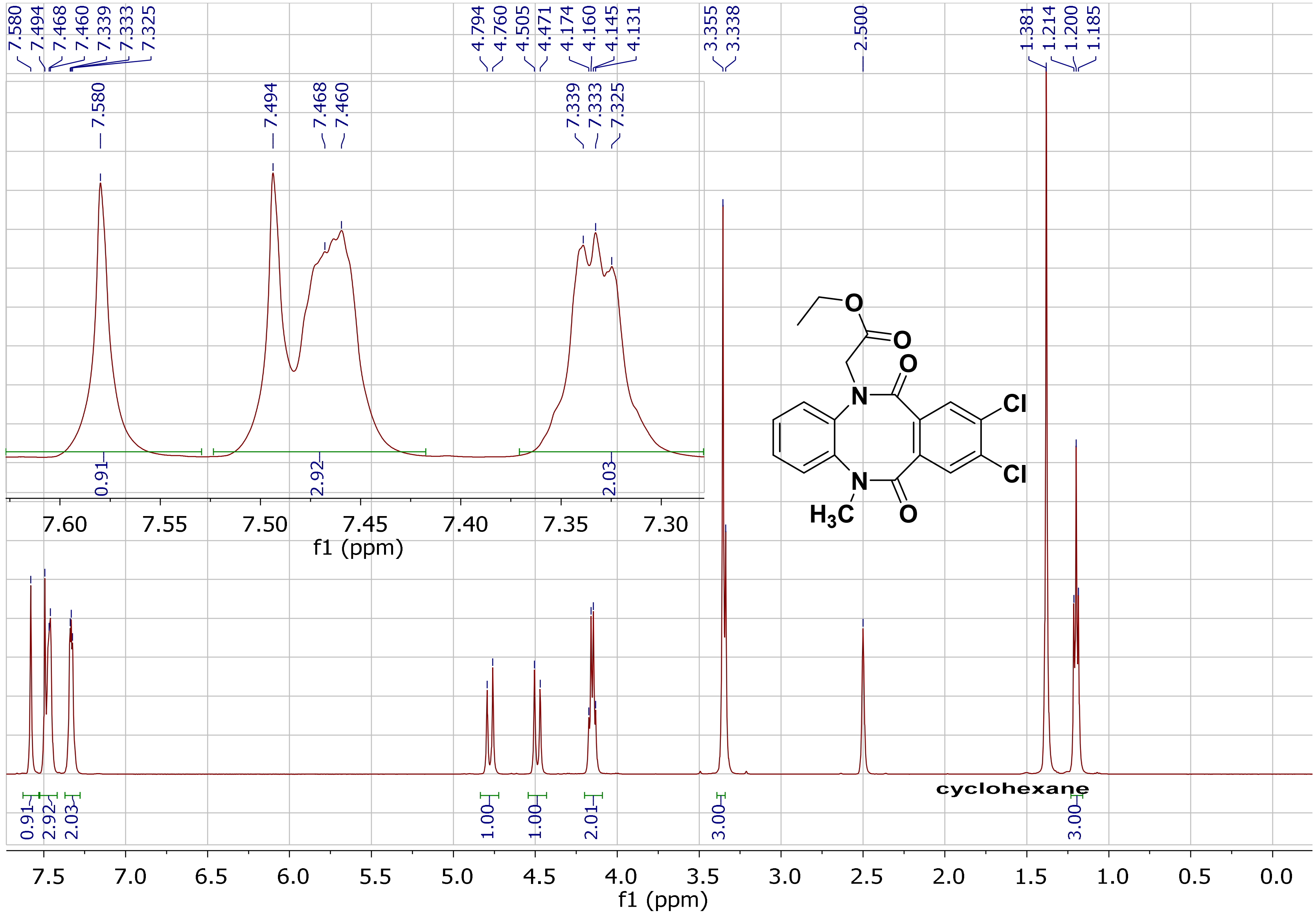

Figure S21. 1H-NMR spectrum for ethyl 2-(8,9-dichloro–12-methyl–6,11-dioxo–11,12-dihydrodibenzo[*b*,*f*][1,4]diazocin–5(6*H*)-yl)acetate (**3n**).

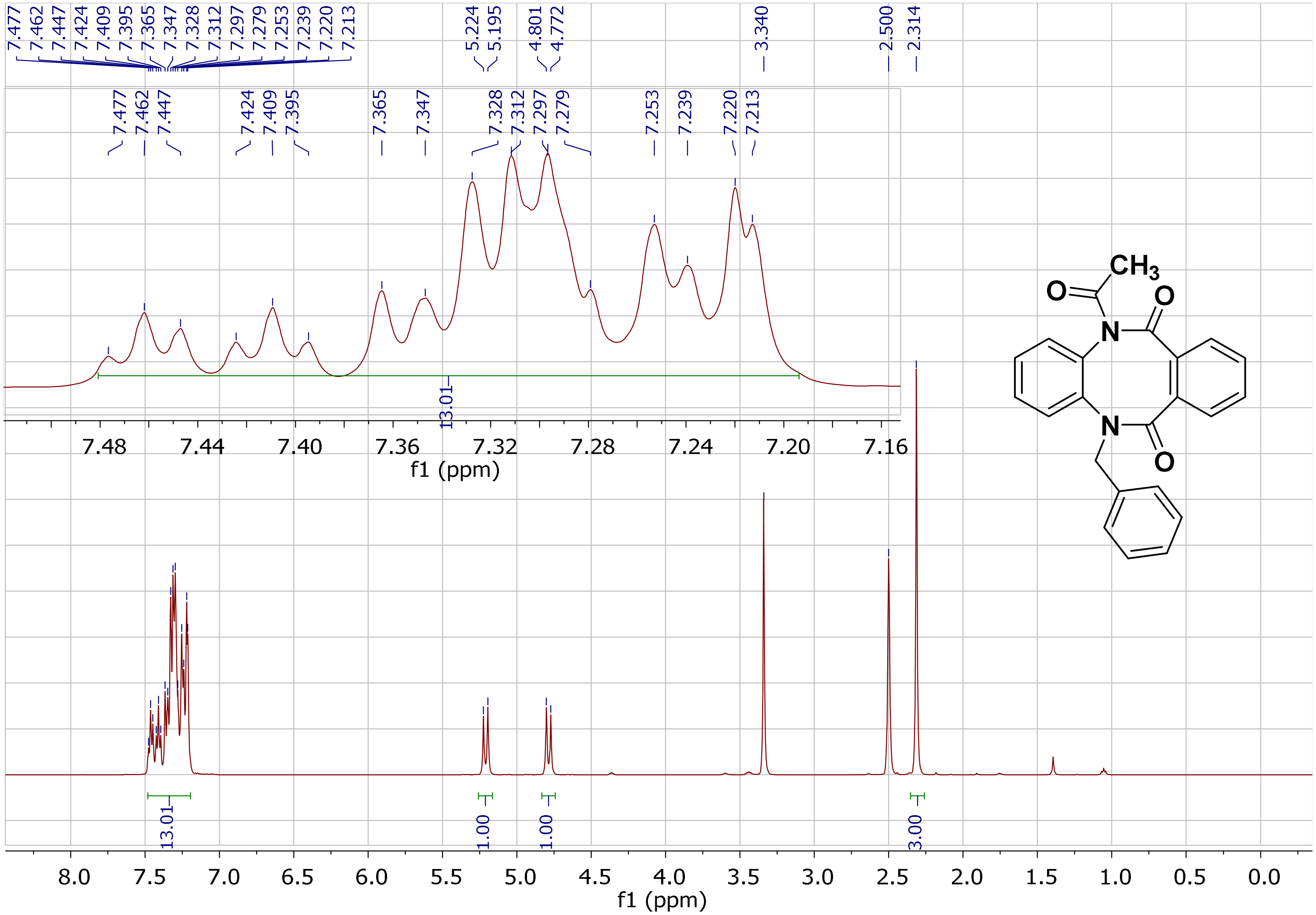

Figure S22. 1H-NMR spectrum for 5-acetyl–12-benzyl–5,12-dihydrodibenzo[*b*,*f*][1,4]diazocine–6,11-dione (**3o**).

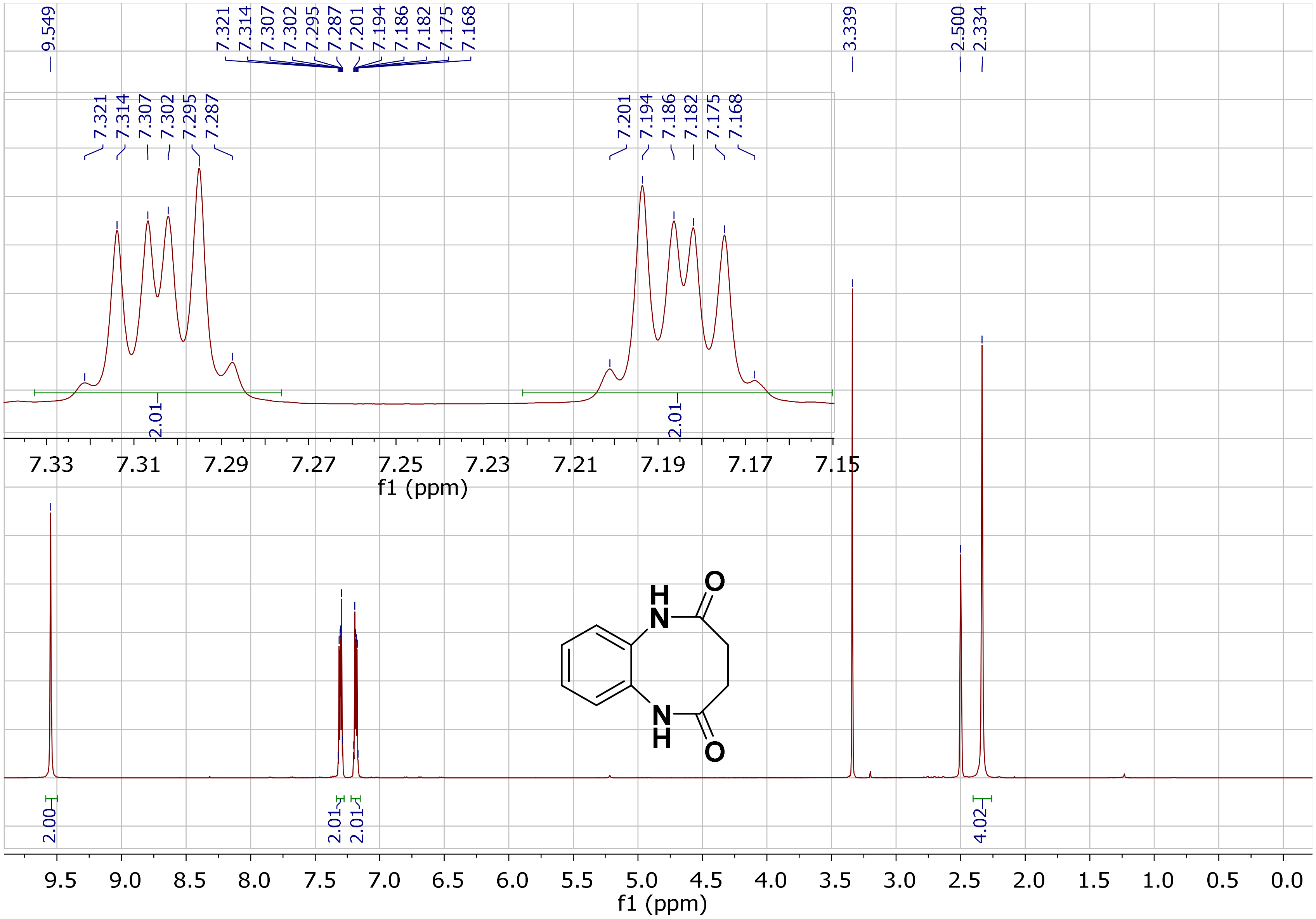

Figure S23. 1H-NMR spectrum for 1,3,4,6-tetrahydrobenzo[*b*][1,4]diazocine–2,5-dione (**6**).

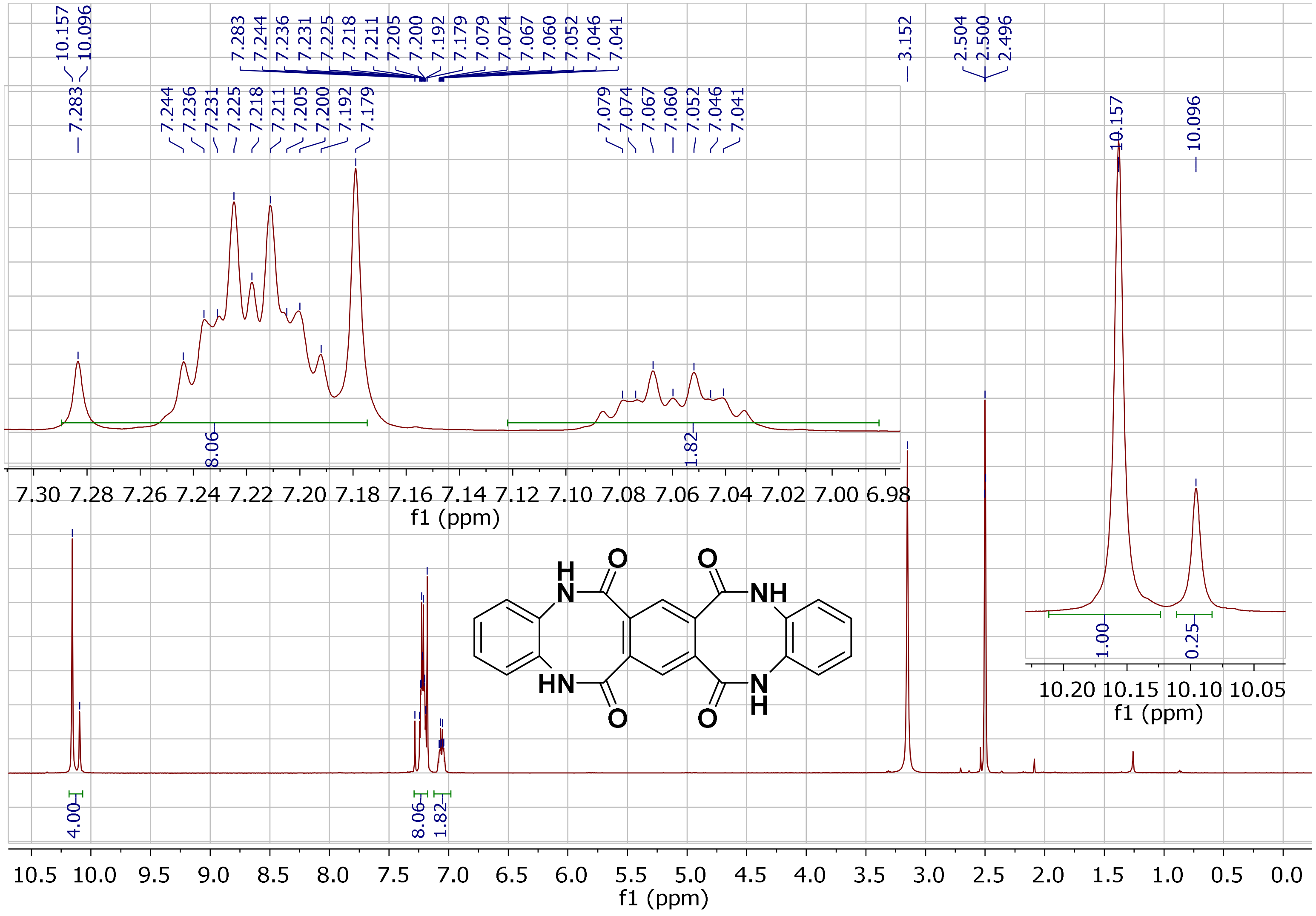

Figure S24. 1H-NMR spectrum for **9a.**

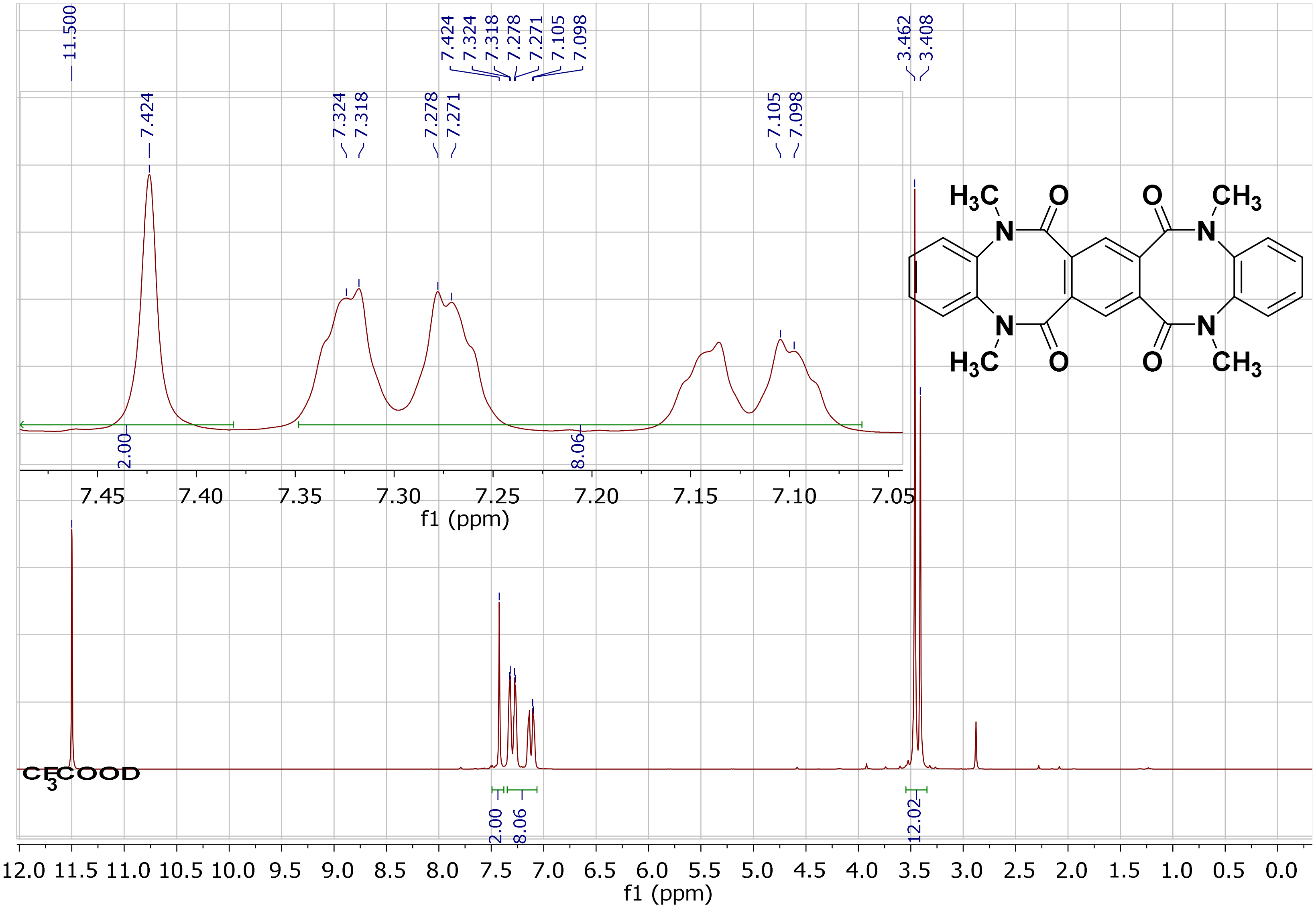

Figure S25a. 1H-NMR spectrum for **9b** (registered in 295 K).

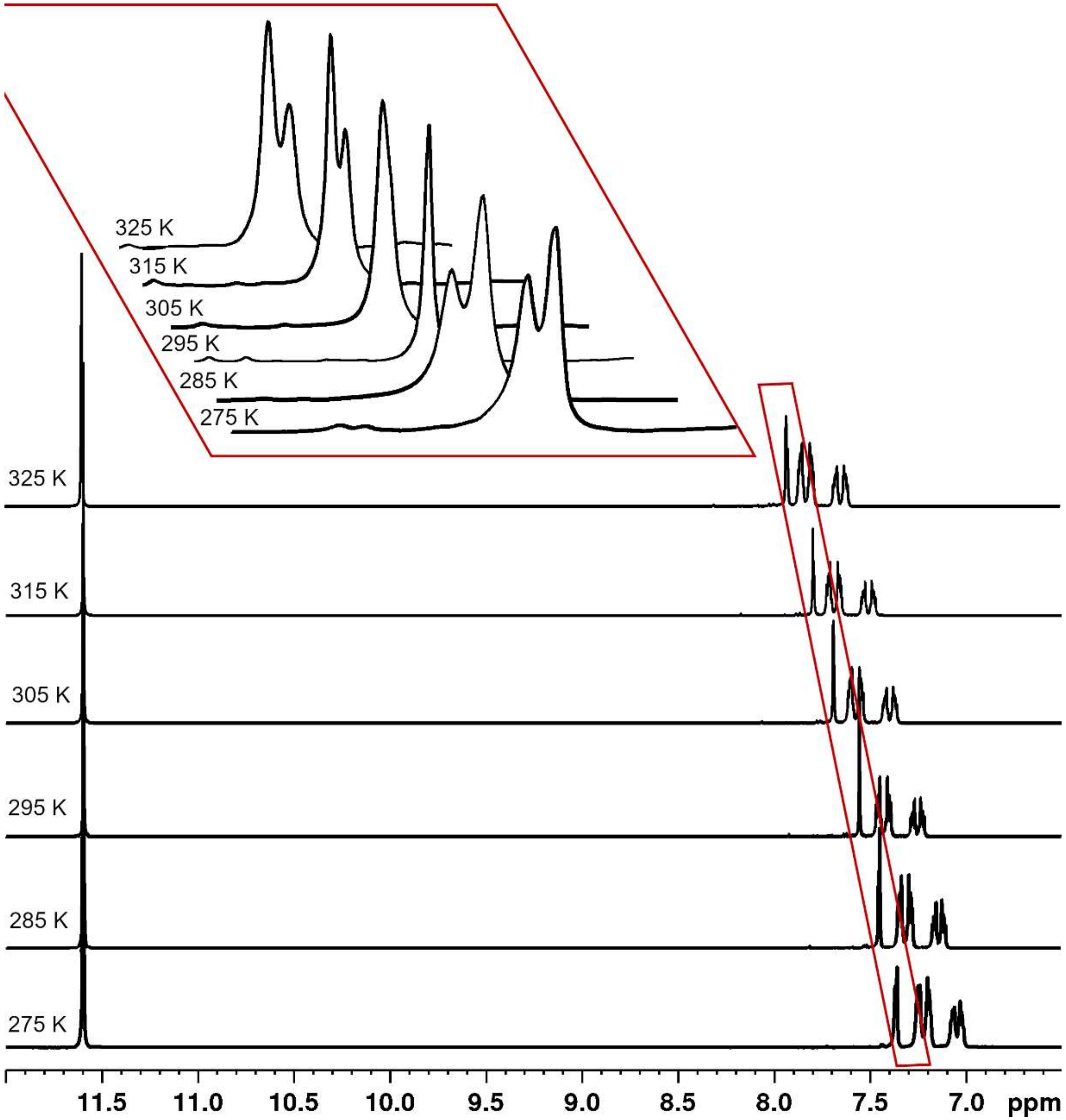

Figure S25b. 1H-NMR spectra for **9b** registered in the temperature range of 275–325 K. The magnified region shows different temperature drift of the two conformers.

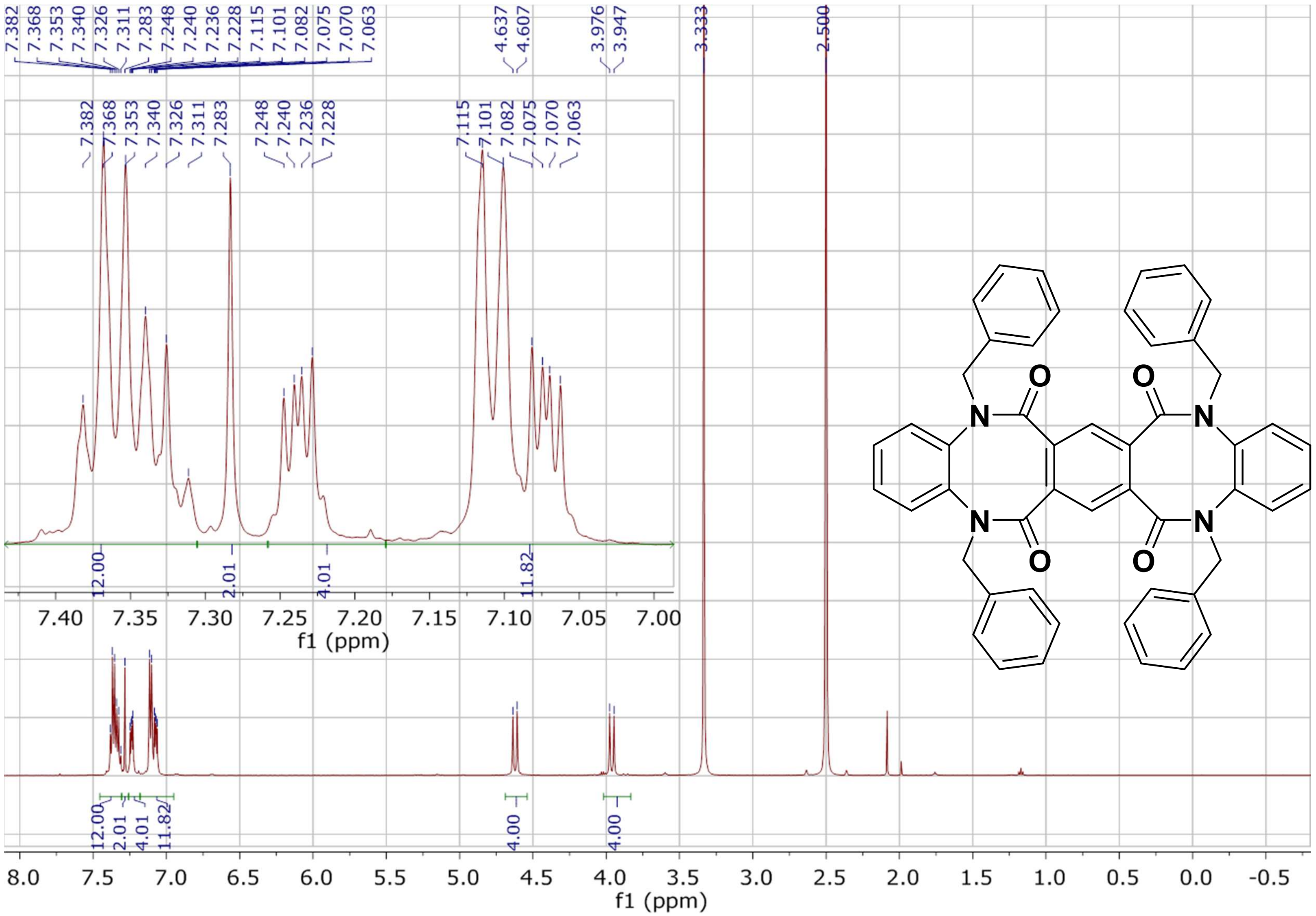

Figure S26. 1H-NMR spectrum for **9c.**

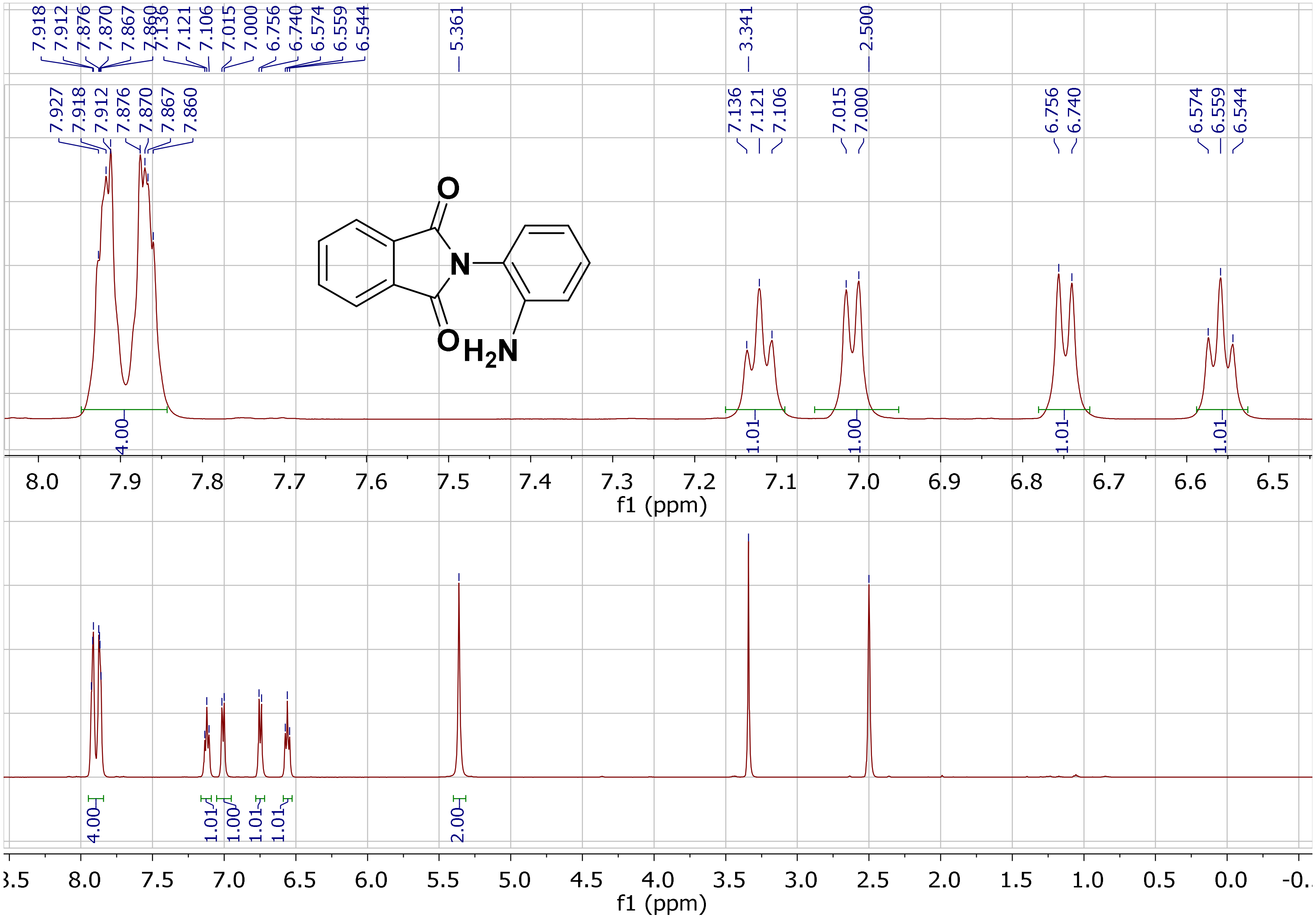

Figure S27. 1H-NMR spectrum for 2-(2-aminophenyl)isoindoline–1,3-dione (**10**).

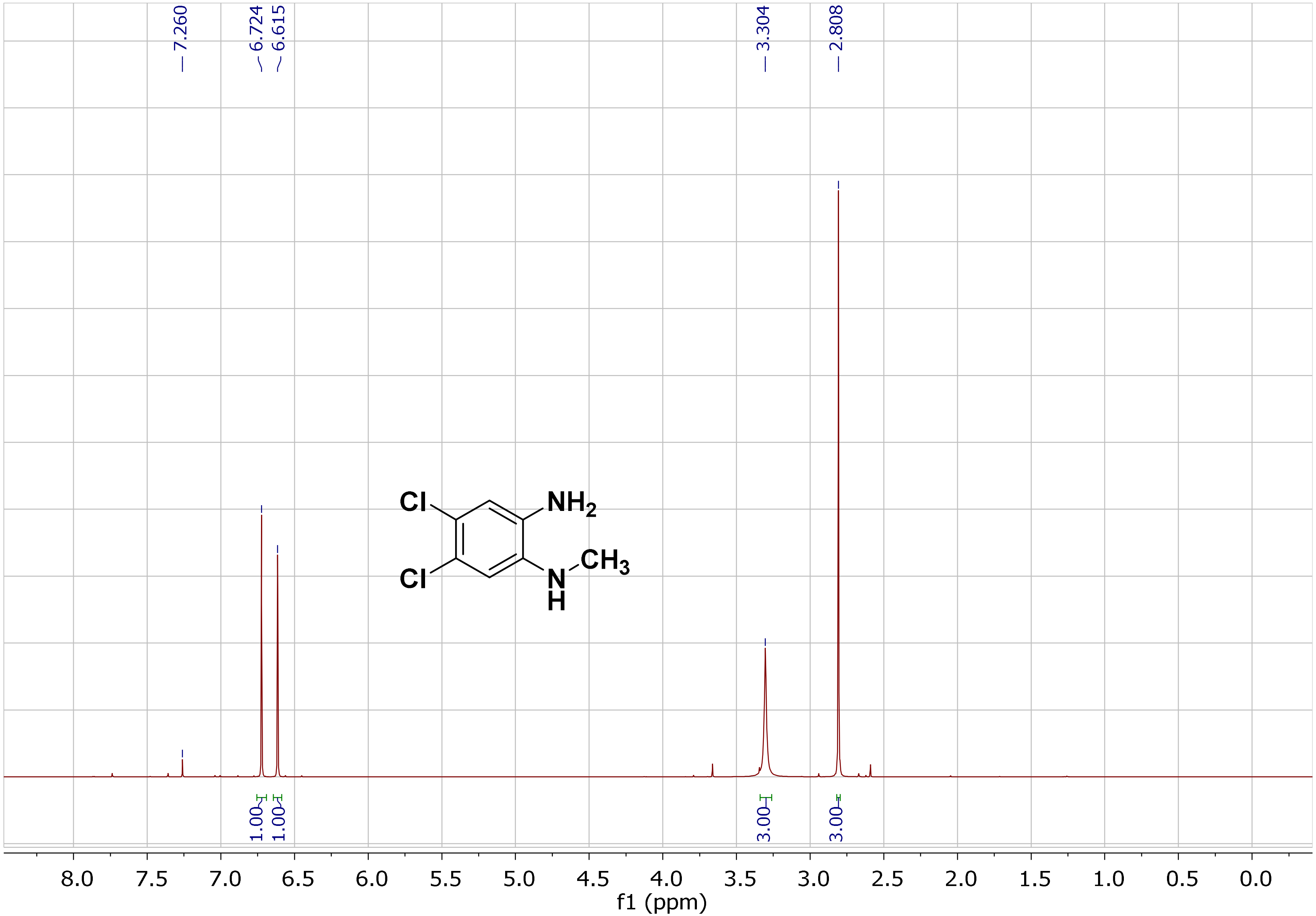

Figure S28. 1H-NMR spectrum for 4,5-dichloro-*N*[1]-methylbenzene–1,2-diamine (**4e**).

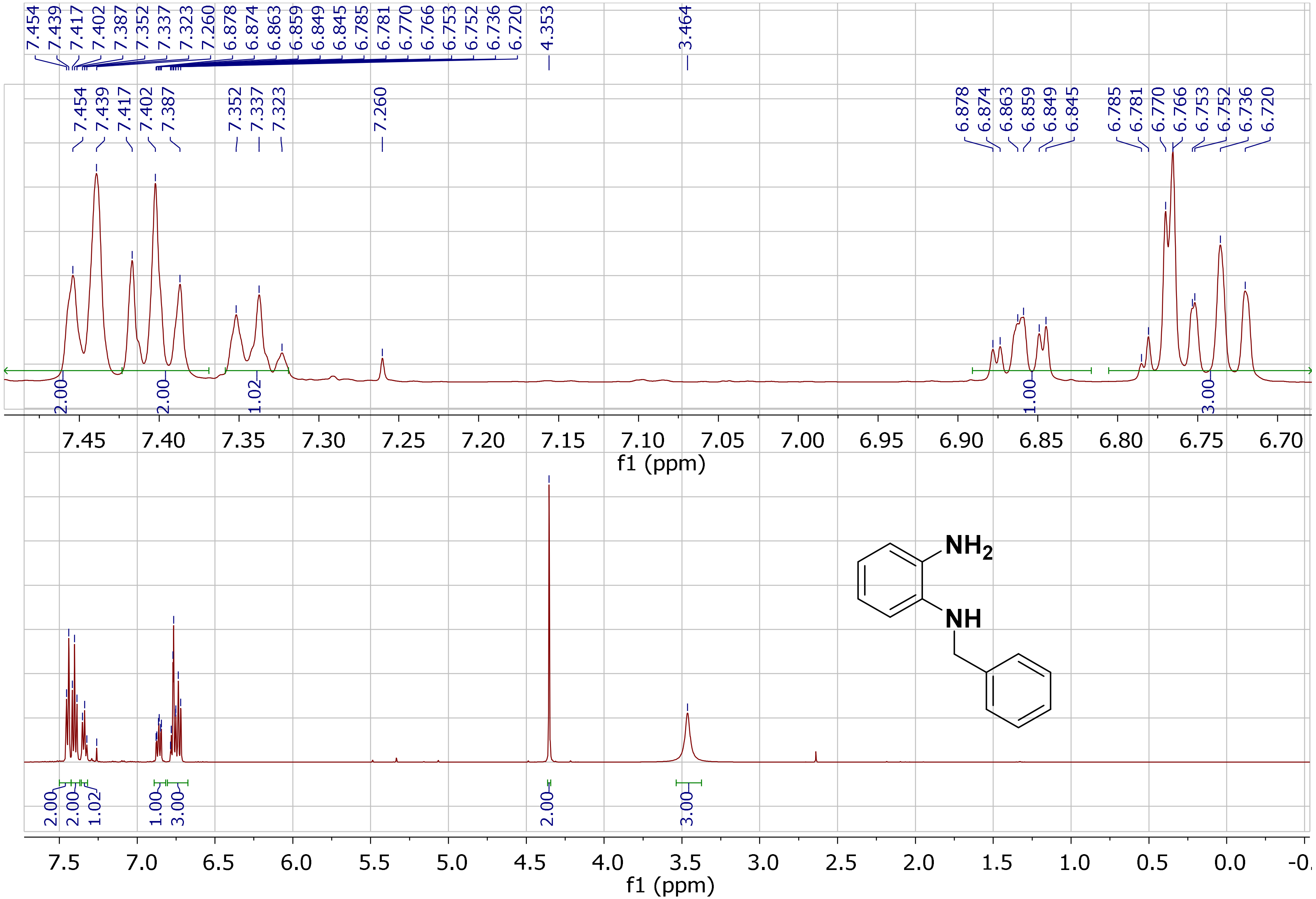

Figure S29. 1H-NMR spectrum for *N*[1]-benzylbenzene–1,2-diamine (**4f**).

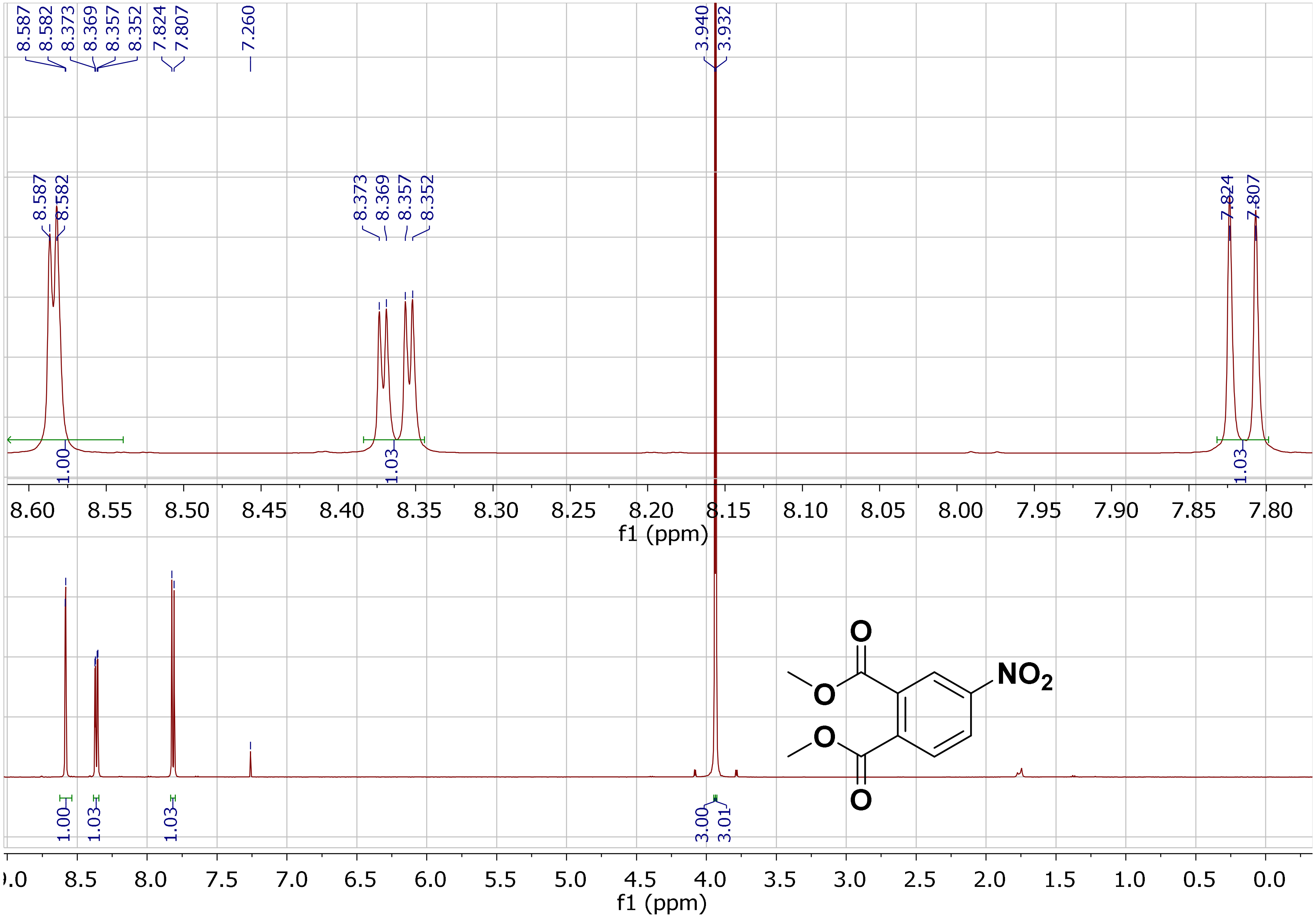

Figure S30. 1H-NMR spectrum for dimethyl 4-nitrophthalate (**5b**).

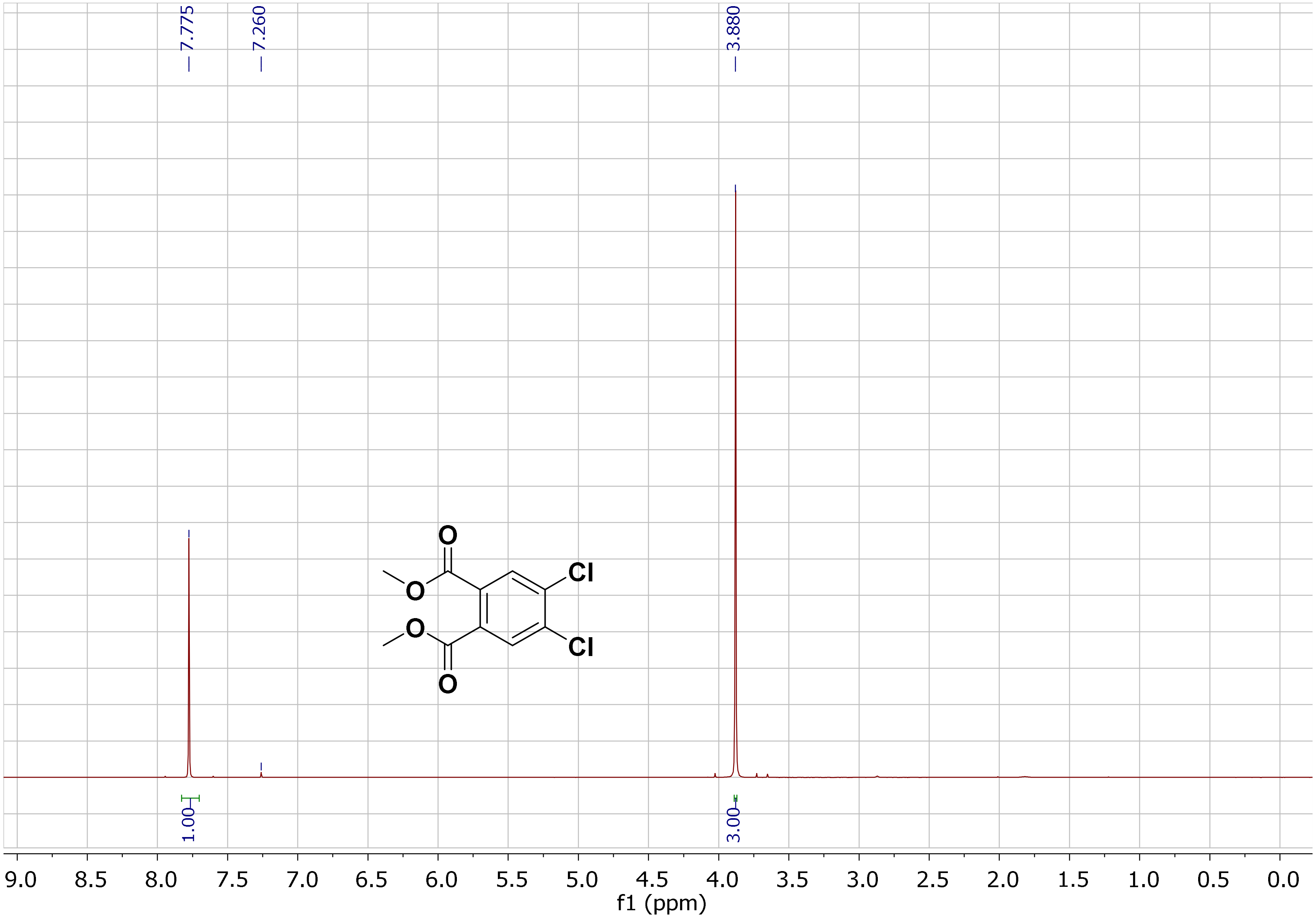

Figure S31. 1H-NMR spectrum for dimethyl 4,5-dichlorophthalate (**5c**).

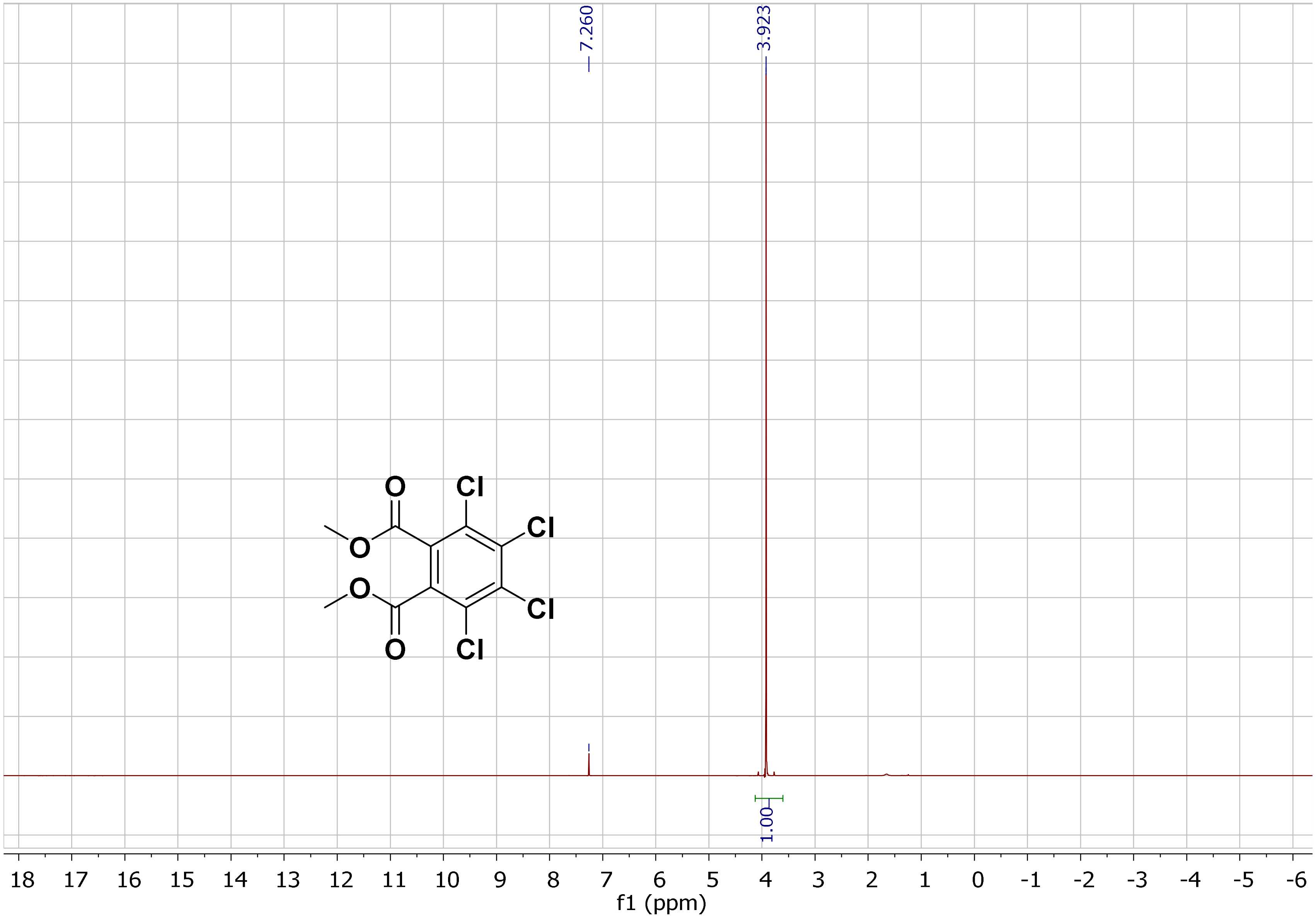

Figure S32. 1H-NMR spectrum for dimethyl 3,4,5,6-tetrachlorophthalate (**5d**).

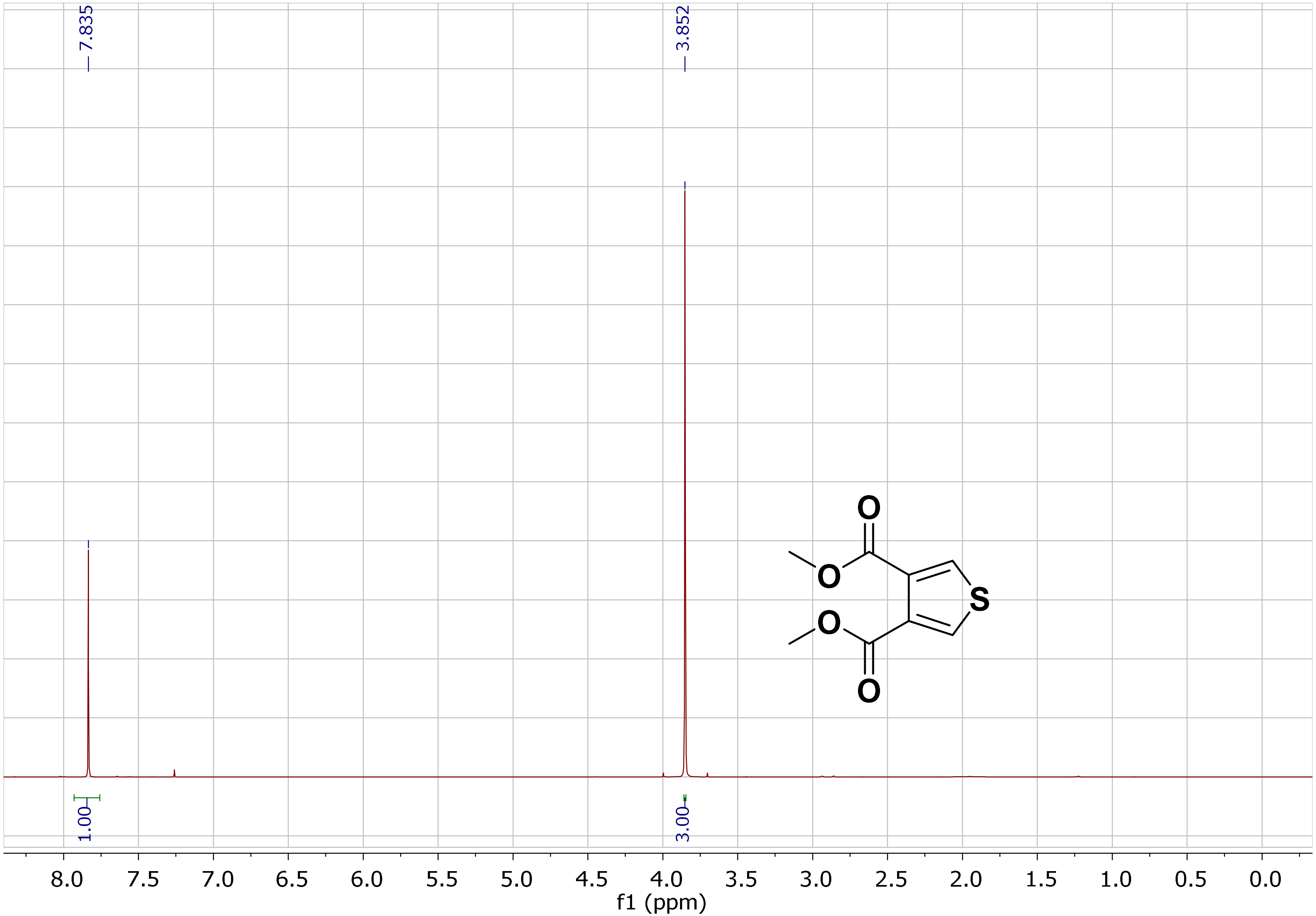

Figure S33. 1H-NMR spectrum for dimethyl thiophene–3,4-dicarboxylate (**5e**).

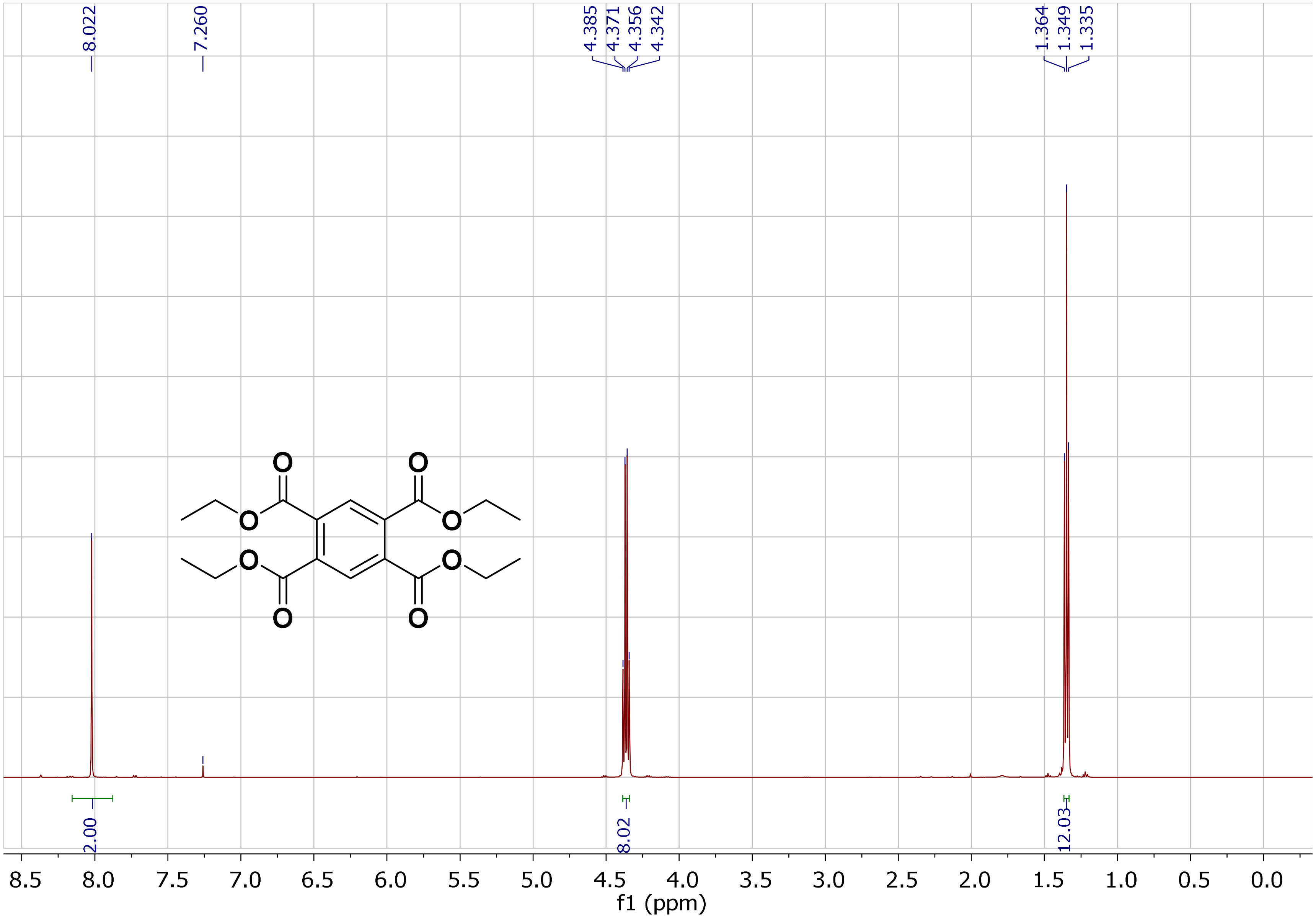

Figure S34. 1H-NMR spectrum for tetraethyl benzene–1,2,4,5-tetracarboxylate (**8**).

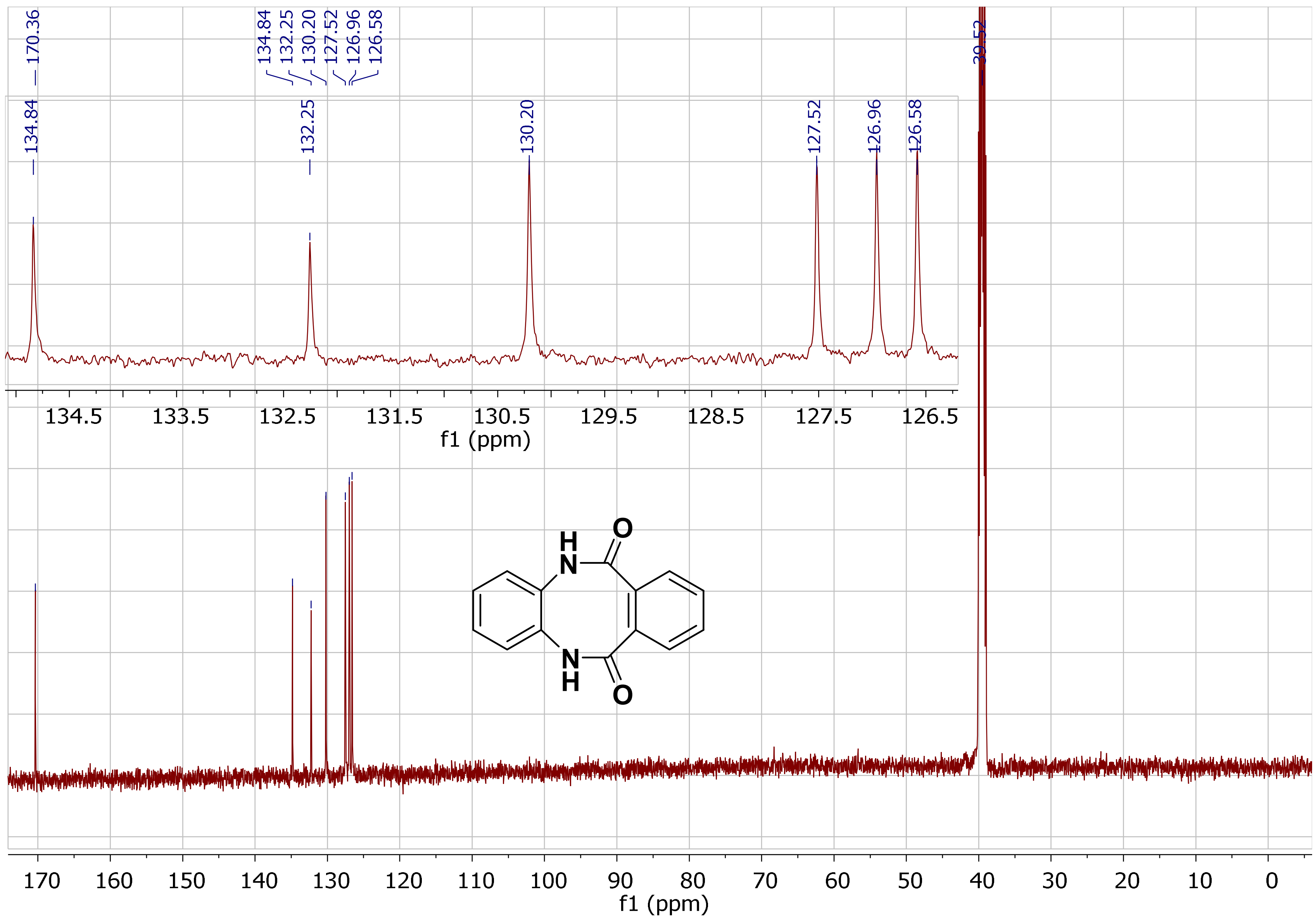

Figure S35. 13C-NMR spectrum for 5,12-dihydrodibenzo[*b*,*f*][1,4]diazocine–6,11-dione (**3a**).

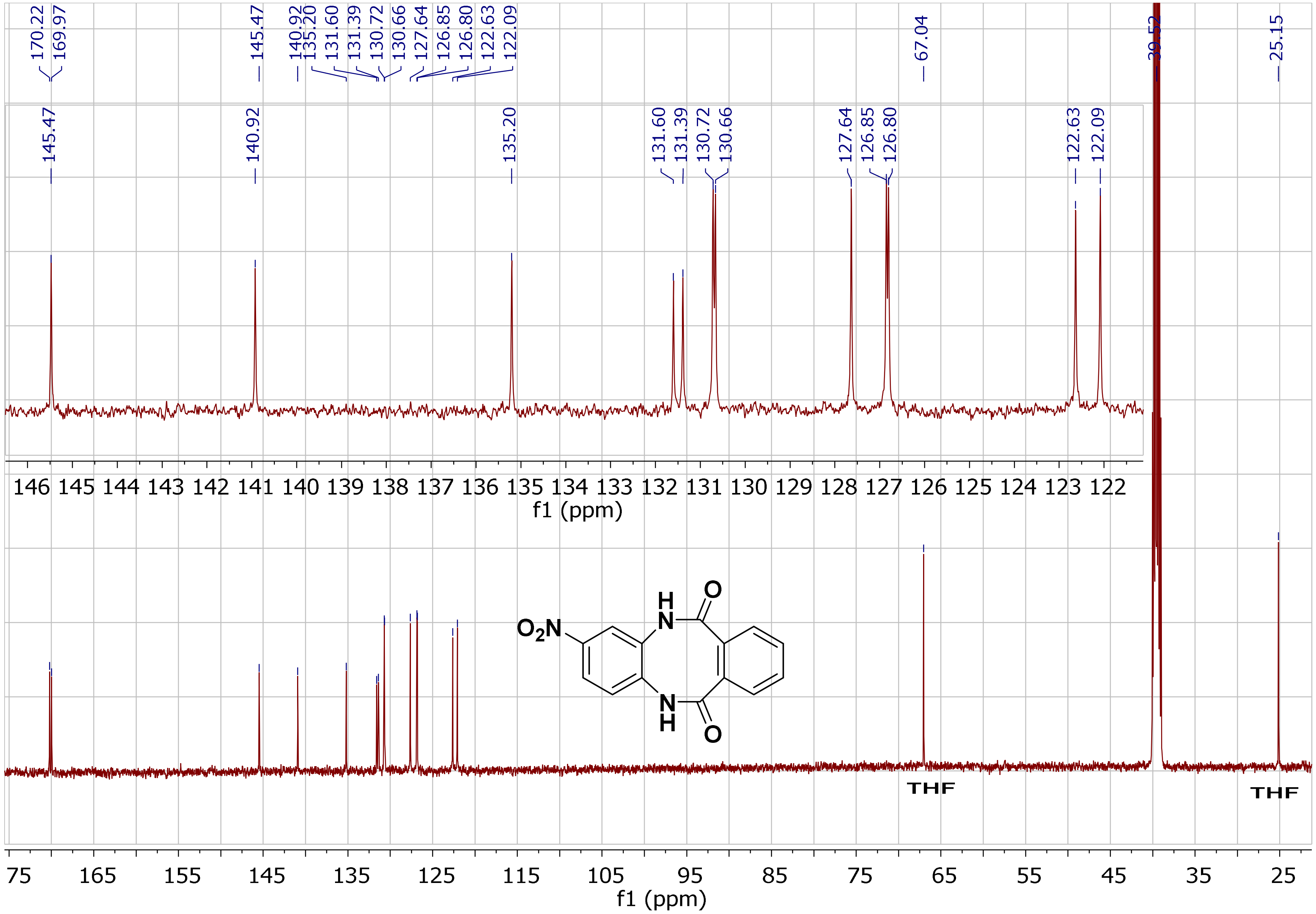

Figure S36. 13C-NMR spectrum for 2-nitro–5,12-dihydrodibenzo[*b*,*f*][1,4]diazocine–6,11-dione (**3b**).

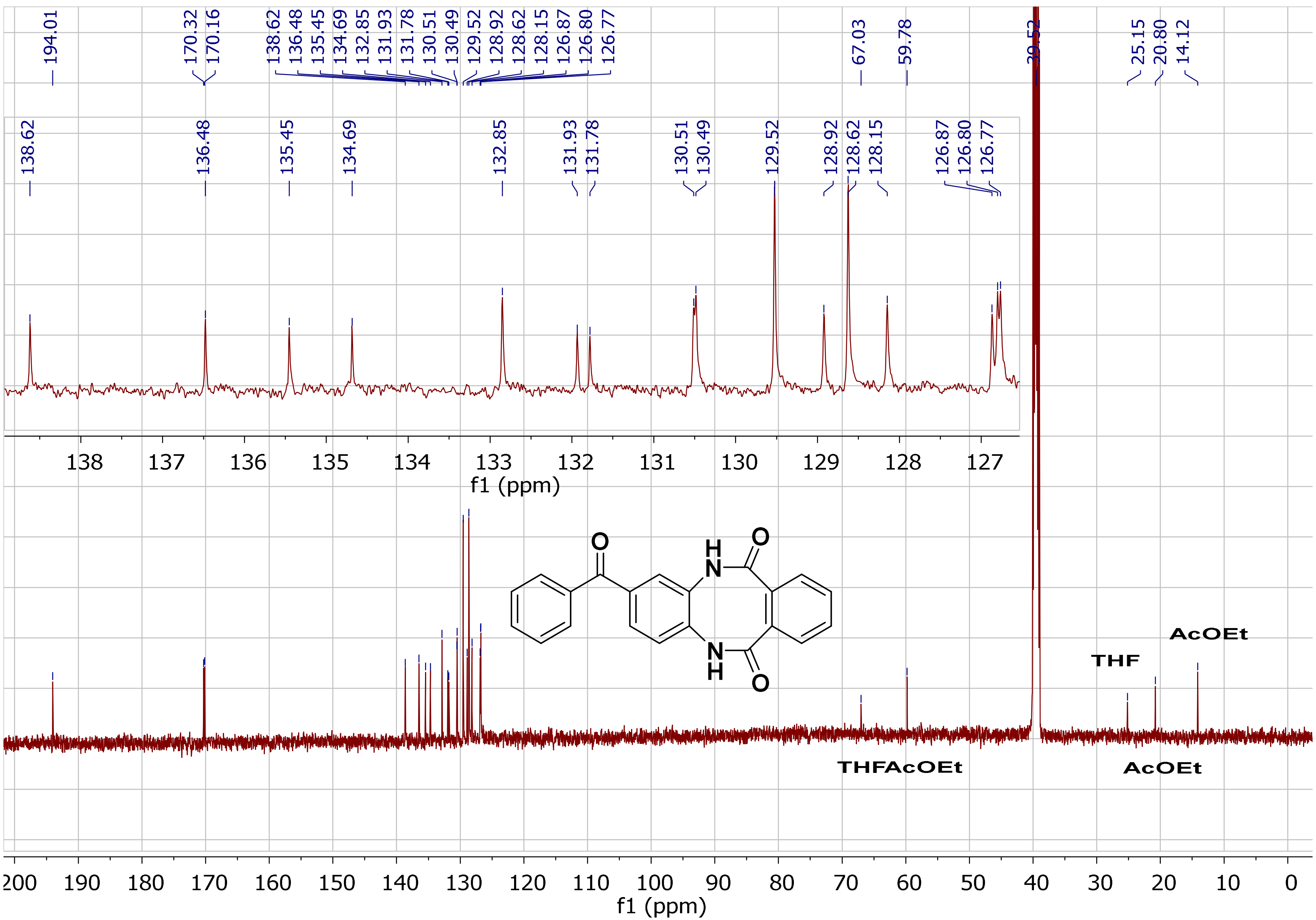

Figure S37. 13C-NMR spectrum for 2-benzoyl–5,12-dihydrodibenzo[*b*,*f*][1,4]diazocine–6,11-dione (**3c**).

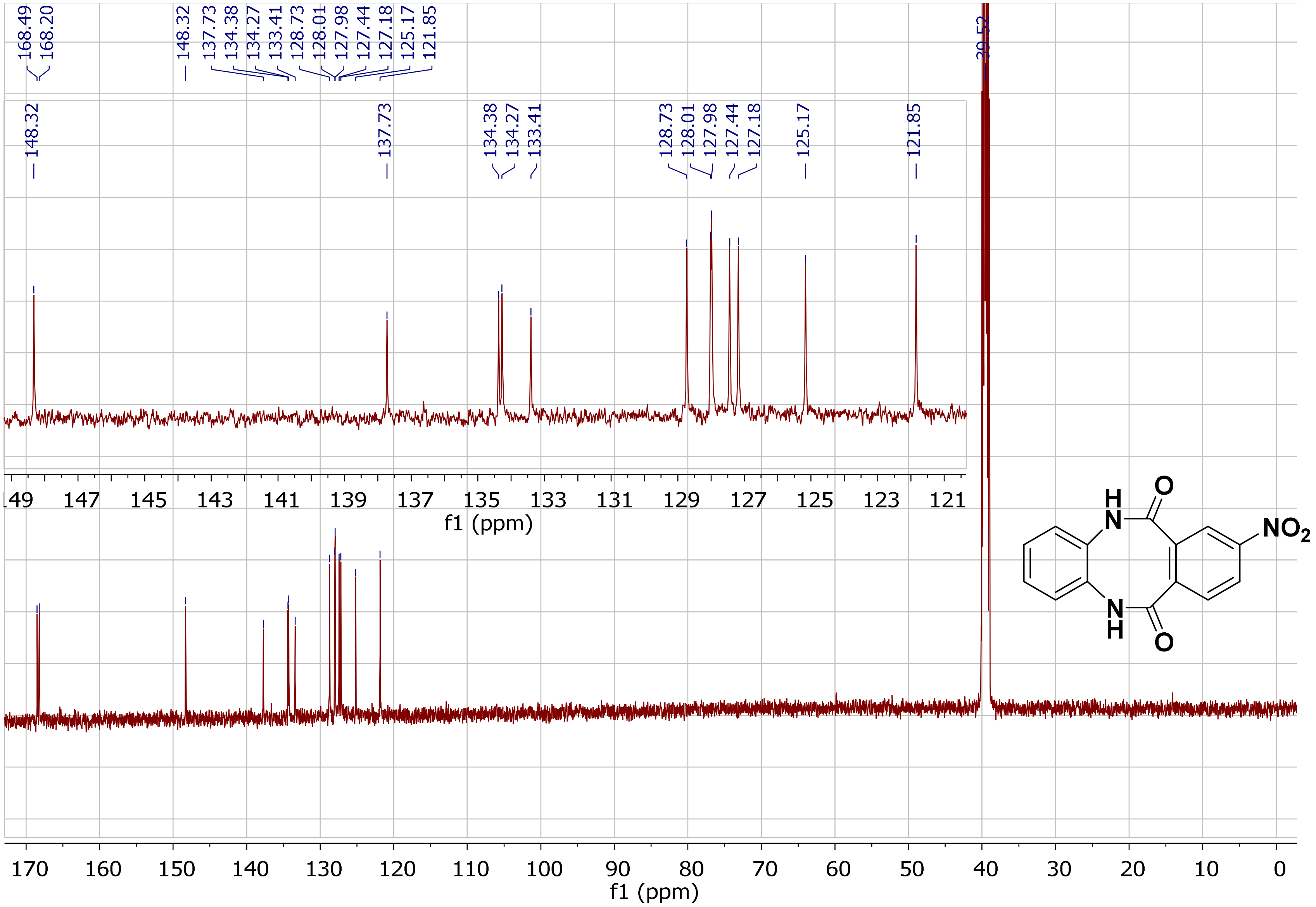

Figure S38. 13C-NMR spectrum for 8-nitro–5,12-dihydrodibenzo[*b*,*f*][1,4]diazocine–6,11-dione (**3d**).

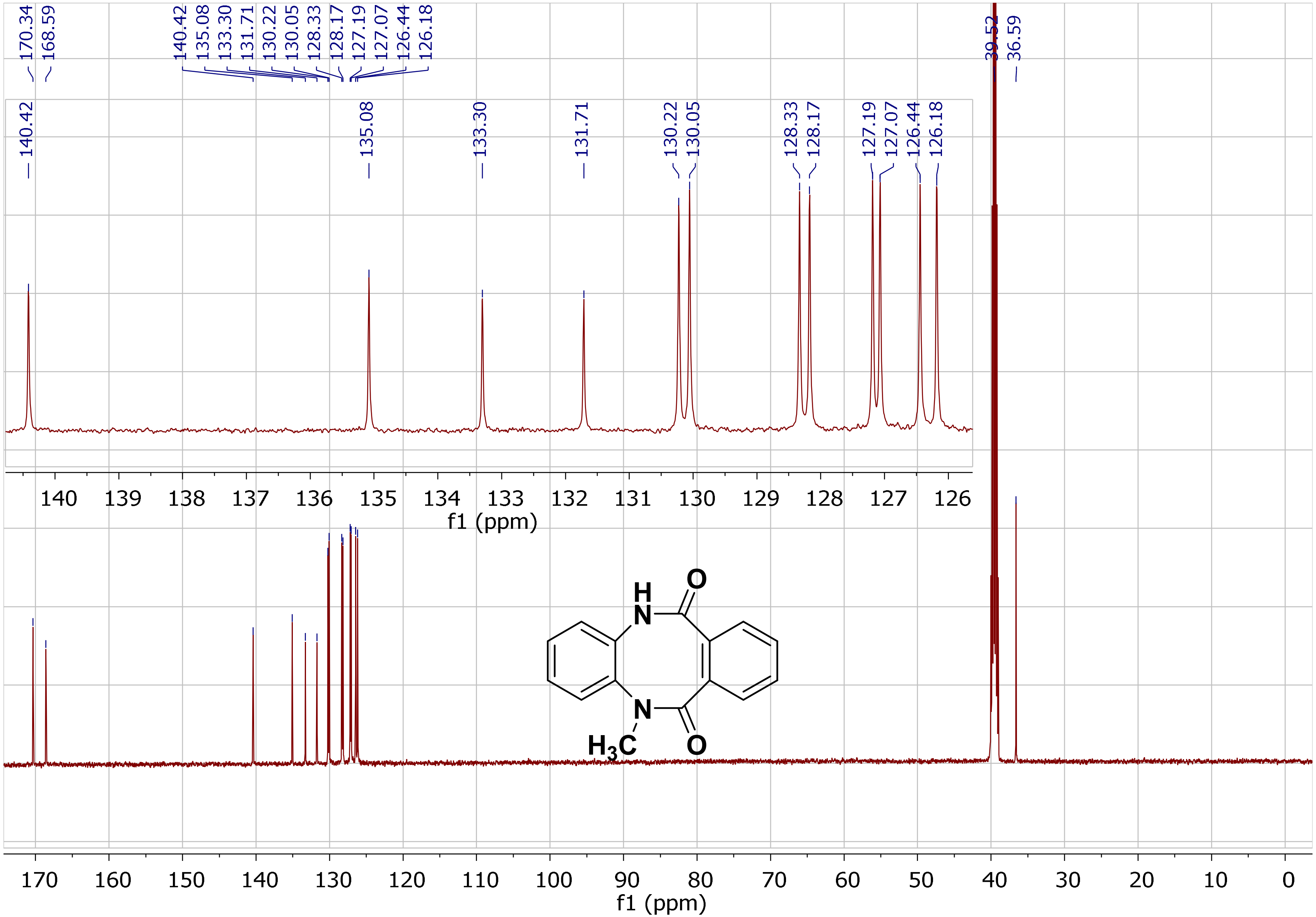

Figure S39. 13C-NMR spectrum for 5-methyl–5,12-dihydrodibenzo[*b*,*f*][1,4]diazocine–6,11-dione (**3e**).

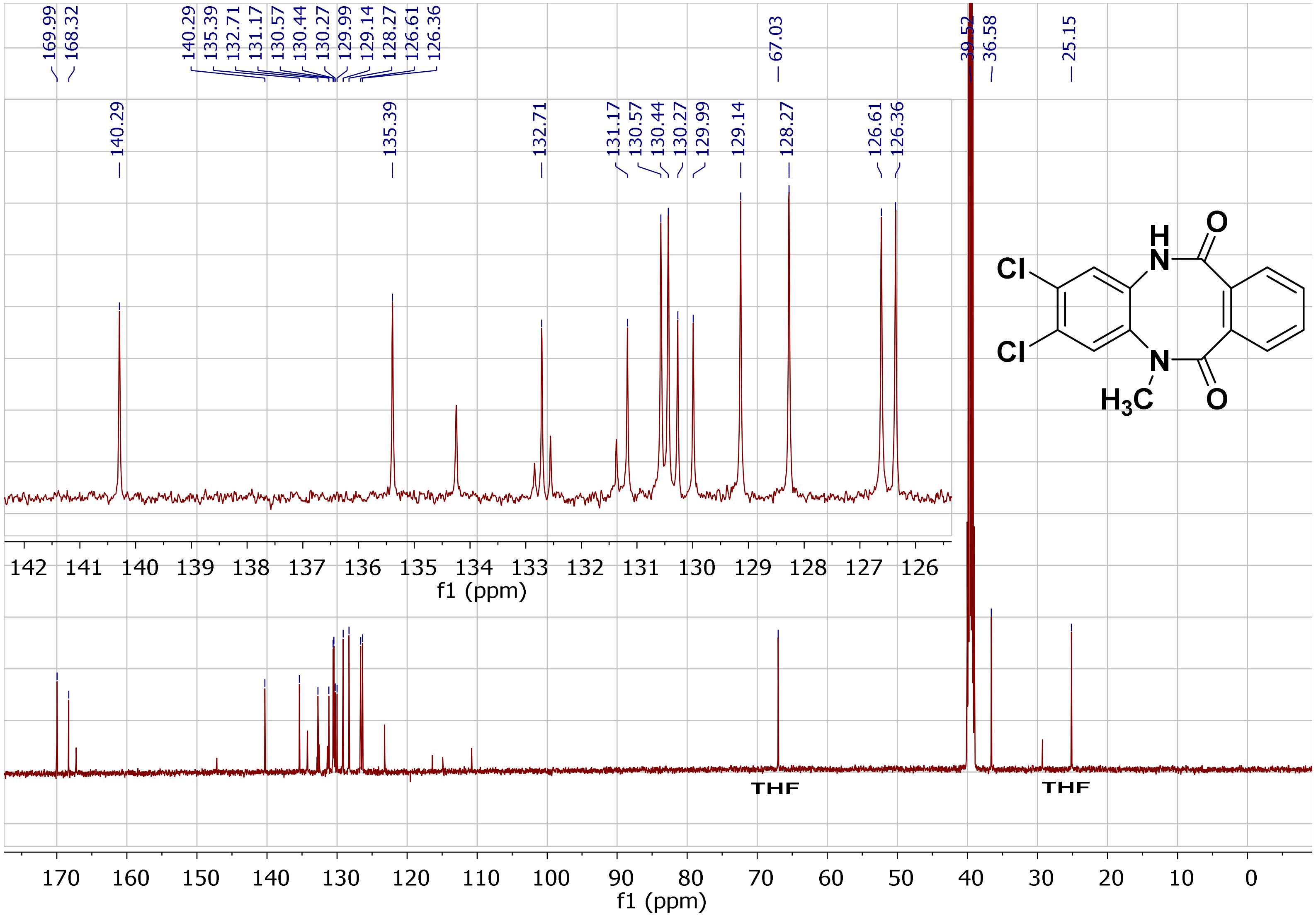

Figure S40. 13C-NMR spectrum for 2,3-dichloro–5-methyl–5,12-dihydrodibenzo[*b*,*f*][1,4]diazocine–6,11-dione (**3f**).

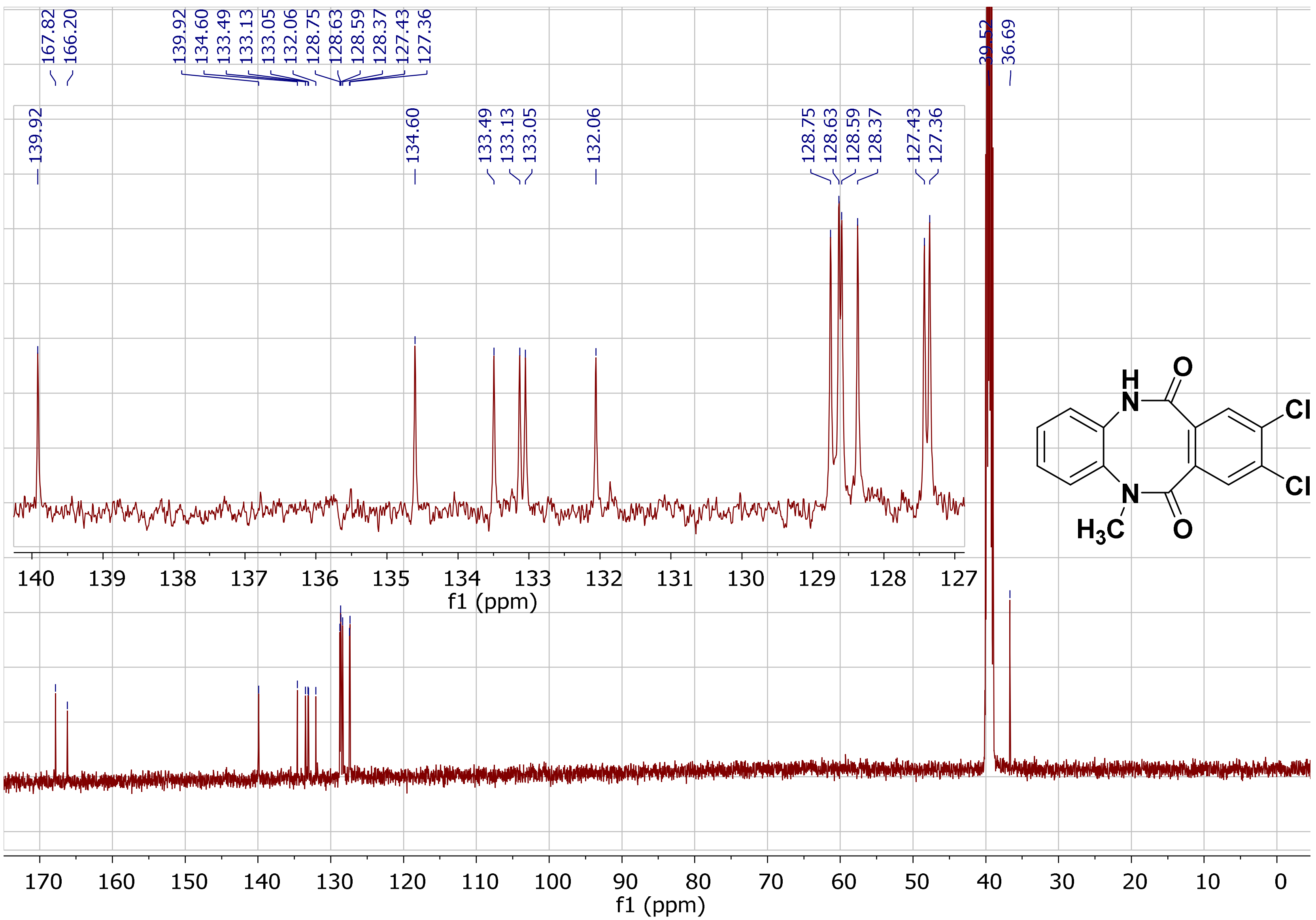

Figure S41. 13C-NMR spectrum for 8,9-dichloro–5-methyl–5,12-dihydrodibenzo[*b*,*f*][1,4]diazocine–6,11-dione (**3g**).

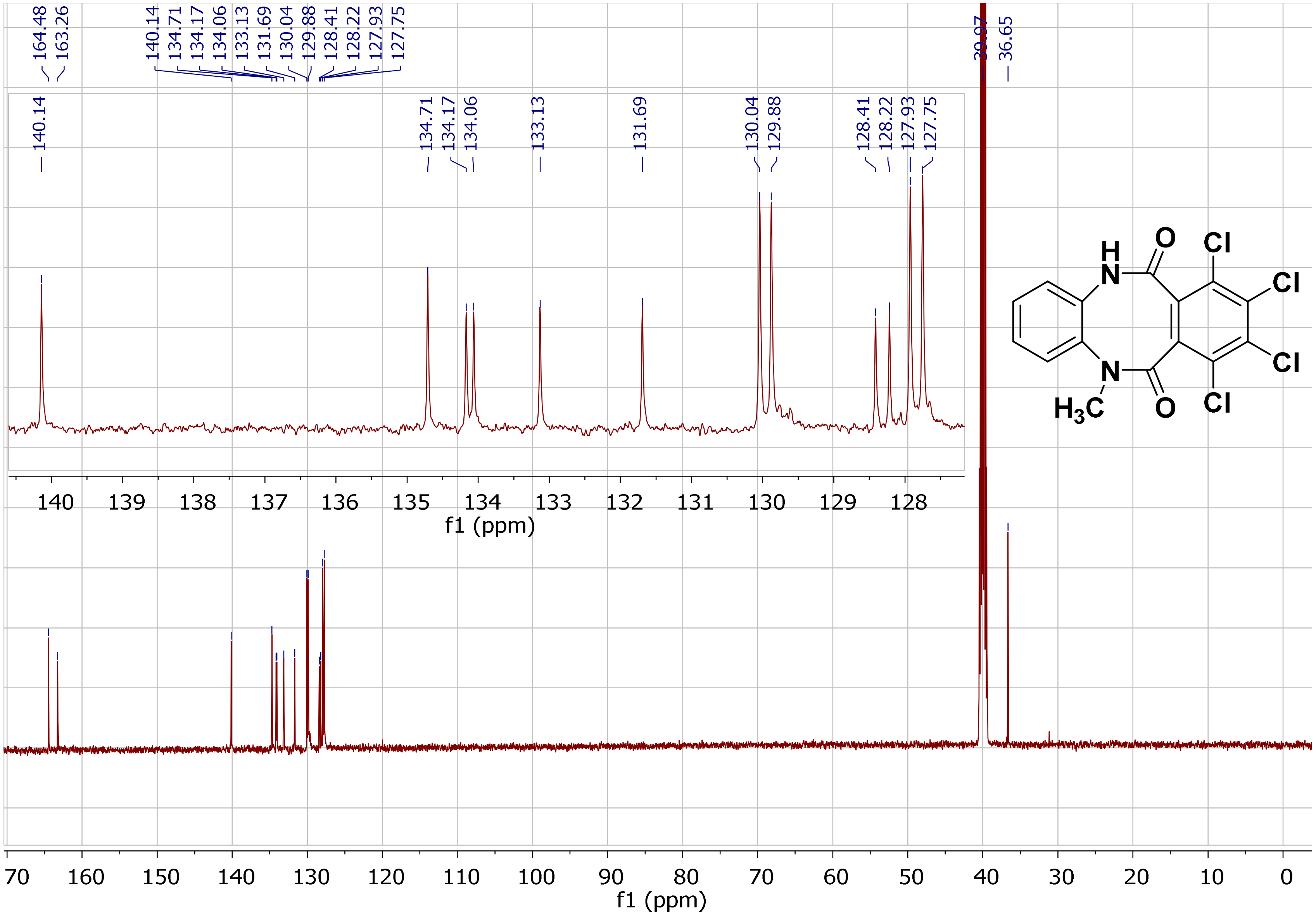

Figure S42. 13C-NMR spectrum for 7,8,9,10-tetrachloro–5-methyl–5,12-dihydrodibenzo[*b*,*f*][1,4]diazocine–6,11-dione (**3h**).

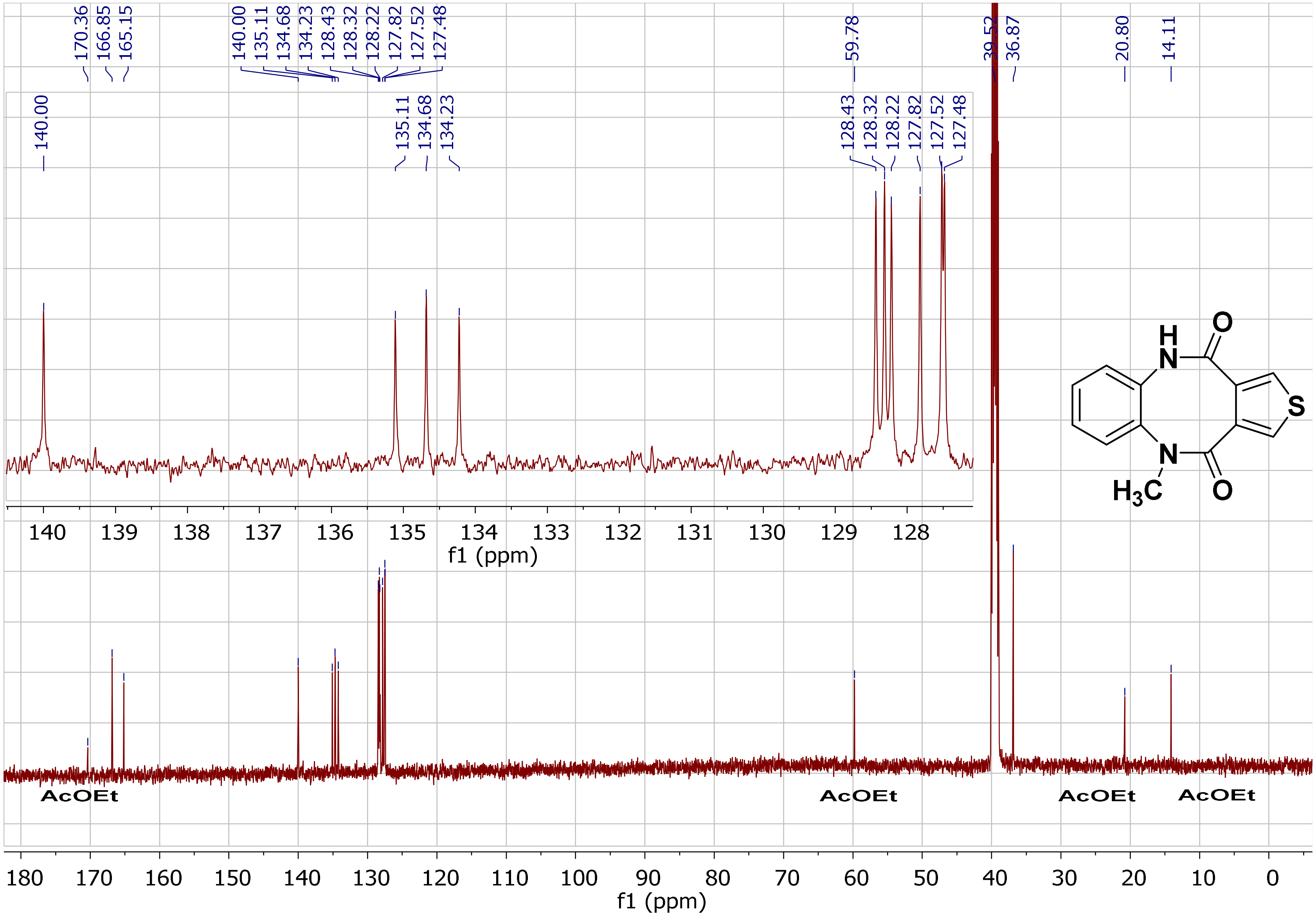

Figure S43. 13C-NMR spectrum for 5-methyl–5,10-dihydrobenzo[*b*]thieno[3,4-*f*][1,4]diazocine–4,11-dione (**3i**).

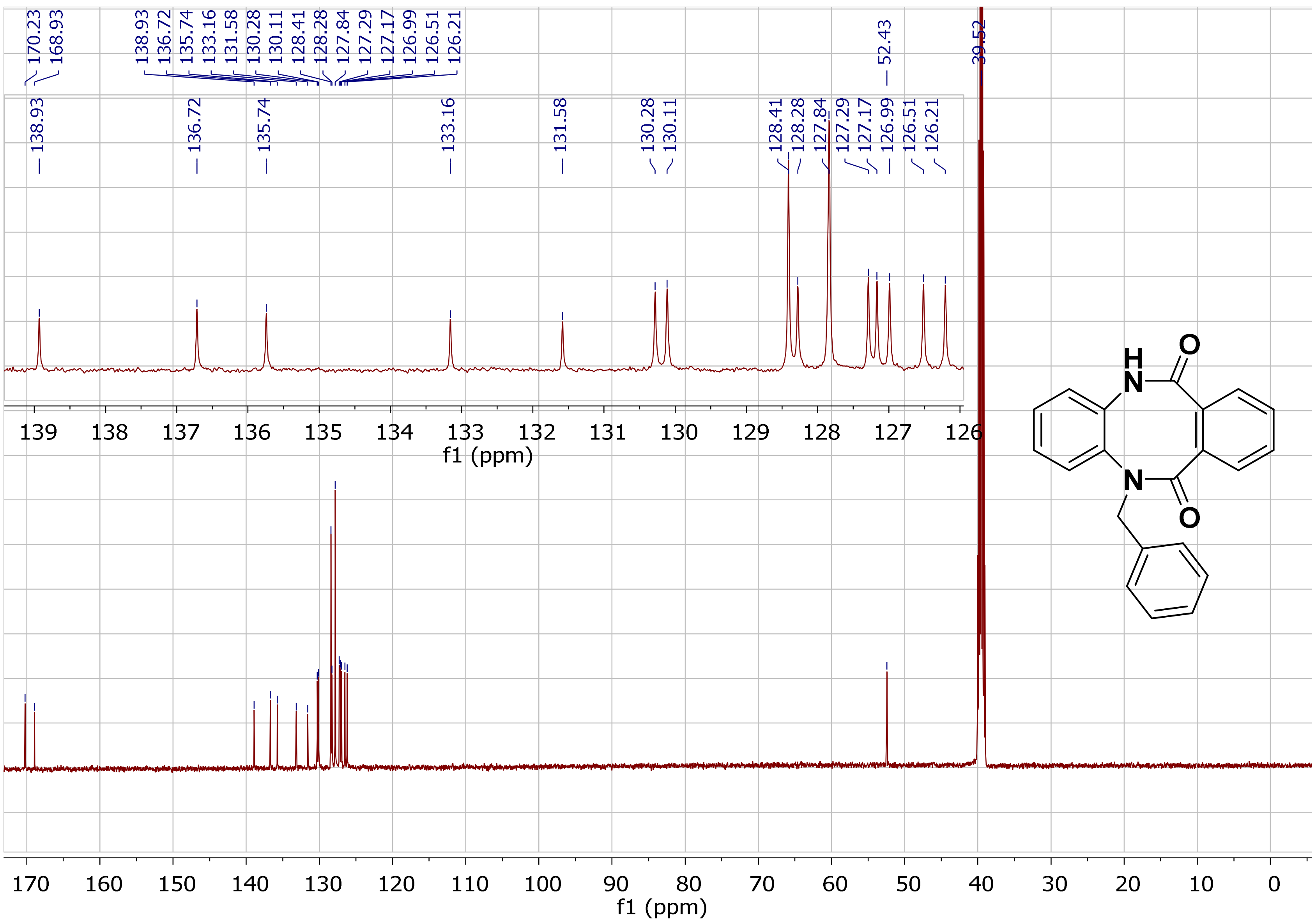

Figure S44. 13C-NMR spectrum for 5-benzyl–5,12-dihydrodibenzo[*b*,*f*][1,4]diazocine–6,11-dione (**3j**).

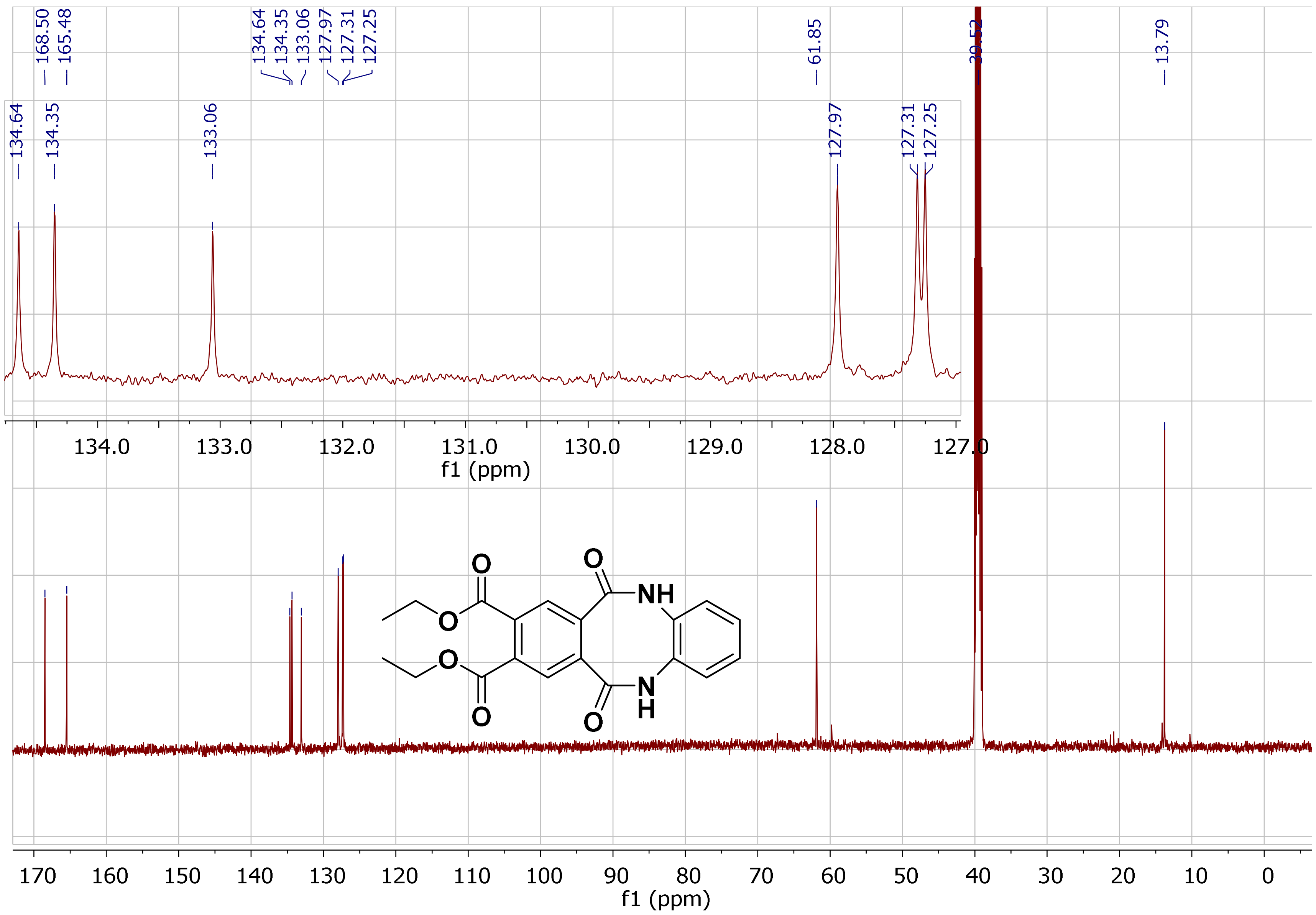

Figure S45. 13C-NMR spectrum for diethyl 6,11-dioxo–5,6,11,12-tetrahydrodibenzo[*b*,*f*][1,4]diazocine–8,9-dicarboxylate (**3k**).

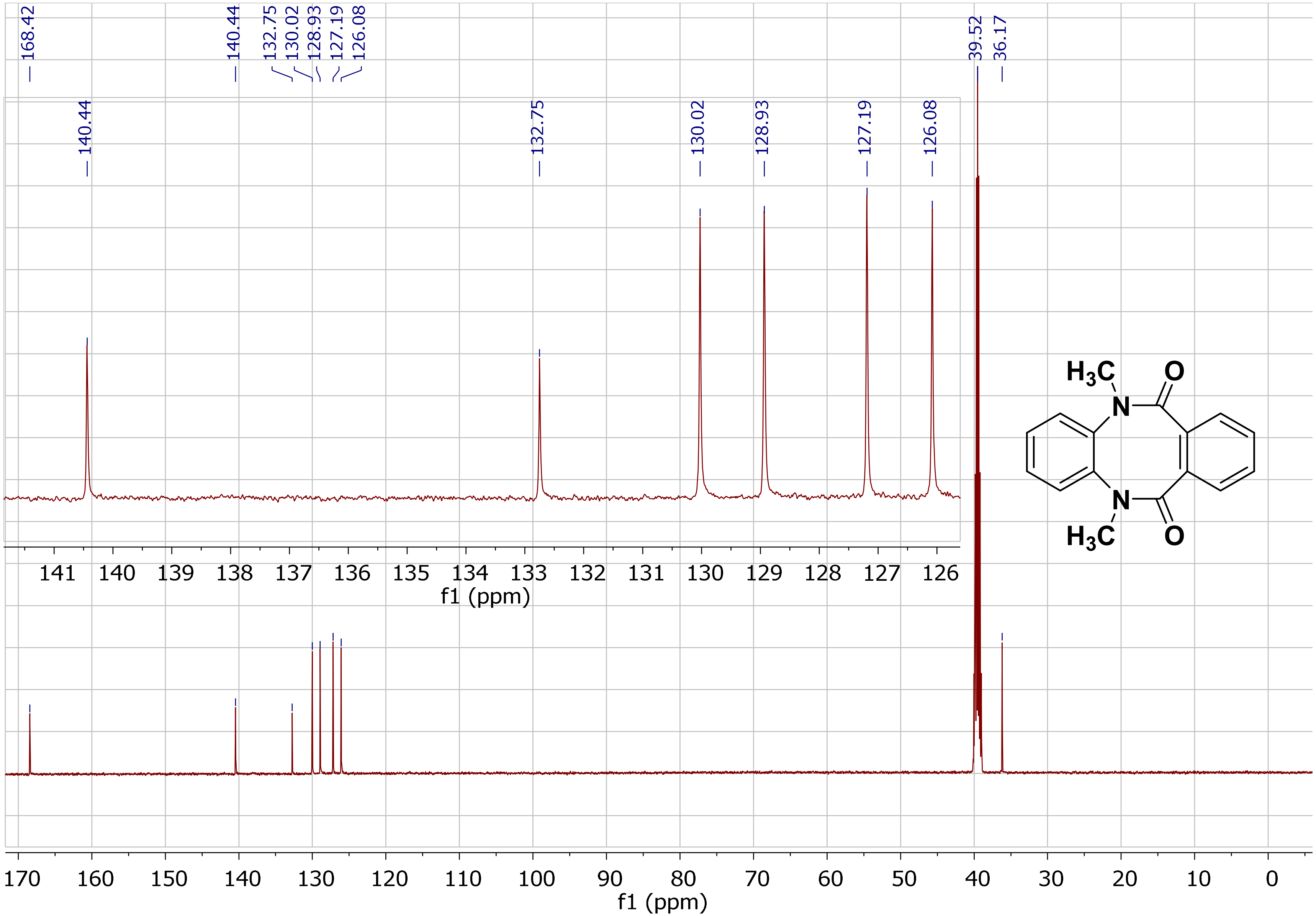

Figure S46. 13C-NMR spectrum for 5,12-dimethyl–5,12-dihydrodibenzo[*b*,*f*][1,4]diazocine–6,11-dione (**3l**).

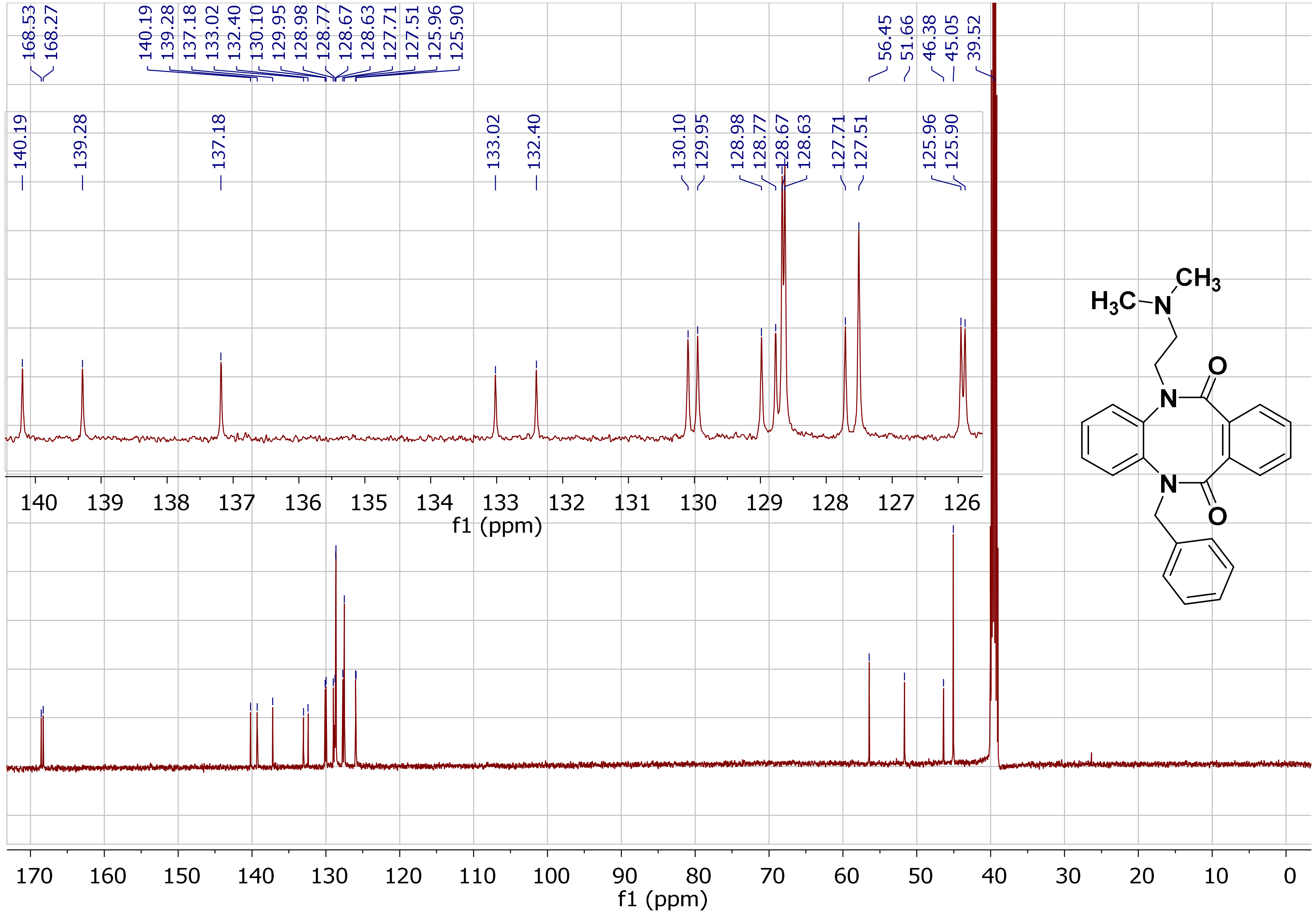

Figure S47. 13C-NMR spectrum for 5-benzyl–12-(2-(dimethylamino)ethyl)–5,12-dihydrodibenzo[*b*,*f*][1,4]diazocine–6,11-dione (**3m**).

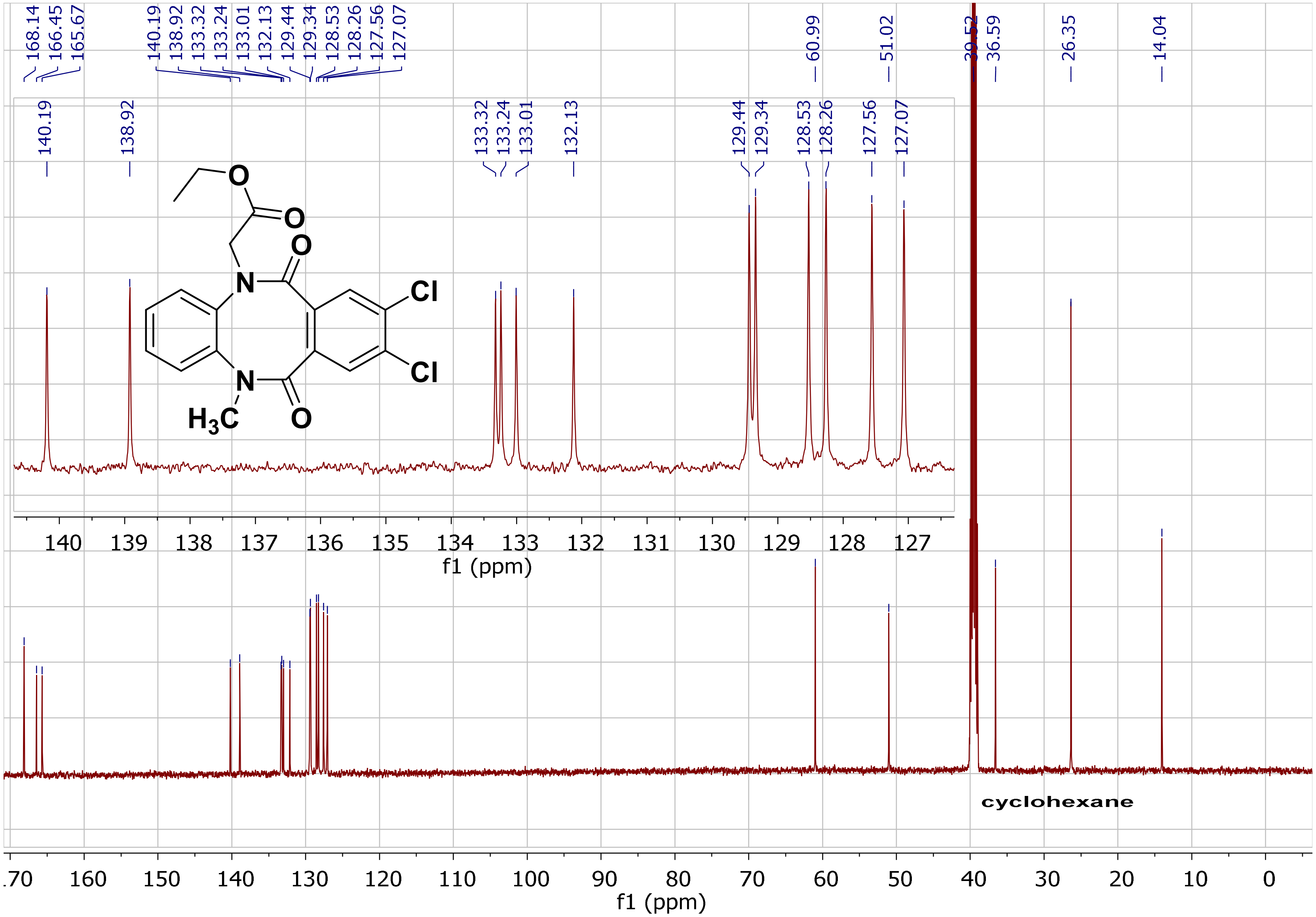

Figure S48. 13C-NMR spectrum for ethyl 2-(8,9-dichloro–12-methyl–6,11-dioxo–11,12-dihydrodibenzo[*b,f*][1,4]diazocin–5(6*H*)-yl)acetate (**3n**).

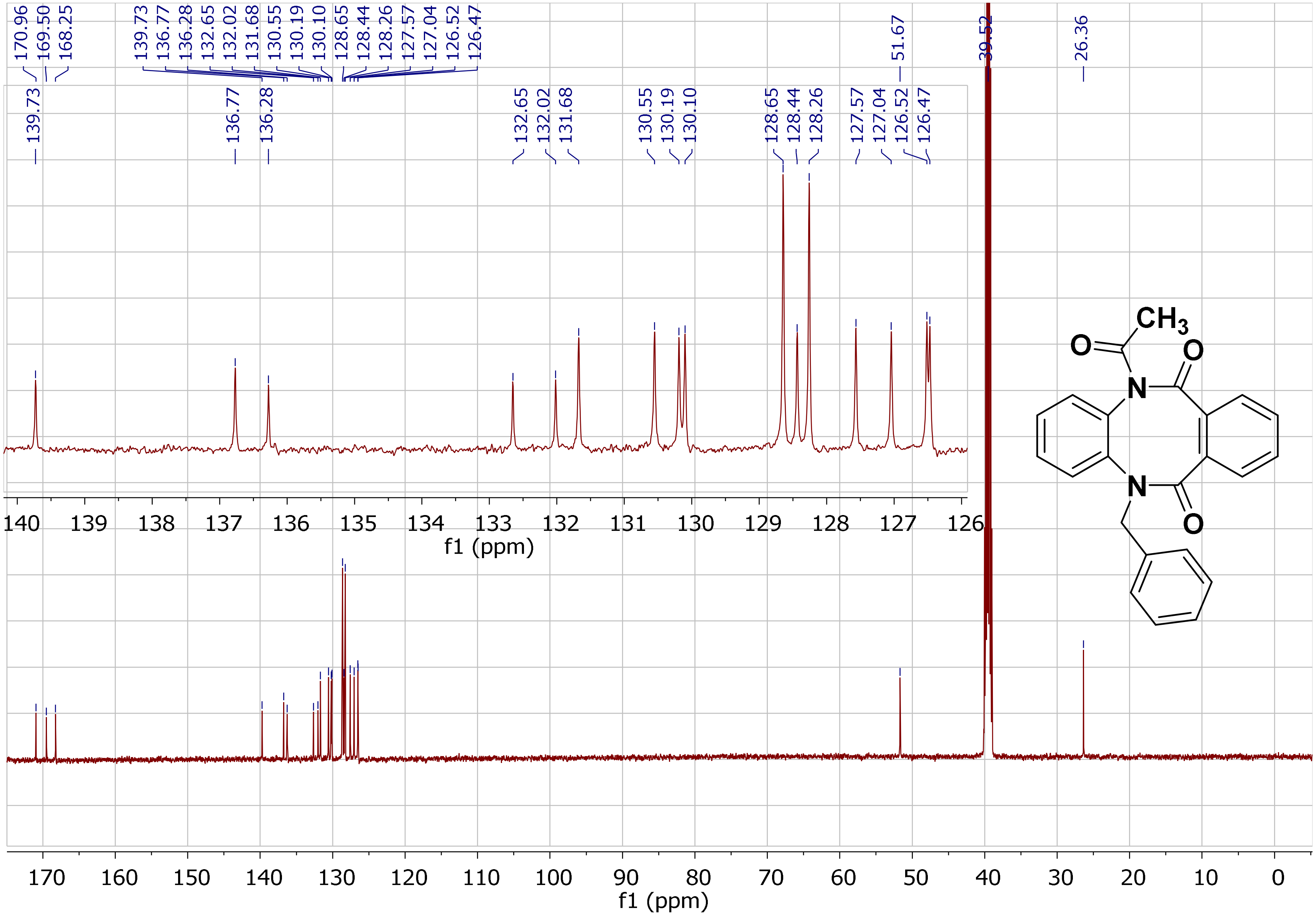

Figure S49. 13C-NMR spectrum for 5-acetyl–12-benzyl–5,12-dihydrodibenzo[*b*,*f*][1,4]diazocine–6,11-dione (**3o**).

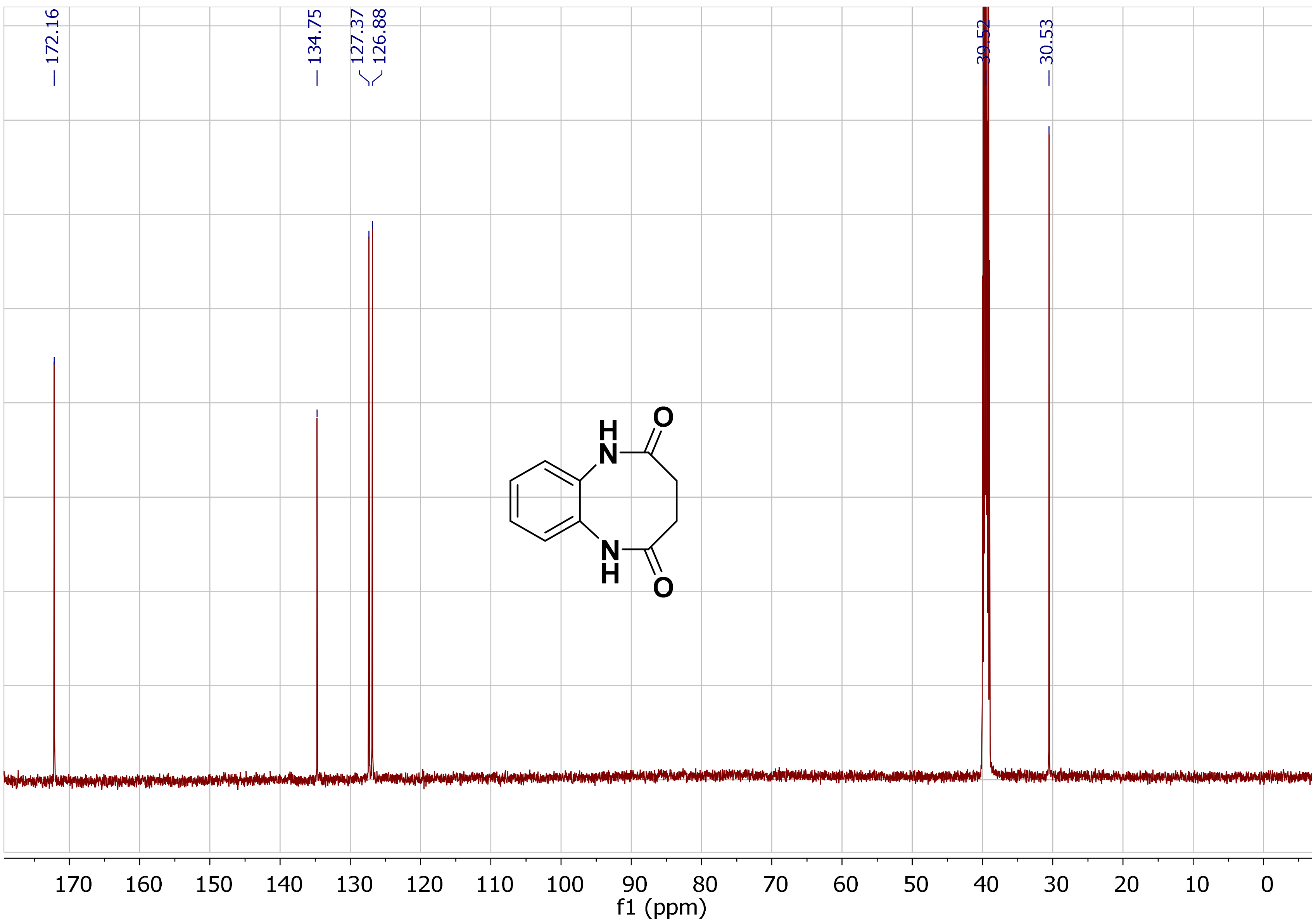

Figure S50. 13C-NMR spectrum for 1,3,4,6-tetrahydrobenzo[*b*][1,4]diazocine–2,5-dione (**6**).

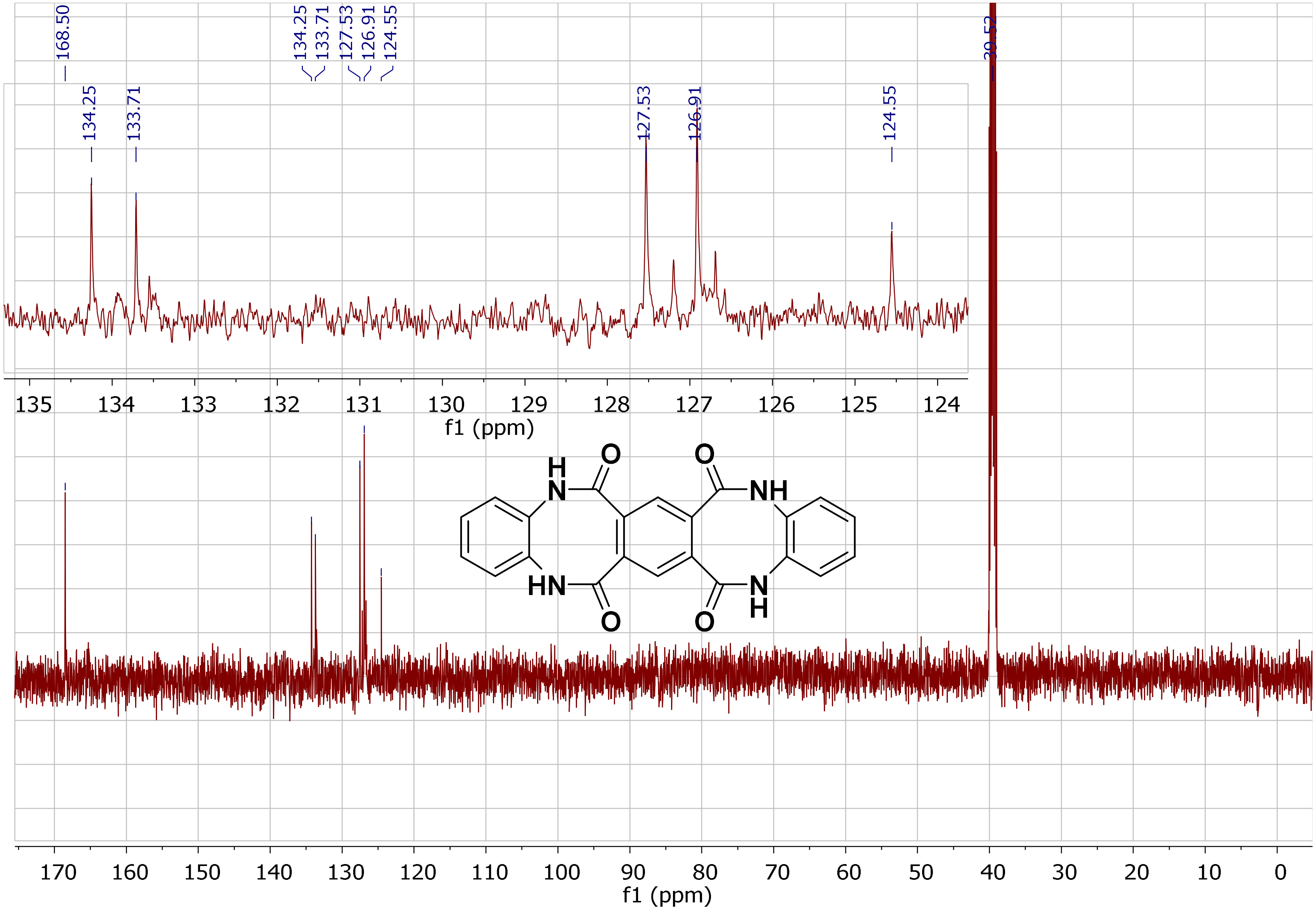

Figure S51. 13C-NMR spectrum for **9a.**

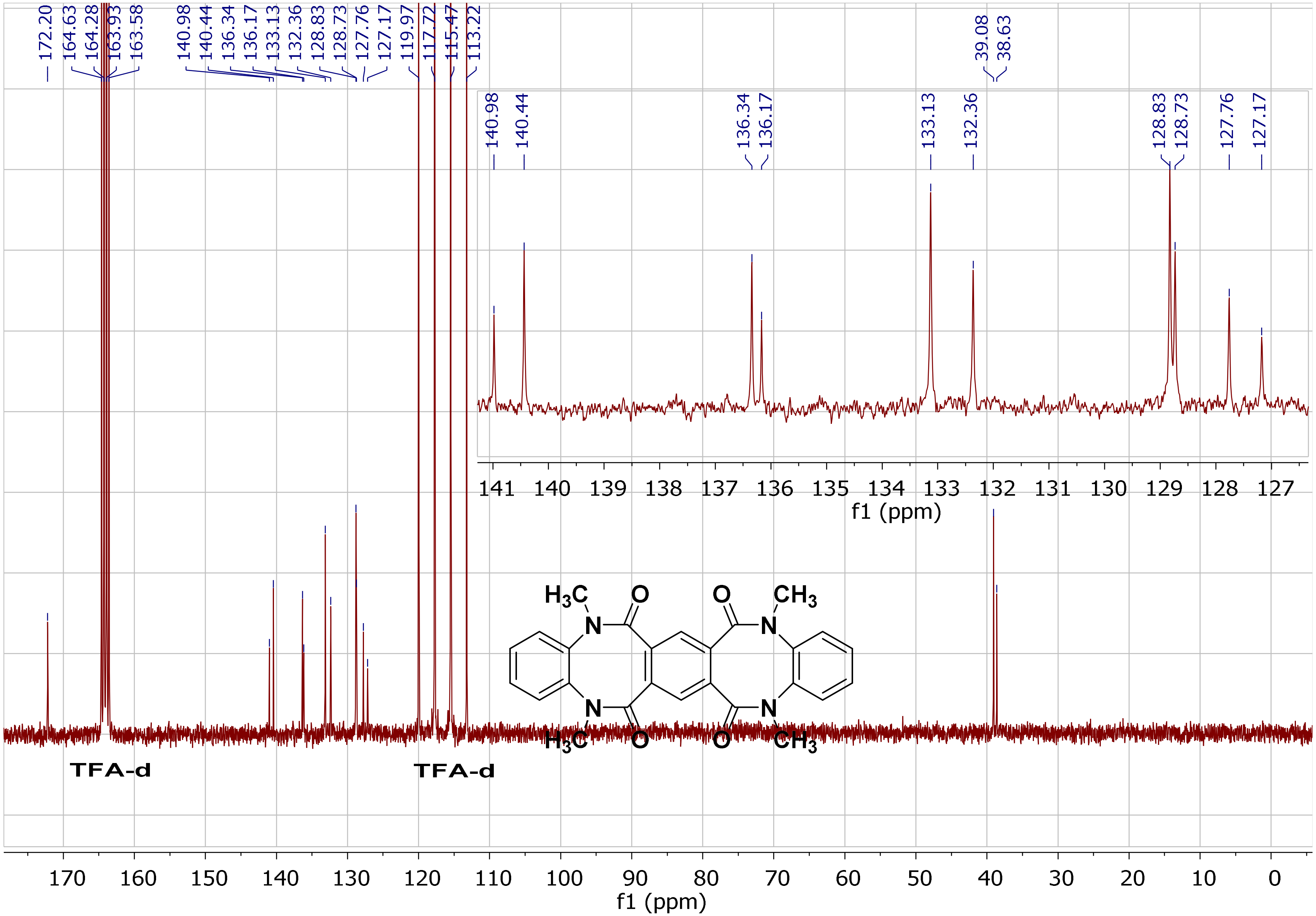

Figure S52. 13C-NMR spectrum for **9b.**

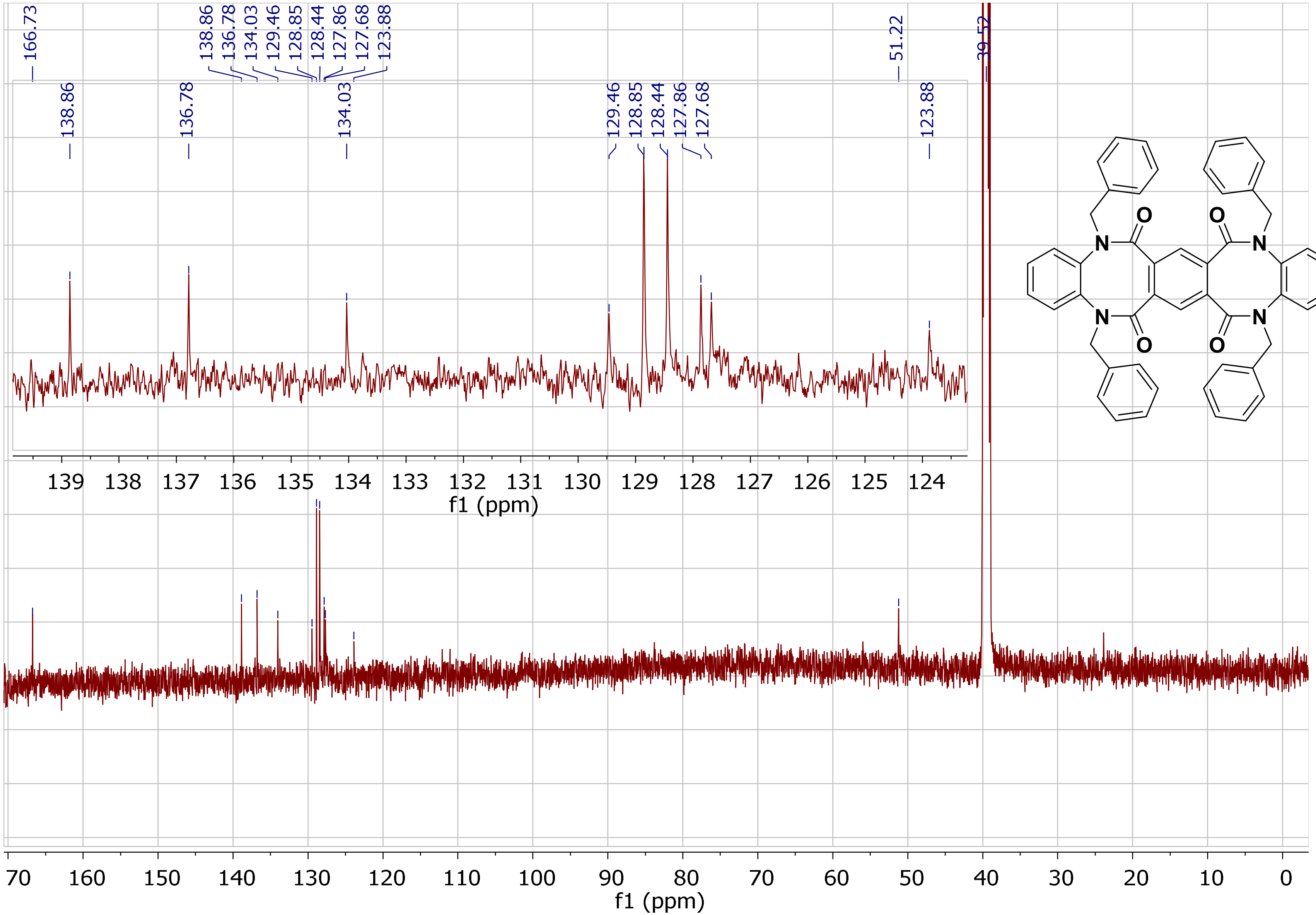

Figure S53. 13C-NMR spectrum for **9c.**

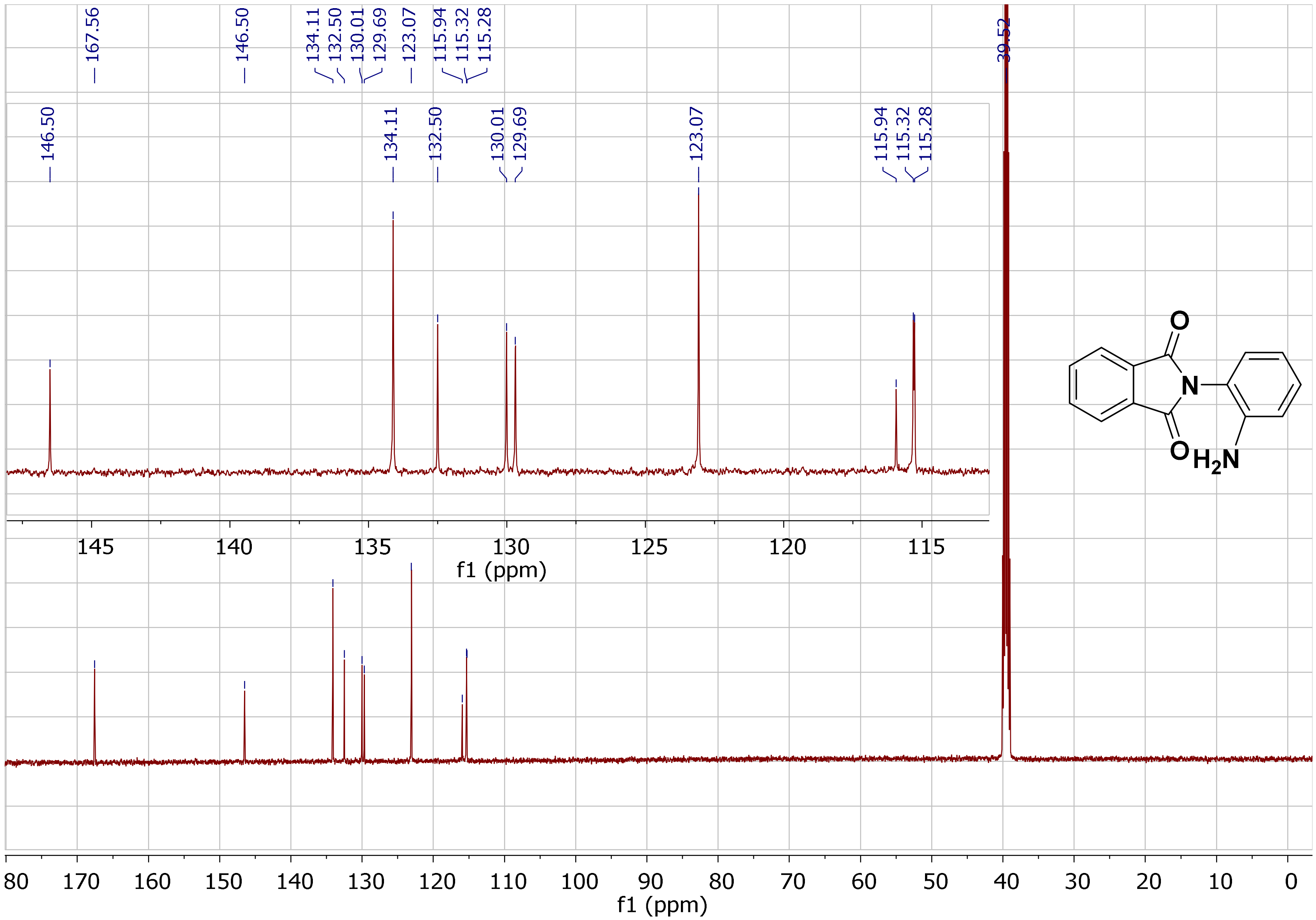

Figure 54. 13C-NMR spectrum for 2-(2-aminophenyl)isoindoline–1,3-dione (**10**).

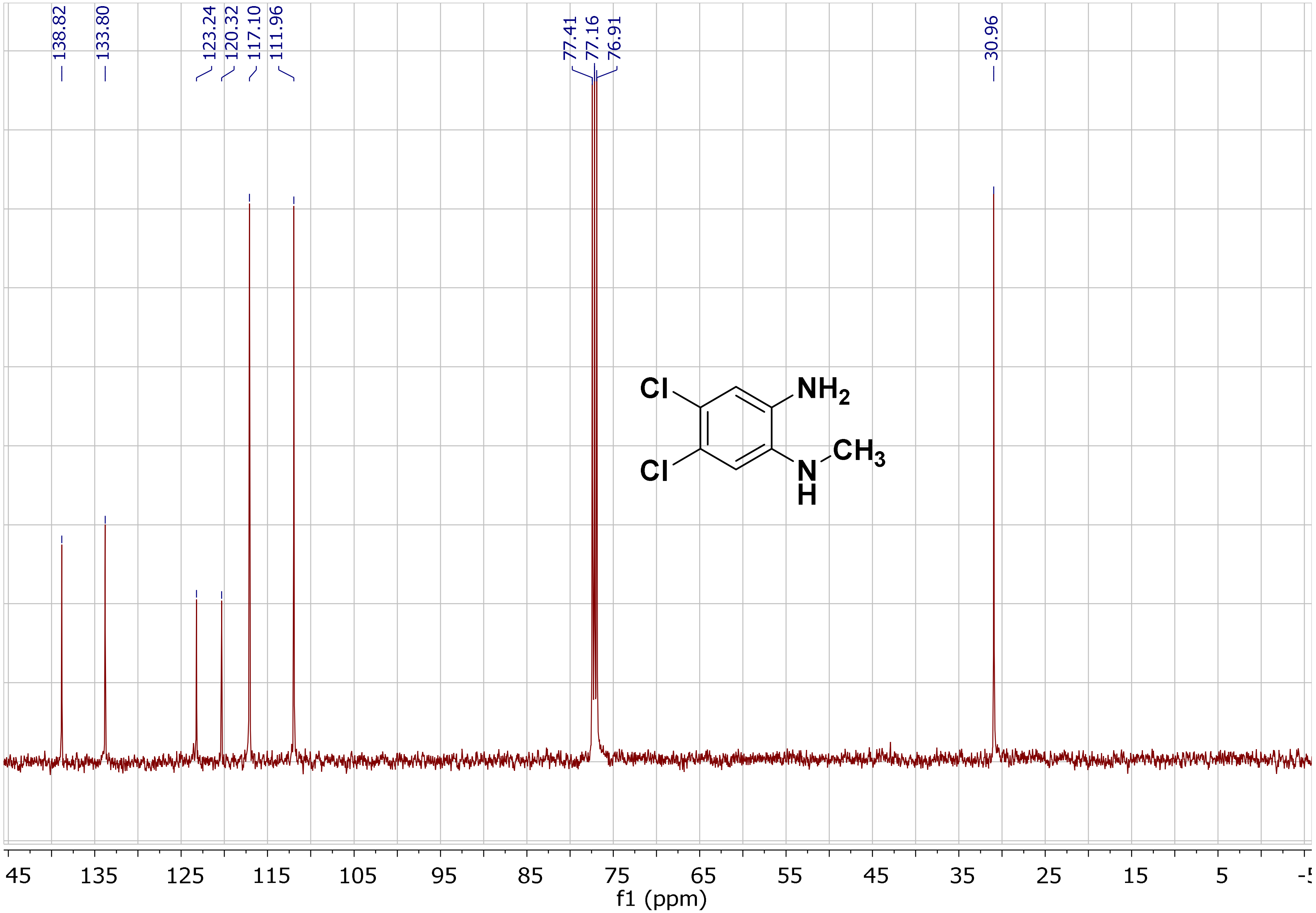

Figure S55. 13C-NMR spectrum for 4,5-dichloro-*N*[1]-methylbenzene–1,2-diamine (**4e**).

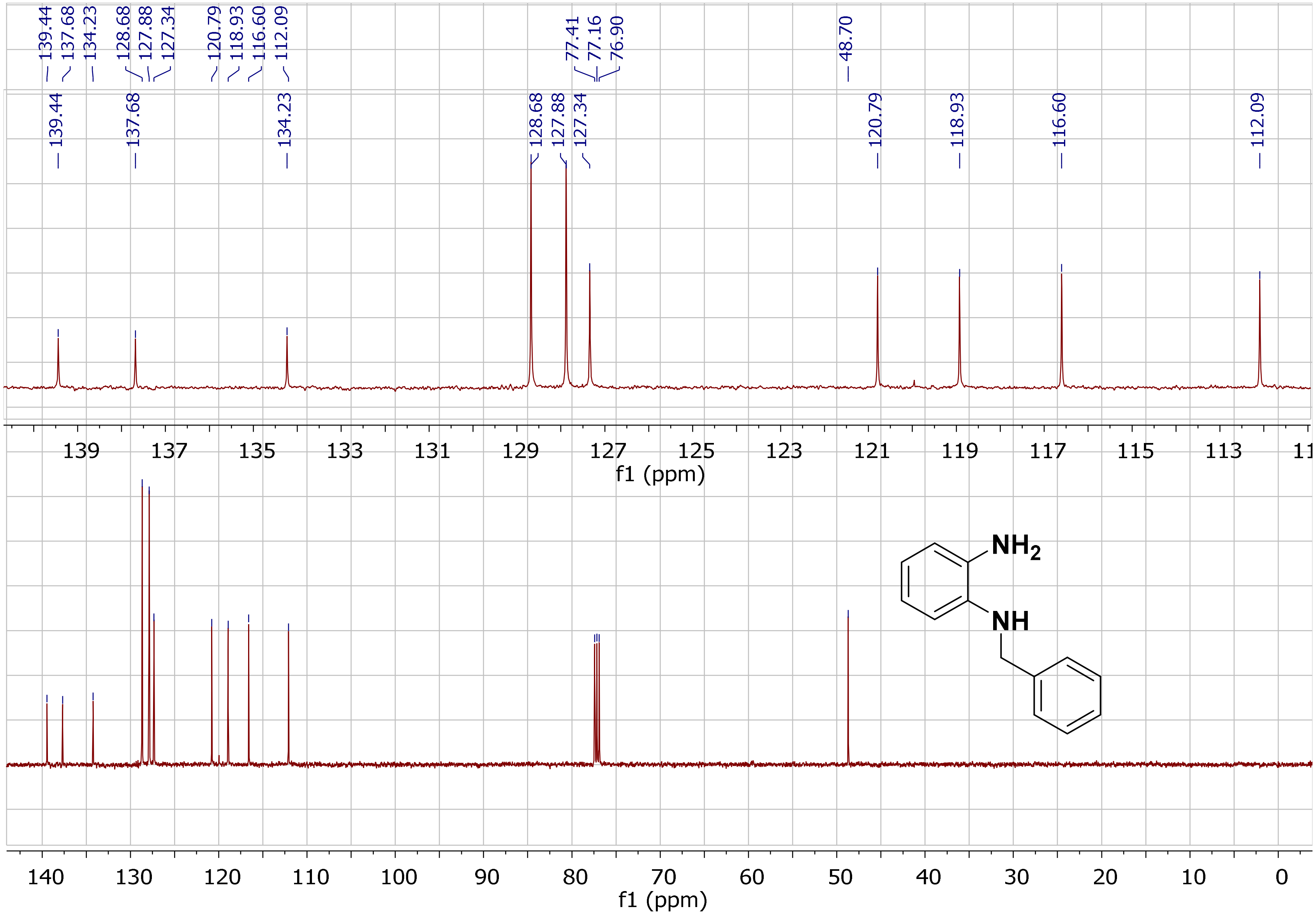

Figure S56. 13C-NMR spectrum for *N*1-benzylbenzene–1,2-diamine (**4f**).

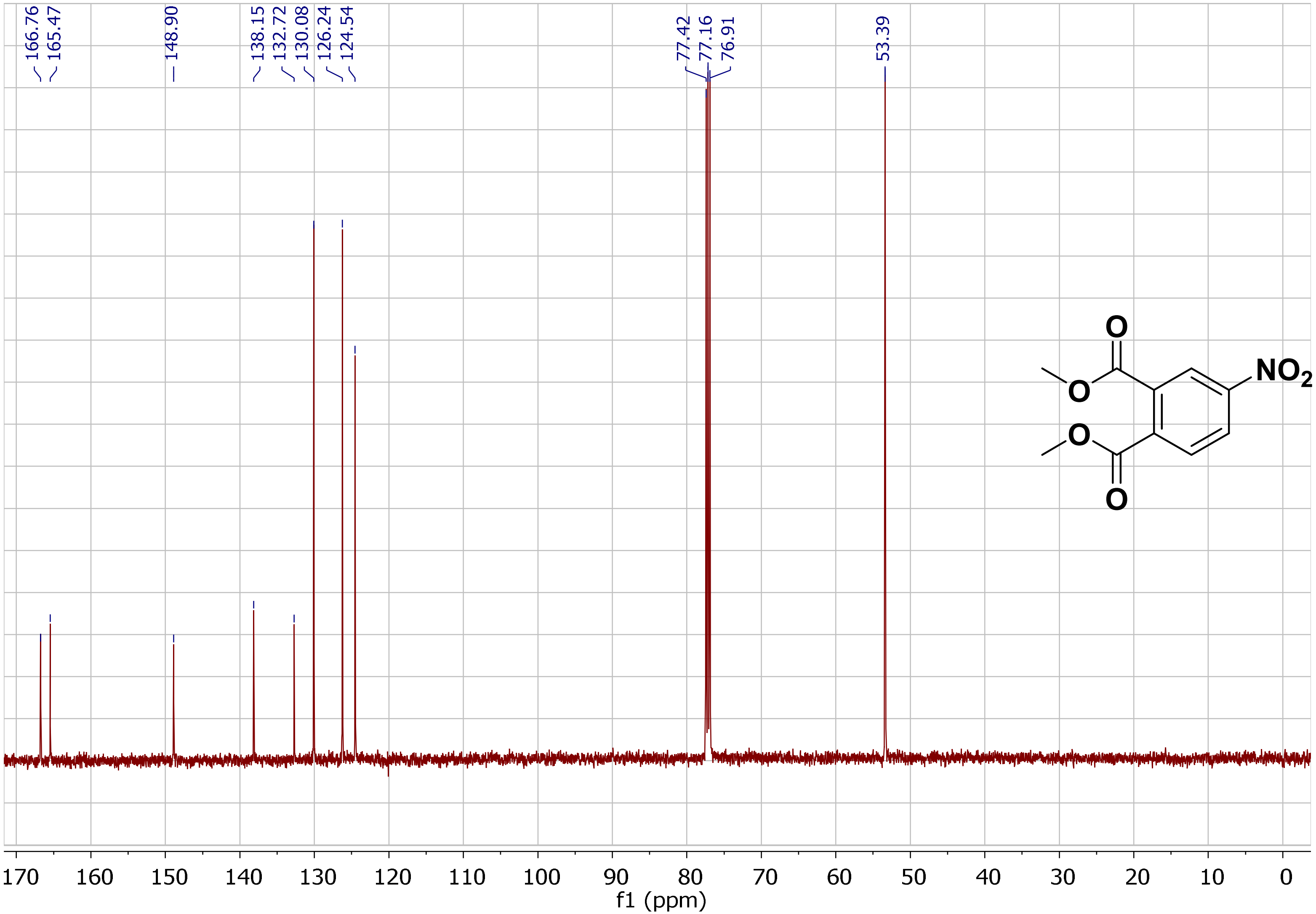

Figure S57. 13C-NMR spectrum for dimethyl 4-nitrophthalate (**5b**).

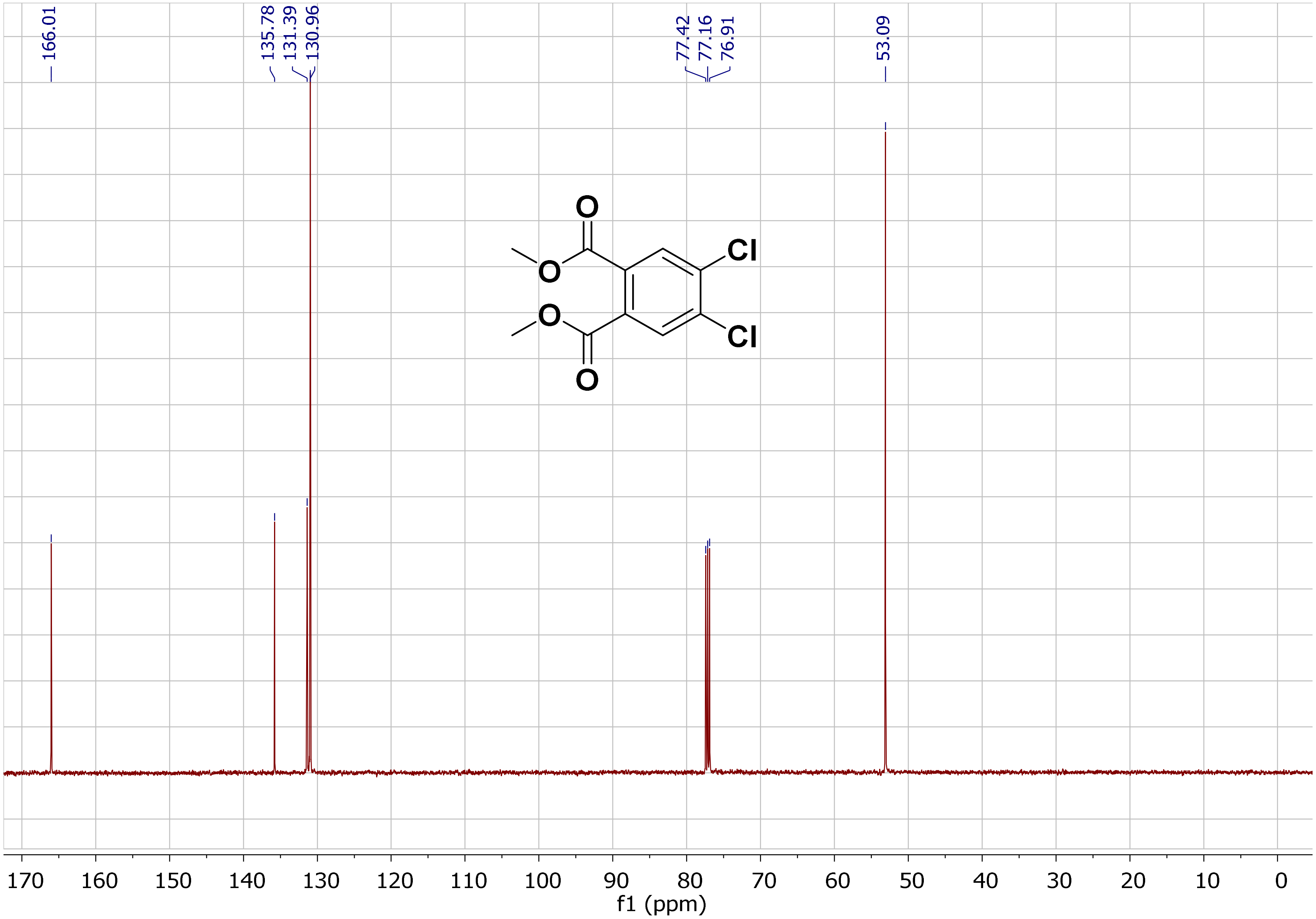

Figure S58. 13C-NMR spectrum for dimethyl 4,5-dichlorophthalate (**5c**).

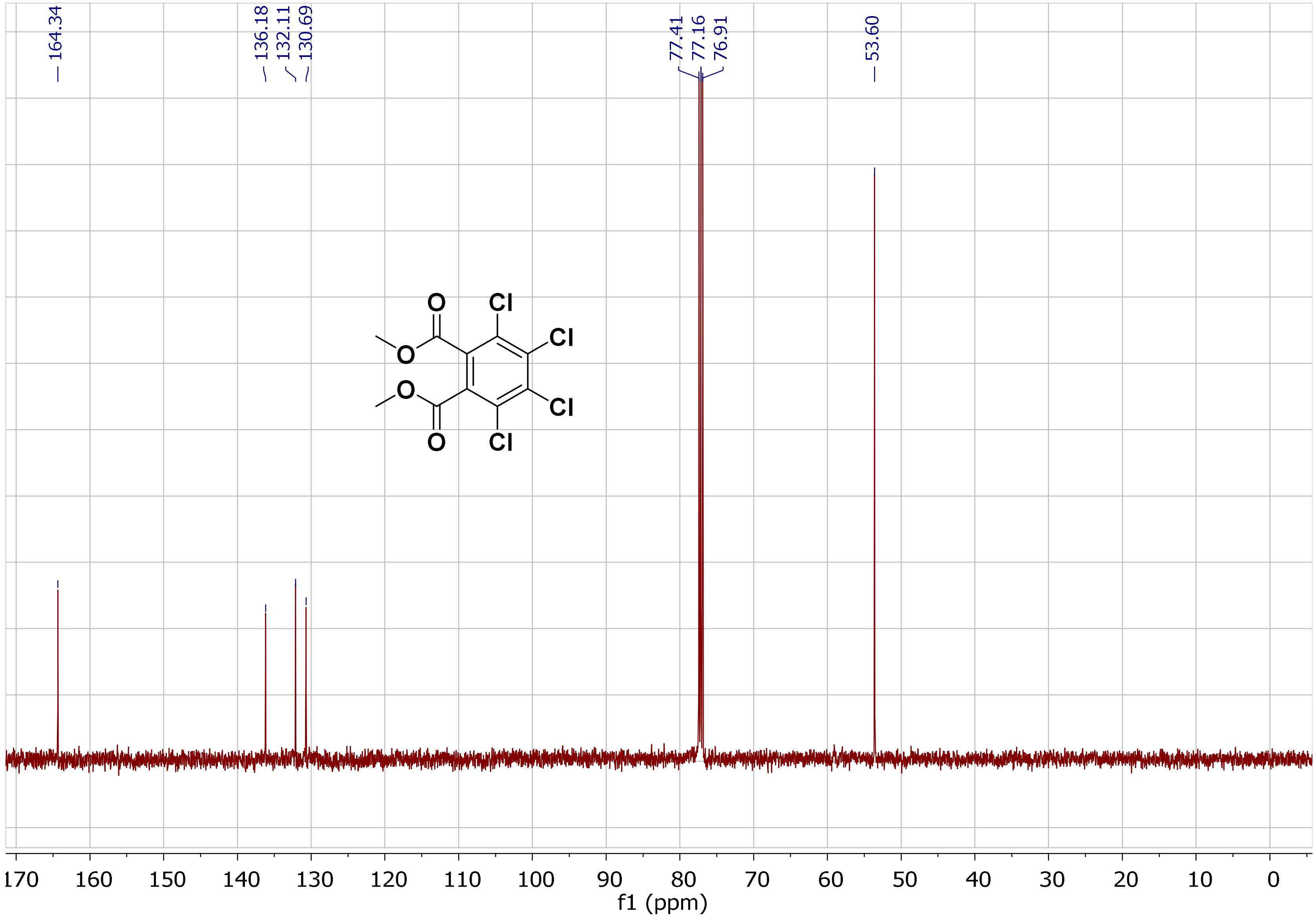

Figure S59. 13C-NMR spectrum for dimethyl 3,4,5,6-tetrachlorophthalate (**5d**).

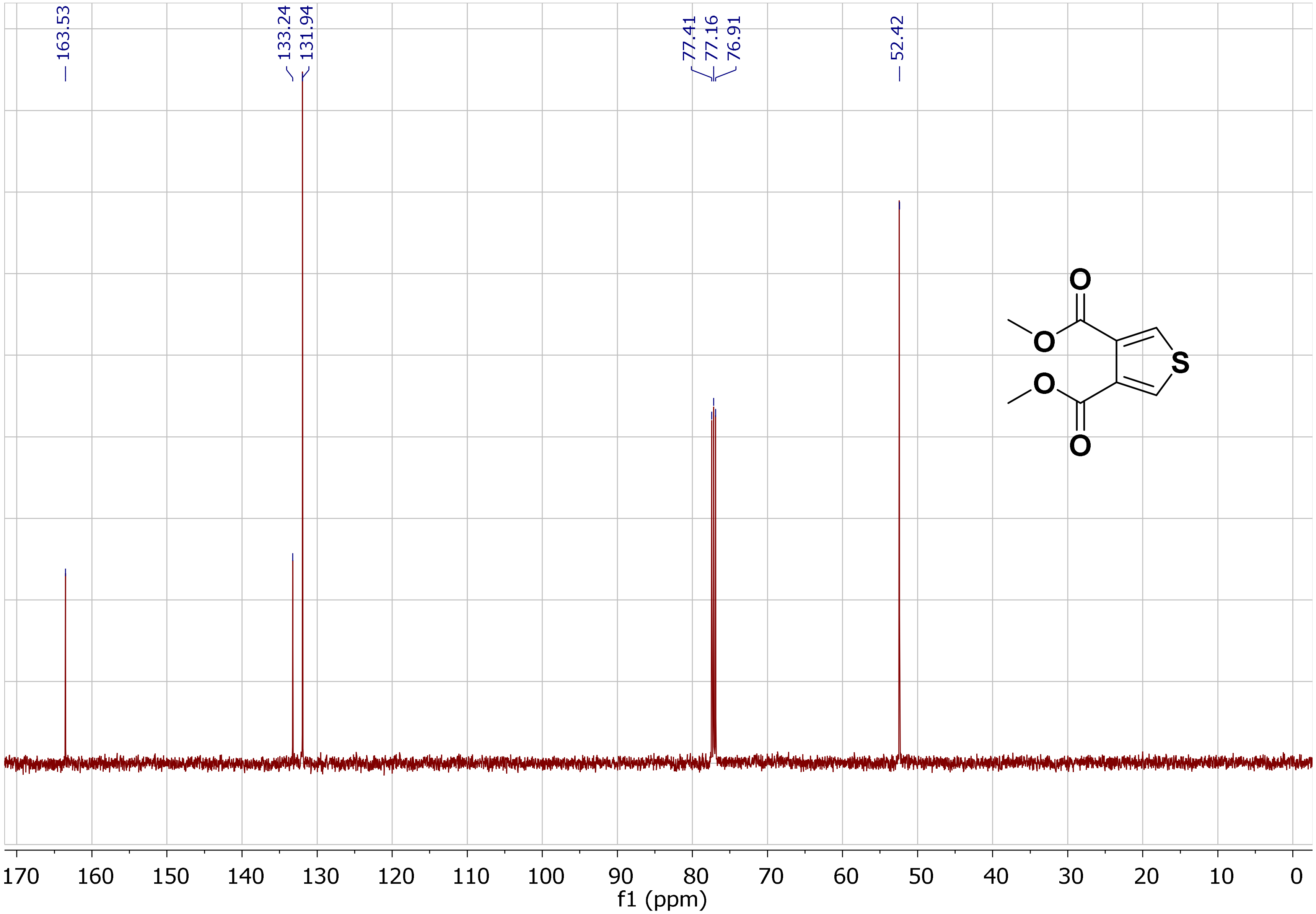

Figure S60. 13C-NMR spectrum for dimethyl thiophene–3,4-dicarboxylate (**5e**).

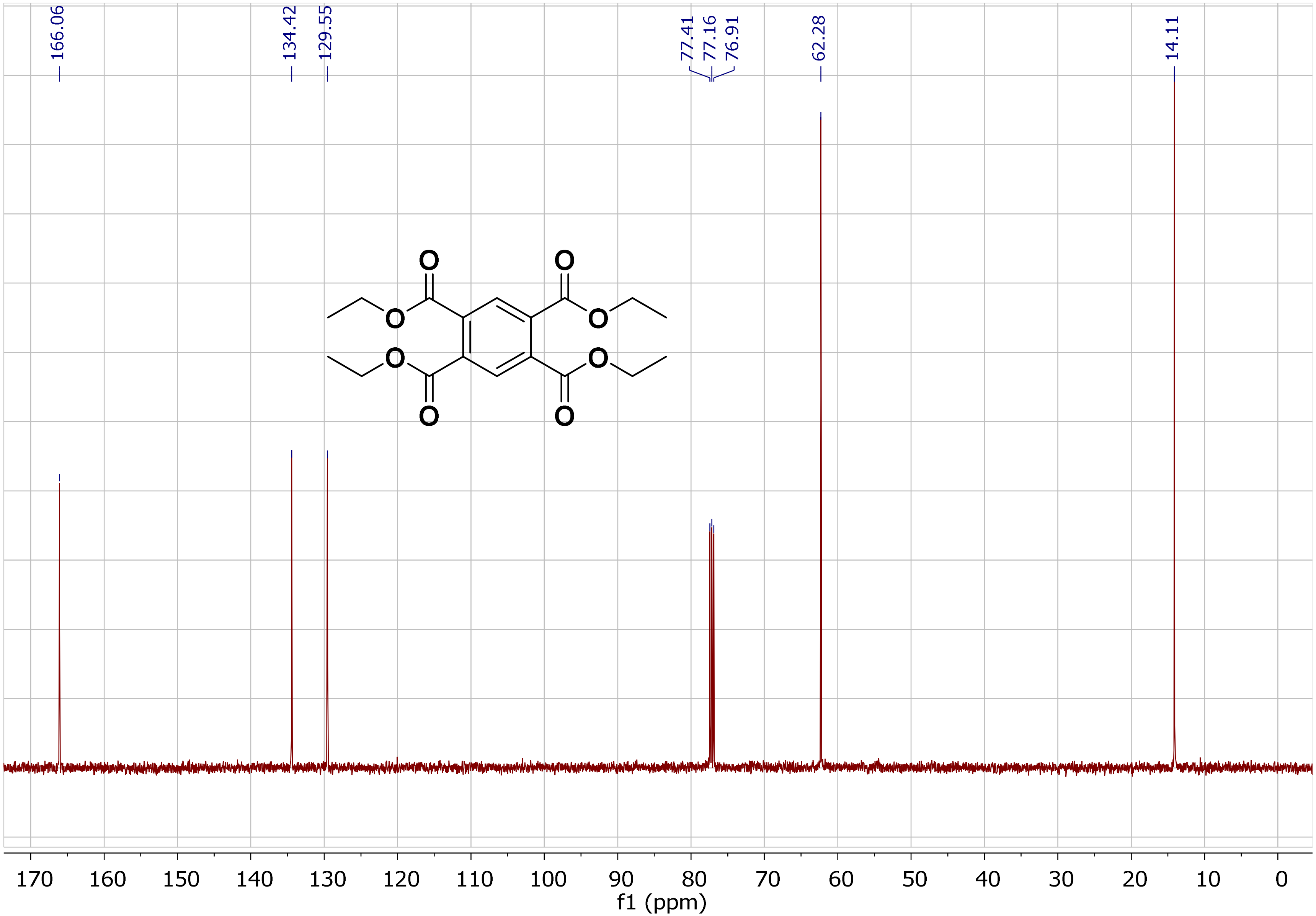

Figure S61. 13C-NMR spectrum for tetraethyl benzene–1,2,4,5-tetracarboxylate (**8**).

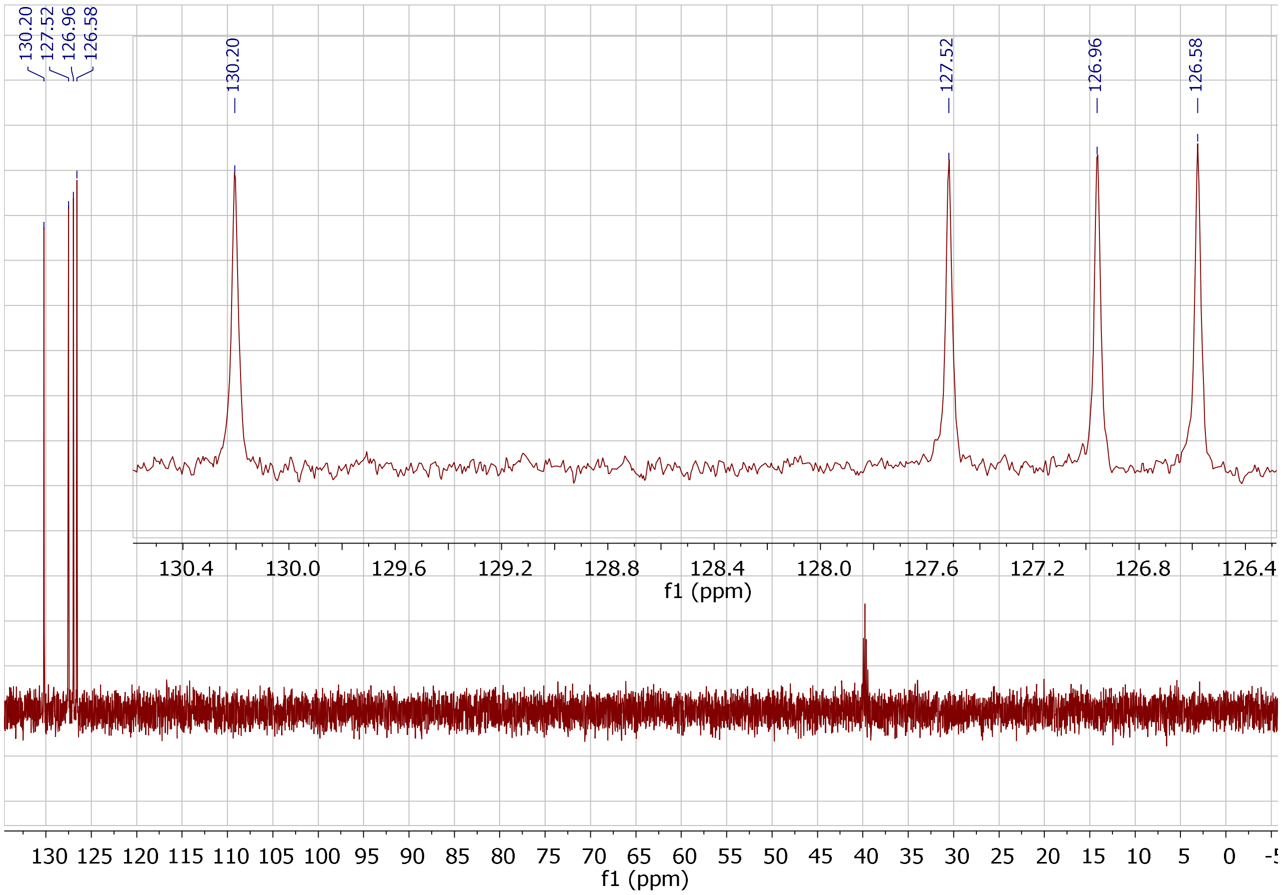

Figure S62. dept135 spectrum for 5,12-dihydrodibenzo[*b*,*f*][1,4]diazocine–6,11-dione (**3a**).

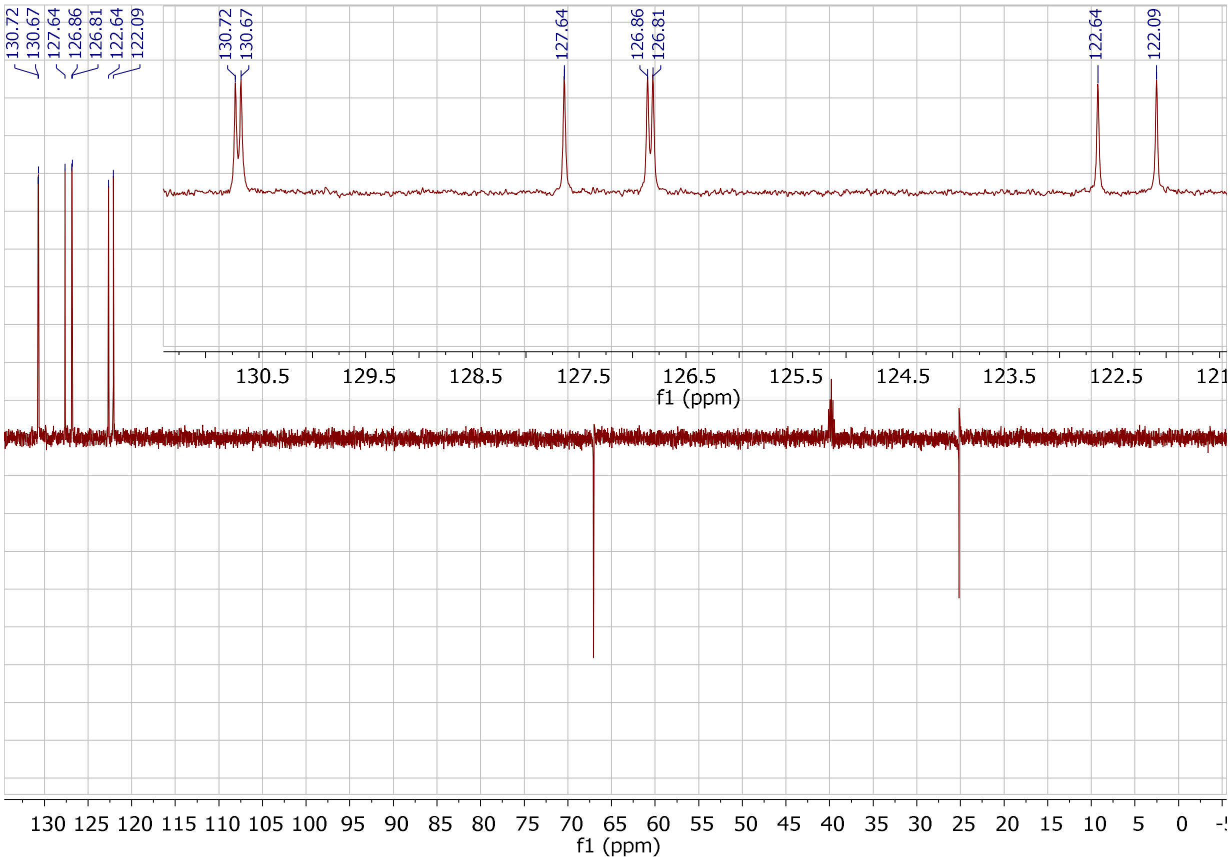

Figure S63. dept135 spectrum for 2-nitro–5,12-dihydrodibenzo[*b*,*f*][1,4]diazocine–6,11-dione (**3b**).

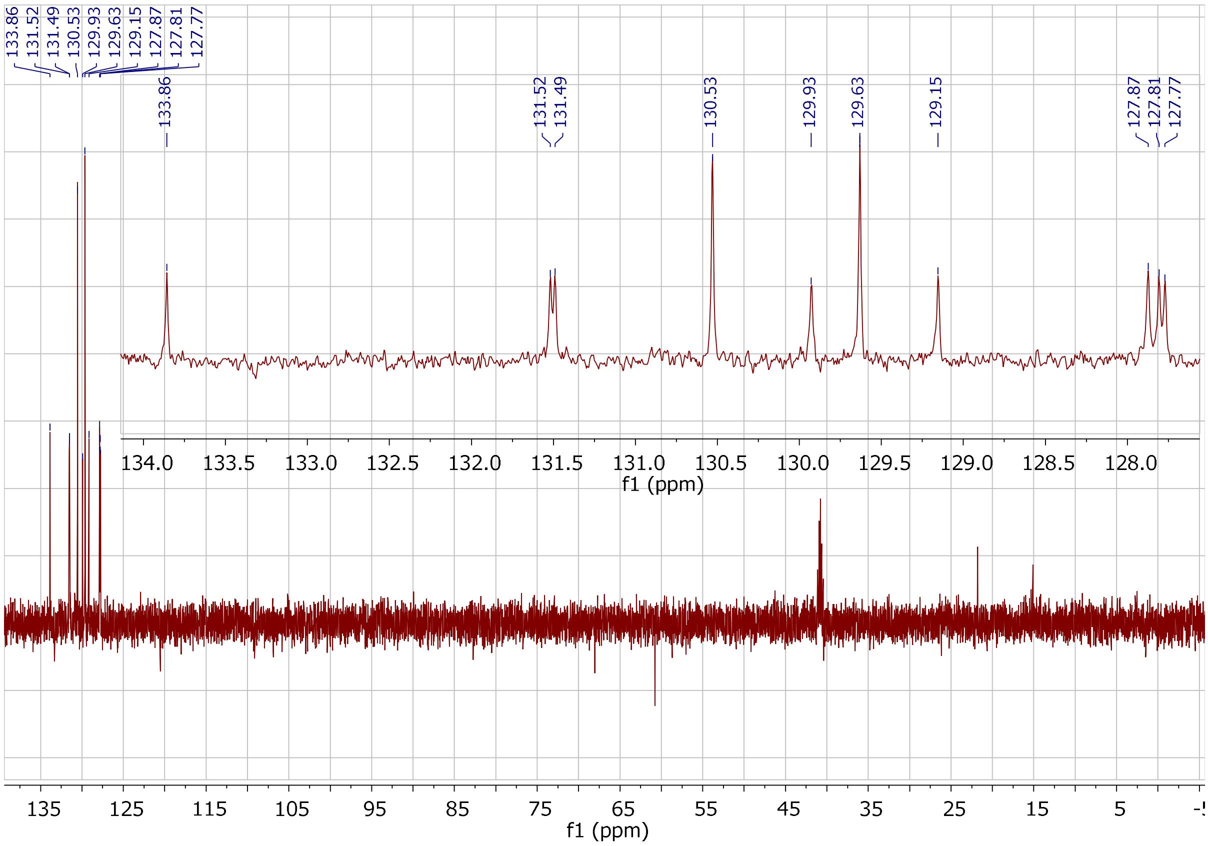

Figure S64. dept135 spectrum for 2-benzoyl–5,12-dihydrodibenzo[*b*,*f*][1,4]diazocine–6,11-dione (**3c**).

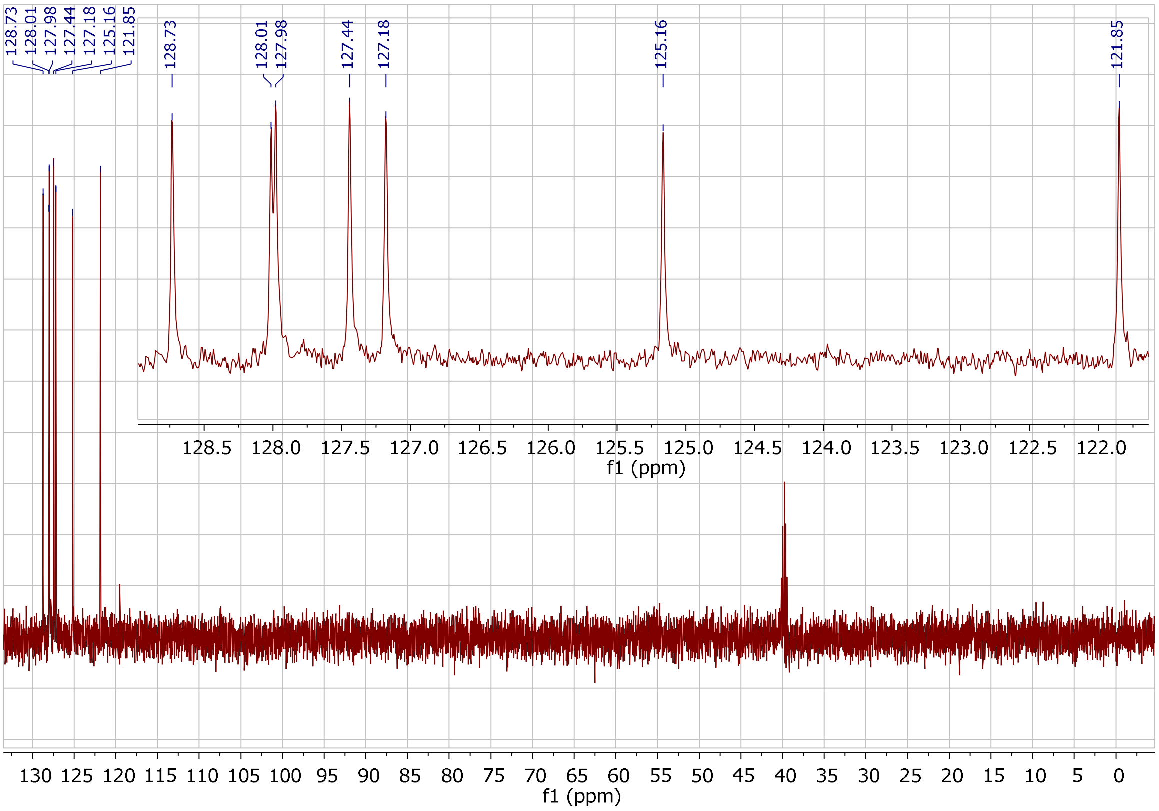

Figure S65. dept135 spectrum for 8-nitro–5,12-dihydrodibenzo[*b*,*f*][1,4]diazocine–6,11-dione (**3d**).

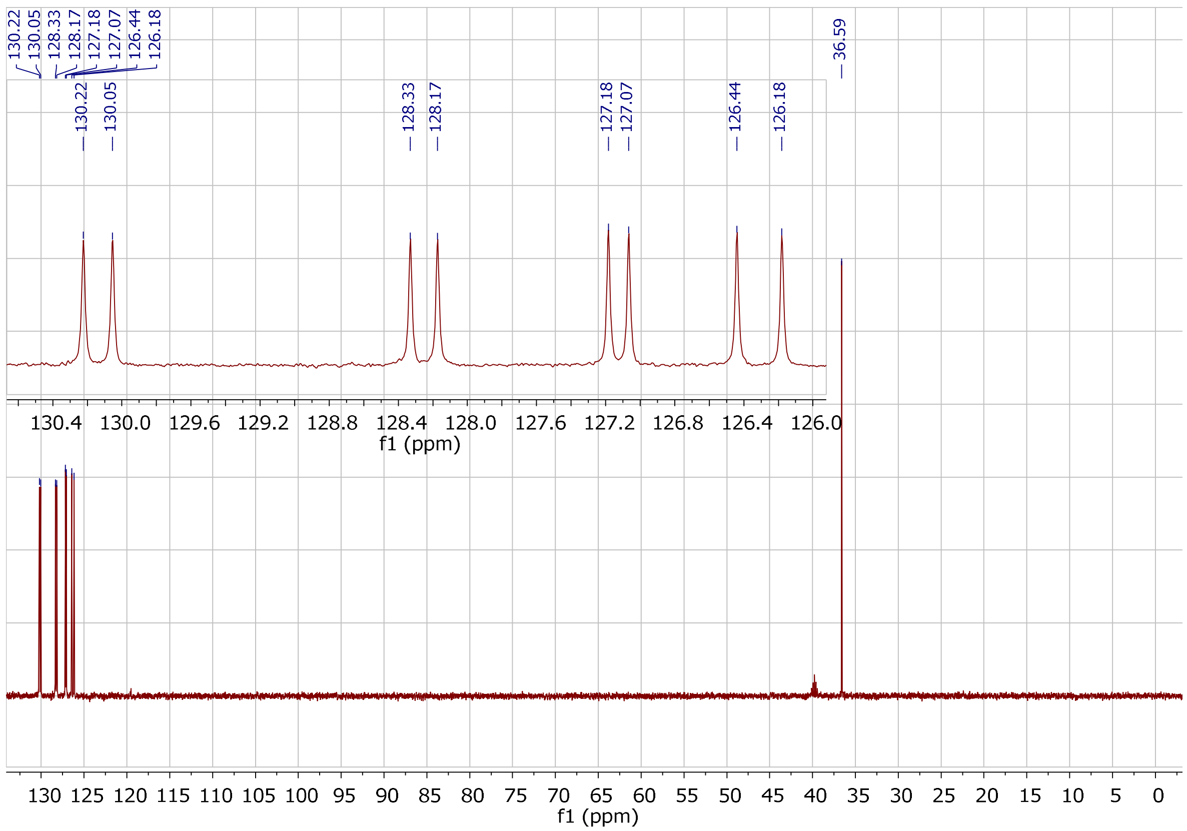

Figure S66. dept135 spectrum for 5-methyl–5,12-dihydrodibenzo[*b*,*f*][1,4]diazocine–6,11-dione (**3e**).

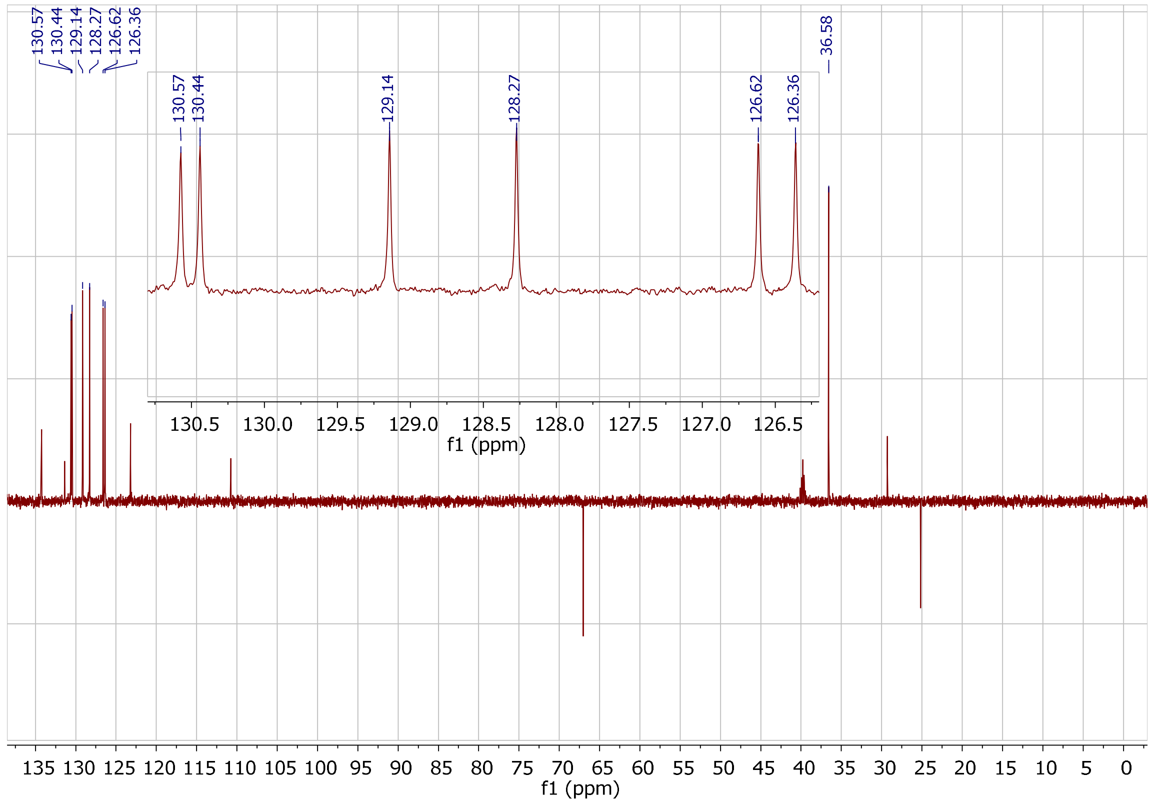

Figure S67. dept135 spectrum for 2,3-dichloro–5-methyl–5,12-dihydrodibenzo[*b*,*f*][1,4]diazocine–6,11-dione (**3f**).

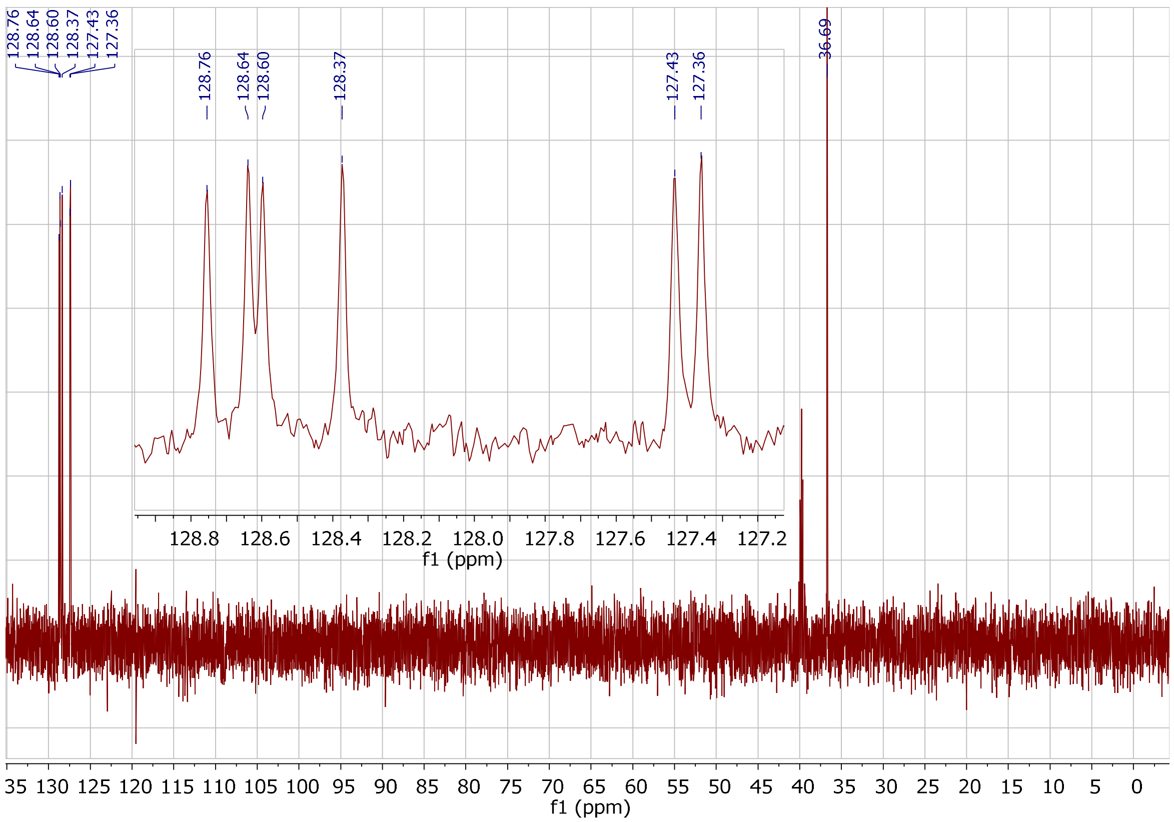

Figure S68. dept135 spectrum for 8,9-dichloro–5-methyl–5,12-dihydrodibenzo[*b*,*f*][1,4]diazocine–6,11-dione (**3g**).

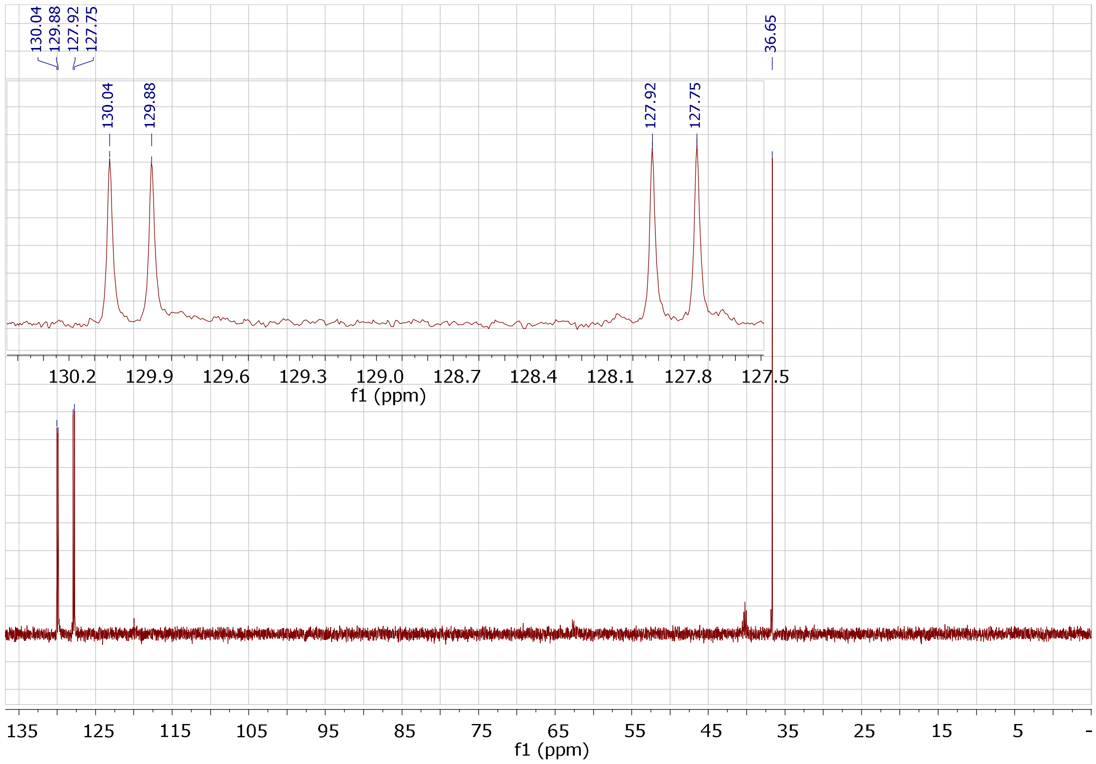

Figure S69. dept135 spectrum for 7,8,9,10-tetrachloro–5-methyl–5,12-dihydrodibenzo[*b*,*f*][1,4]diazocine–6,11-dione (**3h**).

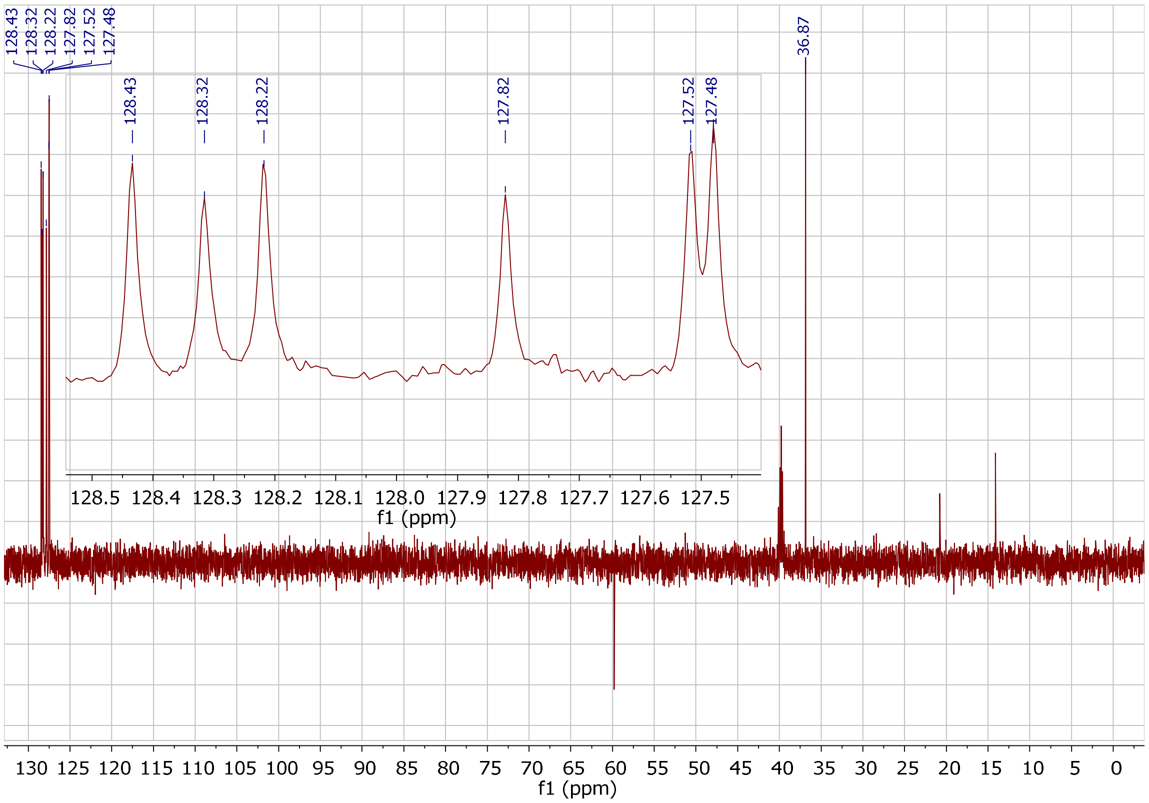

Figure S70. dept135 spectrum for 5-methyl–5,10-dihydrobenzo[*b*]thieno[3,4-*f*][1,4]diazocine–4,11-dione (**3i**).

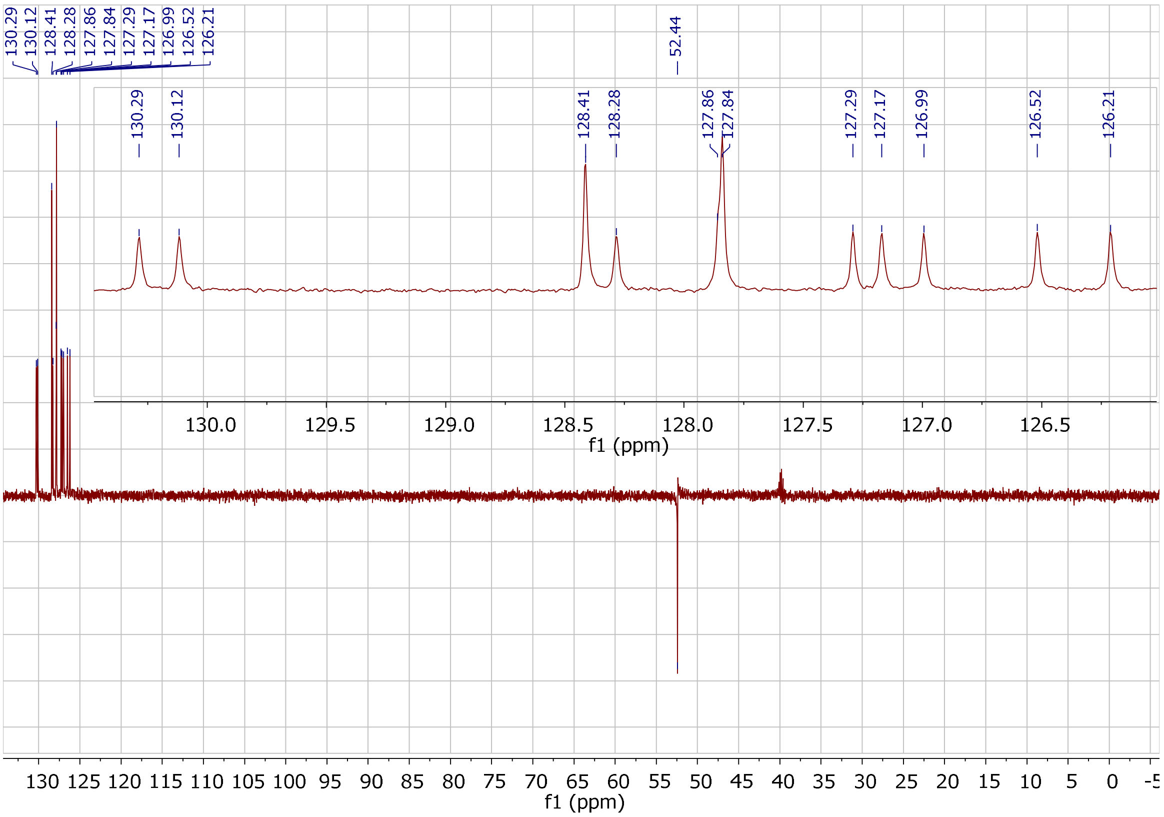

Figure S71. dept135 spectrum for 5-benzyl–5,12-dihydrodibenzo[*b,f*][1,4]diazocine–6,11-dione (**3j**).

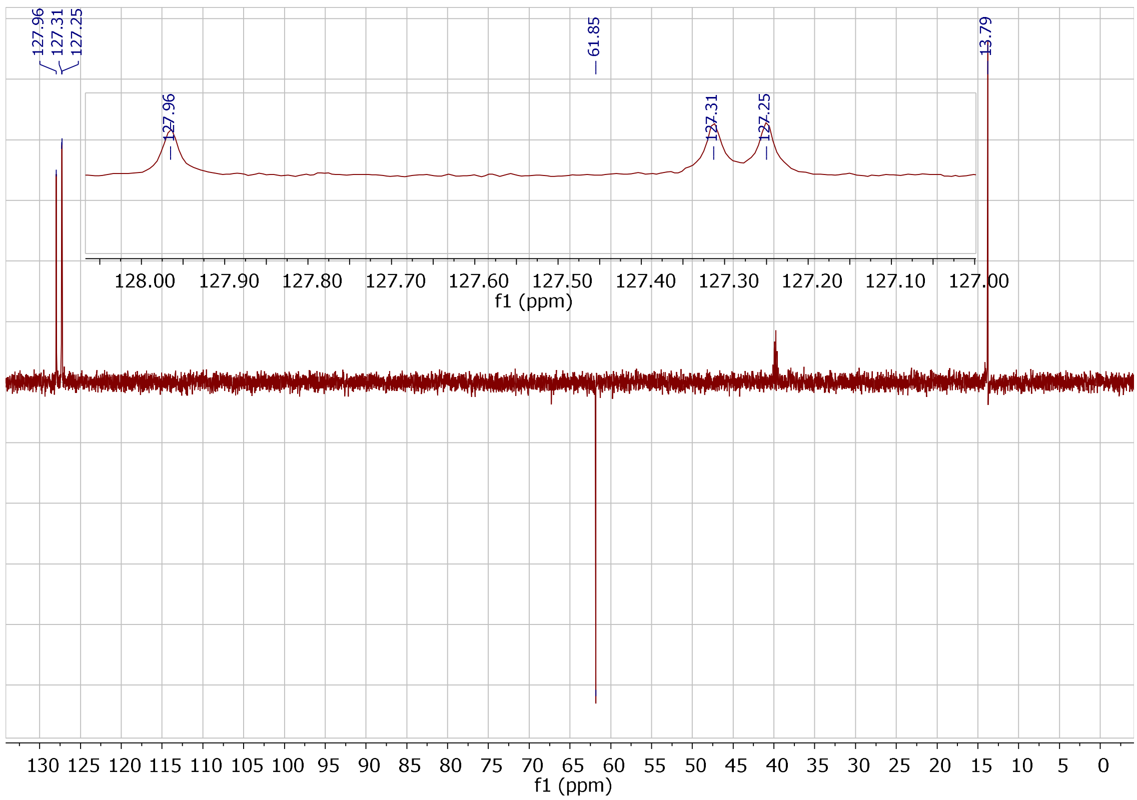

Figure S72. dept135 spectrum for diethyl 6,11-dioxo–5,6,11,12-tetrahydrodibenzo[*b*,*f*][1,4]diazocine–8,9-dicarboxylate (**3k**).

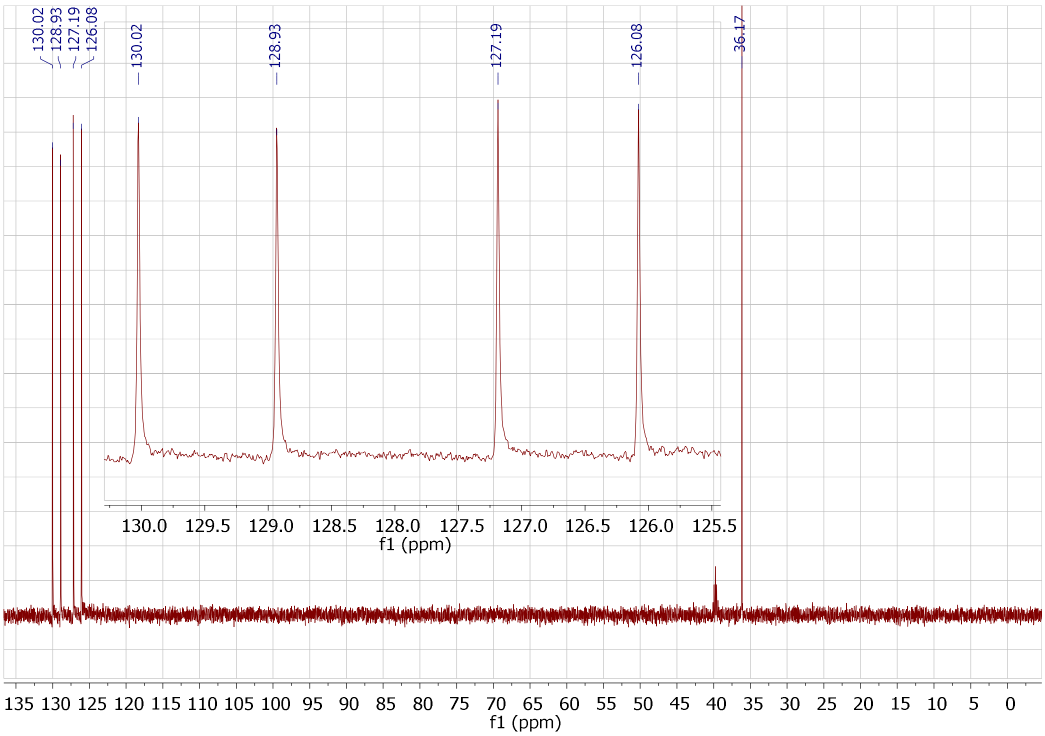

Figure S73. dept135 spectrum for 5,12-dimethyl–5,12-dihydrodibenzo[*b*,*f*][1,4]diazocine–6,11-dione (**3l**).

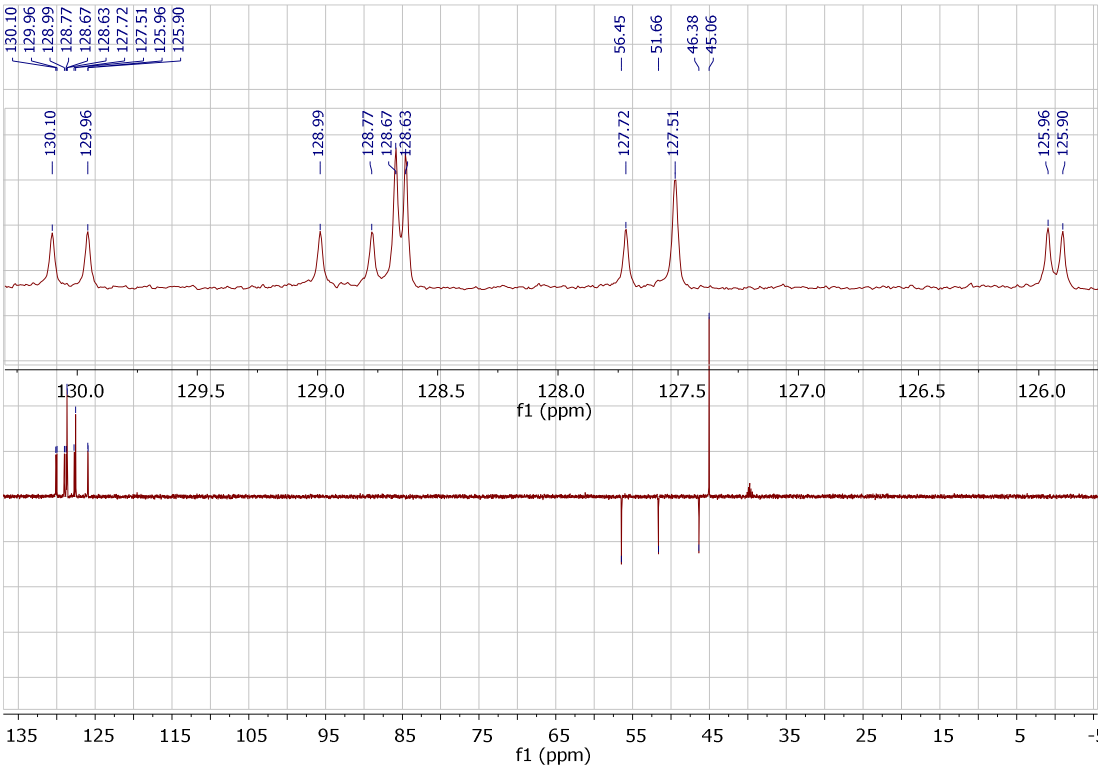

Figure S74. dept135 spectrum for 5-benzyl–12-(2-(dimethylamino)ethyl)–5,12-dihydrodibenzo[*b*,*f*][1,4]diazocine–6,11-dione (**3m**).

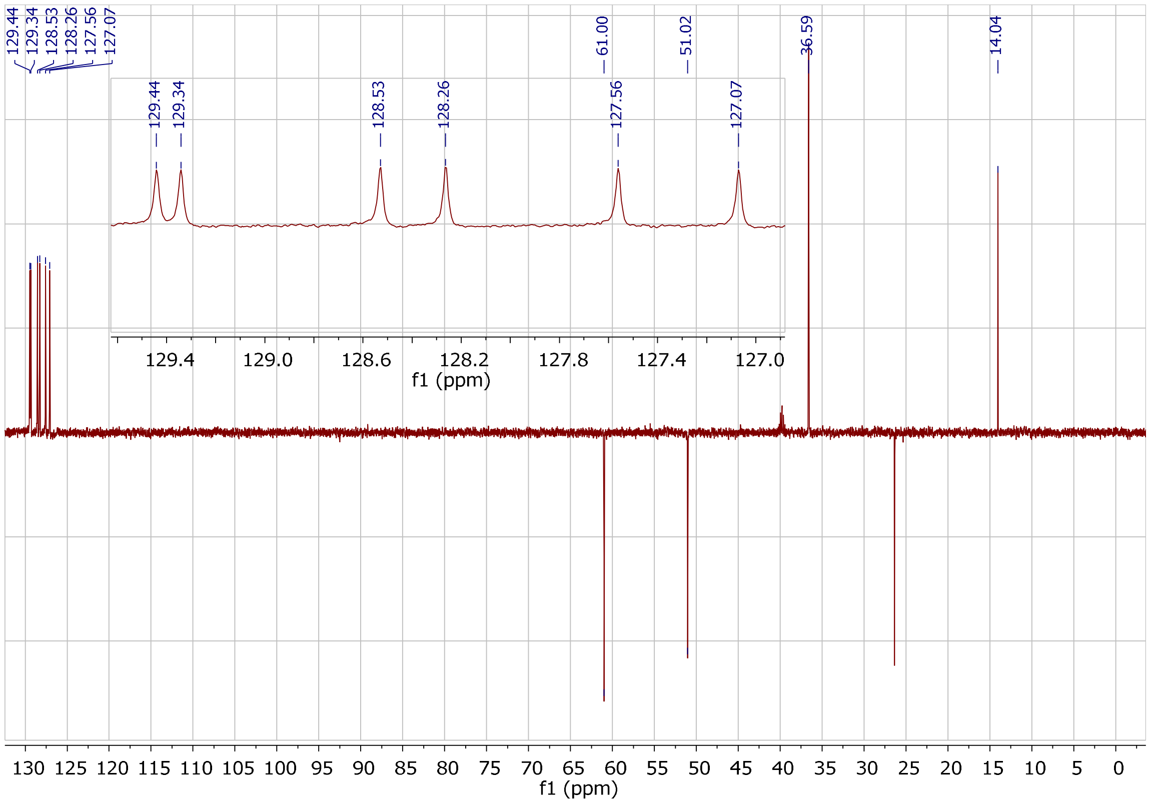

Figure S75. dept135 spectrum for ethyl 2-(8,9-dichloro–12-methyl–6,11-dioxo–11,12-dihydrodibenzo[*b*,*f*][1,4]diazocin–5(6*H*)-yl)acetate (**3n**).

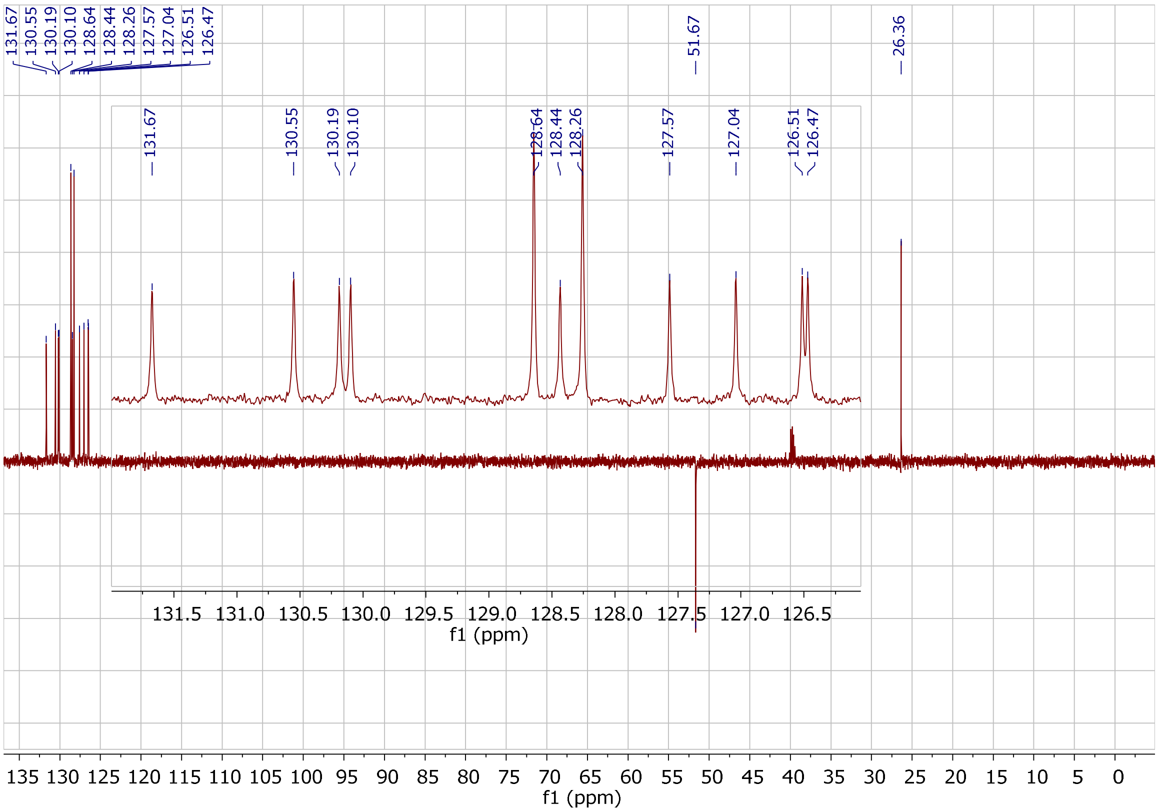

Figure S76. dept135 spectrum for 5-acetyl–12-benzyl–5,12-dihydrodibenzo[*b,f*][1,4]diazocine–6,11-dione (**3o**).

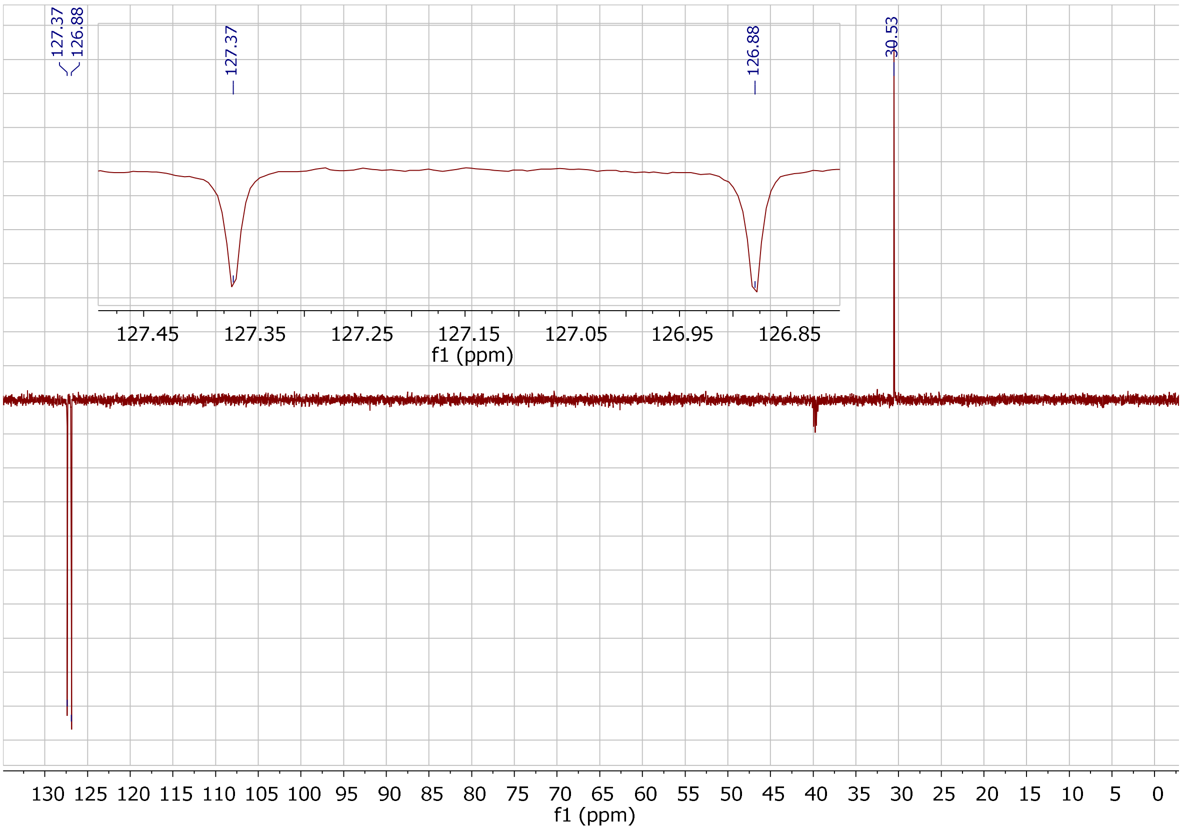

Figure S77. dept135 spectrum for 1,3,4,6-tetrahydrobenzo[*b*][1,4]diazocine–2,5-dione (**6**).

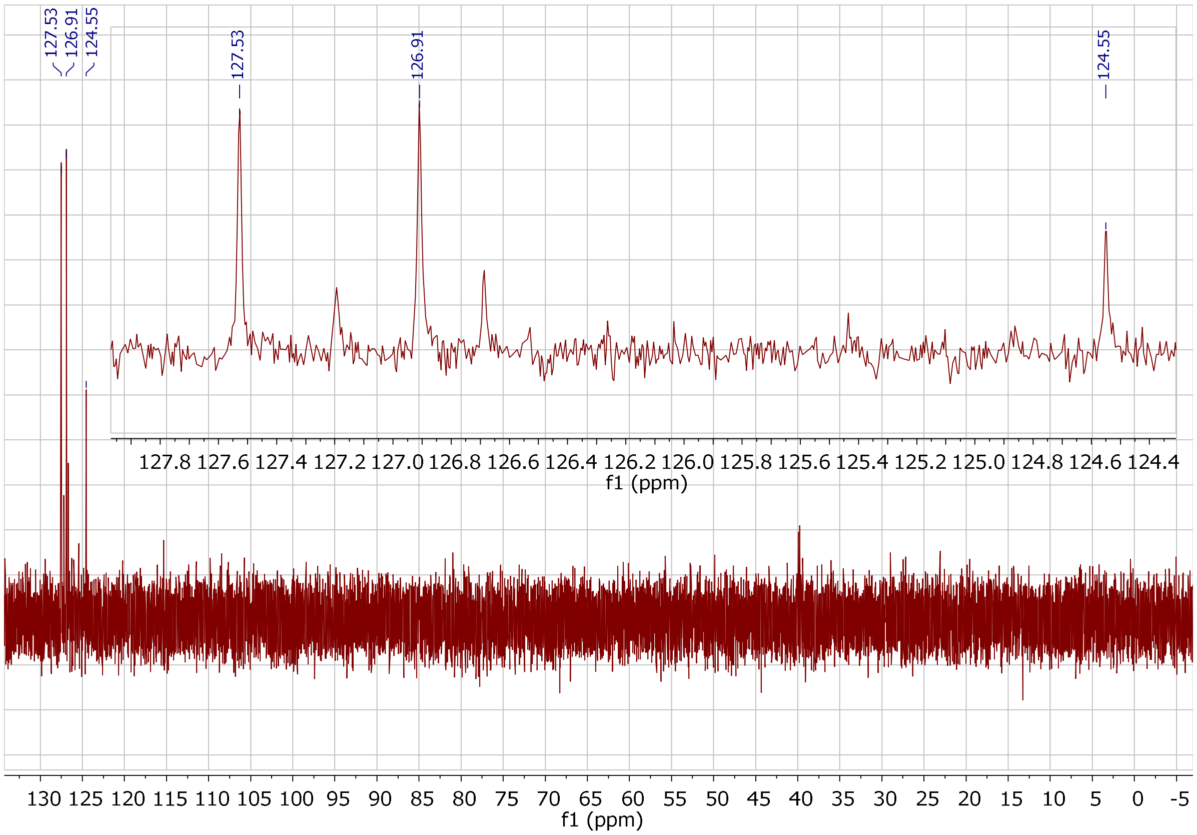

Figure S78. dept135 spectrum for **9a.**

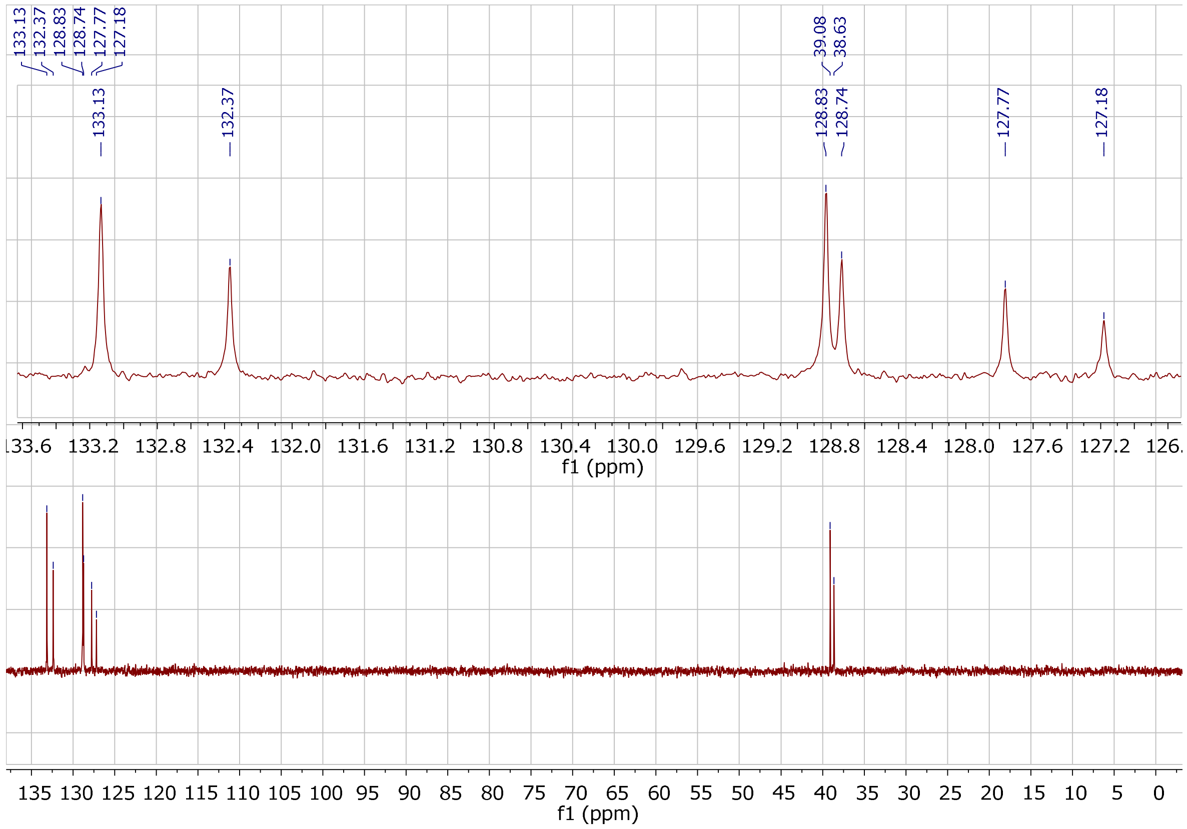

Figure S79. dept135 spectrum for **9b.**

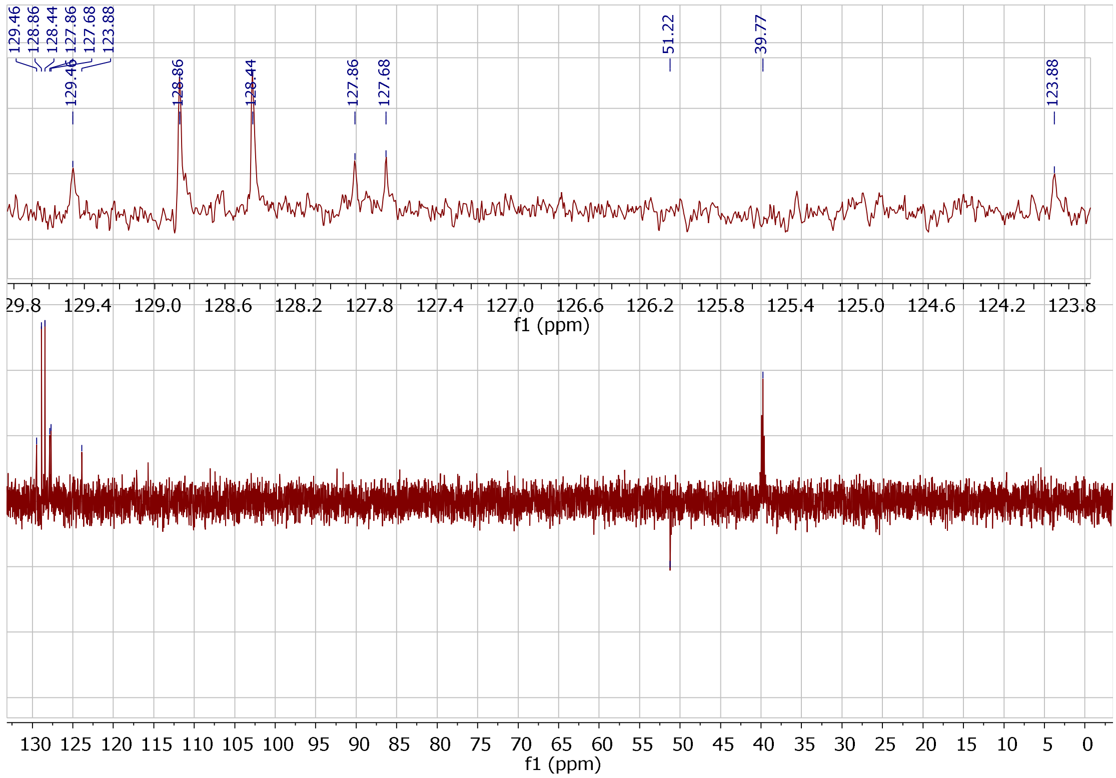

Figure S80. dept135 spectrum for **9c.**

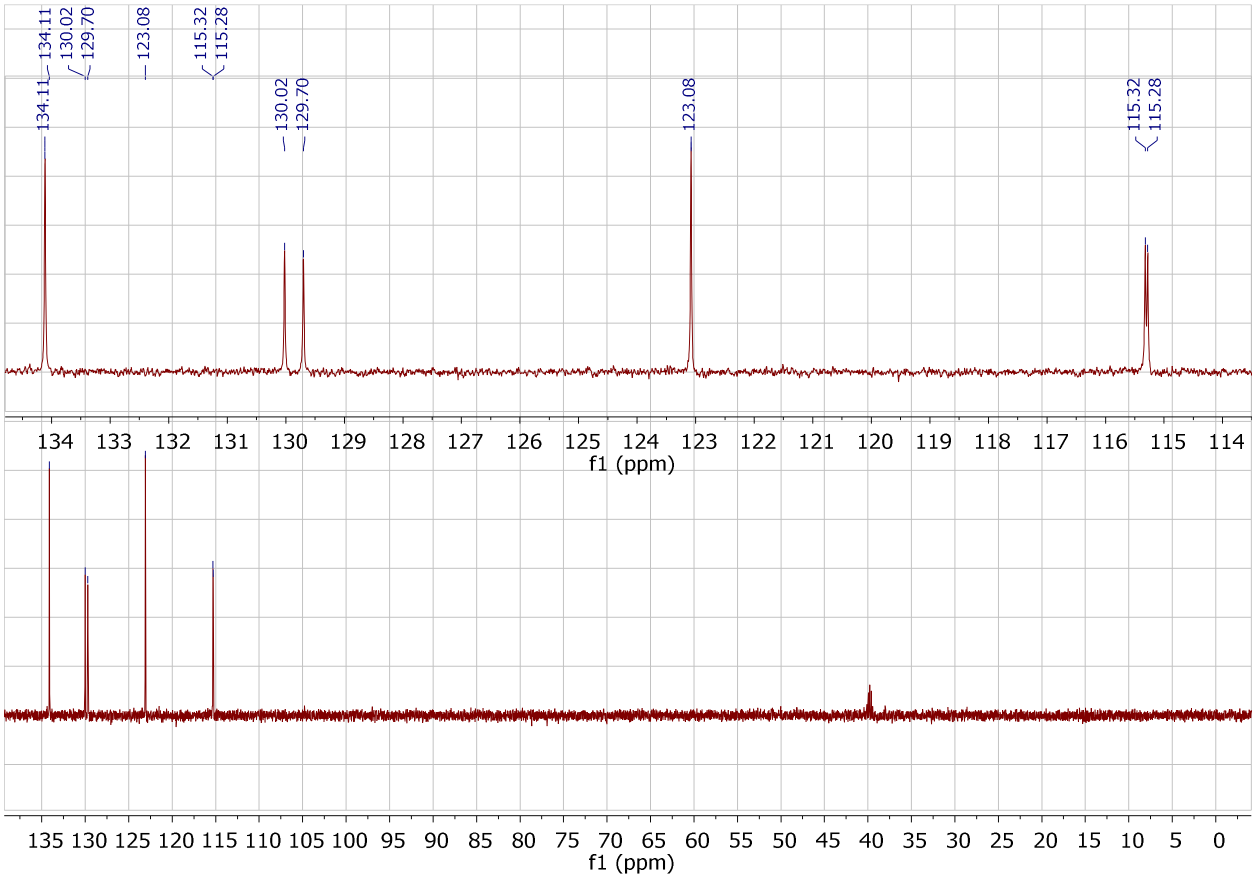

Figure S81. dept135 spectrum for 2-(2-aminophenyl)isoindoline–1,3-dione (**10**).

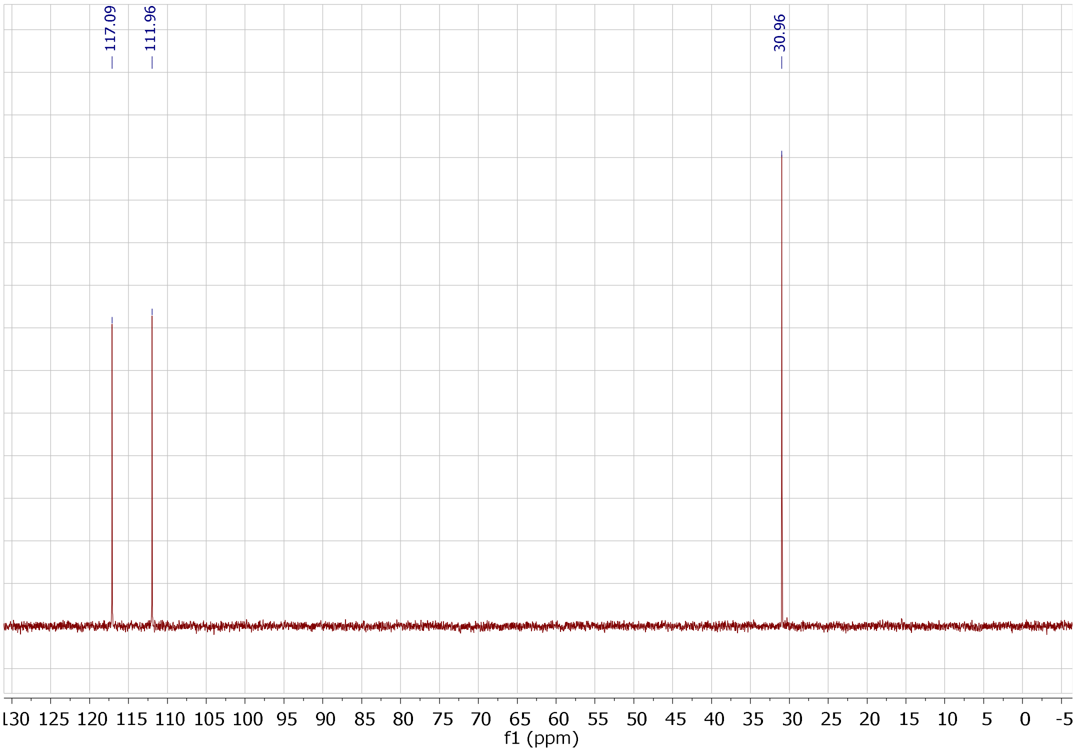

Figure S82. dept135 spectrum for 4,5-dichloro-*N*¹-methylbenzene–1,2-diamine (**4e**).

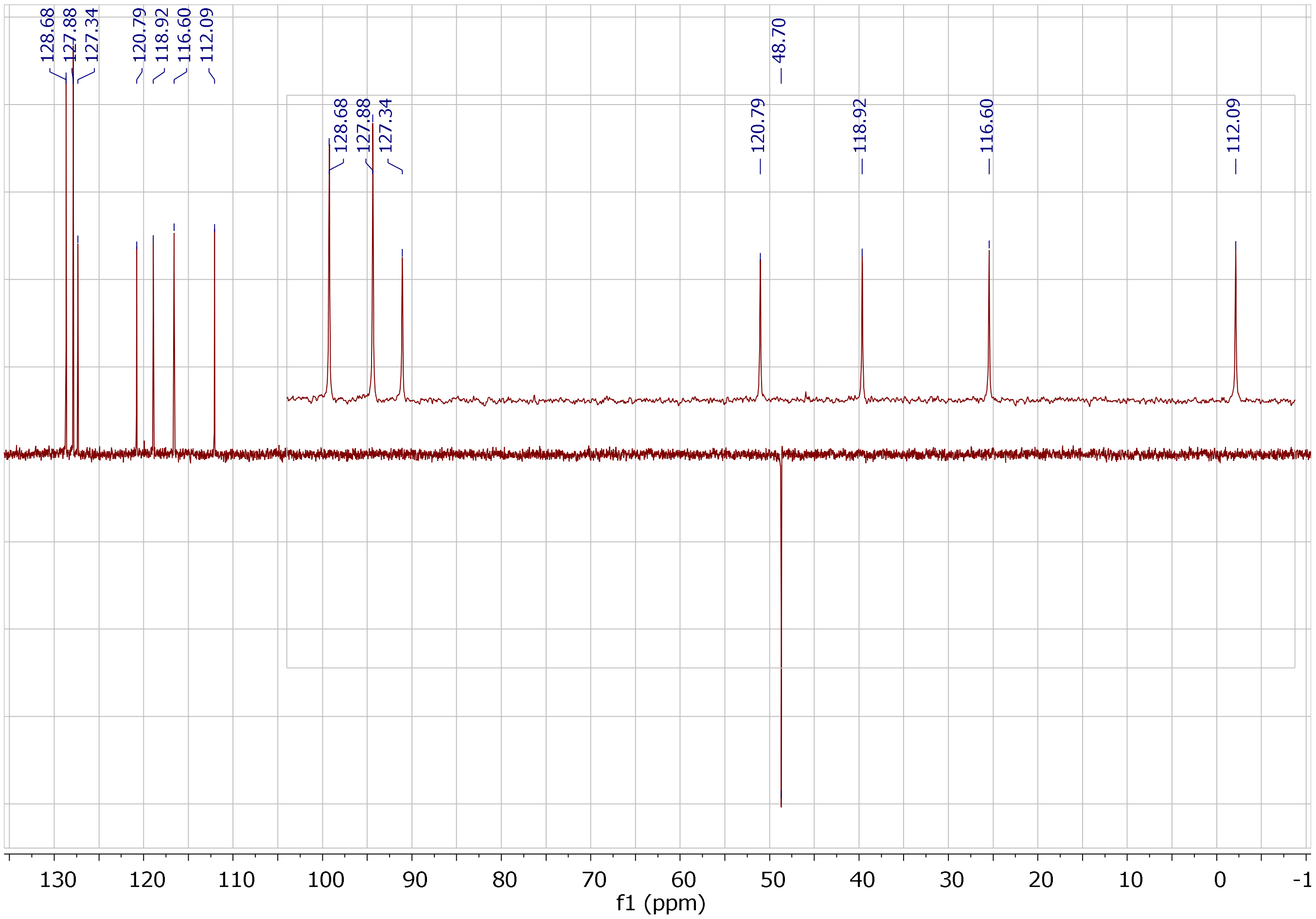

Figure S83. dept135 spectrum for *N*¹-benzylbenzene–1,2-diamine (**4f**).

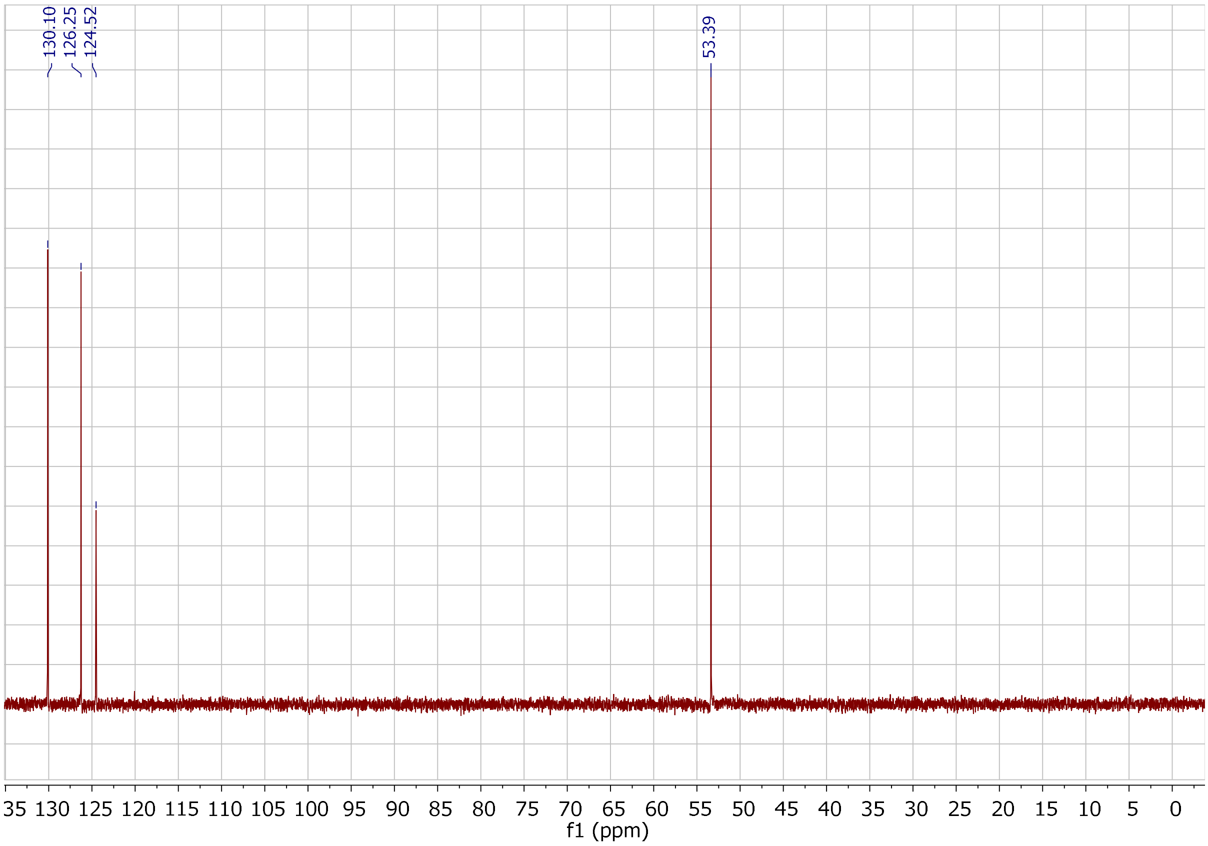

Figure S84. dept135 spectrum for dimethyl 4-nitrophthalate (**5b**).

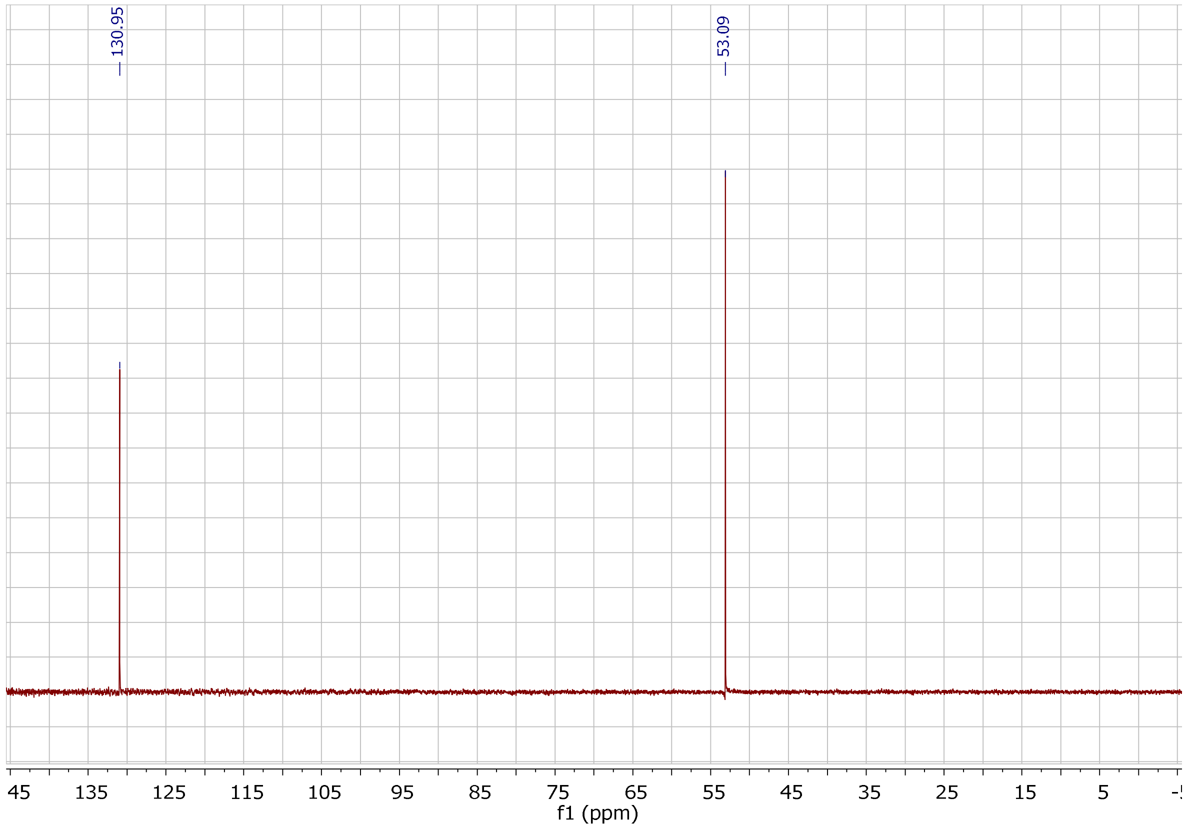

Figure S85. dept135 spectrum for dimethyl 4,5-dichlorophthalate (**5c**).

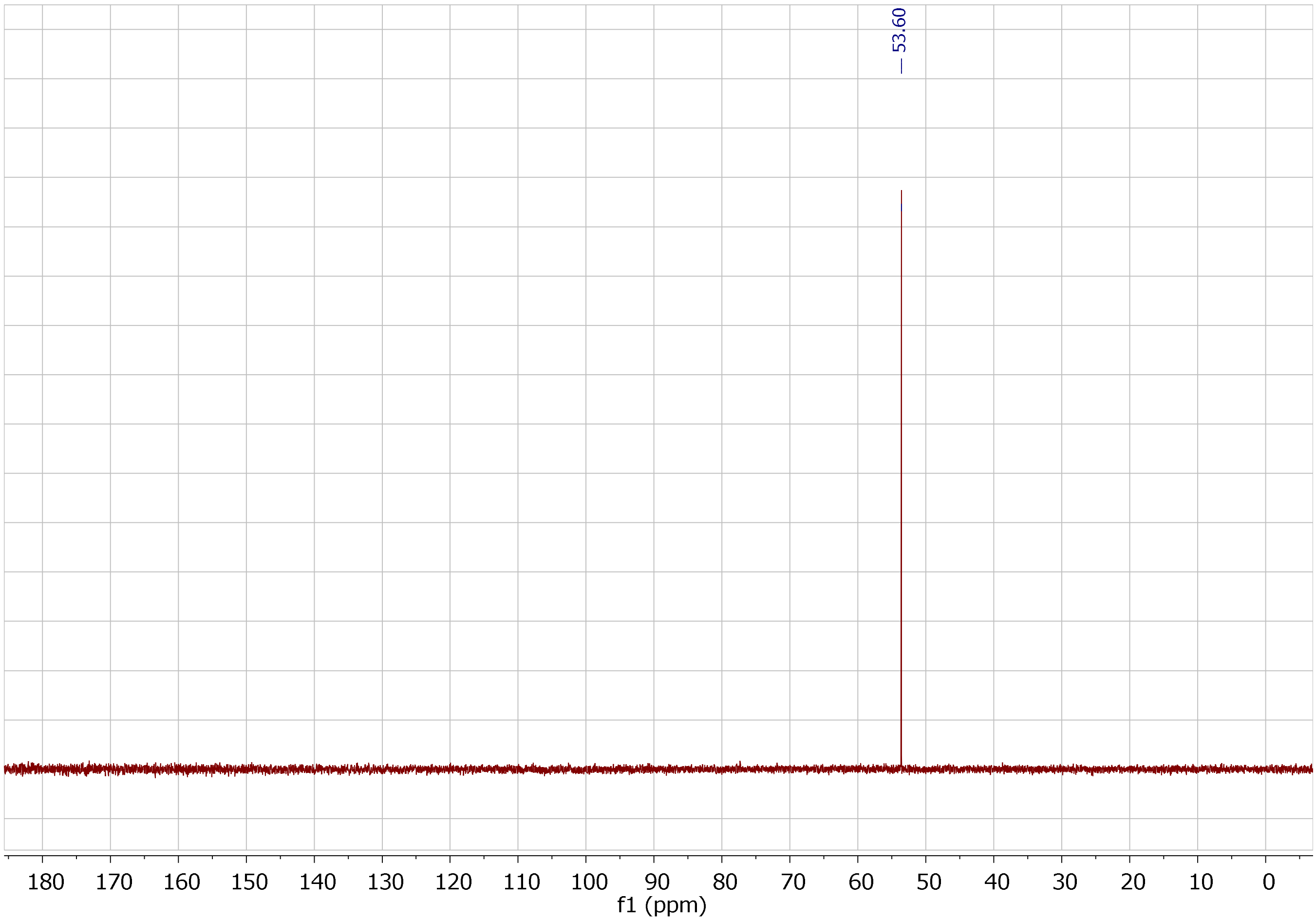

Figure S86. dept135 spectrum for dimethyl 3,4,5,6-tetrachlorophthalate (**5d**).

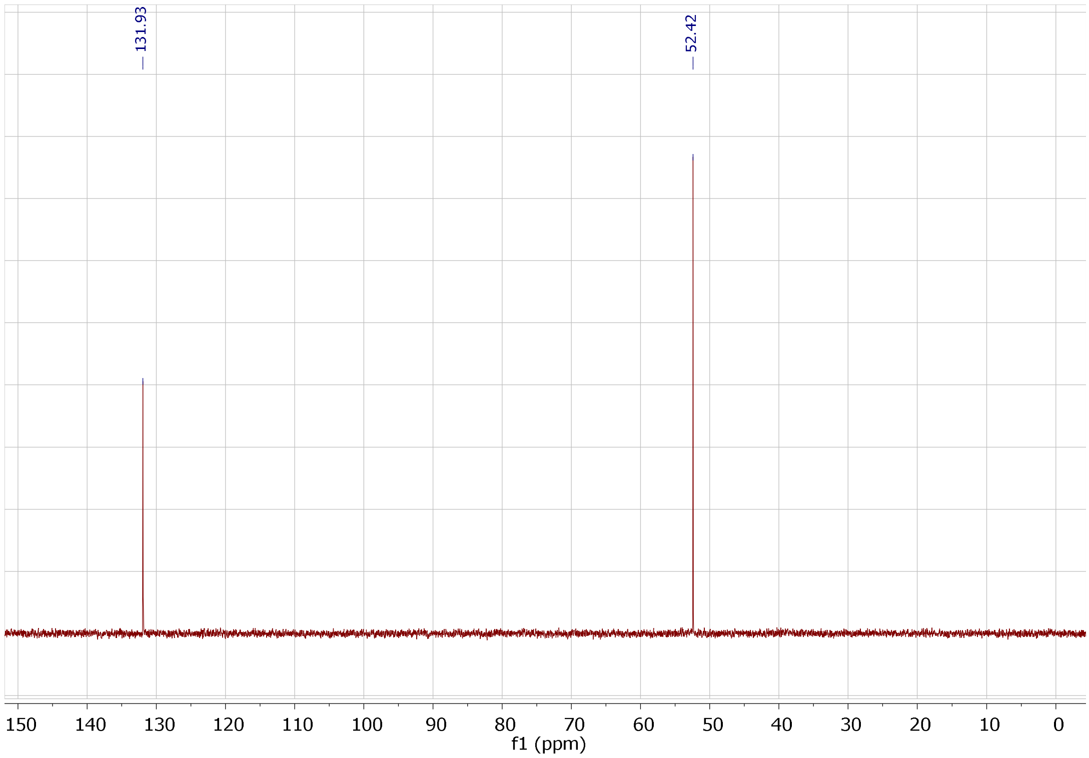

Figure S87. dept135 spectrum for dimethyl thiophene–3,4-dicarboxylate (**5e**).

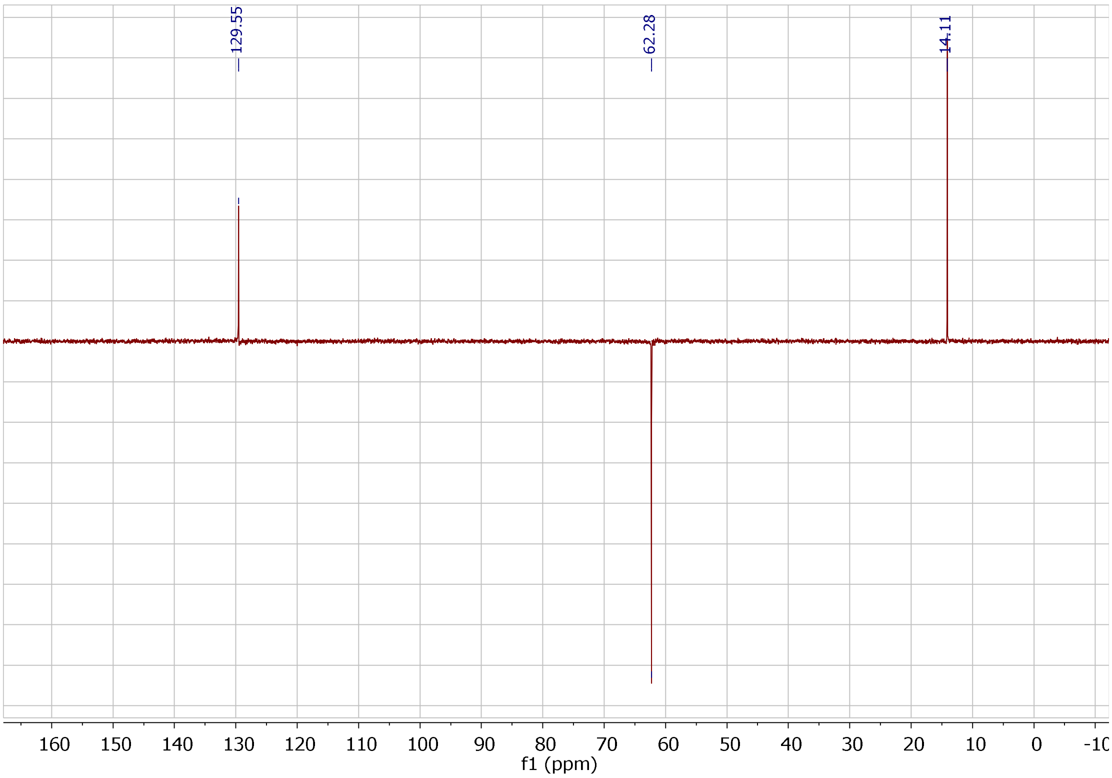

Figure S88. dept135 spectrum for tetraethyl benzene–1,2,4,5-tetracarboxylate (**8**).

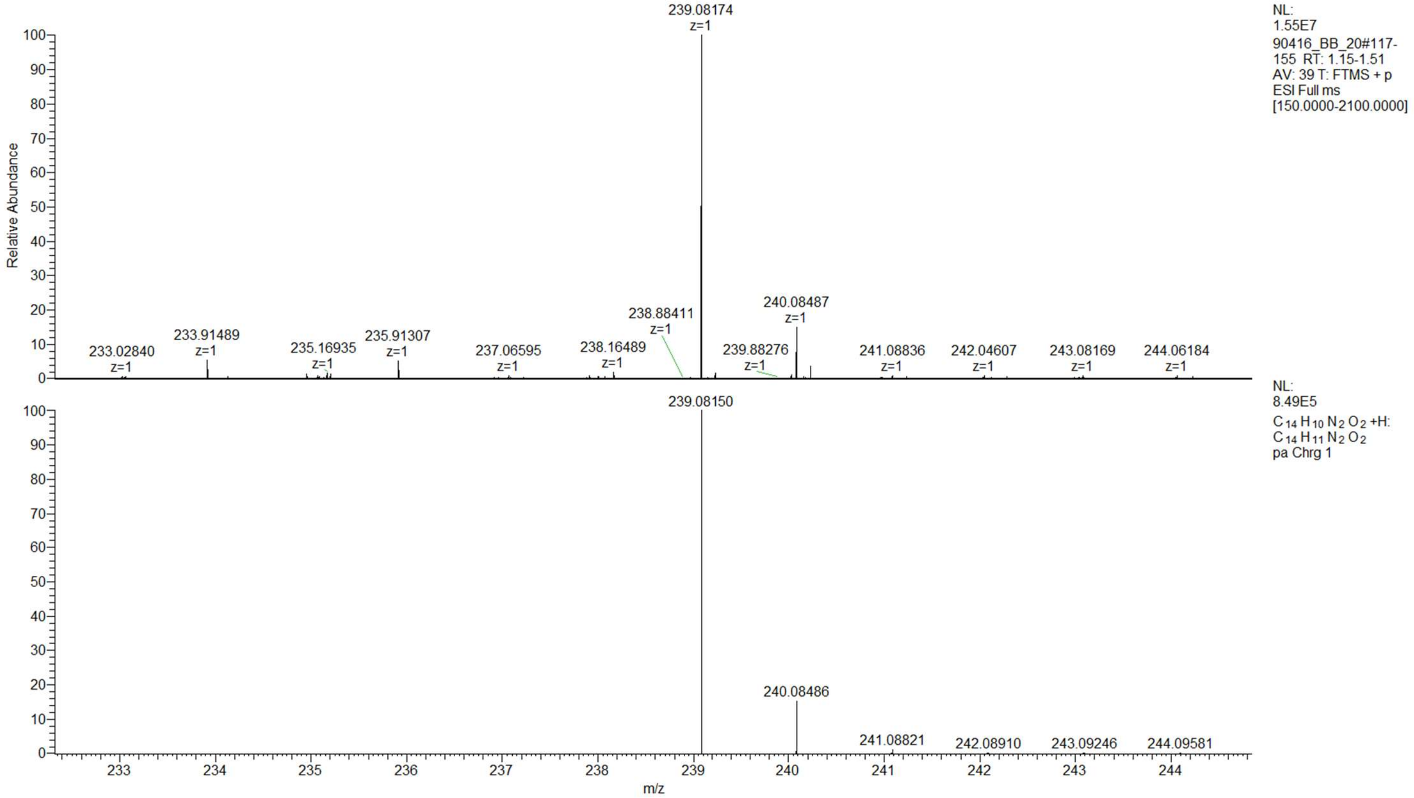

Figure S89. HRMS spectrum for 5,12-dihydrodibenzo[*b*,*f*][1,4]diazocine–6,11-dione (**3a**).

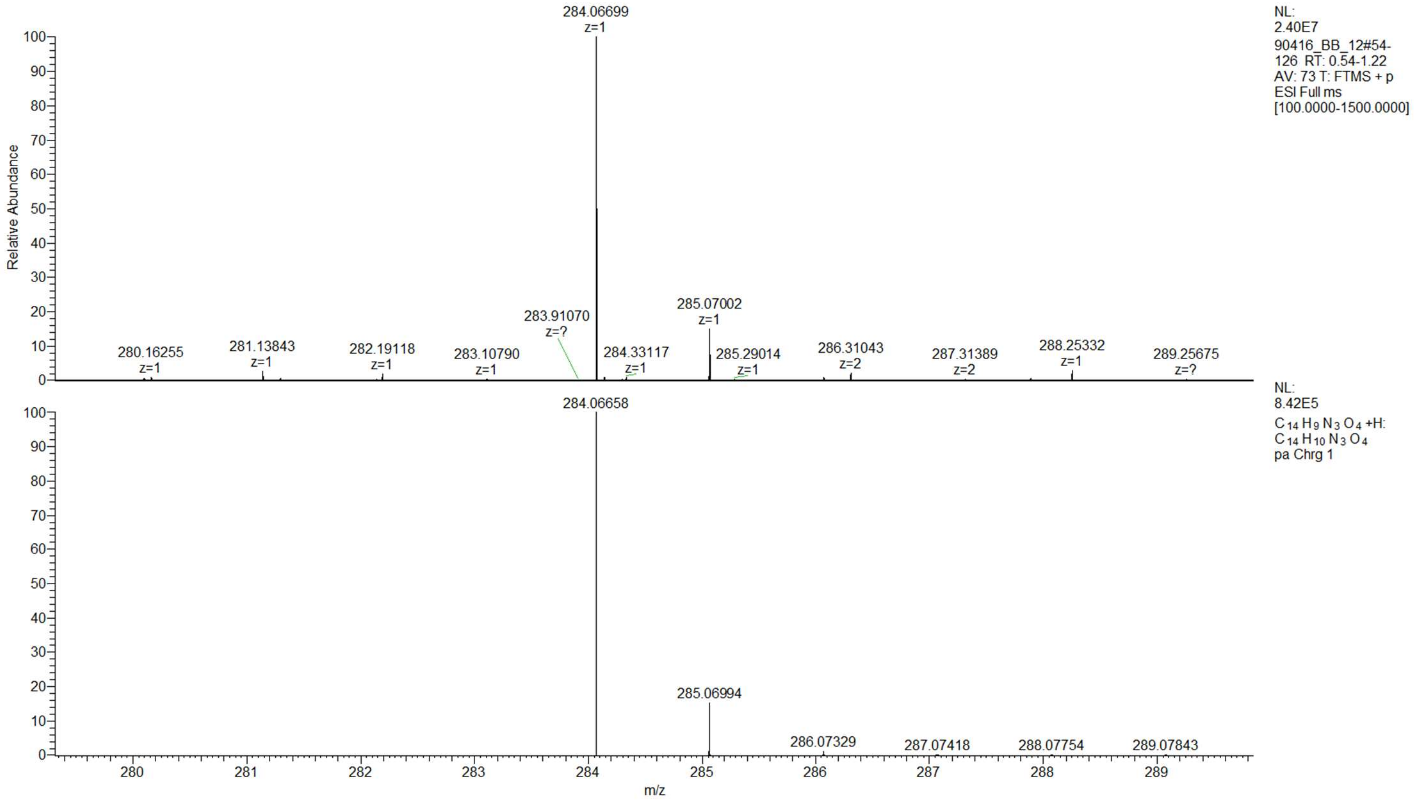

Figure S90. HRMS spectrum for 2-nitro–5,12-dihydrodibenzo[*b*,*f*][1,4]diazocine–6,11-dione (**3b**).

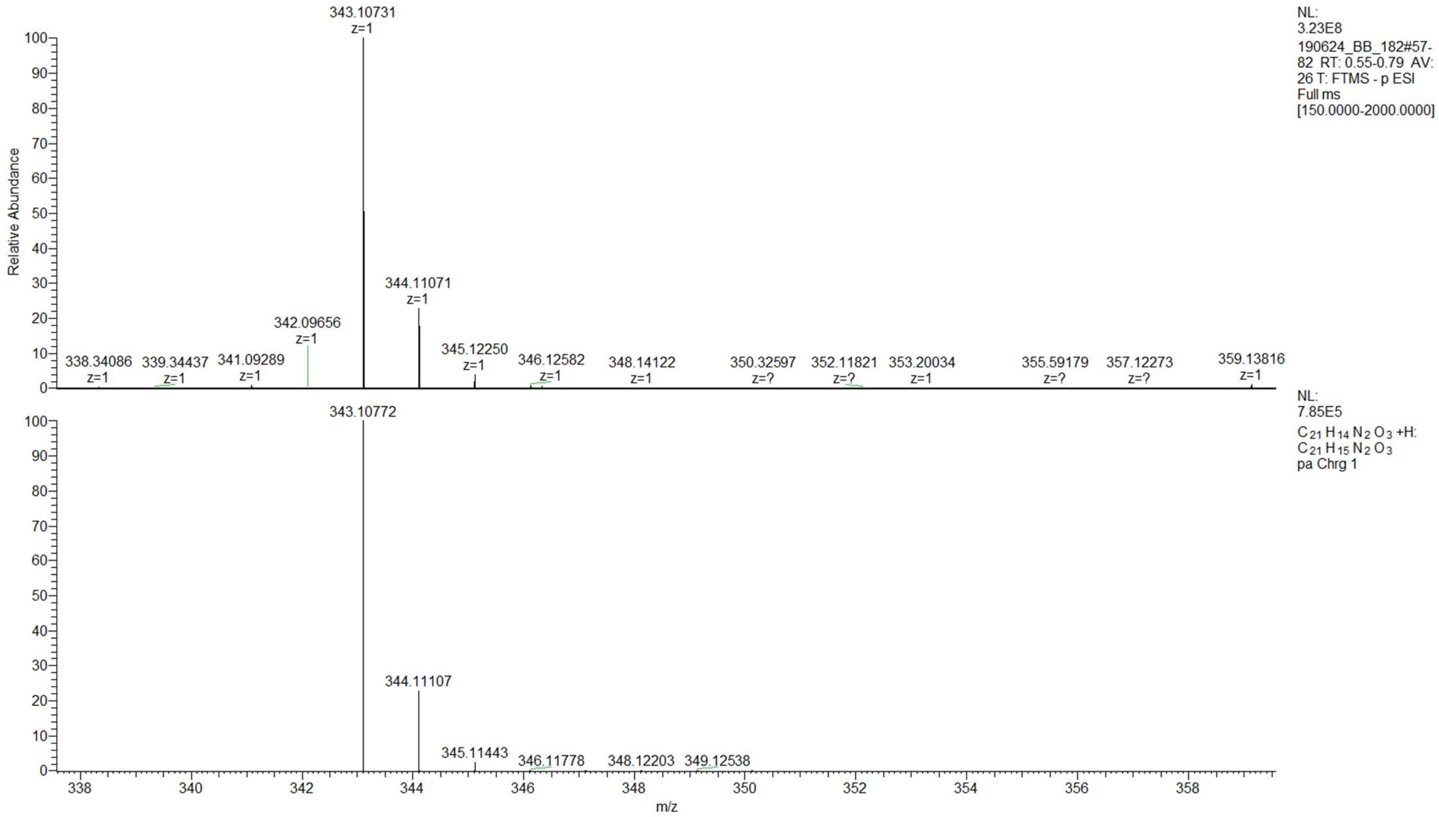

Figure S91. HRMS spectrum for 2-benzoyl–5,12-dihydrodibenzo[*b*,*f*][1,4]diazocine–6,11-dione (**3c**).

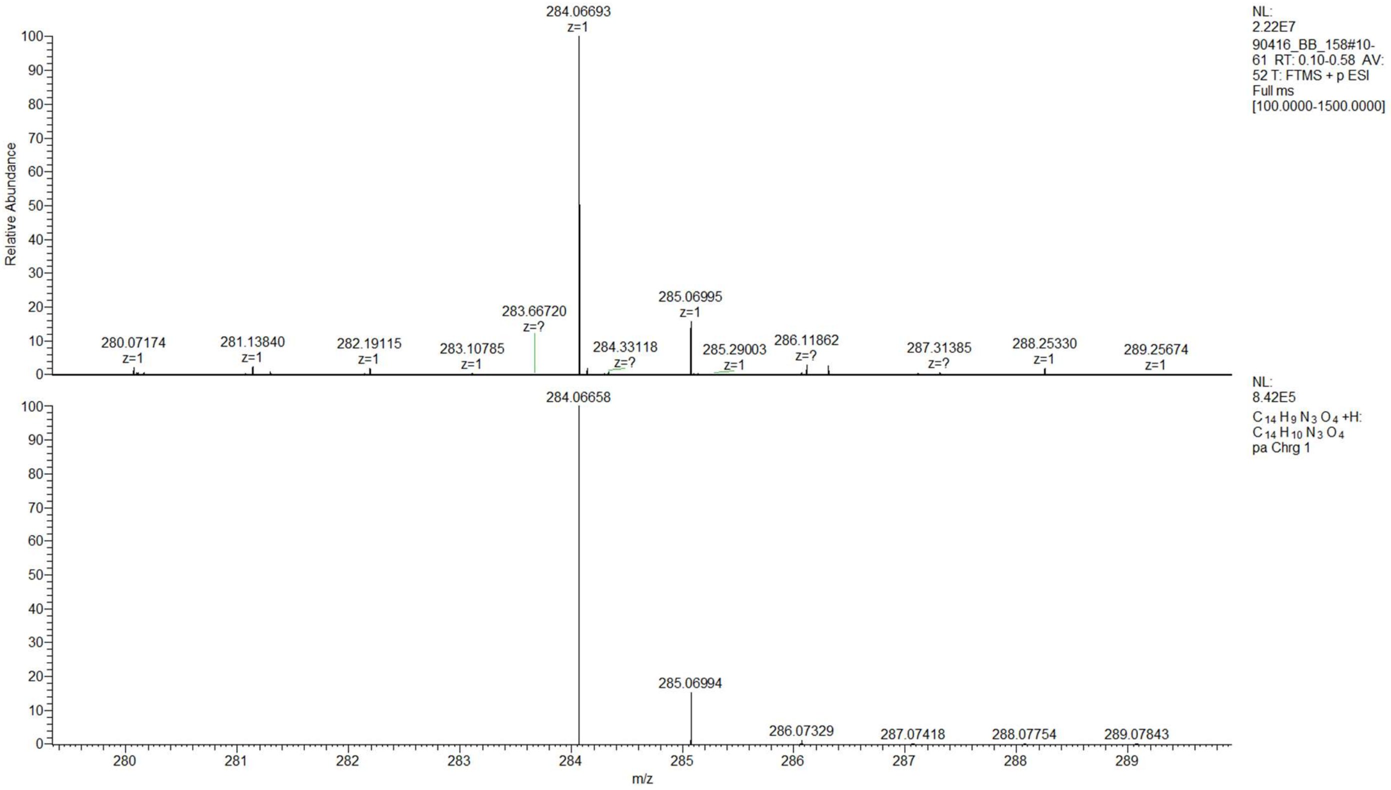

Figure S92. HRMS spectrum for 8-nitro–5,12-dihydrodibenzo[*b*,*f*][1,4]diazocine–6,11-dione (**3d**).

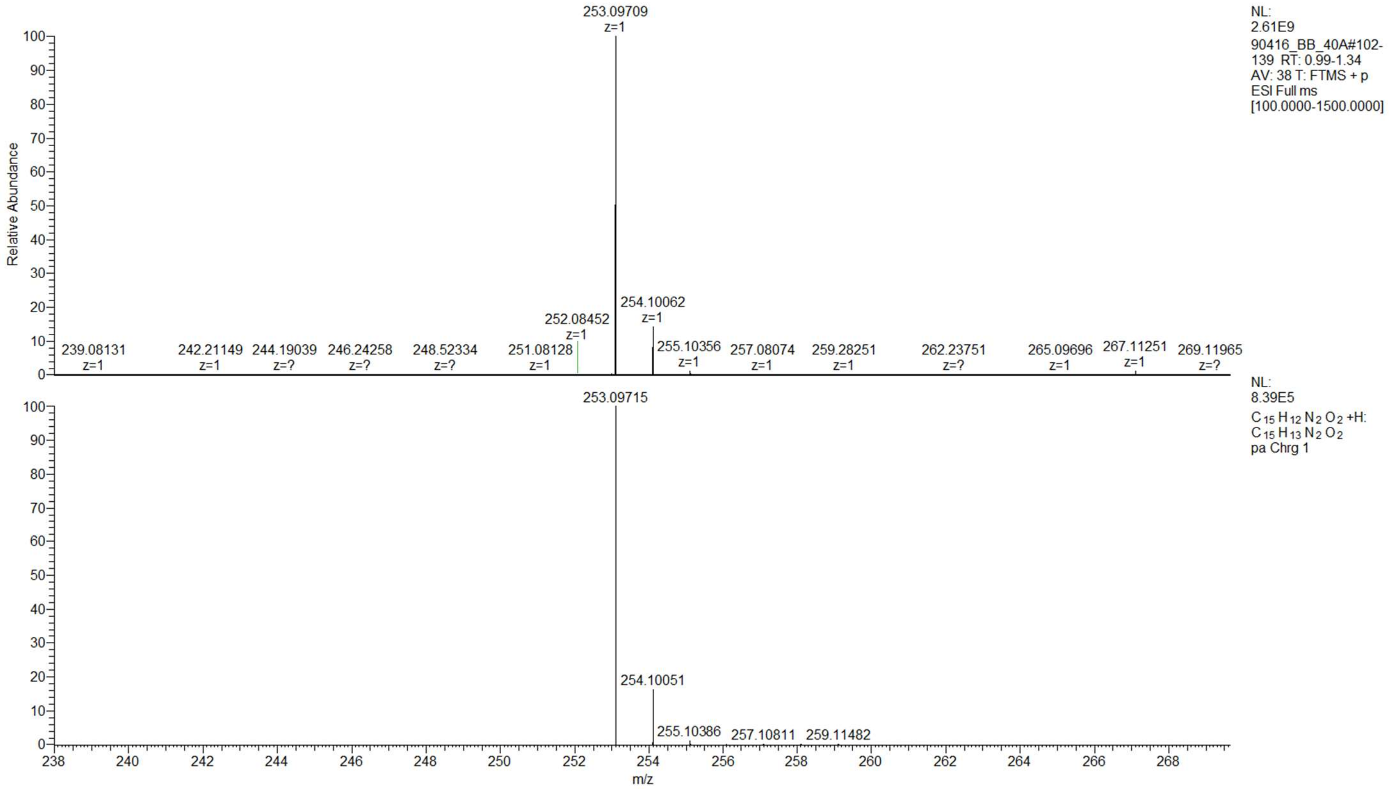

Figure S93. H-NMR spectrum for 5-methyl–5,12-dihydrodibenzo[*b*,*f*][1,4]diazocine–6,11-dione (**3e**).

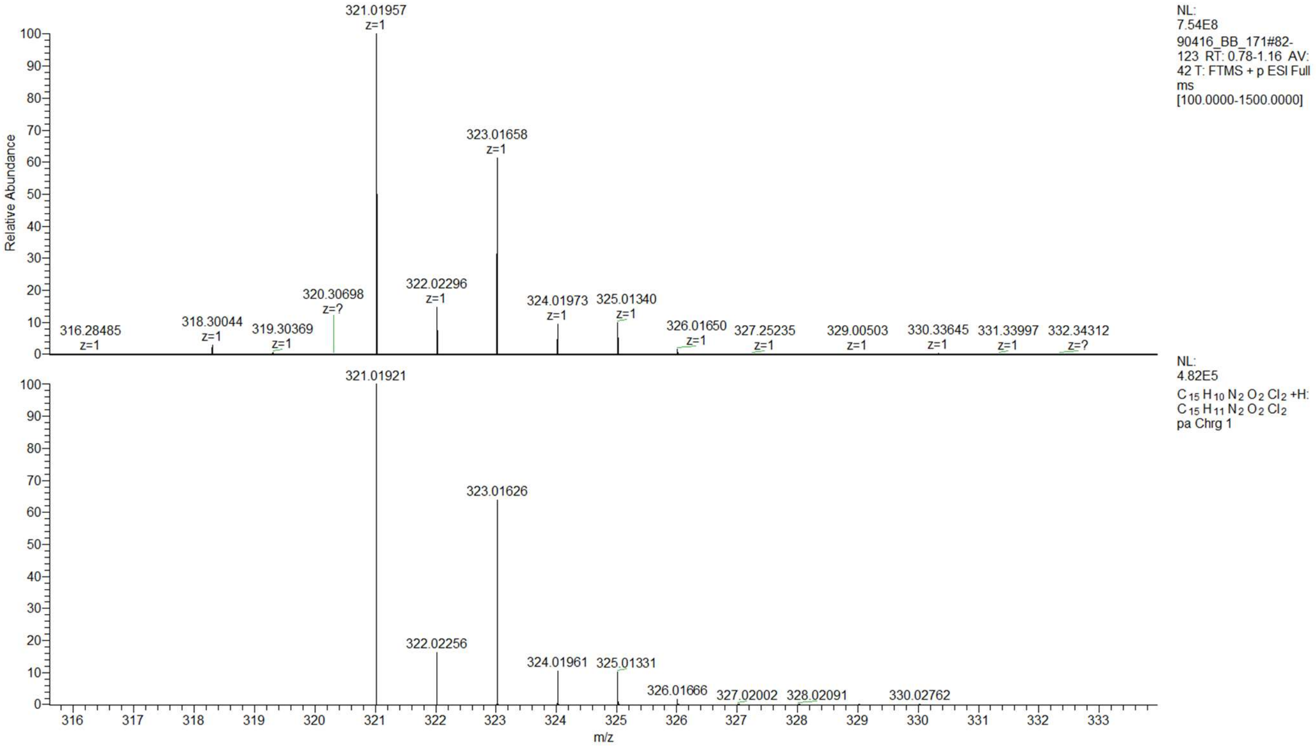

Figure S94. HRMS spectrum for 2,3-dichloro–5-methyl–5,12-dihydrodibenzo[*b*,*f*][1,4]diazocine–6,11-dione (**3f**).

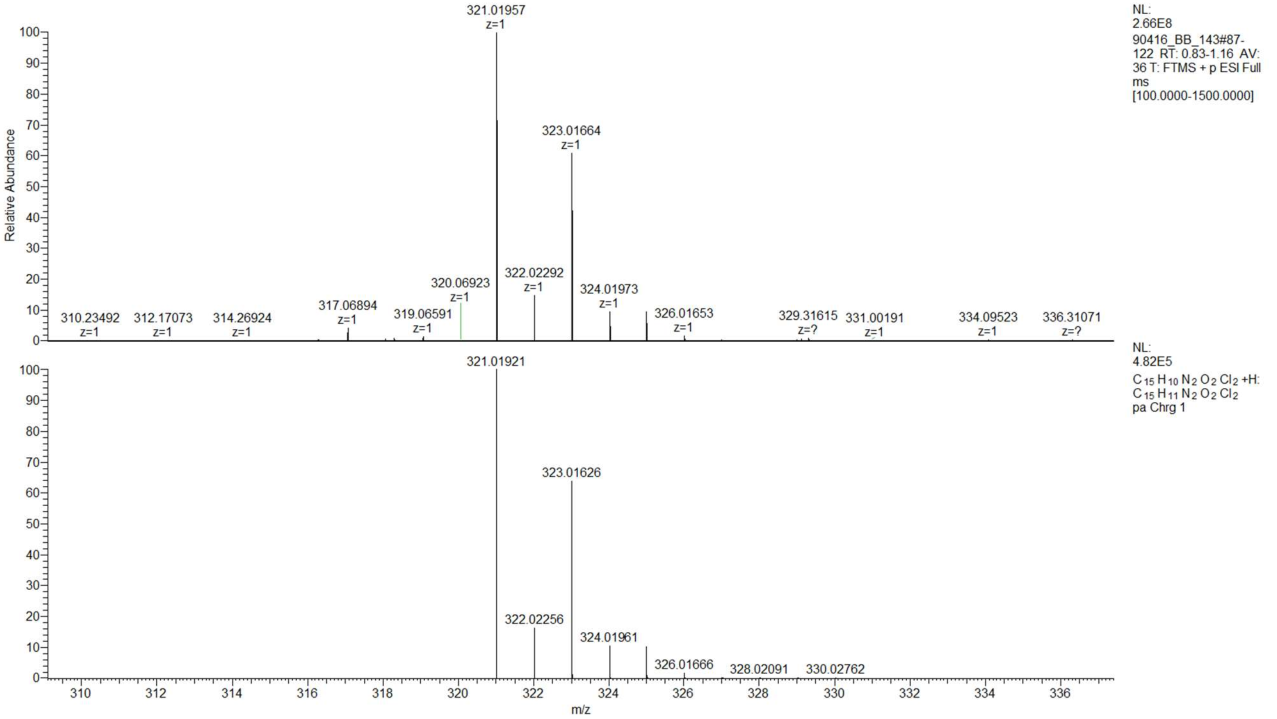

Figure S95. HRMS spectrum for 8,9-dichloro–5-methyl–5,12-dihydrodibenzo[*b*,*f*][1,4]diazocine–6,11-dione (**3g**).

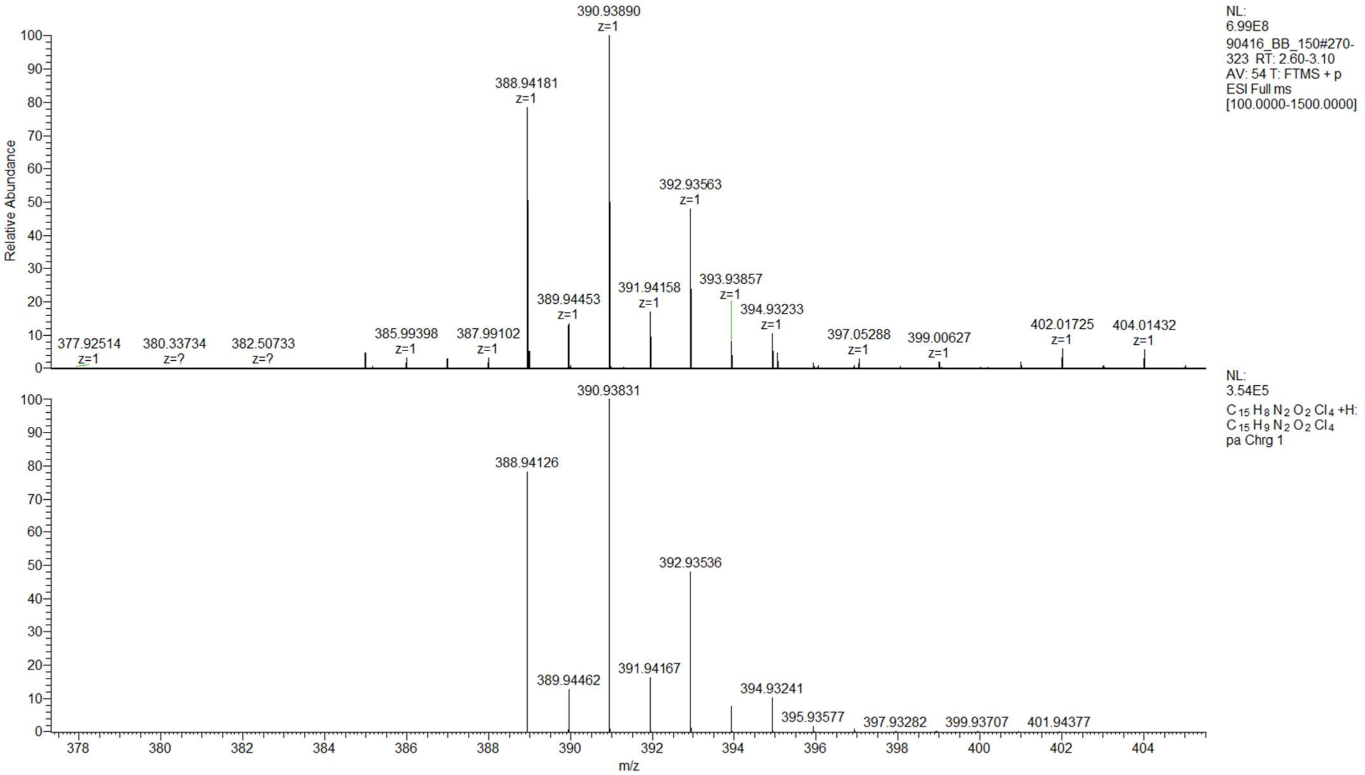

Figure S96. HRMS spectrum for 7,8,9,10-tetrachloro–5-methyl–5,12-dihydrodibenzo[*b,f*][1,4]diazocine–6,11-dione (**3h**).

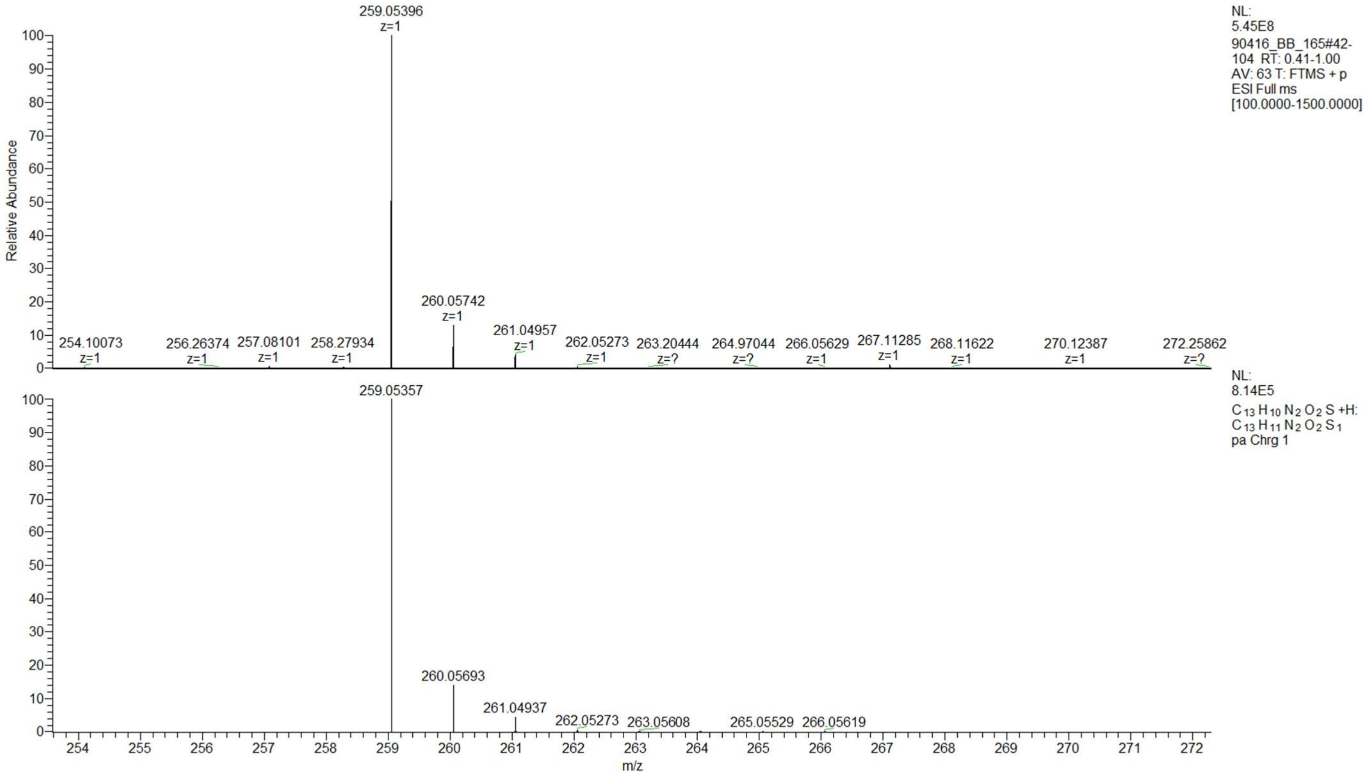

Figure S97. HRMS spectrum for 5-methyl–5,10-dihydrobenzo[*b*]thieno[3,4-*f*][1,4]diazocine–4,11-dione (**3i**).

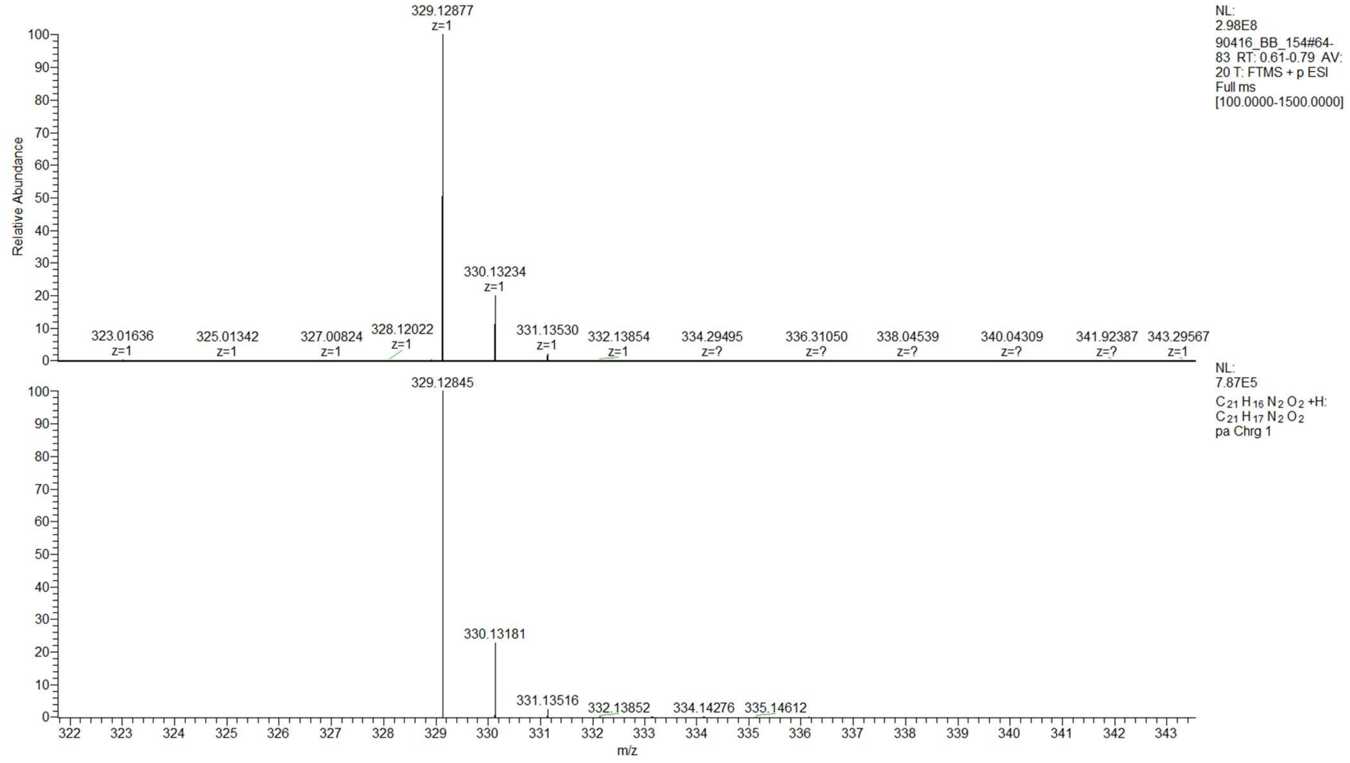

Figure S98. HRMS spectrum for 5-benzyl–5,12-dihydrodibenzo[*b*,*f*][1,4]diazocine–6,11-dione (**3j**).

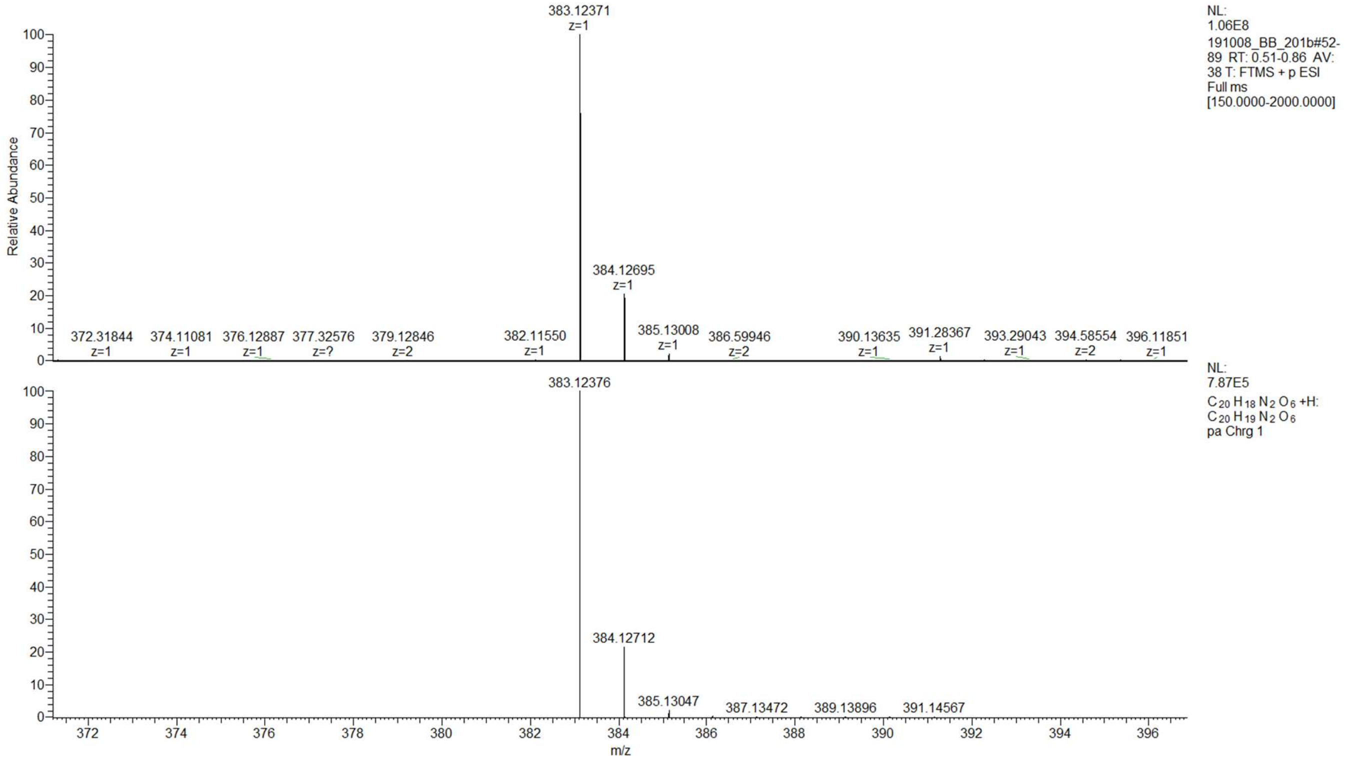

Figure S99. HRMS spectrum for diethyl 6,11-dioxo–5,6,11,12-tetrahydrodibenzo[*b*,*f*][1,4]diazocine–8,9-dicarboxylate (**3k**).

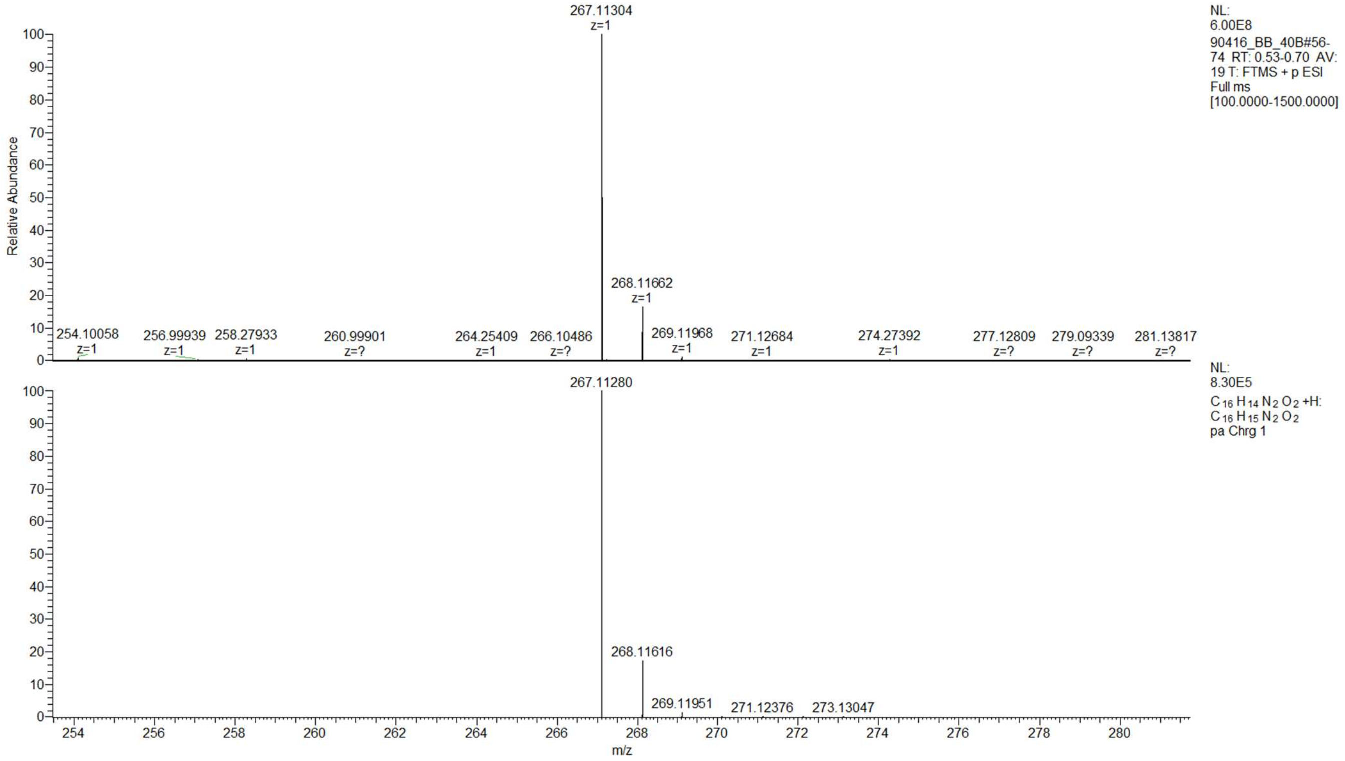

Figure S100. HRMS spectrum for 5,12-dimethyl–5,12-dihydrodibenzo[*b*,*f*][1,4]diazocine–6,11-dione (**3l**).

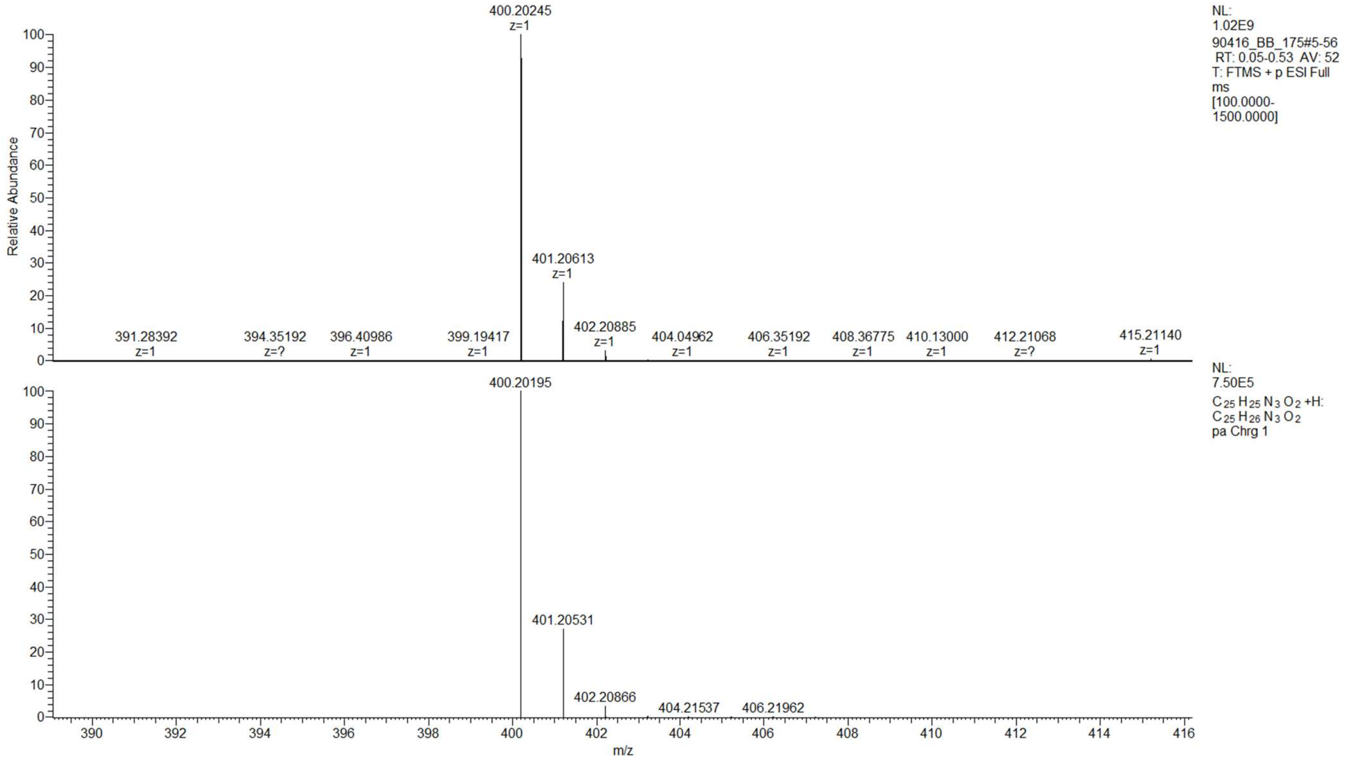

Figure S101. HRMS spectrum for 5-benzyl–12-(2-(dimethylamino)ethyl)–5,12-dihydrodibenzo[*b*,*f*][1,4]diazocine–6,11-dione (**3m**).

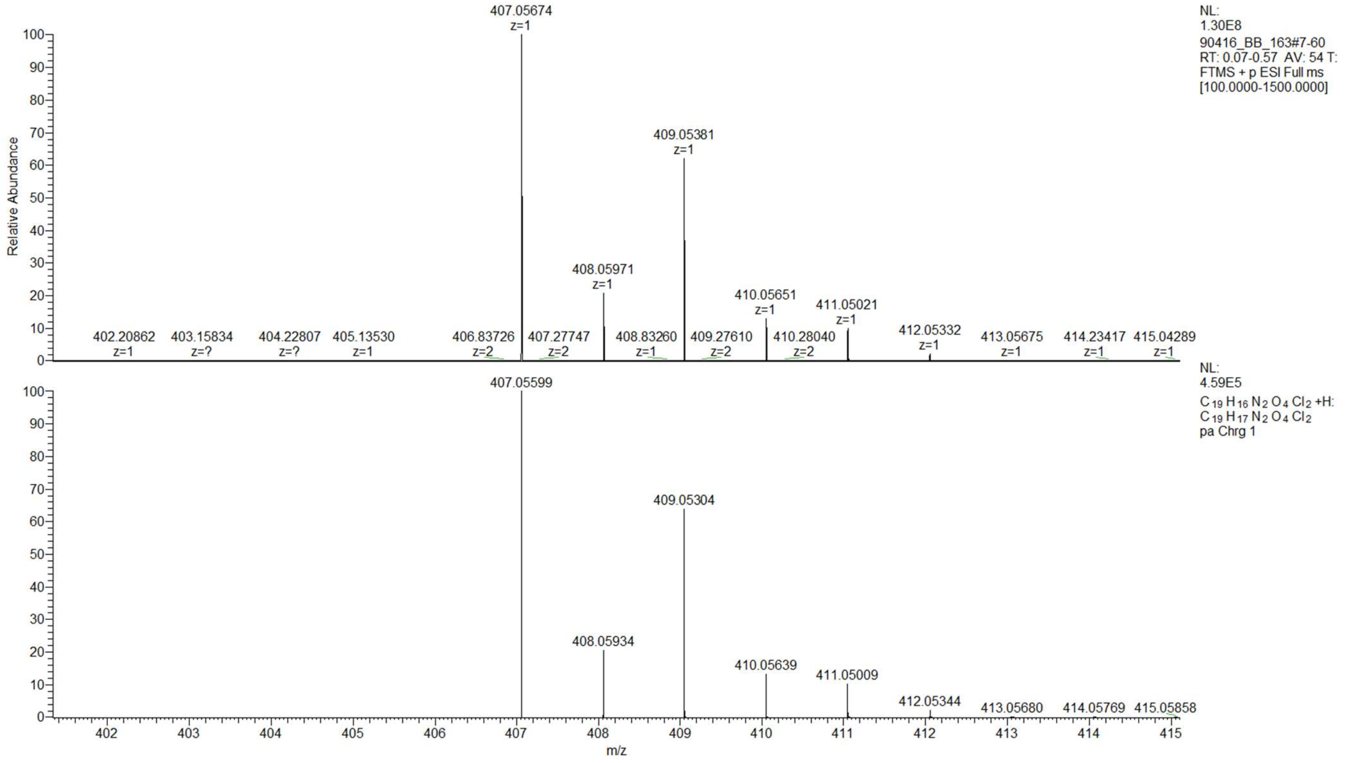

Figure S102. HRMS spectrum for ethyl 2-(8,9-dichloro–12-methyl–6,11-dioxo–11,12-dihydrodibenzo[*b*,*f*][1,4]diazocin–5(6*H*)-yl)acetate (**3n**).

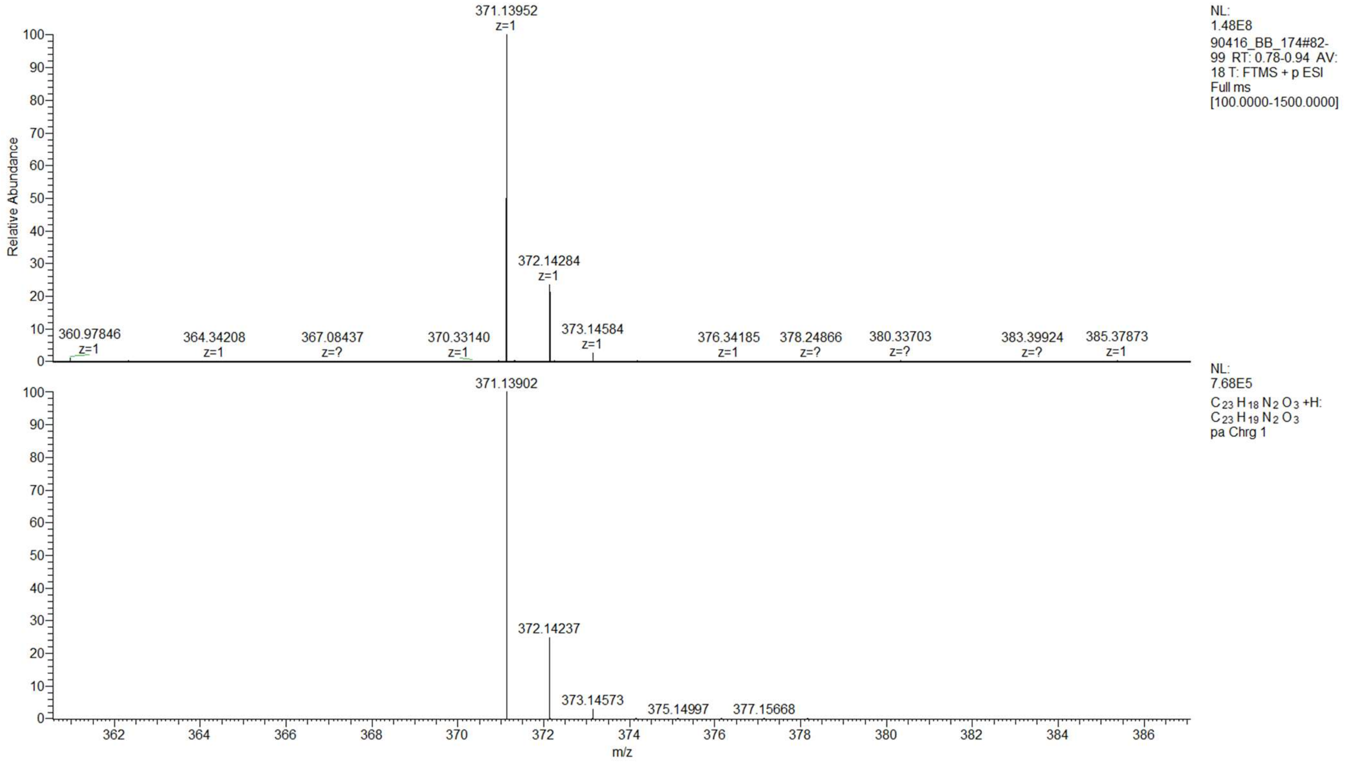

Figure S103. HRMS spectrum for 5-acetyl–12-benzyl–5,12-dihydrodibenzo[*b*,*f*][1,4]diazocine–6,11-dione (**3o**).

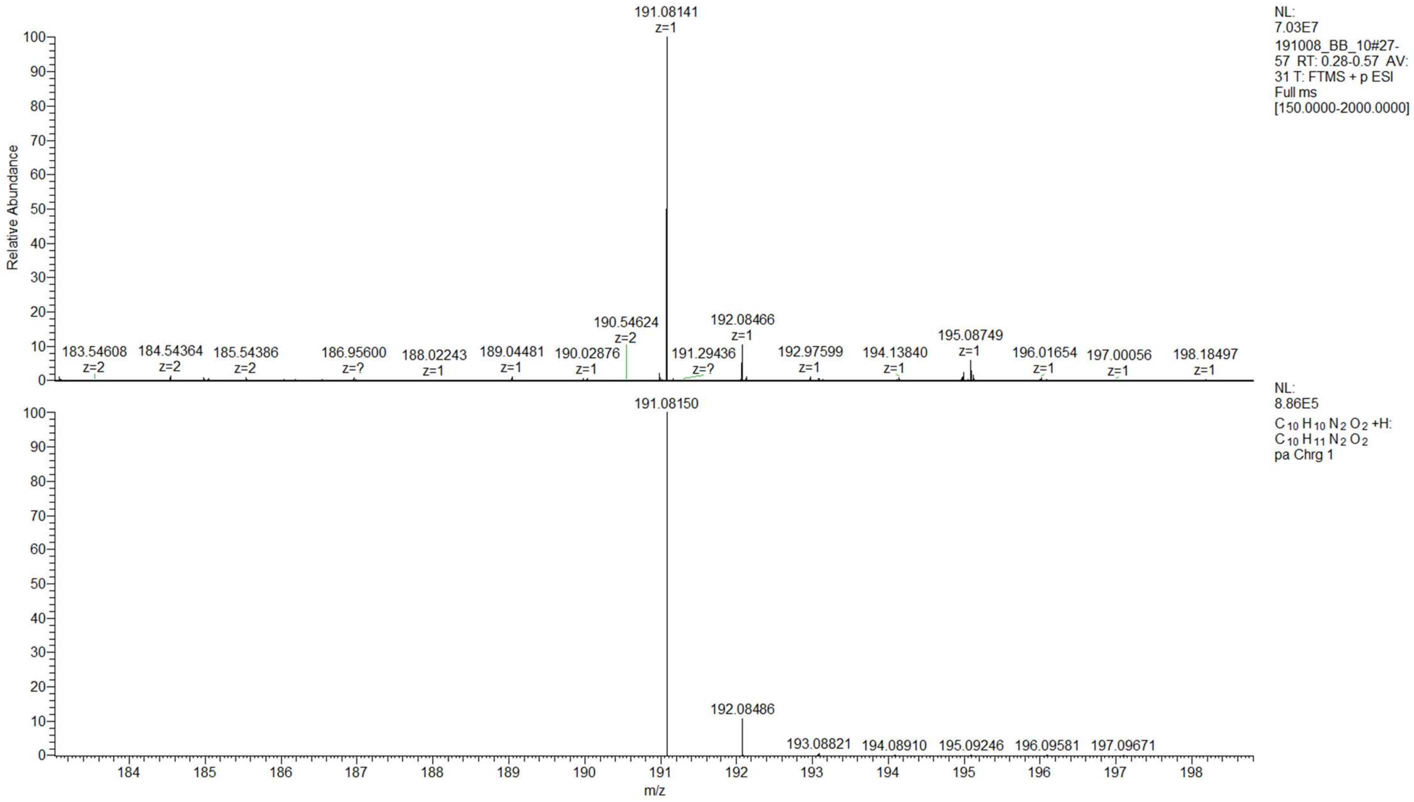

Figure S104. HRMS spectrum for 1,3,4,6-tetrahydrobenzo[*b*][1,4]diazocine–2,5-dione (**6**).

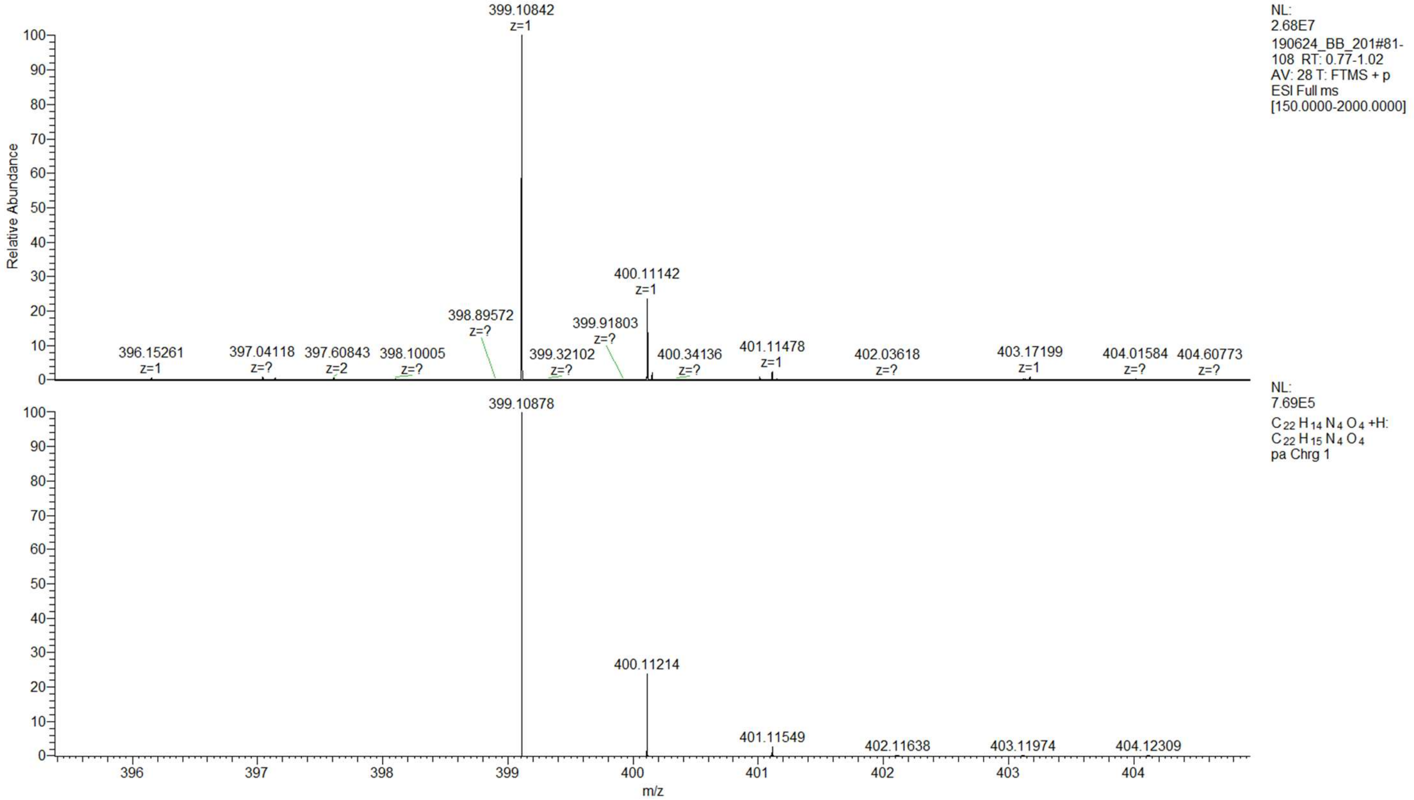

Figure S105. HRMS spectrum for **9a.**

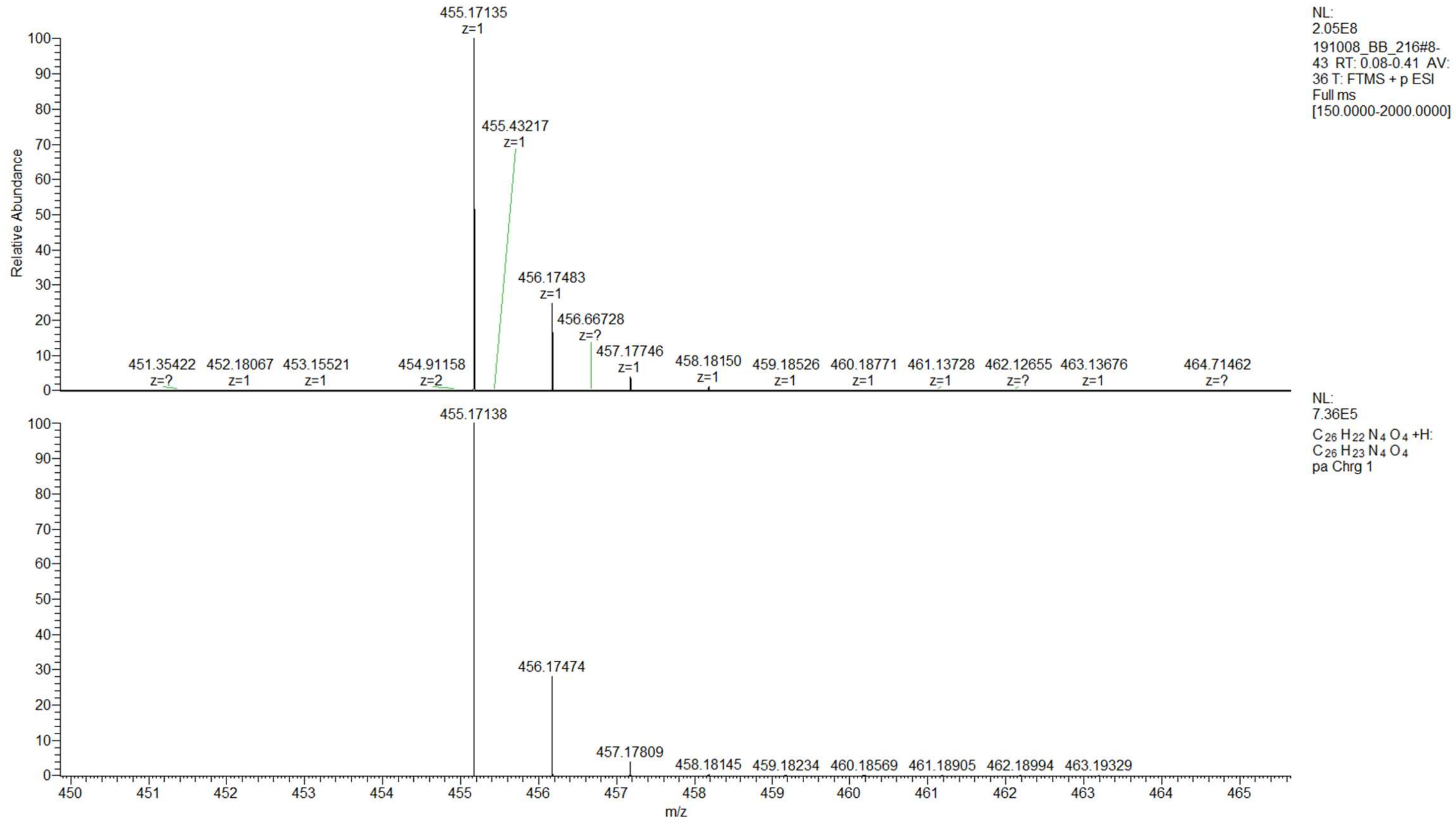

Figure S106. HRMS spectrum for **9b.**

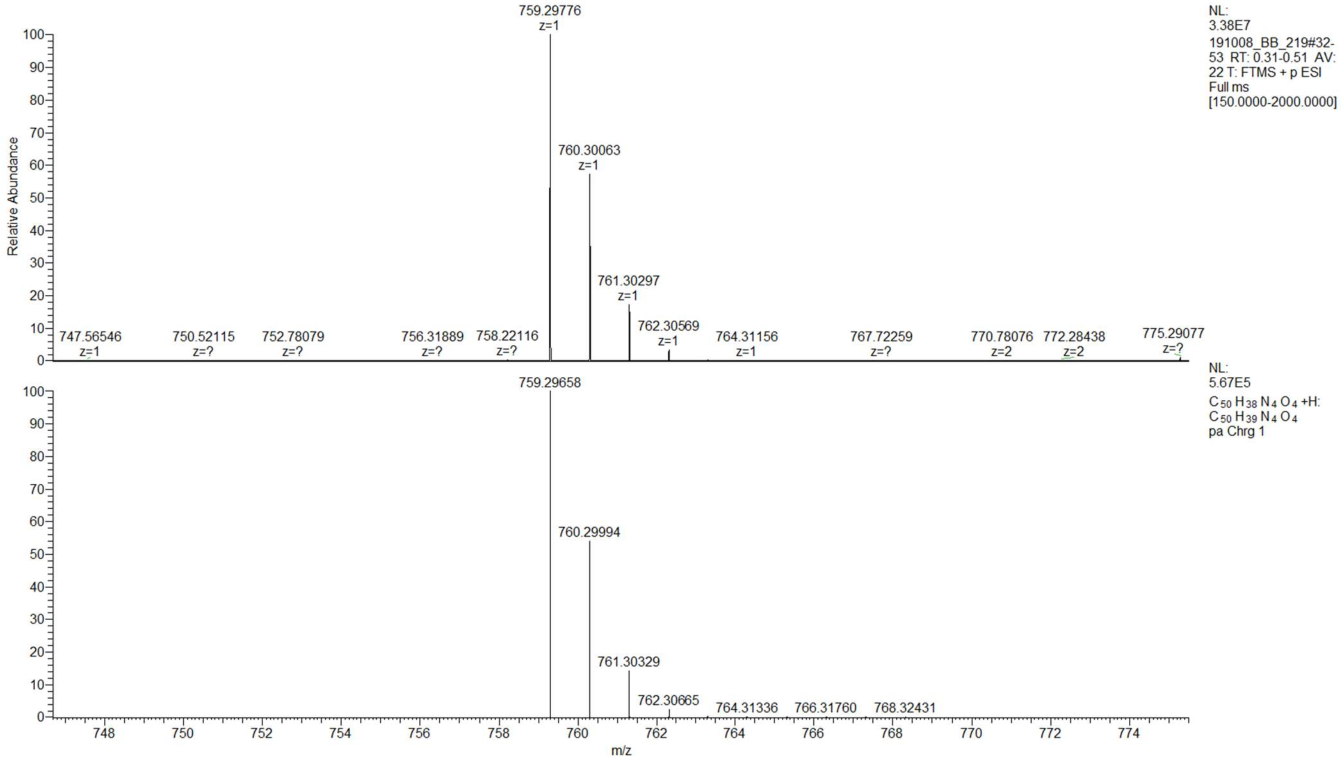

Figure S107. HRMS spectrum for **9c.**

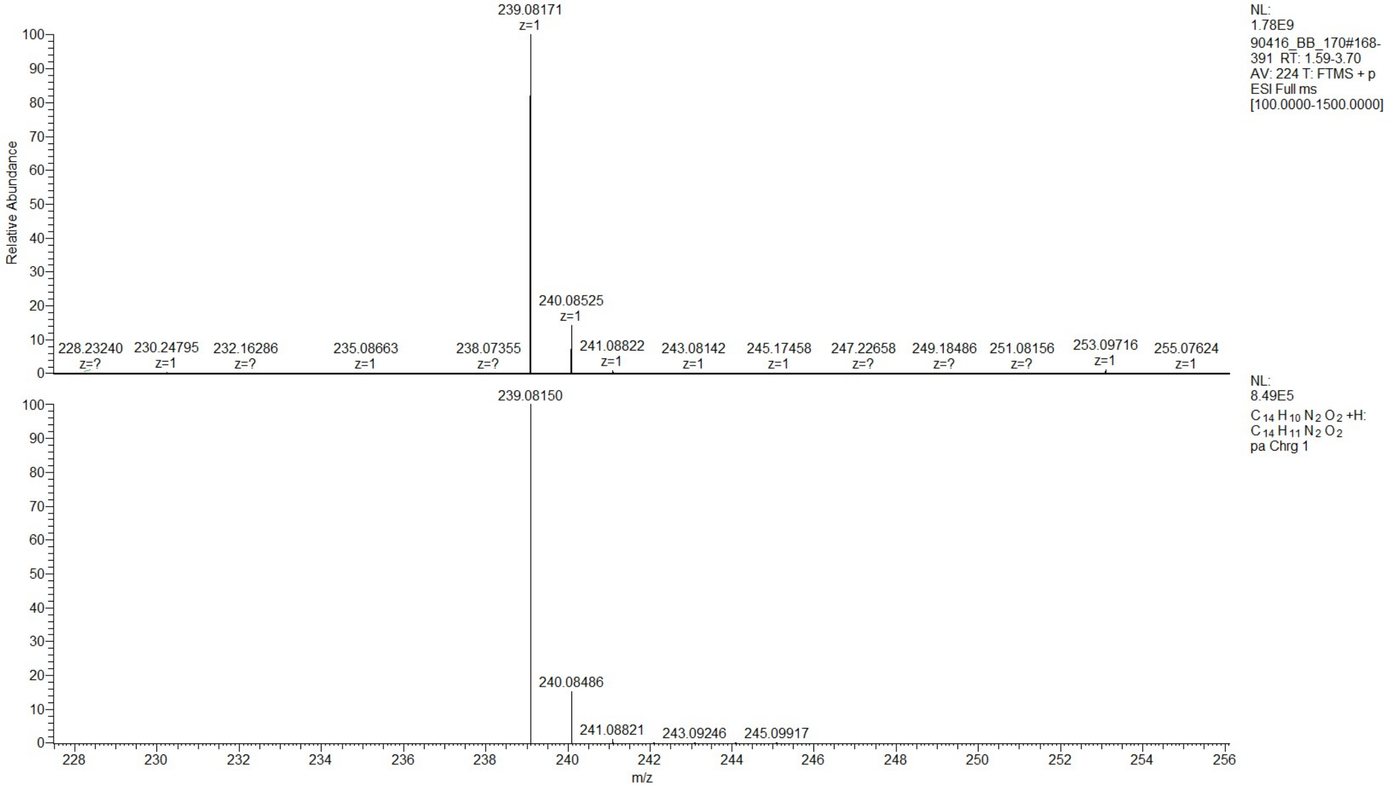

Figure S108. HRMS spectrum for 2-(2-aminophenyl)isoindoline–1,3-dione (**10**).

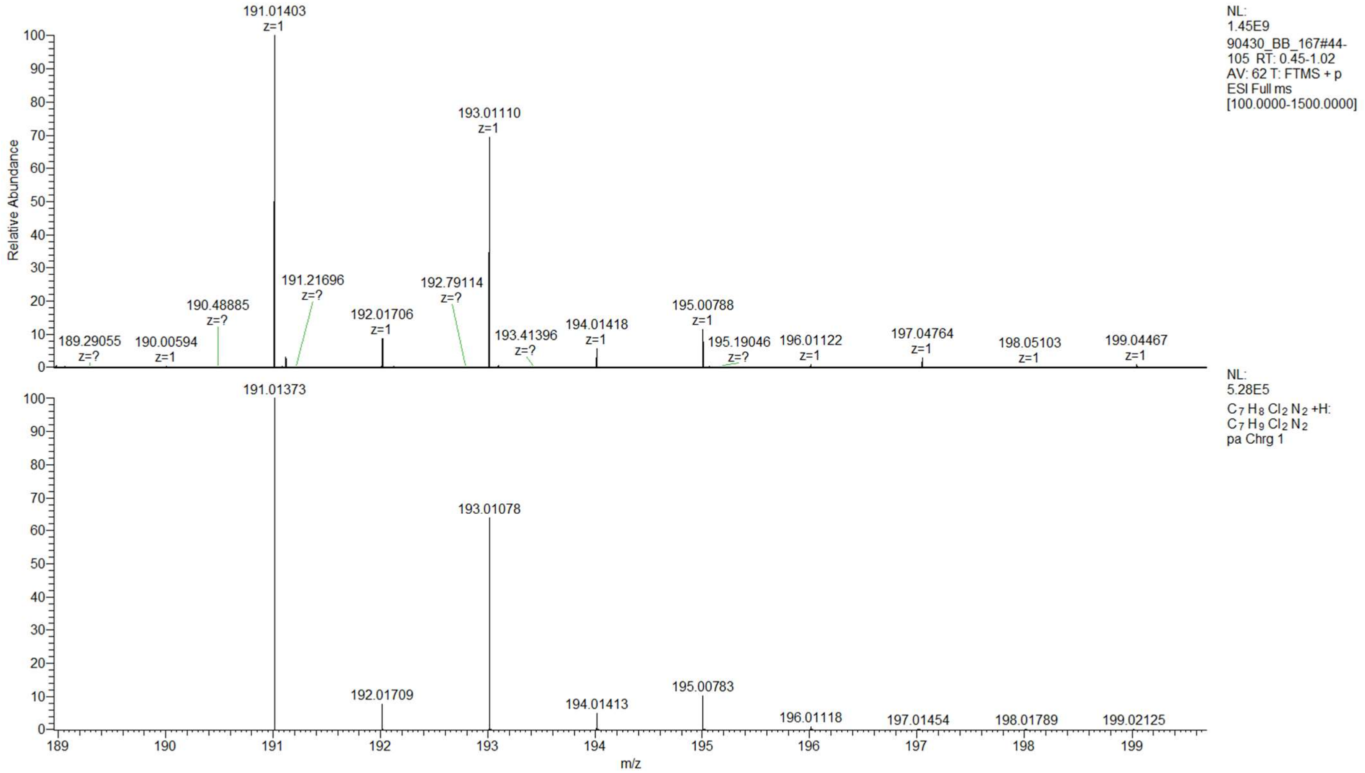

Figure S109. HRMS spectrum for 4,5-dichloro-*N*[1]-methylbenzene–1,2-diamine (**4e**).

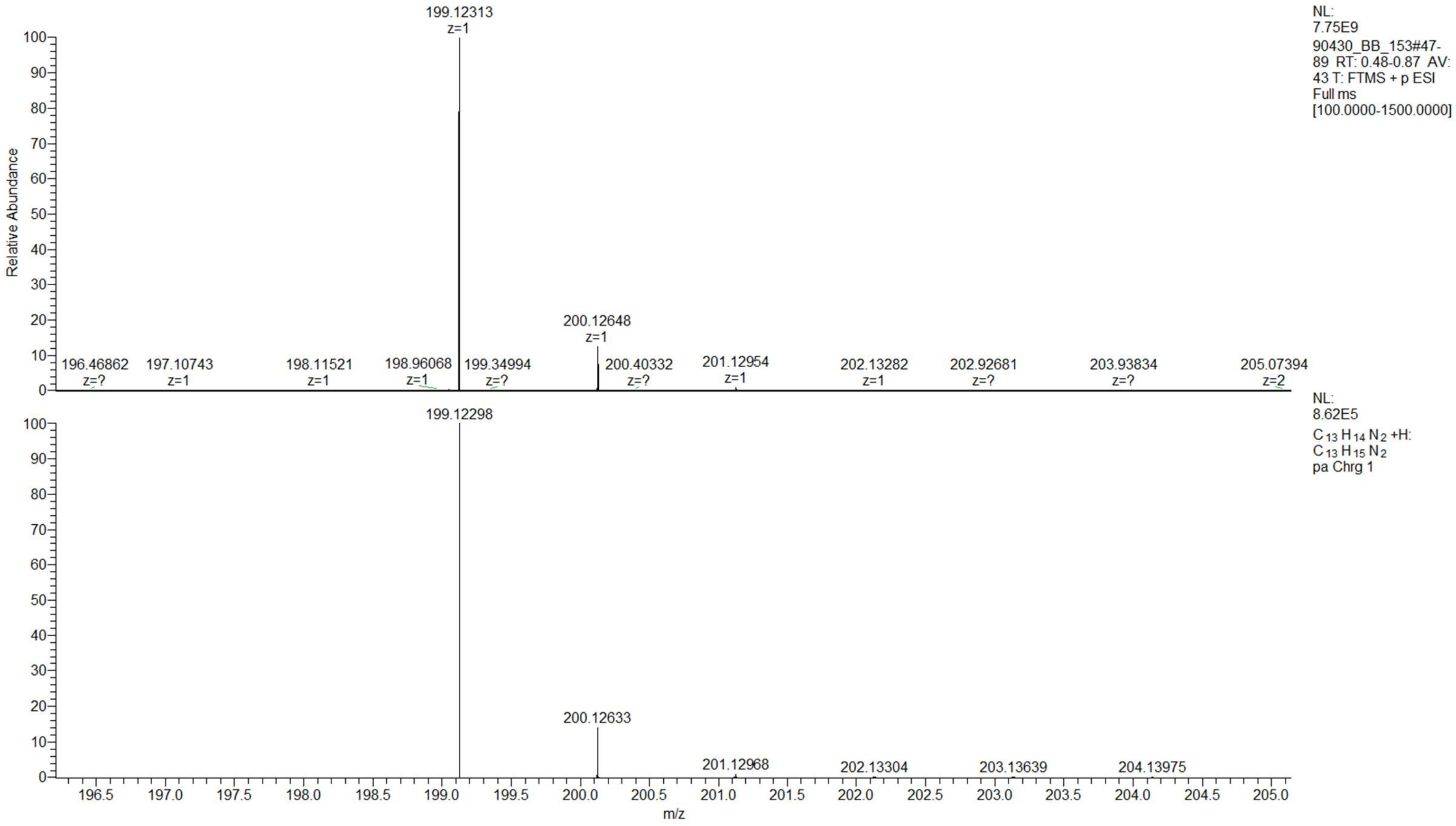

Figure S110. HRMS spectrum for *N*[1]-benzylbenzene–1,2-diamine (**4f**).

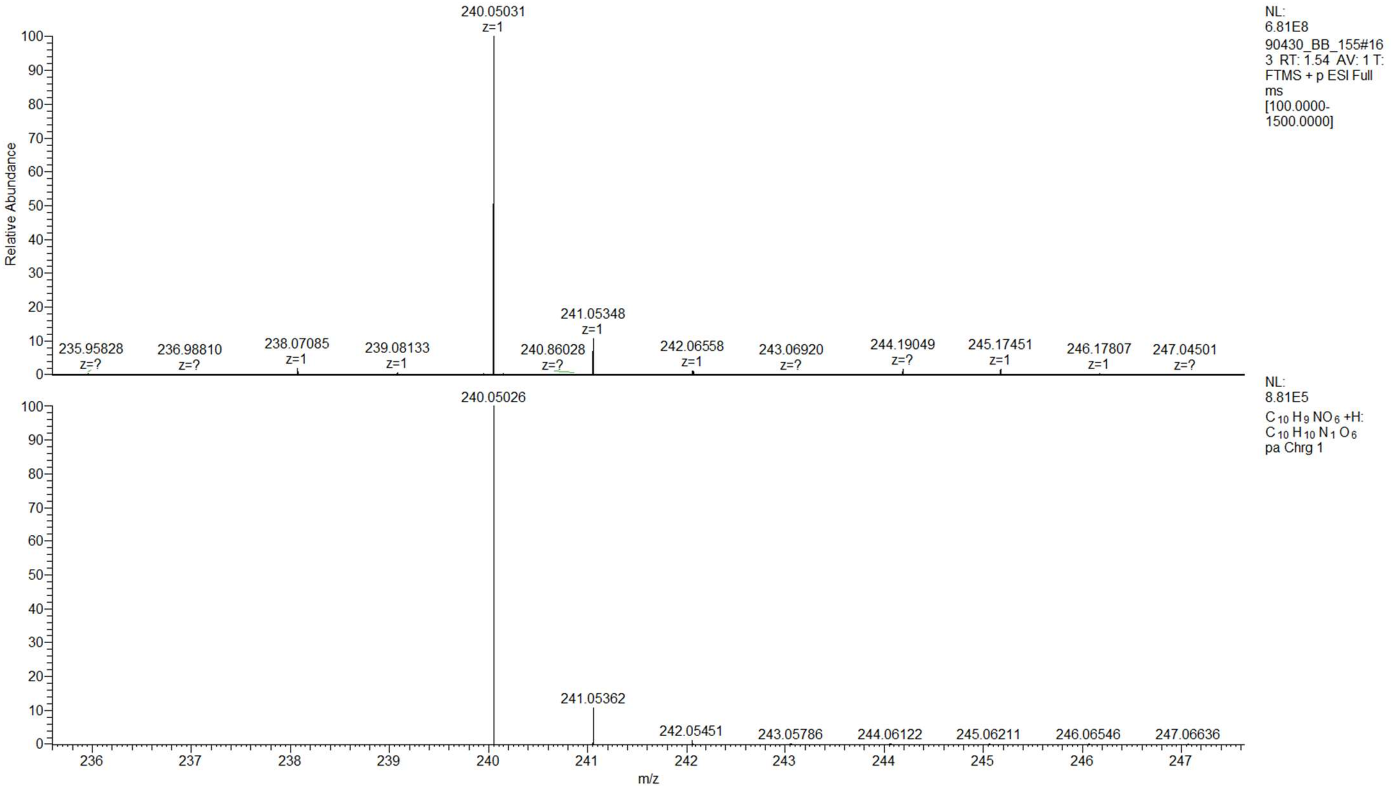

Figure S111. HRMS spectrum for dimethyl 4-nitrophthalate (**5b**).

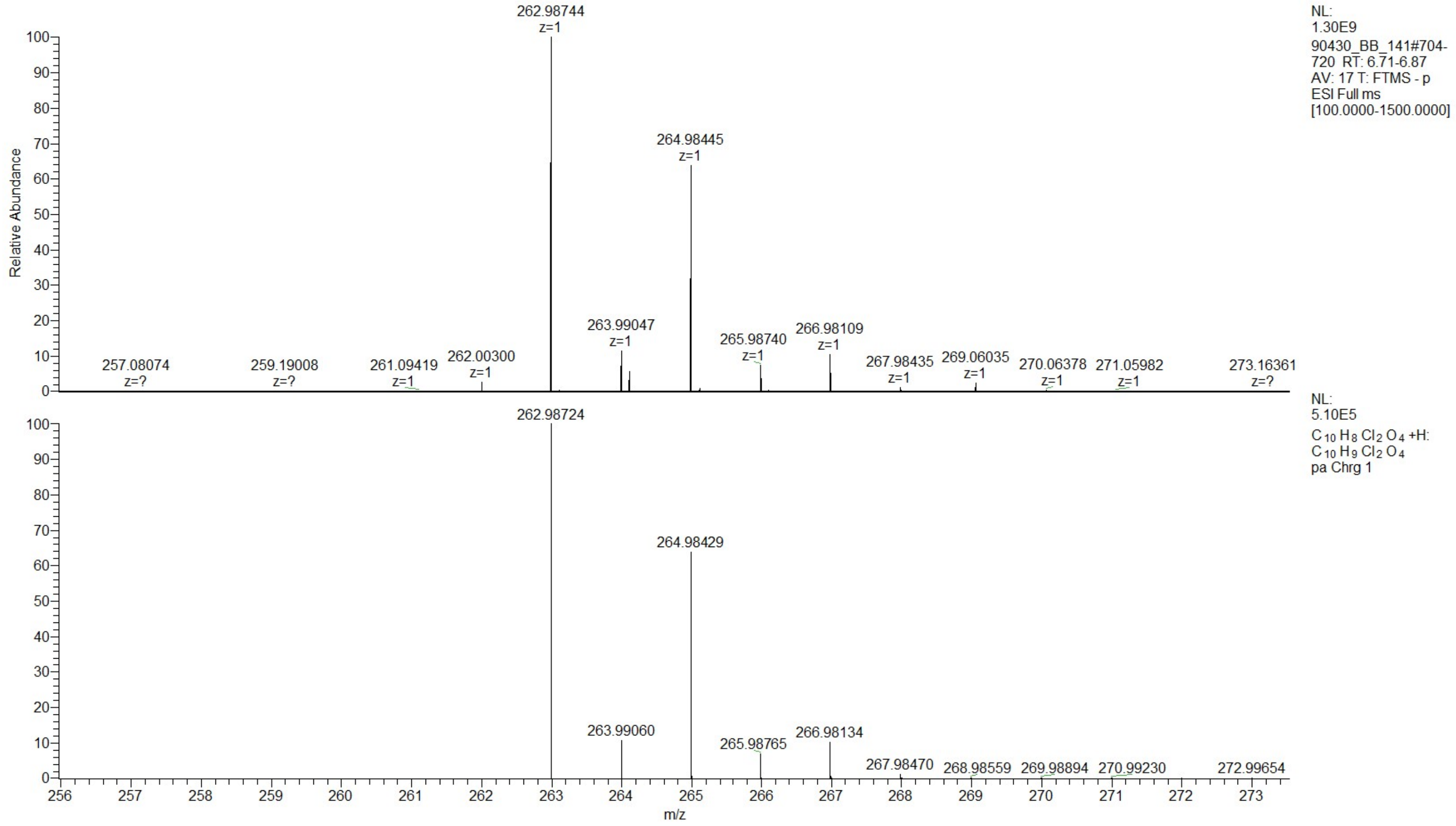

Figure S112. HRMS spectrum for dimethyl 4,5-dichlorophthalate (**5c**).

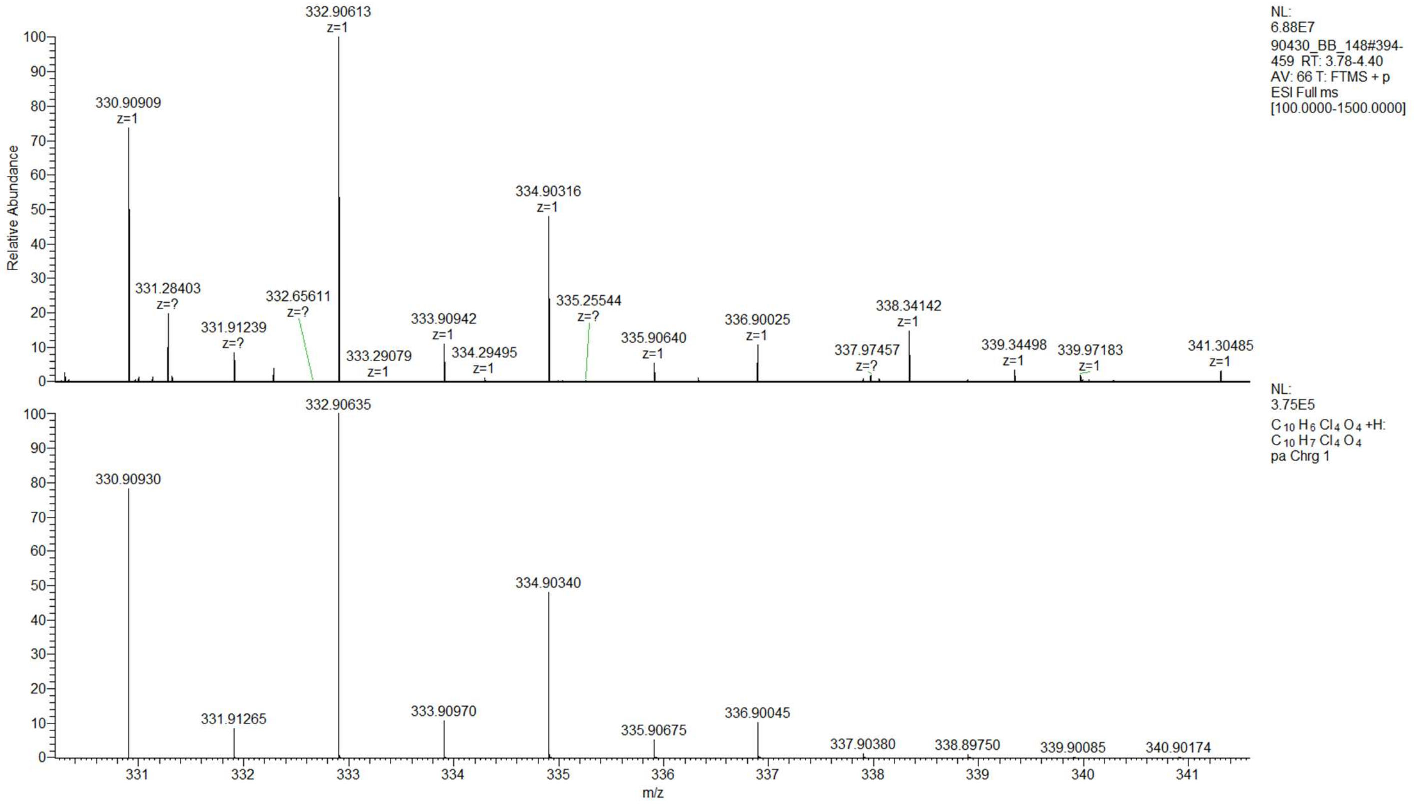

Figure S113. HRMS spectrum for dimethyl 3,4,5,6-tetrachlorophthalate (**5d**).

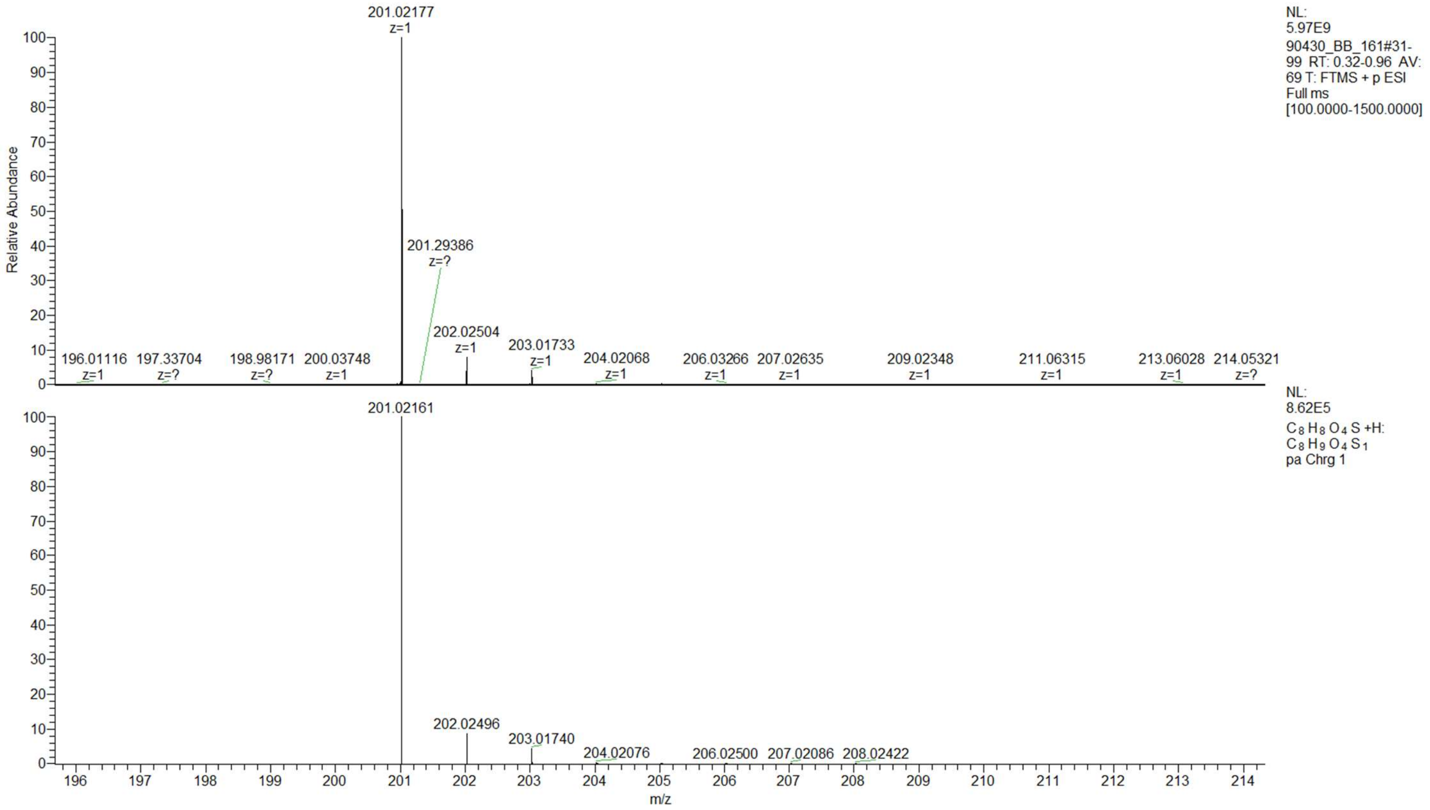

Figure S114. HRMS spectrum for dimethyl thiophene–3,4-dicarboxylate (**5e**).

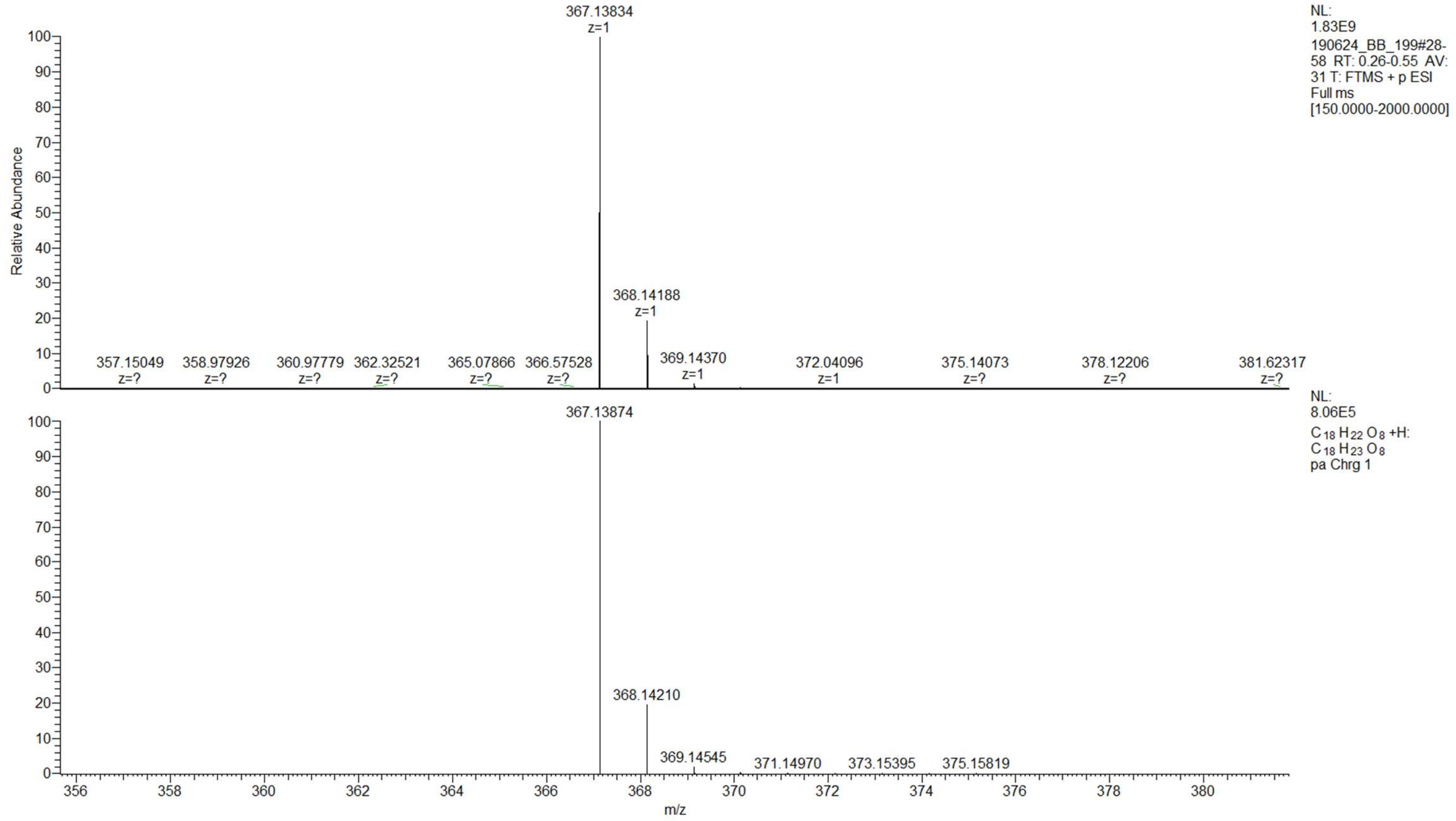

Figure S115. HRMS spectrum for tetraethyl benzene–1,2,4,5-tetracarboxylate (**8**).

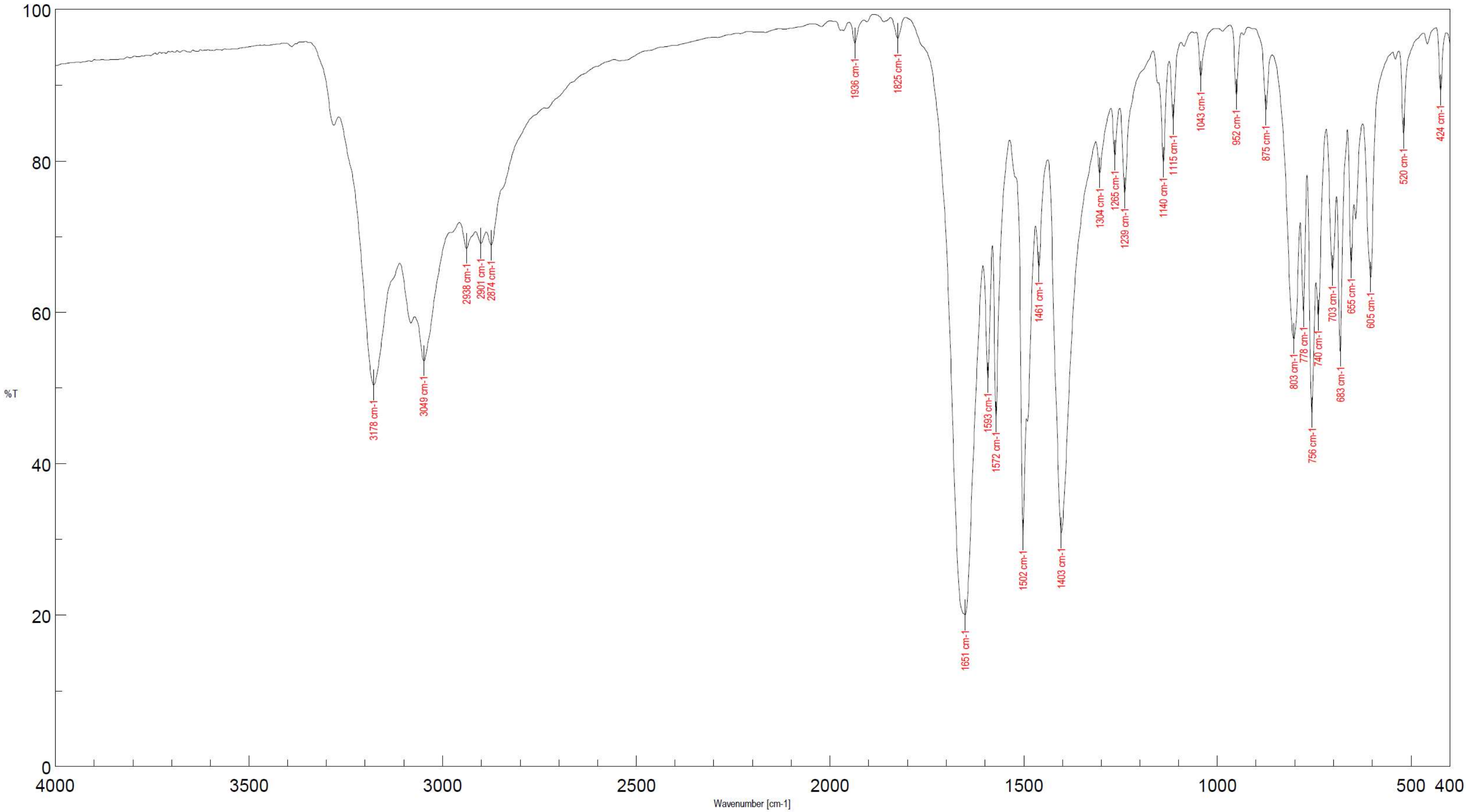

Figure S116. IR spectrum for 5,12-dihydrodibenzo[*b*,*f*][1,4]diazocine–6,11-dione (**3a**).

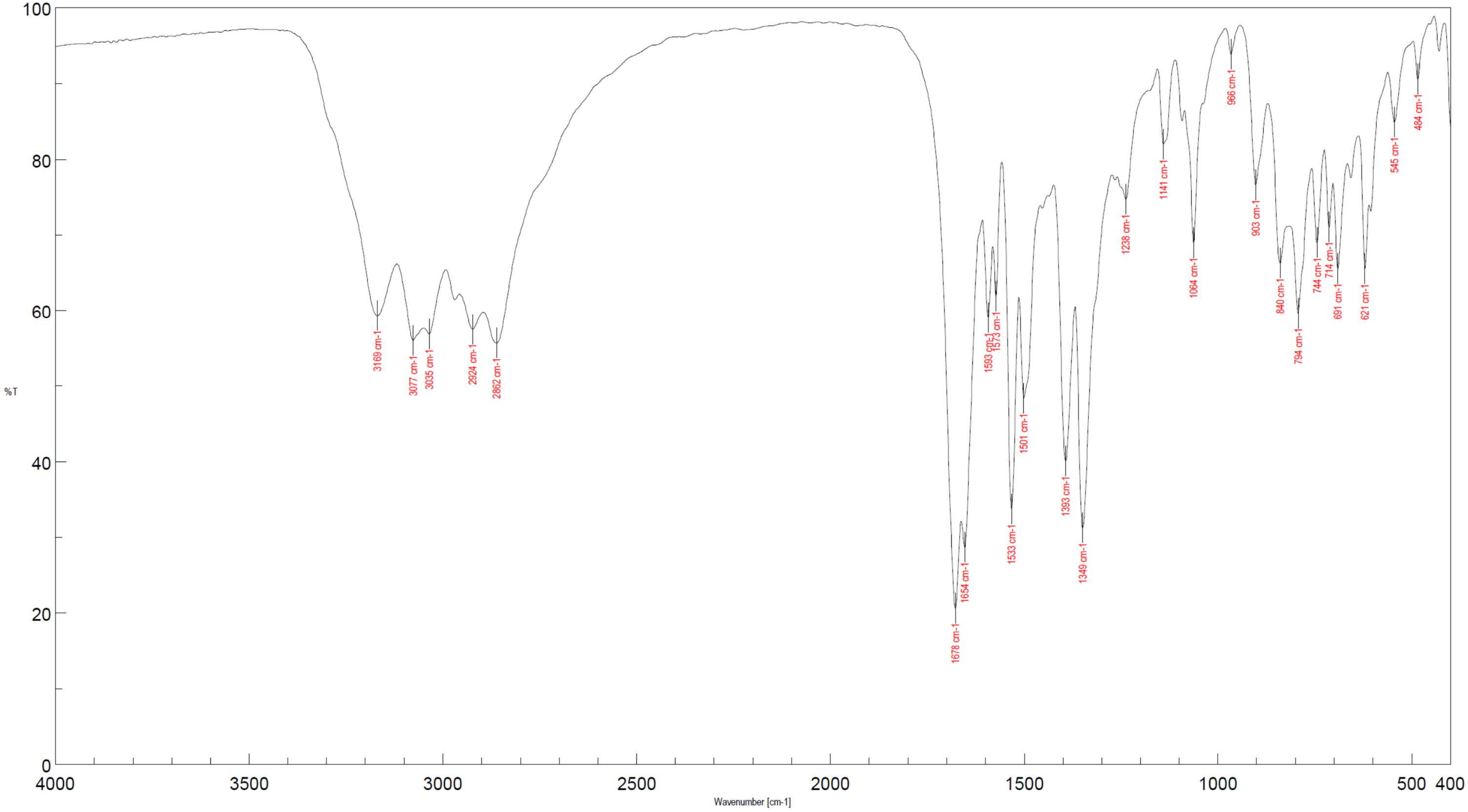

Figure S117. IR spectrum for 2-nitro–5,12-dihydrodibenzo[*b*,*f*][1,4]diazocine–6,11-dione (**3b**).

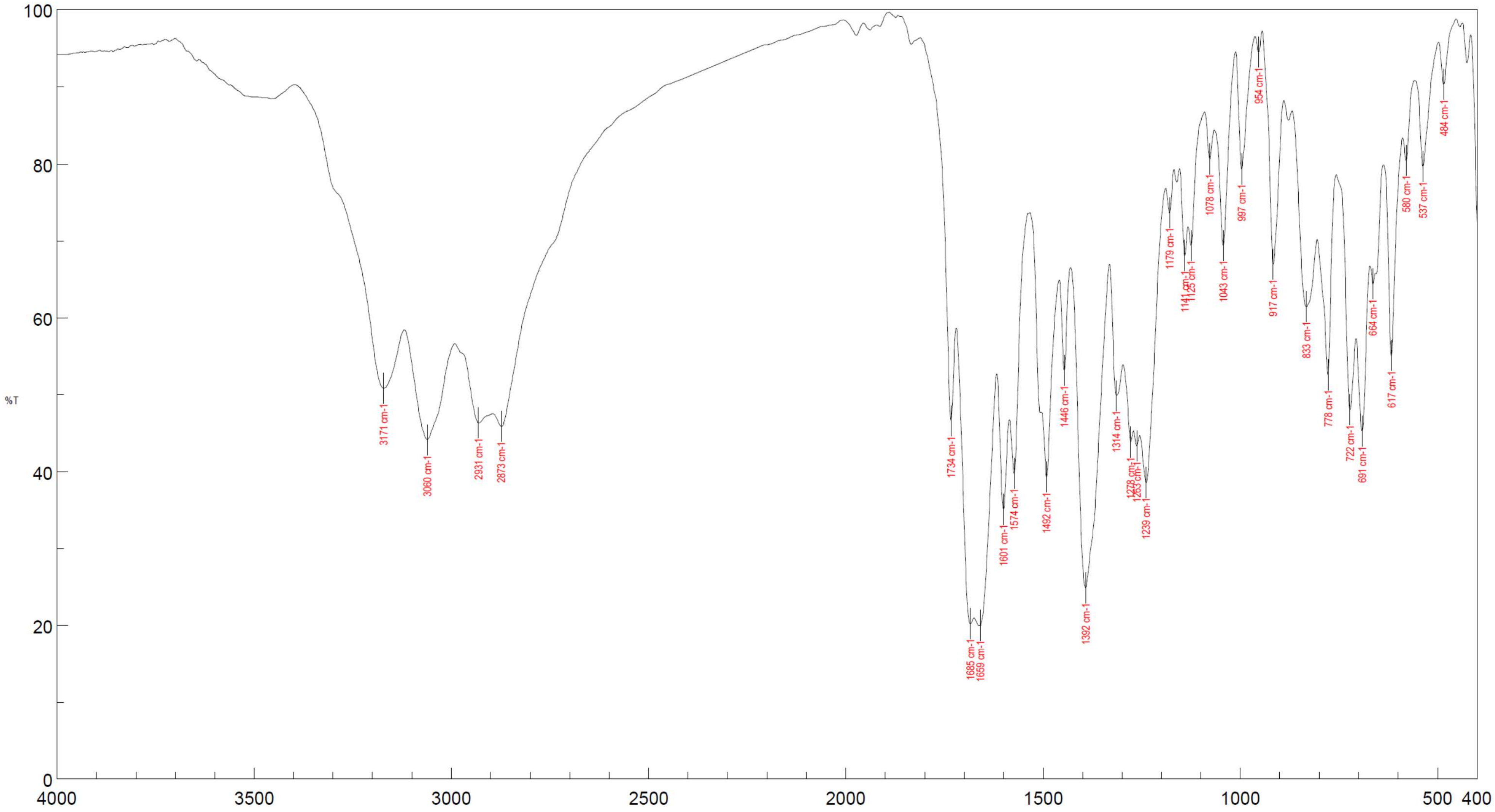

Figure S118. IR spectrum for 2-benzoyl–5,12-dihydrodibenzo[*b,f*][1,4]diazocine–6,11-dione (**3c**).

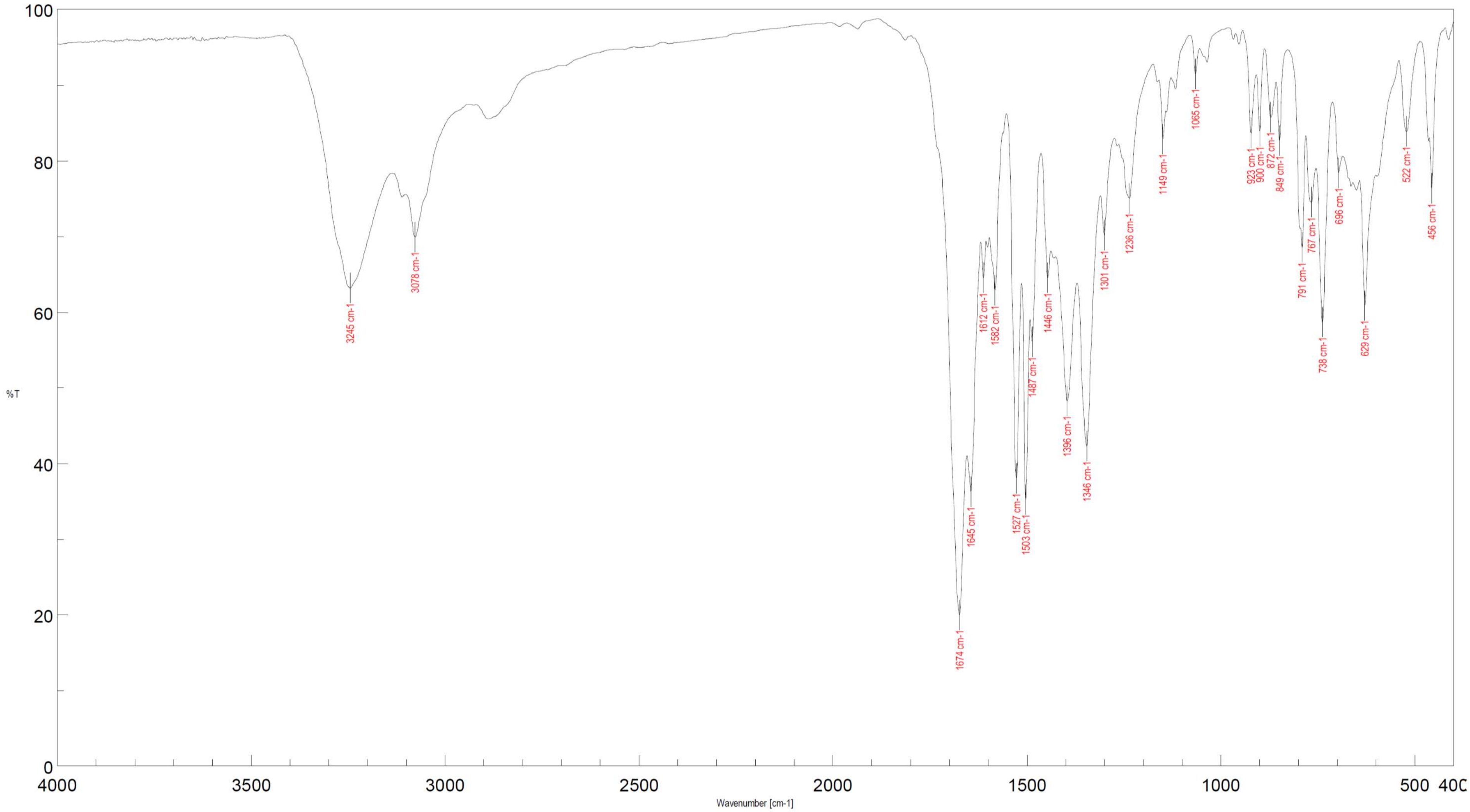

Figure S119. IR spectrum for 8-nitro–5,12-dihydrodibenzo[*b*,*f*][1,4]diazocine–6,11-dione (**3d**).

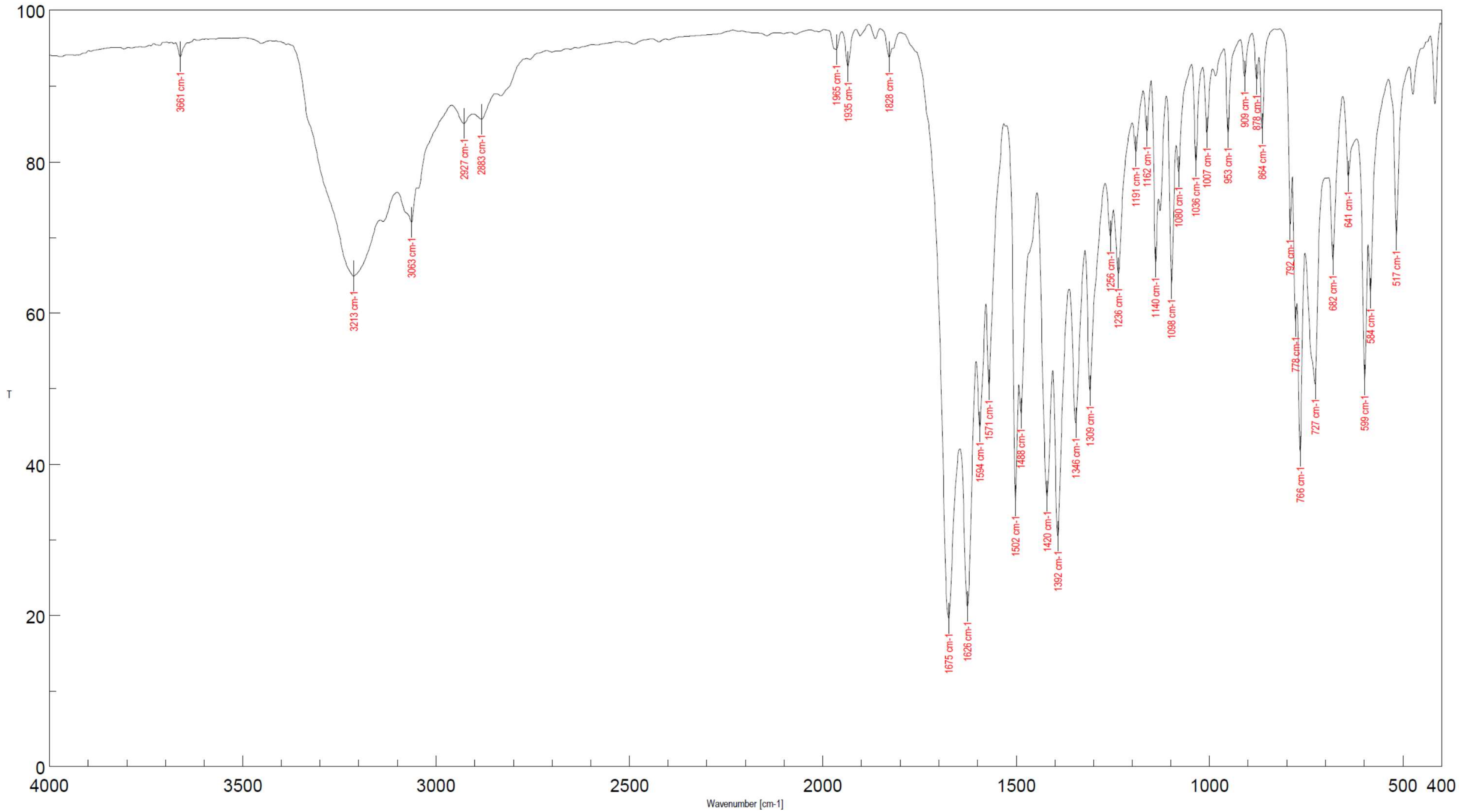

Figure S120. IR spectrum for 5-methyl–5,12-dihydrodibenzo[*b*,*f*][1,4]diazocine–6,11-dione (**3e**).

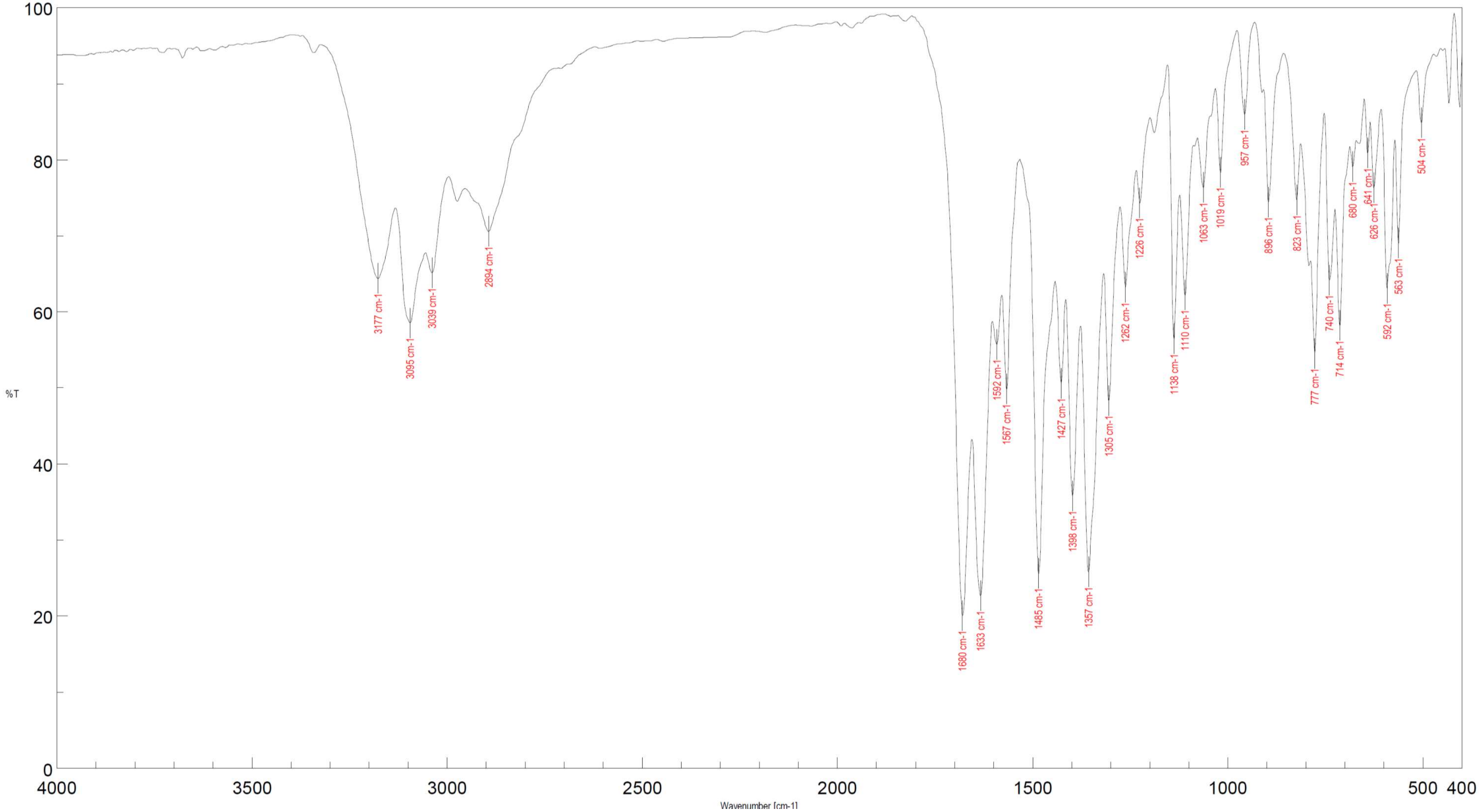

Figure S121. IR spectrum for 2,3-dichloro–5-methyl–5,12-dihydrodibenzo[*b*,*f*][1,4]diazocine–6,11-dione (**3f**).

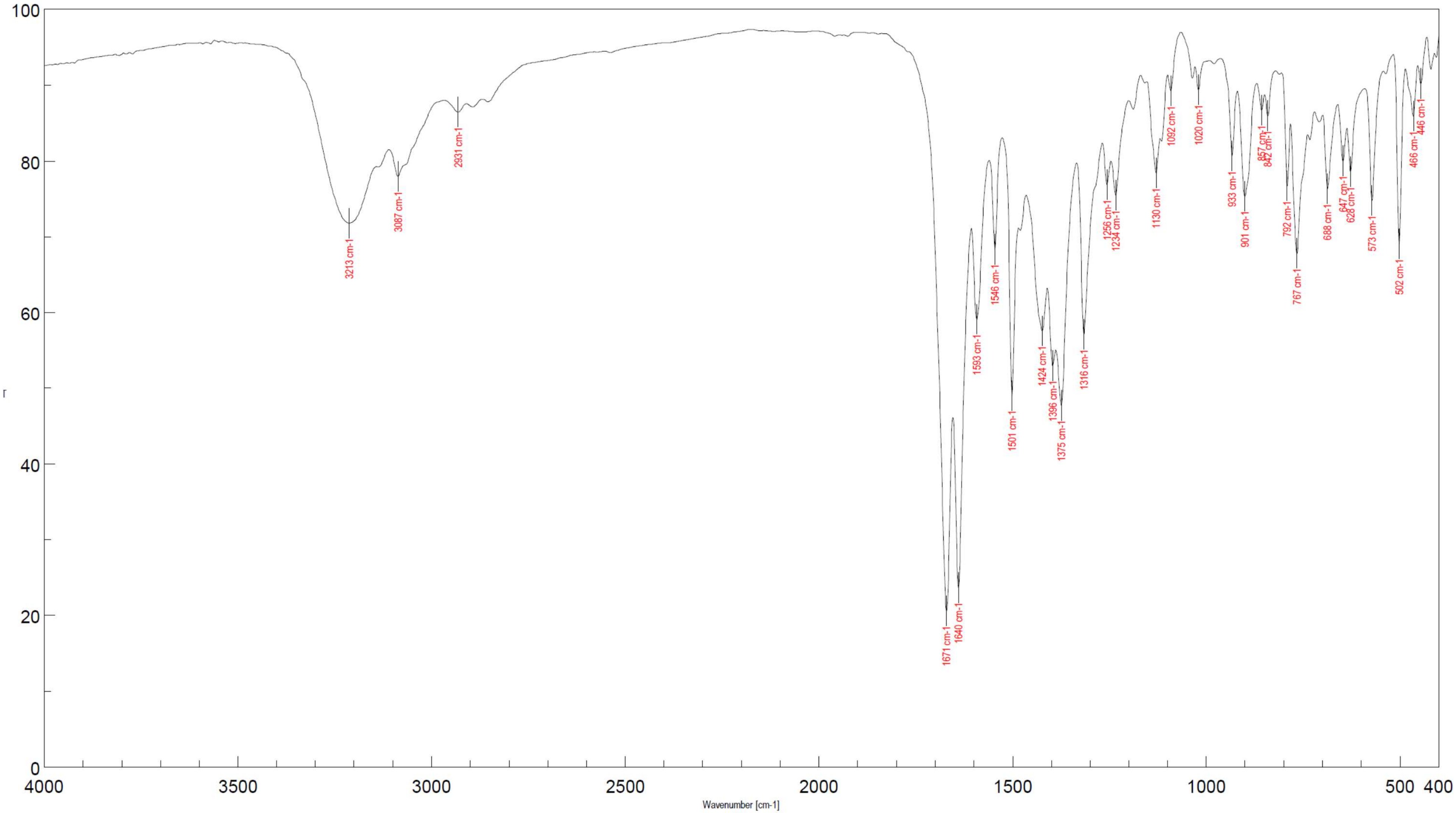

Figure S122. IR spectrum for 8,9-dichloro–5-methyl–5,12-dihydrodibenzo[*b*,*f*][1,4]diazocine–6,11-dione (**3g**).

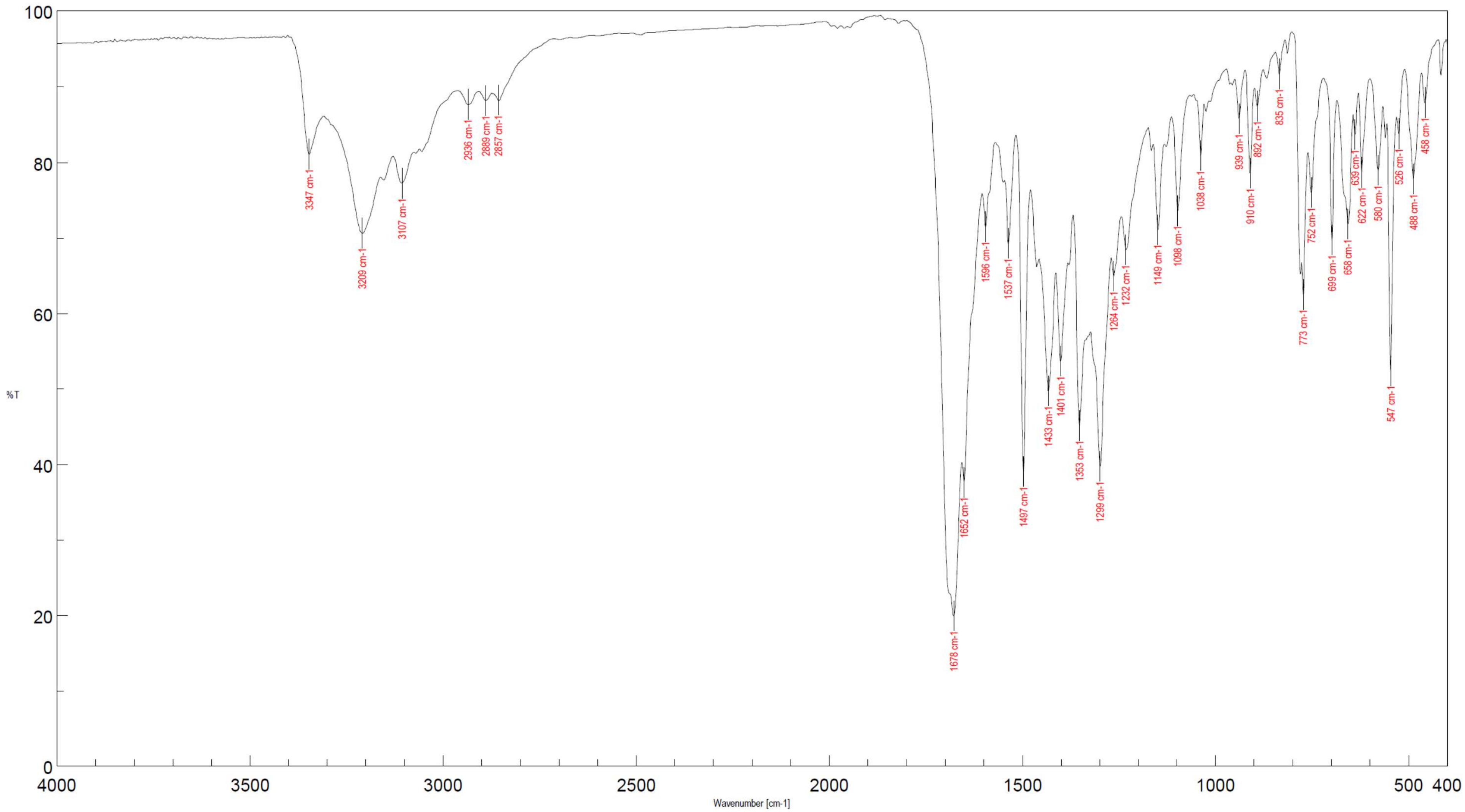

Figure S123. IR spectrum for 7,8,9,10-tetrachloro–5-methyl–5,12-dihydrodibenzo[*b*,*f*][1,4]diazocine–6,11-dione (**3h**).

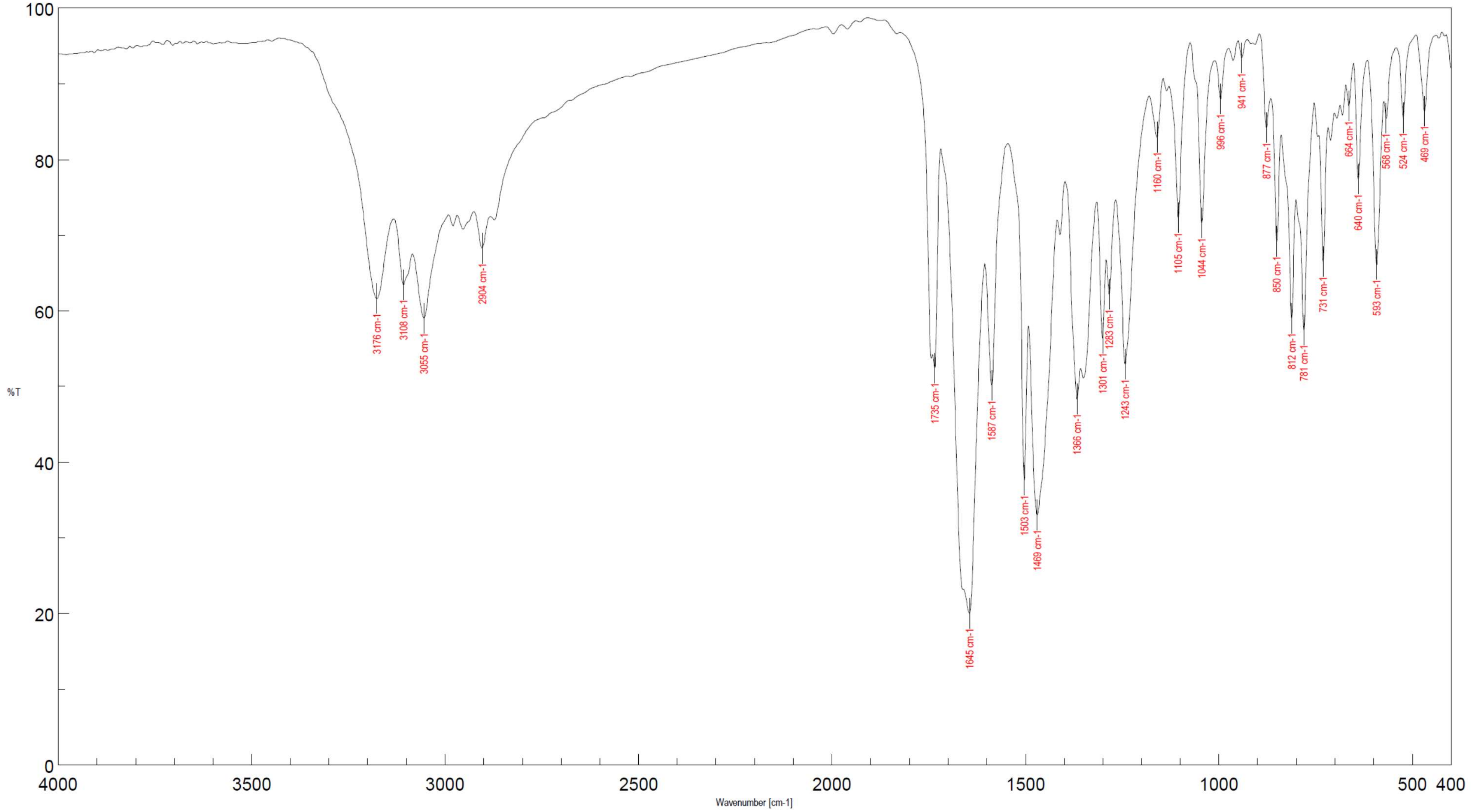

Figure S124. IR spectrum for 5-methyl–5,10-dihydrobenzo[*b*]thieno[3,4-*f*][1,4]diazocine–4,11-dione (**3i**).

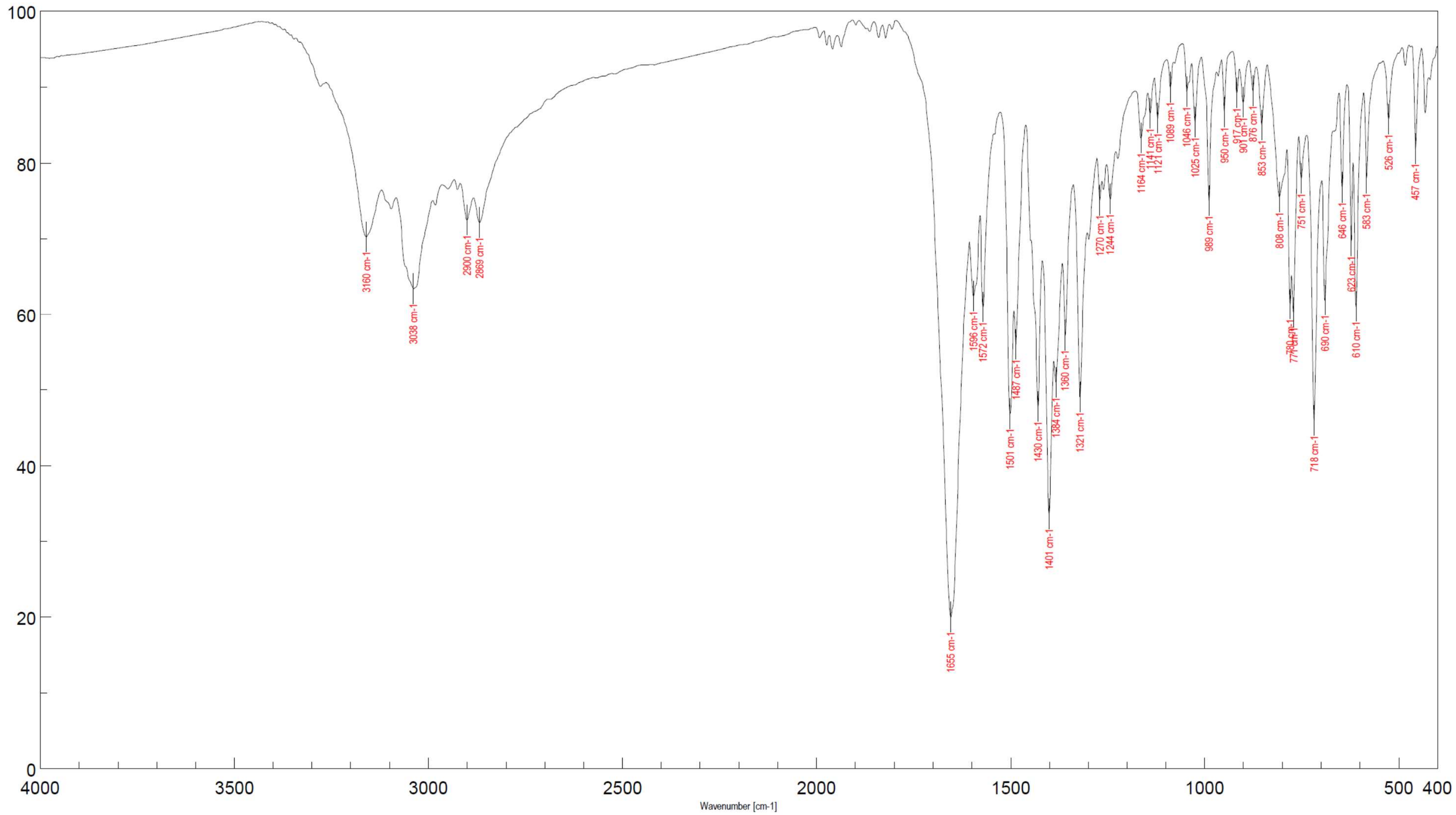

Figure S125. IR spectrum for 5-benzyl–5,12-dihydrodibenzo[*b*,*f*][1,4]diazocine–6,11-dione (**3j**).

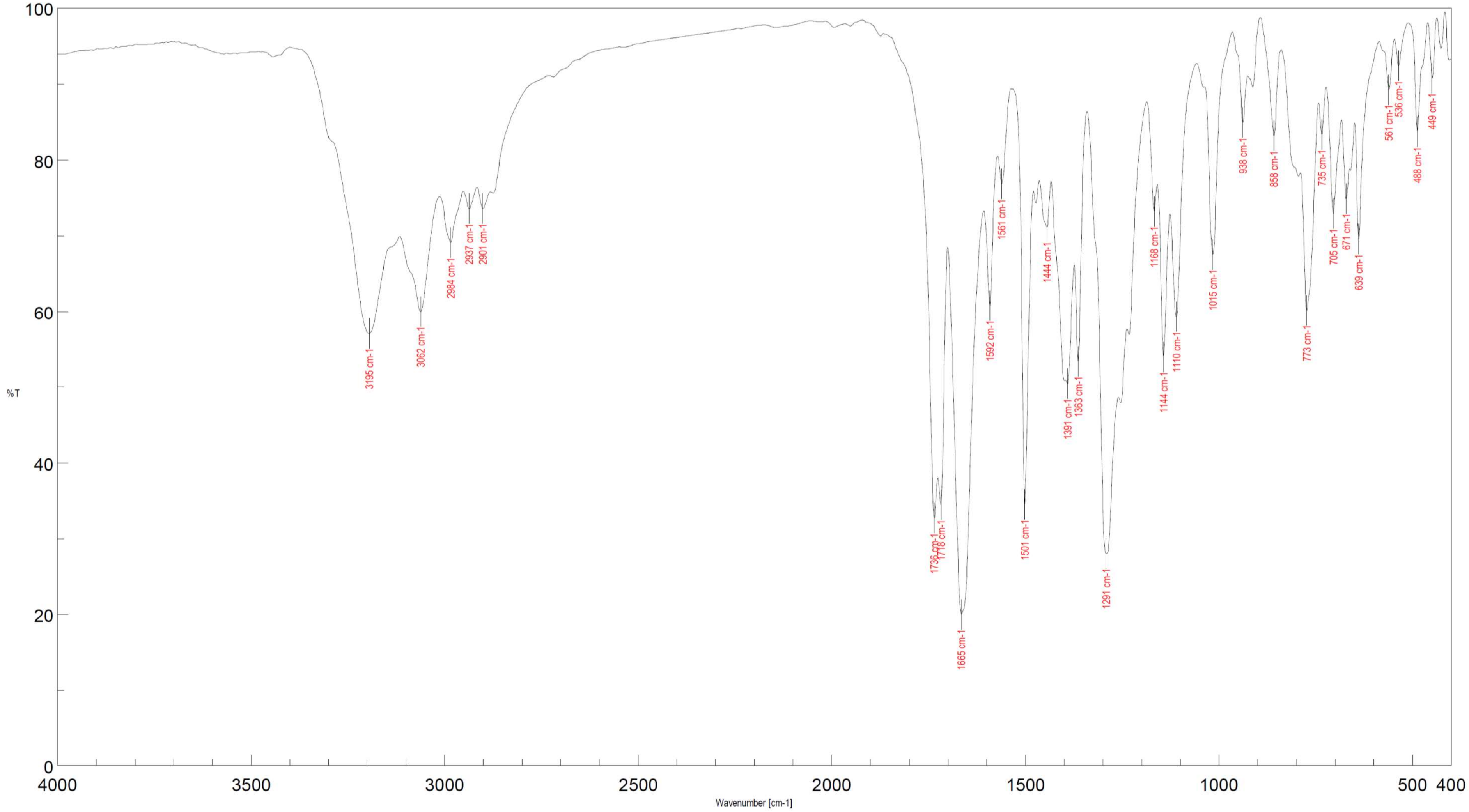

Figure S126. IR spectrum for diethyl 6,11-dioxo–5,6,11,12-tetrahydrodibenzo[*b*,*f*][1,4]diazocine–8,9-dicarboxylate (**3k**).

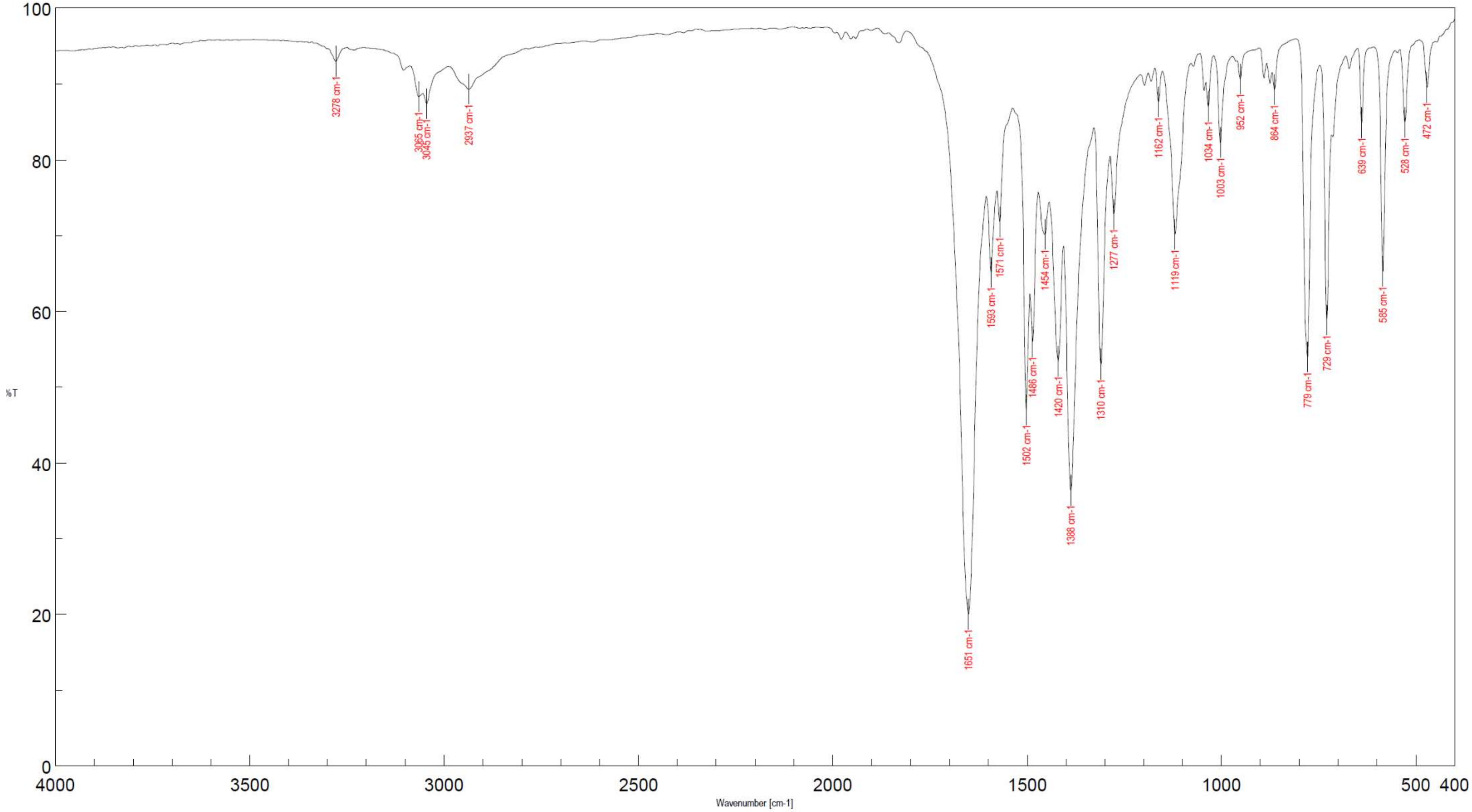

Figure S127. IR spectrum for 5,12-dimethyl–5,12-dihydrodibenzo[*b*,*f*][1,4]diazocine–6,11-dione (**3l**).

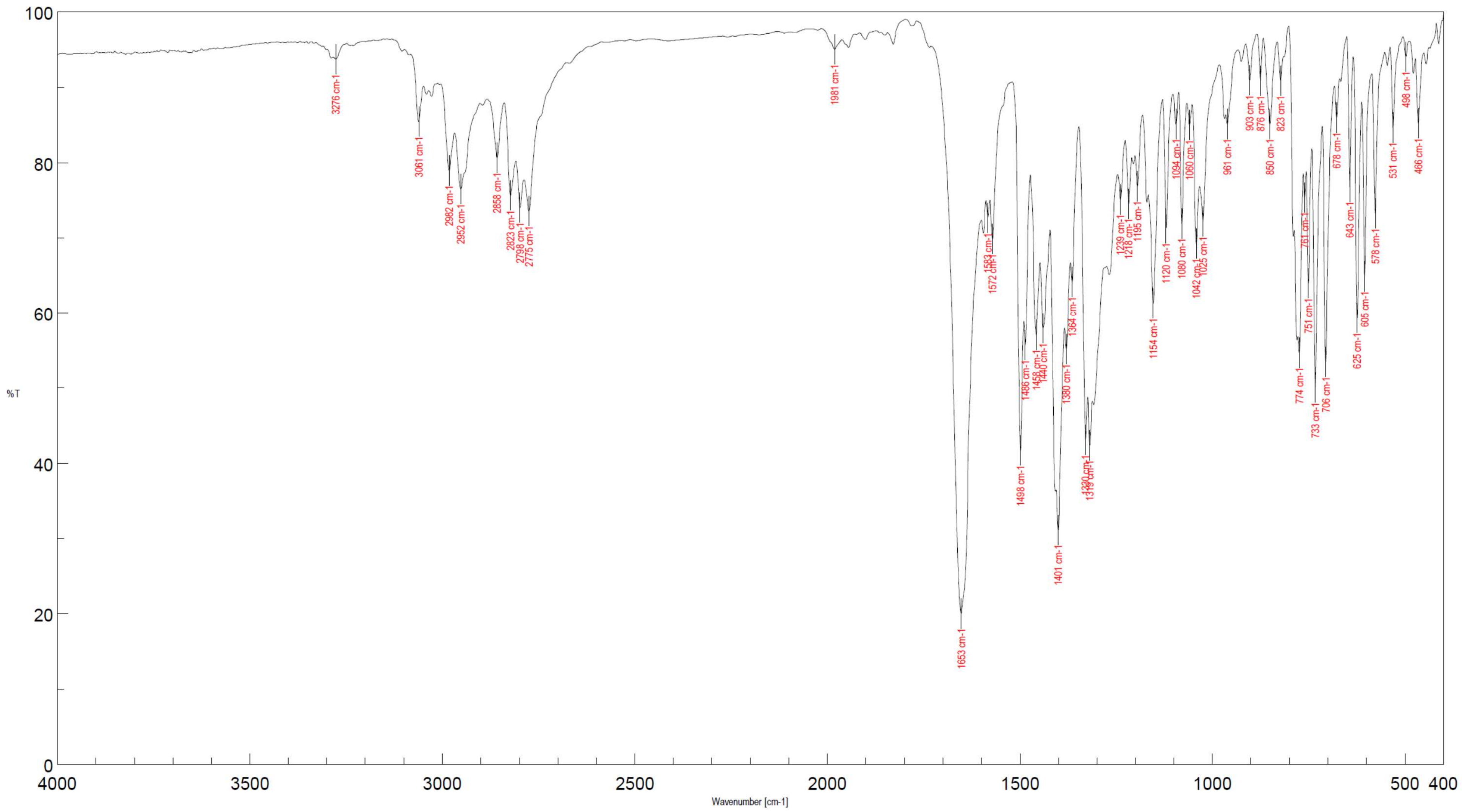

Figure S128. IR spectrum for 5-benzyl–12-(2-(dimethylamino)ethyl)–5,12-dihydrodibenzo[*b*,*f*][1,4]diazocine–6,11-dione (**3m**).

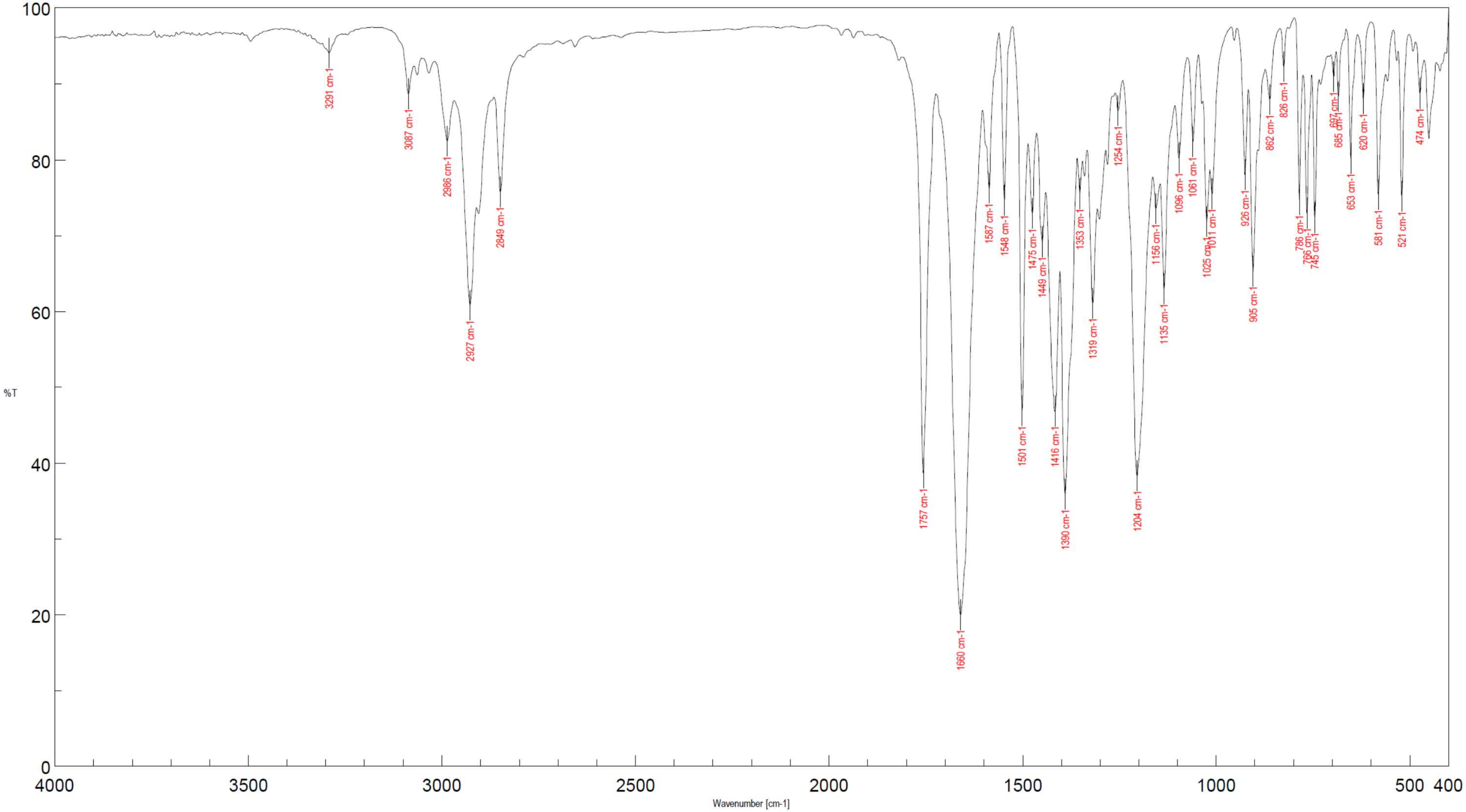

Figure S129. IR spectrum for ethyl 2-(8,9-dichloro–12-methyl–6,11-dioxo–11,12-dihydrodibenzo[*b,f*][1,4]diazocin–5(6*H*)-yl)acetate (**3n**).

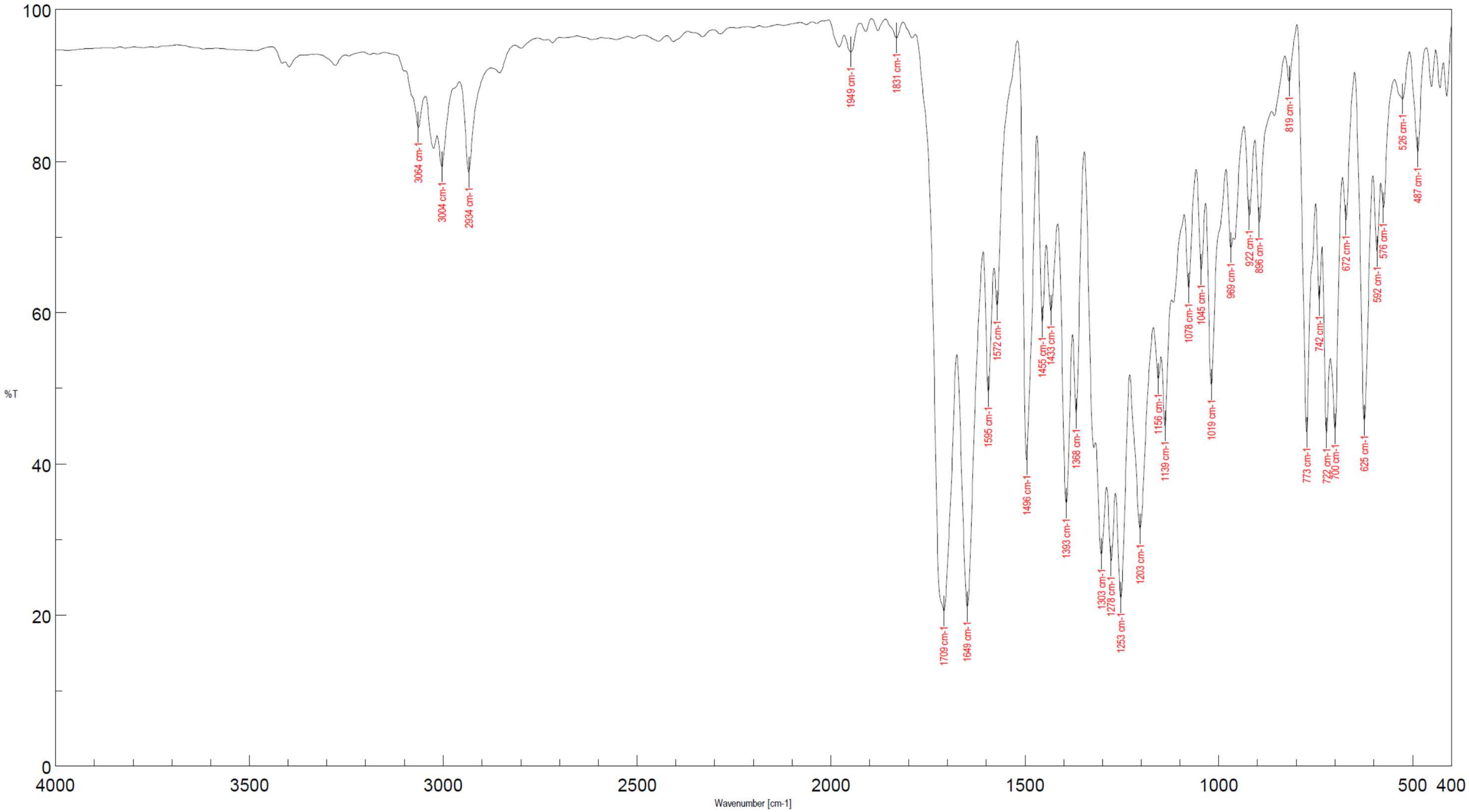

Figure S130. IR spectrum for 5-acetyl–12-benzyl–5,12-dihydrodibenzo[*b*,*f*][1,4]diazocine–6,11-dione (**3o**).

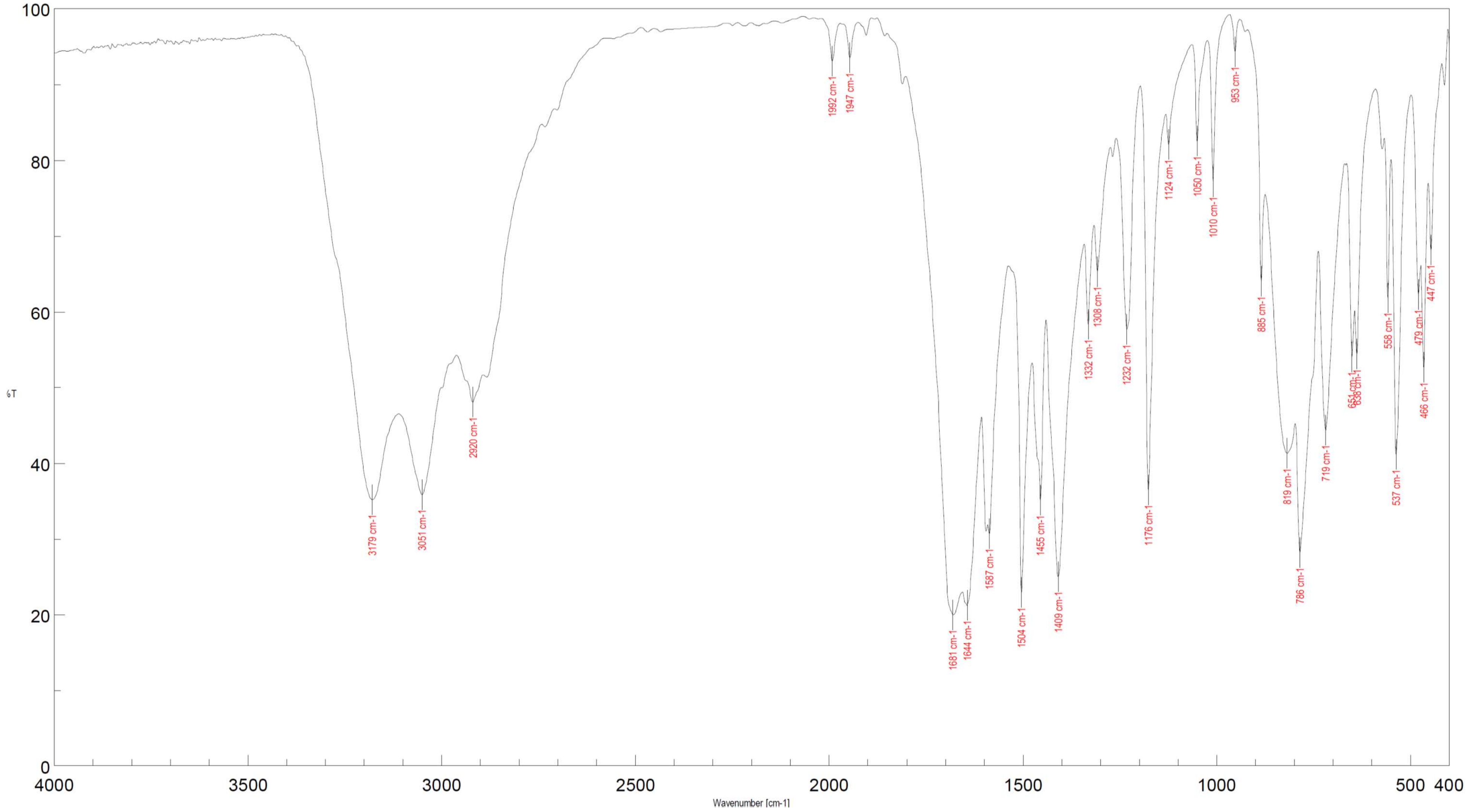

Figure S131. IR spectrum for 1,3,4,6-tetrahydrobenzo[*b*][1,4]diazocine–2,5-dione (**6**).

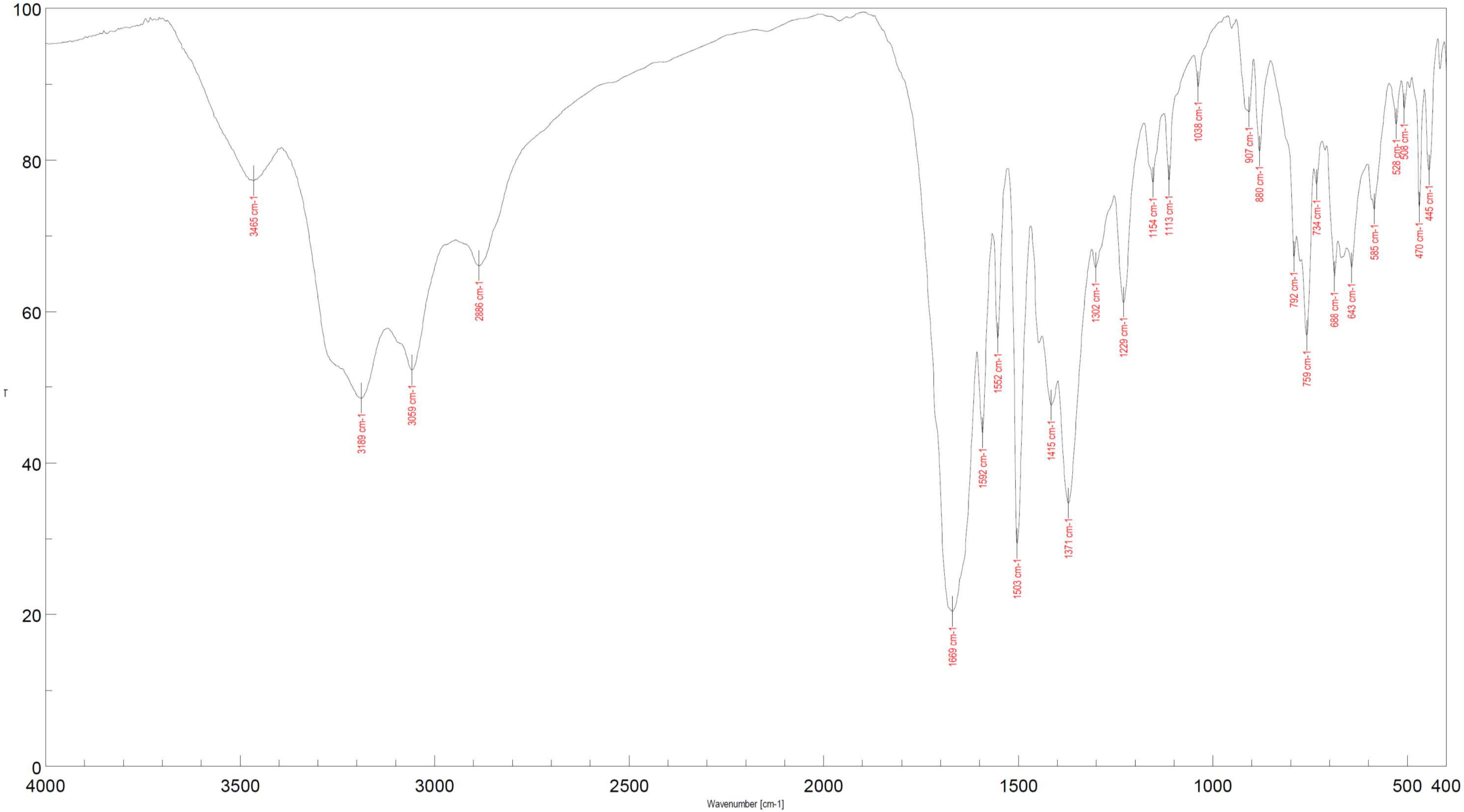

Figure S132. IR spectrum for **9a.**

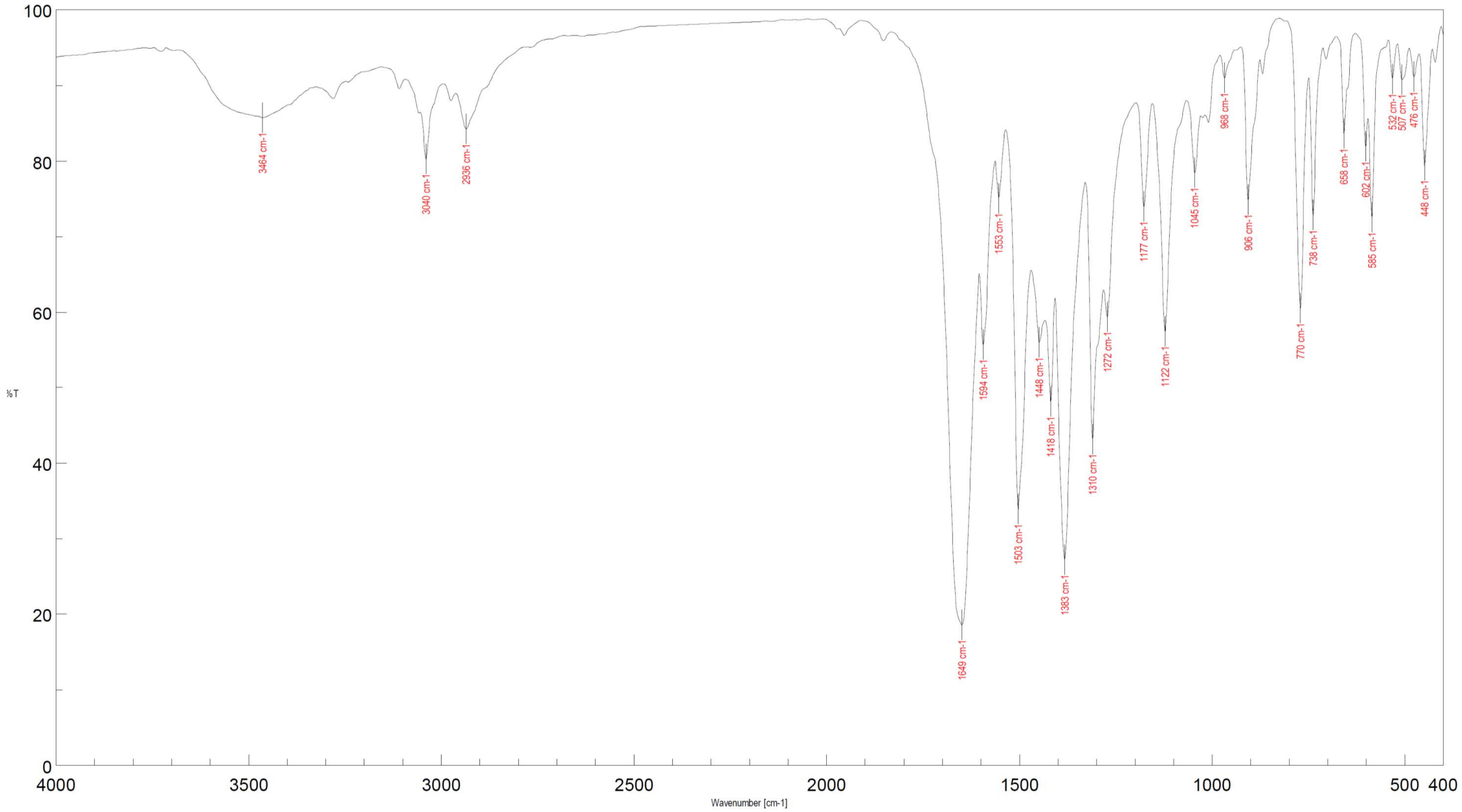

Figure S133. IR spectrum for **9b.**

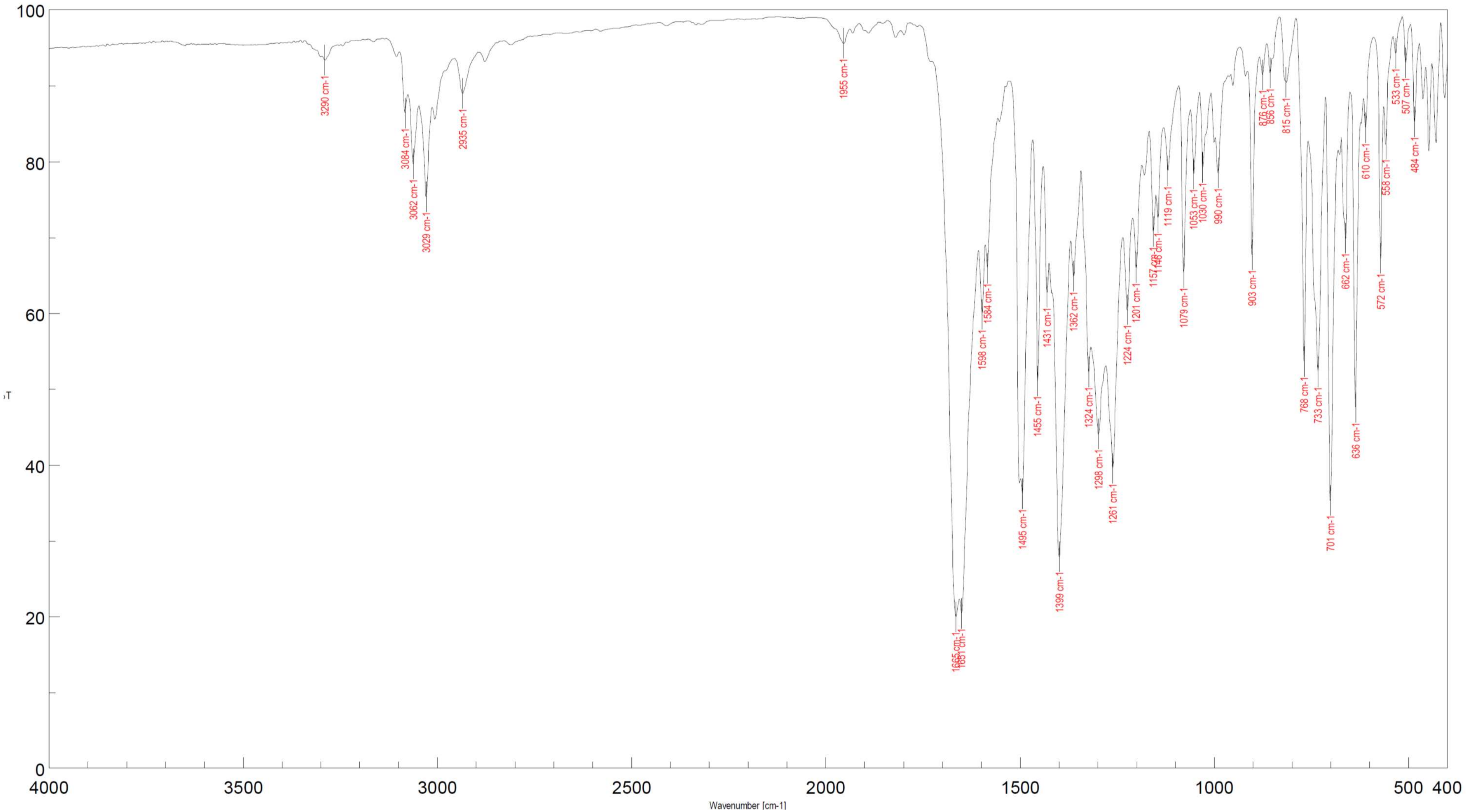

Figure S134. IR spectrum for **9c.**

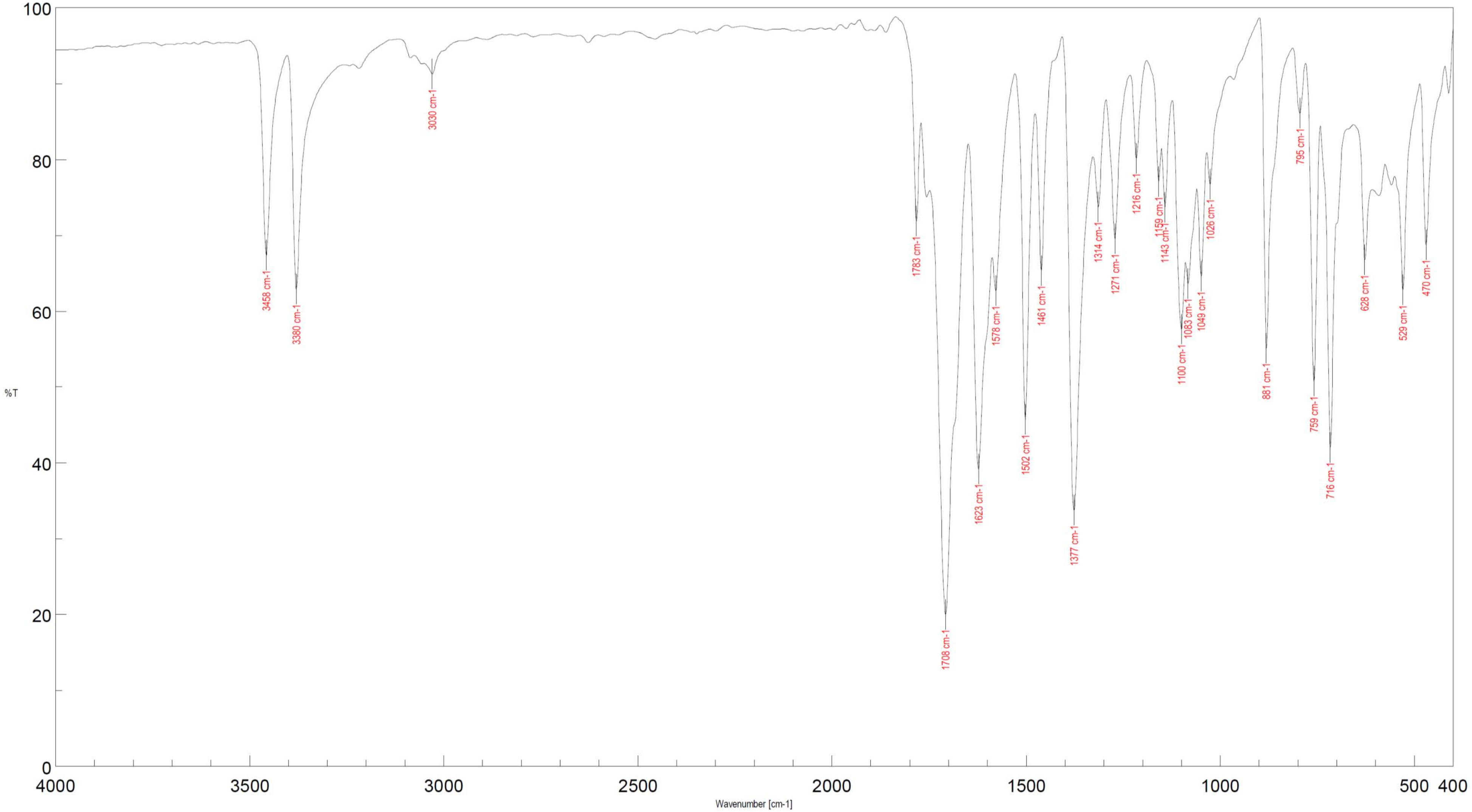

Figure S135. IR spectrum for 2-(2-aminophenyl)isoindoline–1,3-dione (**10**).
